# Photofracking-Assisted Enhancement of Solid-State Photochemical Reactivity: α‑Azido-5-phenyl-2,4-dienoate Derivatives

**DOI:** 10.1021/jacs.5c22335

**Published:** 2026-04-26

**Authors:** Upasana Banerjee, Janaka P. K. Kavikarage, Fiona J. Wasson, W. Dinindu Mendis, Wandana K. S. Henarath Mohottige, Rajkumar Merugu, Anushree Das, Alexis Mack, Nawame Kitil, Savanna Hayes-Bogle, Gabriel D. Patrick, Jack K. Clegg, Durga Prasad Karothu, Panče Naumov, Jeanette A. Krause, Anna D. Gudmundsdottir

**Affiliations:** † Department of Chemistry, 2514 University of Cincinnati, Cincinnati, Ohio 45221, United States; ‡ School of Chemistry and Molecular Biosciences, The University of Queensland, St Lucia, Qld 4072, Australia; § Smart Materials Lab, 167632 New York University Abu Dhabi, PO Box 129188 Abu Dhabi, UAE; ∥ Center for Smart Engineering Materials, New York University Abu Dhabi, PO Box 129188 Abu Dhabi, UAE; ⊥ Research Center for Environment and Materials, Macedonian Academy of Sciences and Arts, Bul. Krste Misirkov 2, MK−1000 Skopje, Macedonia; # Molecular Design Institute, Department of Chemistry, 5894 New York University, 100 Washington Square East, New York, New York 10003, United States

## Abstract

Solid-state photoreactions are unmatched by their liquid or gaseous counterparts for being spatially and temporally controllable and selective; however, these assets usually come with compromised yields―localized surface reactions are favored that generate a passivating layer of photoproducts and shield the crystal interior from reacting. Here, we demonstrate that this inherent limitation could be circumvented by capitalizing on the disintegrative effects of photodynamic crystals, such as cracking and fracturing, which can expose freshly cleaved surfaces and effectively increase the overall conversion. Upon irradiation with natural or artificial visible light, crystals of two 2-azido-5-phenyl-2,4-dienoate derivatives (**1a** and **1b**) are converted into the corresponding 5-phenyl-1*H*-pyrrole-2-carboxylate derivatives (**2a** and **2b**). Crystals were observed to crack and fracture throughout the reaction due to the release of dinitrogen, and the cracking occurs preferentially along planes defined by the weakest intermolecular interactions. Laser-flash photolysis in conjunction with density functional theory calculations confirmed that in both solution and in the solid, the triplet excited states of **1a** and **1b** rearrange to biradicals ^**3**^ **Br1a** and ^**3**^ **Br1b**, which then release dinitrogen to afford biradicals ^3^ **Br2a** and ^3^ **Br2b**; however, in the solid state, the crystal lattice rigidity extends the triplet lifetimes of both **1** and ^3^ **Br1**. This work highlights the benefits of the “photofracking” that stands for photoinduced crystal disintegration, as a convenient strategy that naturally increases the efficiency of the solid-state reactions without external intervention.

## Graphical abstract

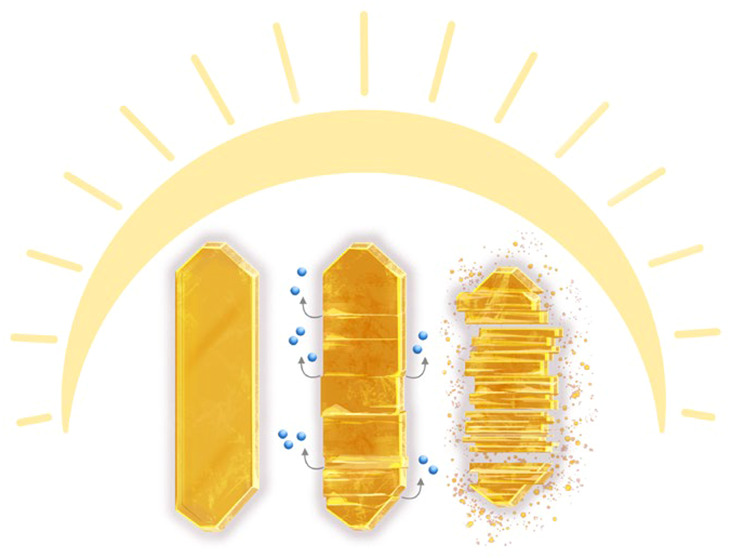

## 1 Introduction

Photochemical reactions are increasingly employed in synthetic chemistry, as they allow the use of sunlight or energy-efficient light-emitting diodes (LEDs) to initiate and drive chemical transformations.^1–7^ Photoreactions in the solid state come with added value by circumventing the necessity to use solvents and are oftentimes endowed with chemical selectivity due to the restrictive nature of the crystalline state for molecular reconfiguration and diffusion. Among other synthetic demonstrations of its advantages, the high selectivity has been cleverly explored in the synthesis of natural products; for example, C–C bonds can be formed stereoselectively by carbon radicals generated adjacently within a crystal lattice owing to hindered rotation during biradical decay.^8–10^ Despite these examples, however, the full synthetic potential of the solid-state reactions does not appear to have been fully exploited yet, with low conversions being one of the most frequently quoted limitations. The difference in the light absorption between the reactant and the product, as well as strong light absorption by the dense solid state, photoproducts tend to be localized on the surface, thereby impeding further reaction. There are numerous examples, mainly dimerizations, where the integrity of the crystal is preserved to appreciably high yields (single-crystal-to-single-crystal photoreactions) due to the small geometric differences between reactants and products.^11–13^ In contrast to these well-studied examples, however, most photoreactive crystals collapse or degrade during reaction, and large structural changes could also lead to melting. At lower temperatures, reconstructive phase transitions cause opacity and thus enhanced light scattering and occasionally domain separation, with the photoproducts sometimes forming as amorphous solids. To address this issue, in practice, solid-state reactions are typically carried out by crushing the crystals and placing them between optically transparent slides to maximize the exposure of the particle surfaces to light. An alternative approach capitalizes on the preparation of nanocrystalline suspensions in water, as nanocrystals provide larger surface area than bulk crystals.^14–16^ However, another approach is based on supramolecular principles, whereby template-switching has been employed to drive solid-state dimerization reactions to high conversions.^17–20^

One of the most exciting recent endeavors is the application of photochemical reactions that use their “smart” capability of converting light into mechanical motion. Historically, these materials were preceded by polymers and elastomers, which have been favored for fabricating smart materials and even adopted for such applications.^21–24^ However, crystals have recently been demonstrated to be able to perform mechanical tasks similar to those achieved by polymeric materials.^25–34^ For example, crystals can rapidly respond to external triggers such as light, heat, and mechanical force, resulting in remarkable effects including long-distance propulsion,^26,35^ twisting,^36^ coiling,^37^ crawling,^38^ bending,^26,36,39^ fracturing,^40,41^ and shattering.^30,42^ These dynamic effects arise from switching between crystal packing arrangements, such as in-phase transitions, or chemical reactions, such as *cis–trans* isomerizations and dimerizations.^43–46^ Such molecular reconfigurations inevitably create strain within the crystal lattice, which is often observable as large-scale crystal movement or disintegration.^47,48^ An alternative and much less-explored approach for inducing motion employs the momentum produced by releasing gas molecules from the crystal lattice. We recently reported the first comprehensive study on crystal disintegration and movement induced by gas release, directly correlating photodynamic behavior with structure for a set of structurally similar compounds.^49^ The work described here underscores the significance of various intracrystalline forces for rationalizing the observed mechanical effects resulting from the release of inert gas molecules. We posit that photodynamic crystals that fracture efficiently when exposed to light can continuously generate new surfaces during irradiation, thereby significantly circumventing the shielding effect imposed by product accumulation at the surface. In cases where the photoproduct is stable toward further irradiation and forms a distinct solid phase, photomechanical fracture can therefore sustain reactivity to full conversion rather than merely delay surface passivation, establishing photofracking as a structure-enabled strategy for overcoming one of the central limitations of solid-state photochemistry.

To test this theory, we studied the ability of crystalline methyl-2-azido-5-phenyl-2,4-dienoate derivatives (**1a** and **1b**) to form the corresponding 5-phenyl-1*H*-pyrrole-2-carboxylate derivatives (**2a** and **2b**) upon irradiation. These systems rely on propulsion via release of gaseous nitrogen and the formation of photoproducts with distinct phases, resulting in sustainable photoreactions. Herein, we demonstrate that azides **1a** and **1b** undergo quantitative conversion in the solid state to form **2a** and **2b**, respectively. However, the two materials exhibit markedly different photodynamic behavior: the surface of **1a** erupts after a short incubation period and then fractures along its shorter axis. In contrast, **1b** expands upon light exposure, before it explodes. We elucidated the reaction mechanism in both crystals and solution using laser-flash photolysis and density functional theory (DFT) calculations. Single-crystal X-ray structure analysis, crystal indexing, and force-field calculations on the respective crystal structures showed that cracking initially occurs along the planes defined by the weakest intermolecular forces, which are disrupted as pressure builds up inside the crystal. Powder X-ray diffraction (PXRD) analysis of the reaction mixtures confirmed that the photoproducts exist in distinct phases, supporting the notion that phase separation contributes to the disintegration and thus to the enhanced reaction efficiency.

## 2 Results and Discussion

### 2.1 Photodynamic Behavior

Upon irradiation with an LED (365, 450, or 532 nm), both single-crystal and polycrystalline samples of **1a** and **1b** were found to be completely converted to the pyrroles **2a** and **2b**, respectively (Scheme 1). We separately confirmed that the photoreaction was driven by the absorption of visible light rather than trace UV light from the LEDs by irradiating **1b** with a green LED (532 nm) through a 400 nm long-pass filter. Notably, the measured absorption spectra of solids **1a** and **1b** extend into the visible region (Figure S9). Under these conditions, the conversion of **1b** to **2b** occurred on a similar time scale as in the unfiltered experiment. To further assess the sustainability of this reaction, crystals of **1a** or **1b** in vials were placed in a snowbank (−10 to −7 °C) and exposed to natural sunlight. Remarkably, complete conversion to the corresponding pyrrole **2a** or **2b** was observed after 22 h of sunlight exposure (Figures S26 and S27). In addition, we demonstrated that nanocrystalline suspensions of **1a** and **1b** in water can be fully converted to photoproducts (Figure 1). We also confirmed with ^1^H NMR spectroscopy that irradiation of **1a** and **1b** in the solid state with a 395 nm LED and in chloroform-*d*_1_ with a 365 nm LED selectively yields **2a** and **2b**, respectively. Finally, the photoreactivity of **1a** and **1b** in acetonitrile was monitored as a function of irradiation time using absorption spectroscopy. The spectra show clear isosbestic points denoting clean transformation of the starting materials into the respective pyrroles (Figures 1A,B and S10–S15). Monitoring the reaction progress by UV–Vis spectroscopy in solution revealed that irradiation with longer-wavelength light (450 nm or 532 nm) also drives the reaction, albeit at slower rates, consistent with the low but nonzero absorbance of **1a** and **1b** in this region. Similarly, irradiation of nanocrystalline suspensions of **1a** and **1b** resulted in complete conversion into the corresponding products, although the absorption bands were expectedly affected by the physical state of the sample (Figure 1C,D). Importantly, as azides **1a** and **1b** absorb light at wavelengths longer than those of **2a** and **2b**, the photoproduct formation does not prevent the starting materials from absorbing light to continue reacting.

**Figure 1:**
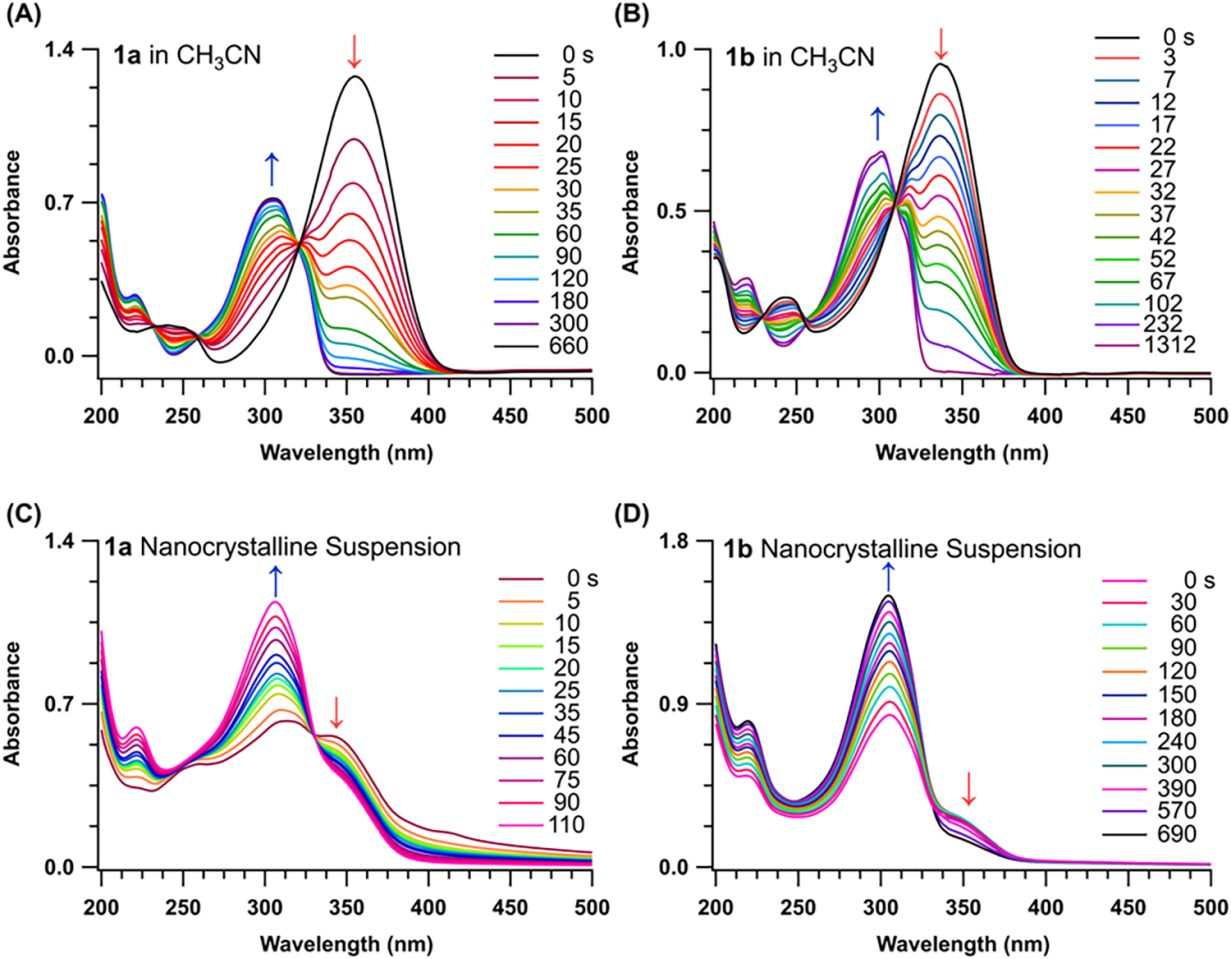
Absorption spectra as a function of irradiation time for (A) **1a** and (B) **1b** in acetonitrile (365 nm LED) and nanocrystalline suspensions of (C) **1a** and (D) **1b** in water (395 nm LED). Absorption λ_max_ for **1a**, **1b**, **2a**, and **2b** are ∼360, 337, 300, and 301 nm in acetonitrile solutions and in nanosuspension 345/314, 355/306, 307, and 305 nm respectively.

**Scheme 1:**
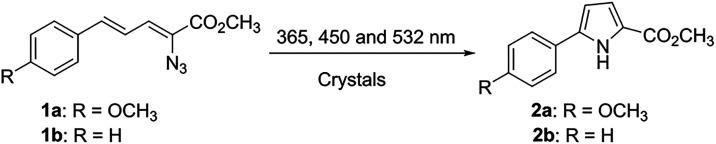
General Photochemical Reaction and Products Formed from Crystals of **1a** and **1b**

The behavior of the crystals of **1a** during exposure to white light was observed and recorded by using an optical microscope. Initially, the transparent crystals turned white and cracked across their short axes. The small crystalline fragments continued to shatter until a crystalline powder of **2a** was formed (Figure 2A,B and Videos 1 and 2). Crystals with imperfect surfaces, such as those to which smaller satellite crystals adhered, also fractured along their short axes, while the smaller pieces were rapidly dislodged from the surfaces. Increased magnification revealed that the opaqueness was caused by the shattering of the small crystals. As irradiation continued, these small crystals were ejected from the surface, and more crystal fragments emerged, followed by further cracking until the crystal was completely transformed into a fine powder of a polycrystalline material. When crystals of **1a** were covered with oil or solvent during irradiation, release of gas bubbles was observed concurrent with the crystals turning opaque and cracking along their short axes (Figure 2C and Video 3).

**Figure 2:**
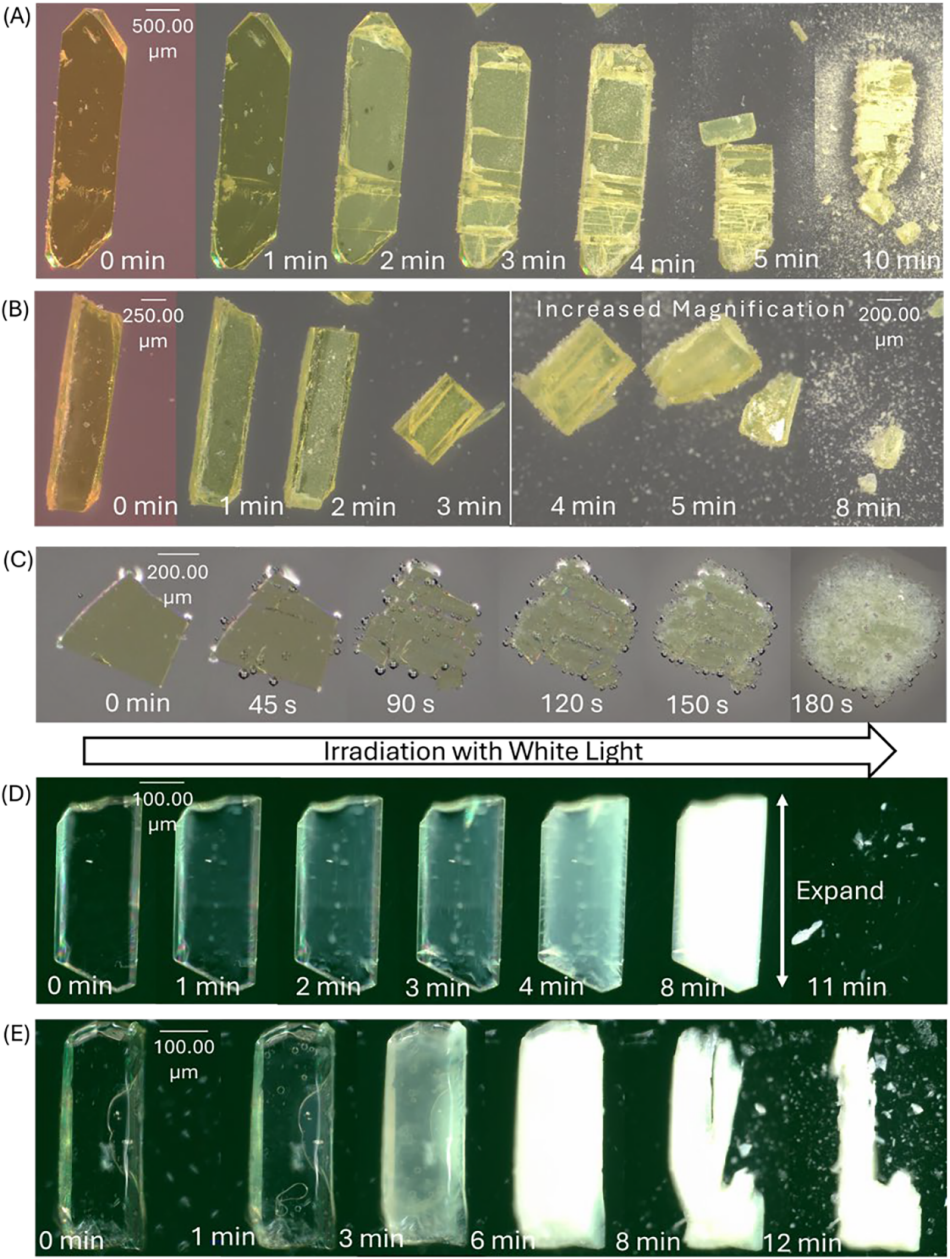
Digital microscope images of crystals of **1a** in (A, B) air or (C) mineral oil and **1b** in (D) air or (E) with the crystal surface coated by a thin layer of methanol during irradiation with white light.

Using microscope white light, we also irradiated transparent crystals of **1b** that were selected to be devoid of any noticeable defects. Initially, short cracks formed along the long axis of each crystal. As irradiation continued, cracking along the long axis increased, causing the crystal to turn opaque and visibly expand. Simultaneously, horizontal cracks appeared along the crystal edges. After becoming fully opaque, the crystals exploded into small pieces, which continued to react until complete conversion into a powder of **2b** was achieved (Figure 2D and Video 4). When the crystals of **1b** were covered with oil or solvent, gas bubbles were released from the crystal surfaces during irradiation. Crystals were grown from an ethyl acetate/hexane mixture. Immediately before irradiation, the crystal surfaces were lightly wetted with methanol to enable clear visualization of bubble formation; the compounds exhibit only minimal solubility in methanol at ambient temperature. Initially, gas bubbles emerged from the defect sites generated within the crystals. With continued irradiation, more gas bubbles were released from the crystal, from cracks along the axis, and the crystal slowly became opaque and splintered (Figure 2E, and Videos 5 and 6). Crystals having defects from the crystallization process behave similarly, although the initial fracturing occurs preferentially at the defects. Thus, the crystals of **1a** and **1b** both react to full conversion because they crack and fracture continuously upon exposure to light; however, disparate photodynamic behaviors are observed for the two compounds. The crystals of **1b** expand significantly before exploding, suggesting that they are more flexible than the crystals of **1a**, which begin to fracture after a short exposure time.

### 2.2 Laser-Flash Photolysis

To determine the reaction mechanism for the formation of **2** from **1**, we performed laser-flash photolysis^50^ in solution and nanocrystalline suspensions. Photolysis of **1a** in argon-saturated acetonitrile resulted in a transient absorption with a maximum absorption between 390 and 420 nm (Figure 3A). As this transient decayed, a weaker and broader absorption band formed between 500 and 600 nm. The lifetime of the initial transient was ∼7 ns (*k* = 1.36 × 10^8^ s^–1^, 400 nm; Figure 3B), whereas the transient at longer wavelengths formed with a rate constant *k* = 2.83 × 10^6^ s^–1^ (τ ∼ 353 ns; Figure 3C) and decayed with *k* = 3.45 × 10^5^ s^–1^ (τ ∼ 3.0 μs) (Figure 3D). In air- and oxygen-saturated acetonitrile, the transient at longer wavelengths was quenched, whereas the transient at 400 nm only became slightly shorter lived. We assigned the transient with λ_max_ at 400 nm to the triplet excited state (T) of **1a** based on the comparison to its calculated spectrum (Figure 4A), which has major electronic transitions at 393 (*f* = 0.1673) and 423 nm (*f* = 0.6749). Additional support for this assignment was provided by the lifetime of the transient, as triplet vinylnitrenes generally have lifetimes on the microsecond time scale.^51–54^ The broad transient at longer wavelengths was assigned to ^3^ **Br2a** based on the comparison to its calculated spectrum (Figure 4B), which has major electronic transitions at 348 (*f* = 0.0535), 356 (*f* = 0.048), 482 nm (*f* = 0.0201), and 510 nm (*f* = 0.004). Thus, upon irradiation, **1a** forms the T of **1a**, which decays to ^3^ **Br1a**. However, this transient is not observed directly with the absorption of ^3^ **Br2a** being observed instead. Laser-flash photolysis of **1b** in argon-saturated acetonitrile resulted in a broad spectrum with λ_max_ values at ∼390 and ∼525 nm (Figures S28–S29). The transient at ∼390 nm decayed with *k* = 1.52 × 10^8^ s^–1^ (τ ∼ 7 ns). At 525 nm, the transient was formed with *k* = 9.12 × 10^6^ s^–1^ (τ ∼ 110 ns) and decayed with *k* = 1.85 × 10^6^ s^–1^ (τ ∼ 538 ns). Analogous to **1a**, the short-lived transient at 400 nm was assigned to T of **1b** and the longer-lived transient at 525 nm was assigned to ^3^ **Br2b**.

**Figure 3:**
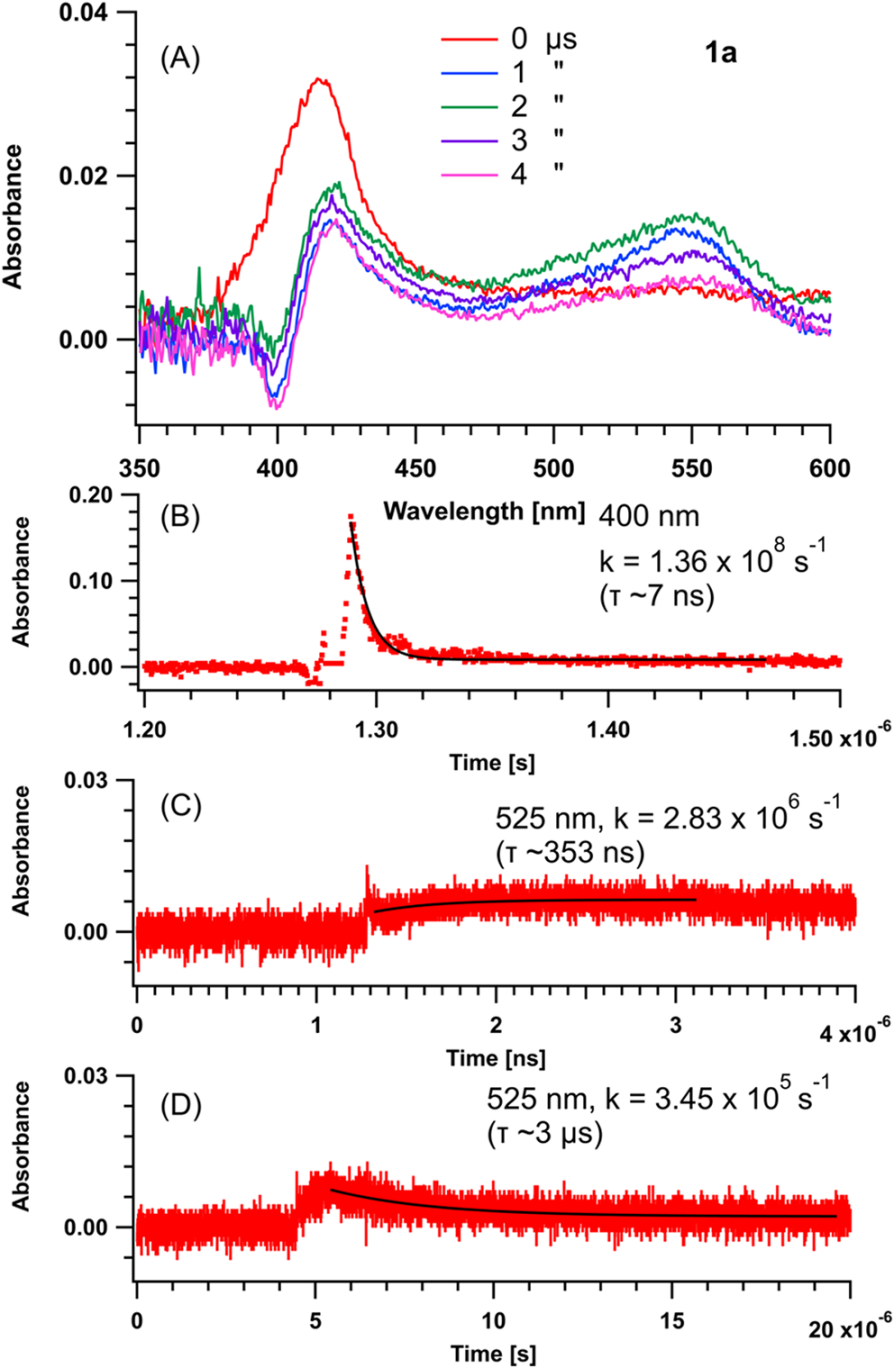
(A) Transient spectra obtained by laser-flash photolysis of **1a** in argon-saturated acetonitrile. Kinetic traces obtained at (B) 400 nm, (C) 525 nm on a 4 μs time scale, and (D) 525 nm on a 20 μs time scale.

**Figure 4:**
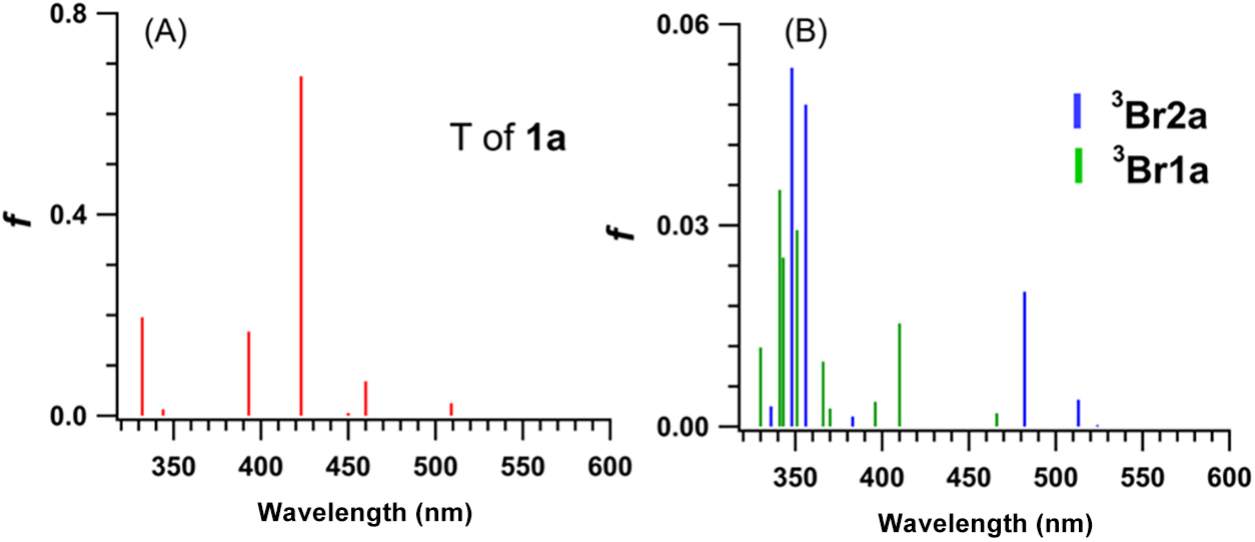
TD-DFT-calculated (B3LYP-D3/6–31+G­(d)) spectra of (A) T of **1a** (red sticks), (B) ^**3**^ **Br1a** (green sticks), and ^**3**^ **Br2a** (blue sticks).

Laser-flash photolysis of a nanocrystalline suspension of **1a** revealed that the transient due to T of **1a** was longer lived in nanocrystals than in acetonitrile (Figures S30–S32). Specifically, T of **1a** decayed with *k* = 1.4 × 10^7^ s^–1^ (*t* ∼ 72 ns) in nanocrystals. In addition, both the transient corresponding to T of **1a** and the broad absorption due to the formation of ^3^ **Br2a** appeared at longer wavelengths. Following the decay of T of **1a**, the transient corresponding to ^3^ **Br2a** was formed at ∼550 nm with *k* = 1.88 × 10^6^ s^–1^ (τ ∼ 532 ns) and then decayed with *k* = 7.49 × 10^5^ s^–1^ (τ ∼ 1.33 μs). Similar results were obtained for laser-flash photolysis of a nanocrystalline suspension of **1b**, with the transient at ∼400 nm being longer lived in nanocrystals (*k* ∼ 9.81 × 10^6^ s^–1^; τ ∼ 101 ns) than in acetonitrile (Figures S33–S36). The transient at longer wavelengths (450 and 500 nm) was formed with *k* = 1.73 × 10^6^ s^–1^ (τ ∼ 577 ns). Finally, we obtained a transient spectrum of the nanocrystalline suspension of **1b** using a PMT detector, which allowed us to observe the transient absorption below 380 nm. The transient absorption at 340 nm was best fitted as a biexponential function, and the resulting rate constants of 9.81 × 10^6^ s^–1^ and 6.15 × 10^5^ s^–1^ (τ ∼ 1.6 μs) corresponded to the rates of decay for T of **1b** and ^3^ **Br2b**, respectively. Based on the laser-flash photolysis and product study results, we propose a mechanism for **1** forming **2** in solution and in the solid state (Scheme 2). In the solid state, the lifetime of T of **1** is significantly longer than that in solution, and the rate of forming ^3^ **Br2** is also slower. Therefore, we suggest that the restriction of movement in the solid state causes both T of **1** and ^3^ **Br1** to be longer lived in nanocrystals than in solution. Finally, PXRD analysis of the nanosuspension of **1b**, performed as described previously,^55^ yielded a diffraction pattern consistent with that calculated from the single-crystal structure (CCDC 2352526), confirming that the nanosuspension retains the bulk crystal packing (Figure S66).

**Scheme 2:**
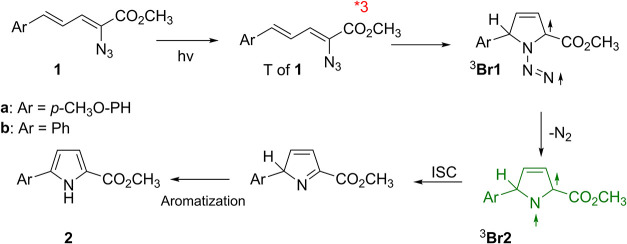
Proposed Mechanism for the Photochemical Conversion of **1** into **2**

### 2.3 Computational Modeling

To support the reaction mechanism shown in Scheme 2, we optimized the structures of **1**, T of **1**, ^3^ **Br1**, ^3^ **Br2**, ^**3**^ **1N**, and **2** for both derivatives (**1a** and **1b**) using DFT (B3LYP-D3/6-31+G­(d,p)).^56–58^ Optimization of **1** revealed the presence of several minimal energy conformers differing by a few kcal mol^‑1^. The TD-DFT-calculated vertical energies of S_1_ of **1** and T_1_ of **1** are located 76 and 38 kcal mol^‑1^, respectively, above the S_0_ of **1**. The lowest optimized structure of T_1_ of **1** is located 35 kcal mol^‑1^ above its S_0_ (Figure 5A). Spin-density calculations located the unpaired electrons on the vinylic bonds with smaller spin densities observed on the phenyl group and ester oxygen atoms (Figure 6). The optimized structures of ^3^ **Br1b** and ^3^ **Br2b** with a N_2_ molecule are located 31 and 10 kcal mol^‑1^, respectively, above the S_0_ of **1b** (Figure 5A). Spin-density calculations for ^3^ **Br1b** and ^3^ **Br2b** showed that the unpaired electrons are located on the terminal N atom, adjacent carbon atoms, and C atom next to the phenyl group (Figure 6), indicating biradical structures. The triplet excited state of the azido moiety (T_A_) of **1b** is located 34 kcal mol^‑1^ above the S_0_ of **1b**. Furthermore, spin-density calculations of T_A_ of **1b** revealed that the unpaired electrons are mainly located on the α- and γ-N atoms (0.48 and 0.73, respectively; Figure 6), as expected for a triplet azido moiety. In addition, we optimized the structure of triplet vinylnitrene ^3^ **1aN**, resulting in two minimal energy conformers, **A** and **B**. Nitrene ^3^ **1bN-A** and an N_2_ molecule are 5 kcal mol^‑1^ more stable than S_0_ of **1b** (Figure 5B). As expected, the calculated spin densities of vinylnitrene ^3^ **1bN** are located on the N atom and the vinylic C atoms next to the phenyl ring (Figure 6).

**Figure 5:**
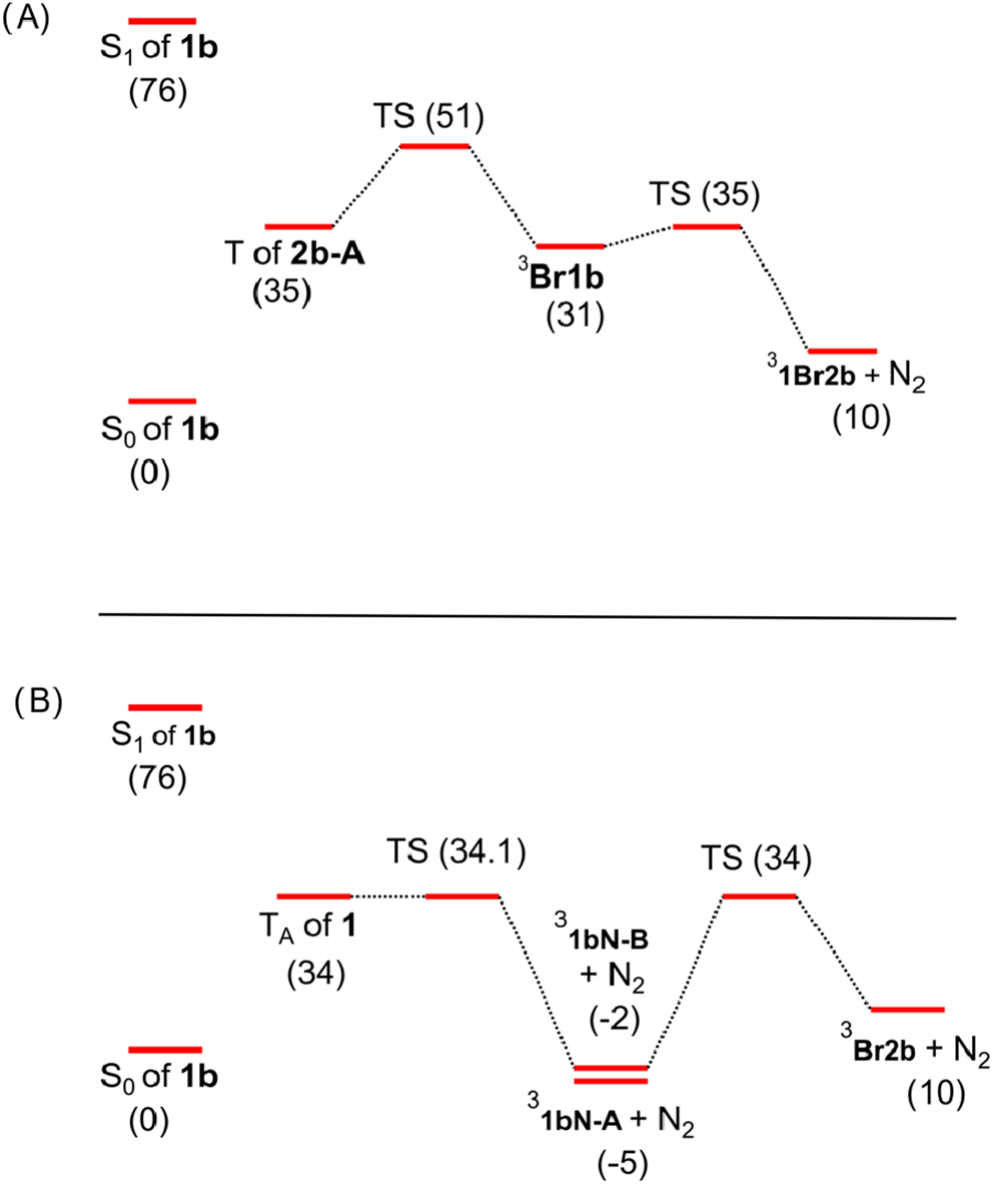
Calculated (B3LYP-D3/6-31+G­(d,p)) stationary points on the triplet surface of **1b** for (A) forming **2b** from T of **1b** and (B) forming **2b** from ^3^ **1bN**. Energies are in kcal mol^‑1^.

**Figure 6:**
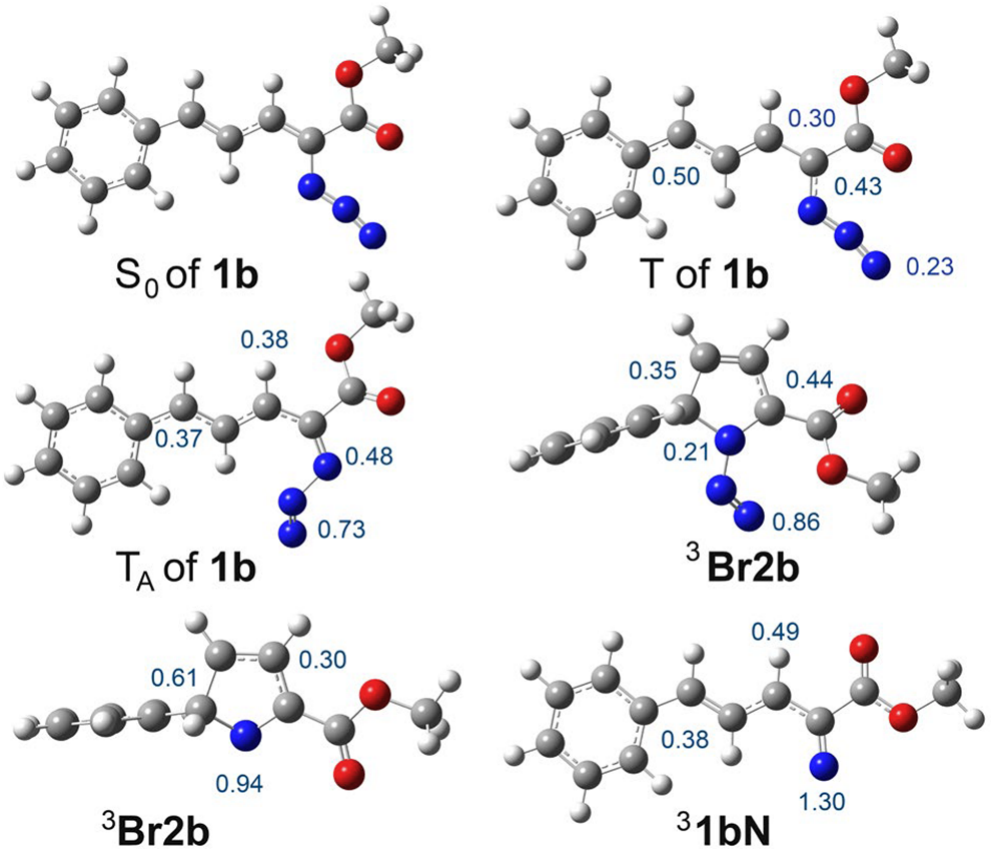
Calculated structures of **1b**, T of **1b**, T_A_ of **1b**, ^3^ **Br1b**, ^3^ **Br2b**, and ^3^ **1bN**. The numbers correspond to the calculated spin densities.

The calculated transition state barrier for the T of **1b** forming ^3^ **Br1b** is located 16 kcal above the T of **1b** (Figure 5A). It is noted that T of **1b** must rotate about its double bonds to adopt a higher energy conformation suitable for forming ^3^ **Br1b**. In comparison, the calculated transition state for ^3^ **Br1b** forming ^3^ **Br2b** is only 4 kcal mol^‑1^. By contrast, the calculated transition state barrier for ^3^ **1bN** formation is only 0.1 kcal mol above the T of **1ba**. However, as ^3^ **1bN** is stable and the calculated transition state for this nitrene reacting to form ^3^ **Br2b** is 36 kcal mol^‑1^ (Figure 5B), this reaction pathway is not viable at ambient temperature. Thus, the calculations further support the mechanism in Scheme 2, as nitrene formation is unlikely to precede product formation. Because T of **1b** has a higher energy than T_A_ of **1b**, T of **1b** reacting to form ^3^ **Br1b** must be more efficient than internal conversion to populate T_A_ of **1b**. Calculation of the triplet surface of **1a** yielded results and conclusions similar to those obtained for **1b** (Figure S58).

### 2.4 Crystal Structures

As described above, we were intrigued by the complete conversion yet distinctly different photodynamic behavior of crystals of **1a** and **1b**. To clarify the origin of this phenomenon, we determined the crystal structures of **1a** and **1b** and attempted to correlate their molecular packing with the observed disintegration patterns. Azide **1a** crystallizes in the orthorhombic space group *Pbca* with one molecule in the asymmetric unit. The molecules stack on top of each other, with alternating aromatic groups and ester moieties forming the corners of a herringbone motif. The intermolecular distance between the molecules is approximately 4 Å, and the angle between the herringbone strands in the crystal, viewed along the *c*-axis, is approximately 60°. Importantly, the azido groups are aligned on the top of one another within the lattice with alternating layers causing these moieties to crisscross. Indexing of the crystals revealed that the (001)/(001̅) face cracks more readily along the short axis of the block crystal, with a lesser extent of cracking occurring along the long axis of the crystal corresponding to face (001)/(001̅) of the *b*-axis (Figures 7A and S51). Azide **1b** crystallizes in the monoclinic *P*2_1_/*c* space group with two molecules in the asymmetric unit. The packing analysis revealed stacked molecules arranged in alternating layers, in which the phenyl groups align with the azido groups, providing a more flexible structure. In this packing arrangement, the azido groups nearly crisscross, with a distance of 5.3 Å between these moieties. Indexing of the main crystal facets revealed that the formation of the fine cracks occurs on its (001̅) surface and along the longer axis of the crystal in the *c*-axis. As the reaction proceeds, additional cracking occurs across the shorter axis of the (011) plane, followed by the formation of additional cracks on the (01̅1) face (Figure 7D). After crystallization from ethyl acetate–hexane, the crystal structures of photoproducts **2a** and **2b** were also determined. Both compounds crystallize in the monoclinic *P*2_1_/*c* space group. Because the shape or molecular volume of **1a** within its crystal lattice cannot accommodate the structure of **2a**, as determined from its crystal structure, we propose that this spatial incompatibility promotes phase separation during the reaction (Figure 7C). It should be noted, however, that **2a** may adopt a crystal packing arrangement that is different when formed *in situ* upon irradiation than when obtained by recrystallization. An analogous situation is observed for **1b** and **2b**, where the mismatch in packing likewise promotes separation into distinct crystalline domains.

**Figure 7:**
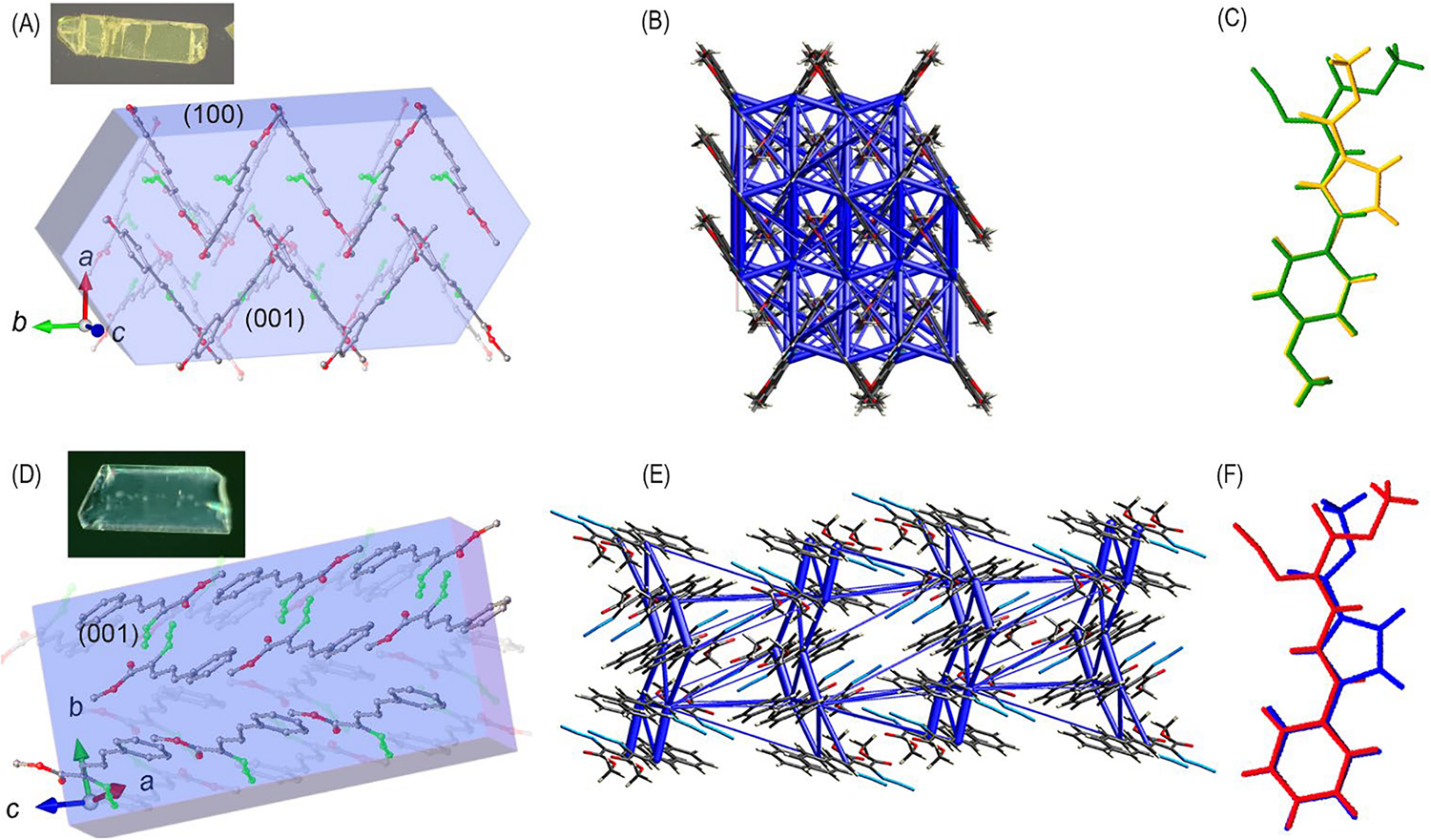
Indexed crystals of (A) **1a** and (D) **1b** after 1 min of irradiation displaying the crystallographically equivalent (001)/(001̅) and (001̅)/(001) faces, respectively. Calculated intermolecular forces for **1a** (B) and **1b** (E) are shown in the same orientation as the indexed crystals to enable a direct comparison. Overlaid representations of the crystal structures of (C) **1a** (green) and **2a** (yellow) and (F) **1b** (red) and **2b** (blue).

To rationalize the reproducible fracture directions observed for crystals of **1a** and **1b**, we performed force-field calculations based on their crystal structures,^59^ which revealed distinct arrangements of strong and weak intermolecular interactions. The crystal structure of **1a** adopts a crossed herringbone motif (Figure 7A) characterized by π-stacking between the phenyl and vinyl moieties with significant crossover between adjacent molecular stacks. This arrangement gives rise to moderately strong intermolecular interactions of 28–29 kJ mol^–1^, which generate a 3D network of interlocked, box-shaped forces throughout the lattice. The weaker interactions (8, 11, 15, and 17 kJ mol^–1^) lie diagonally across these force boxes and are reinforced by the stronger contacts. For example, the 28 and 29 kJ mol^–1^ interactions are paired with an 8 kJ mol^–1^ contact in the (−*x* + 1/2, −*y*, *z* + 1/2) plane, while the 11 and 15 kJ mol^–1^ interactions in the (−*x*, −*y*, −*z*) plane and the 29 kJ mol^–1^ interaction in the (−*x* + 1/2, *y* + 1/2, *z*) plane interlock along the *c*-axis (Figure S41). In contrast, the crystal structure of **1b** retains a more layered arrangement in which the phenyl group of one molecule stacks above the azide group of the next. Force-field calculations reveal that the strongest interaction (24 and 45 kJ mol^–1^) holds the *a* and *c* axes together at a stacking distance of ∼3.5 Å (Figure S44). These layers are further stabilized by intermediate interactions of 18 and 19 kJ mol^–1^, which act diagonally across the layers and are perpendicular to them. Additional interactions of 6, 9, 12, and 18 kJ mol^–1^ link the strongest contacts, creating a three-dimensional box-like framework that is notably more open than that found in **1a**. The box-like framework in **1b** includes a stronger two-dimensional ladder motif than in **1a**, giving rise to its more pronounced layered organization. The weakest interactions (6 kJ mol^–1^), arising from polarized azido−π contacts,^22^ occur diagonally across these force boxes in the c-plane. Upon irradiation, we propose that the weakest forces break first, allowing N_2_ to accumulate between layers, while the strong stacking interactions remain intact. As the pressure increases, the stronger 24 and 45 kJ mol^–1^ forces are eventually overcome, resulting in sudden explosive failure. Because the force network in **1b** is less tightly interwoven than that in **1a**, the lattice can expand during early gas release as seen (Figure 2 and Videos 1, 2, 3, 4, 5, and 6), giving **1b** a brief period of flexibility prior to explosive rupture.

Finally, the molecular shapes and packing arrangements of **2a** and **2b** differ substantially from those of **1a** and **1b** (Figure 7C,F and Table S68). As expected, the loss of the N_2_ molecules results in smaller unit-cell volumes for **2a** and **2b** (10% and 15%) relative to their precursors. Because the photoproducts possess smaller molecular volumes, we propose that this geometric mismatch destabilizes the lattice and triggers phase separation during the transformation. It should be noted that although we obtained an X-ray structure of a dimorph of **1b** (CCDC 2352525), we have not been able to reproducibly generate this polymorph or characterize its photodynamic behavior.

We also checked the possibility of pedal motion, typical for some double bonds, in the crystal structure of **1a** determined at 150, 200, 250, and 295 K to determine whether the conjugated vinylic bonds (C2 and C4 regions) undergo pedal motion within the crystals. As the temperature increased, the anisotropic displacement parameters and residual electron density only exhibited typical increases in thermal motion for all of the atoms. This behavior confirms a lack of pedal-like motions. Thus, the formation of pyrrole **2a** occurs via a flexible triplet excited state rather than through a pedal motion in the solid state.

### 2.5 Phase Change

To establish the changes in the crystal structure, we studied the solid-state reaction of **1a** and **1b** using PXRD.^60^ After the reactant crystals were ground, the diffraction patterns of the reactants before the reaction were recorded. Subsequent patterns were recorded after the powders were irradiated with a 365 nm LED for specific times. For **1a**, the sample did not become amorphous during irradiation. Instead, **2a** formed in a crystal lattice resembling that of the solved crystal structure of the photoproduct, whose structure was determined from crystals grown from ethyl acetate and hexanes. The major peaks of **1a** at 2θ = 10.0° and 25.2° decreased, while the peaks at 2θ = 8.9° and 27.0° corresponding to the photoproduct increased, and full conversion was achieved after 71.5 h (Figure 8A). Azide **1b** exhibited similar behavior, with the sample remaining crystalline; however, peaks corresponding to a new phase appeared at 2θ = 8.7° and 19.6°, and full conversion was achieved after 41.5 h (Figure 8B).

**Figure 8:**
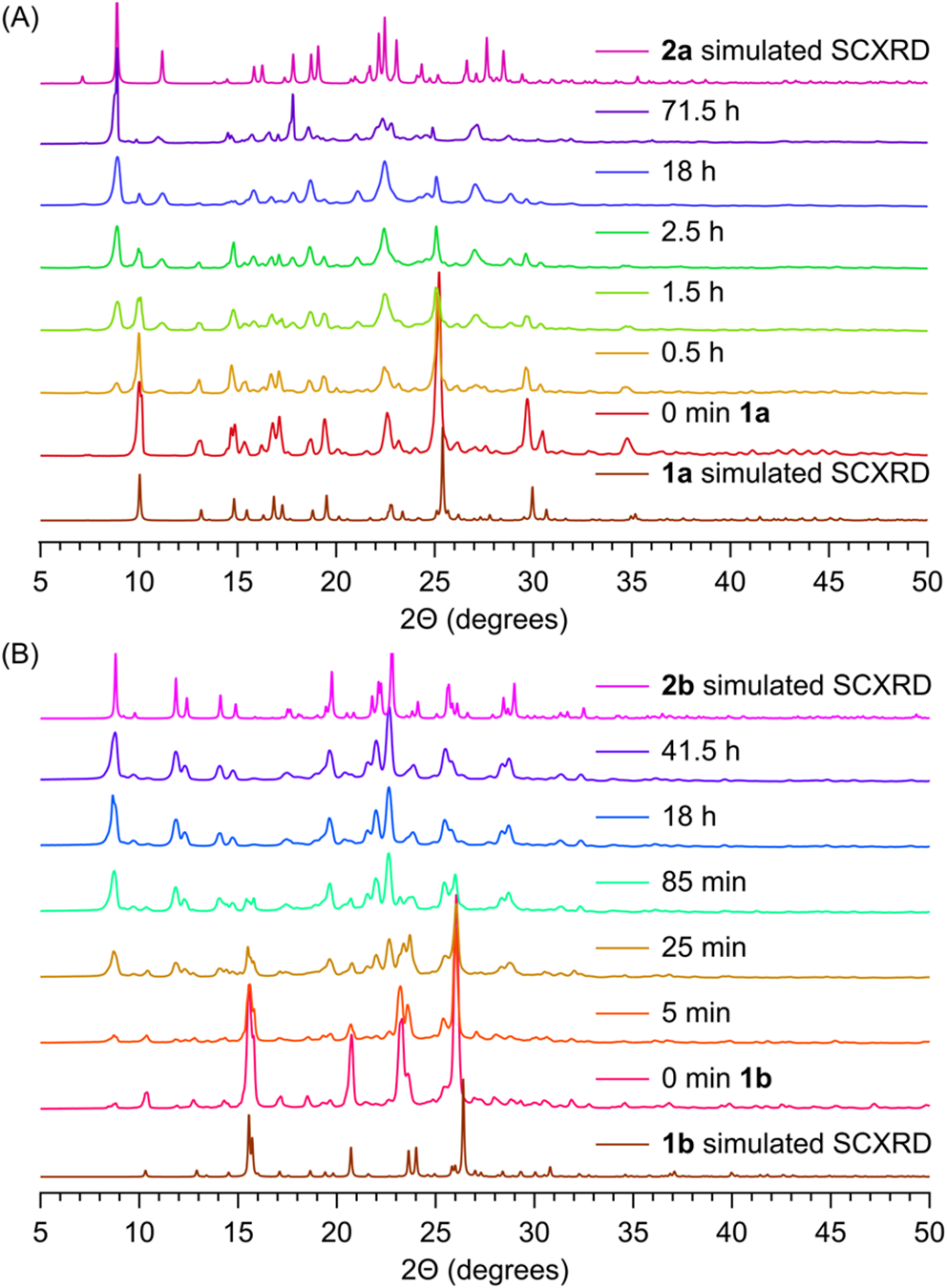
PXRD patterns as a function of the irradiation time for (A) **1a** and (B) **1b**.

After prolonged irradiation of **1a** quantitatively transforms into **2a** as confirmed by PXRD. The PXRD of **2a** is significantly broadened, however, due to the photofracking resulting in residual microstrain and preferred orientation effects during the solid-state transformation.^61–63^ Consistent with this interpretation, SEM reveals well-defined 400–600 nm crystallites of **2a**, while material derived from **1b** is less uniform, highlighting differences in lattice response and fragmentation pathways between the two systems.

These observations indicate that the reaction proceeds through a phase-separation mechanism because the photoproduct cannot be accommodated within the original crystal lattice. The disparity in the molecular shape between the starting material and the photoproduct prevents the lattice from maintaining structural integrity during the reaction. Instead, the cooperative action of phase separation, which accommodates the structural and volumetric mismatch, together with photofracking, which generates new reactive surfaces, enables the reaction to proceed efficiently and thereby enhances the overall reactivity of the crystals.

## 3 Conclusions

We determined the reaction mechanism for azides **1a** and **1b** forming pyrroles **2a** and **2b**, respectively, in the solid state and solution using laser-flash photolysis. The reactions occurred from the triplet excited state that cyclized to form a biradical, which released a N_2_ molecule to yield the photoproduct rather than forming a triplet nitrene intermediate. Importantly, the solid-state reactions proceeded to completion because the crystals continuously cracked and fractured upon light exposure, exposing new crystal surfaces. Furthermore, the absorption properties of the photoproducts did not impede light absorption by the starting materials. Although azides **1a** and **1b** had complex crystal packing arrangements, their specific photodynamic behavior could be explained by correlating the intermolecular forces within the crystal lattice with the crystal indexing. The crystals were found to fracture along planes where weaker intermolecular forces fail to align with stronger intermolecular forces. Finally, phase juxtaposition between the azide and photoproduct lattices contributes to the observed photodynamic motion, demonstrating that photofracking can be exploited to enhance the efficiency of solid-state photochemical reactions.

## 4 Experimental Section

### 4.1 Photofracking Reactions

Photofracking reactions driven by LED lights were carried out on single crystals or crystalline aggregates of **1a** or **1b** under ambient conditions. Crystals were grown from ethyl acetate/hexane mixture and placed on a glass slide or inside a beaker, a transparent Pyrex vial, or a test tube. Samples were irradiated using various LED light sources (365, 450, or 532 nm; measured emission profiles are provided in the SI) at a fixed source–sample distance of 10 cm, with the illuminated area encompassing the entire crystal(s). Reaction progress was monitored by periodically removing small portions of the irradiated solid, dissolving the material in CDCl_3_ (0.5 mL) and recording the ^1^H NMR spectra of the resulting solutions. For the LED experiments, irradiation using a 532 nm LED resulted in significantly slower photoreaction rates compared with irradiation with 365 or 450 nm LEDs, consistent with the weaker absorption of **1a** and **1b** at longer wavelengths.

Sunlight photofracking experiments on crystallines **1a** and **1b** were carried out by placing the solids in a beaker covered with a watch glass or in a transparent Pyrex vial. To minimize thermal effects during irradiation, the beaker or the vial was placed in a dry ice bath or ice bath during summer experiments or in a snowbank during winter experiments. Sunlight exposure was conducted outdoors under clear conditions, and the incident irradiance was measured by using a calibrated power meter.

## Supplementary Material















## Notes

Azide **1a** (crystal A) in air irradiated and monitored using an optical microscope with a video camera; the 10 min recording shortened to 1 min (Video 1) (AVI)
Azide **1a** (crystal B) in air irradiated and monitored using an optical microscope with a video camera; the 8 min recording shortened to 1 min (Video 2) (AVI)
Azide **1a** (crystal C) in mineral oil irradiated and monitored using an optical microscope with a video camera; the 7 min recording shortened to 1 min (Video 3) (AVI)
Azide **1b** (crystal D) in air irradiated and monitored using an optical microscope with a video camera; the 11 min recording shortened to 30 s (Video 4) (AVI)
Azide **1b** (crystal E) in mineral oil monitored using an optical microscope with a video camera; the 5 min recording shortened to 1 min (Video 5) (AVI)
Azide **1b** (crystal F) in minimal MeOH irradiated and monitored using an optical microscope with a video camera; the 12 min recording shortened to 30 s (Video 6) (AVI)
General procedures; synthesis of **1a** and **1b**; product studies; spectra characterizarion for **1** and **2**; transient spectra and kinetics; size determination of nanoparticles; and Cartesian coordinates, energies, and vibrational frequencies of **1**, T of **1**, T_A_ of **1**, ^3^ **Br1**, ^3^ **Br2**, ^3^ **1N**, and **2** (PDF)
