# Supplementary material for: Photofracking-Assisted Enhancement of Solid-State Photochemical Reactivity: α‑Azido-5-phenyl-2,4-dienoate Derivatives
Source: J Am Chem Soc. 2026 Apr 26;148(17):17696–706. doi: 10.1021/jacs.5c22335 (PMC13154190; doi:10.1021/jacs.5c22335)
Supplement: Supplementary file 7 [file ja5c22335_si_007.pdf]

Supporting Information

For

Photofracking-Assisted Enhancement of Solid-State Photochemical Reactivity: α-Azido-5-phenyl-2,4-dienoate Derivatives

Upasana Banerjee,[1] Janaka P. K. Kavikarage,[1] Fiona J. Wasson,[1] W. Dinindu Mendis,[1] Wandana K. S. Henarath Mohottige,[1] Rajkumar Merugu,[1] Anushree Das,[1] Alexis Mack,[1] Nawame Kitil,[1] Savanna Hayes-Bogle,[1] Gabriel D. Patrick,[1] Jack K. Clegg,[2] Durga Prasad Karothu,[3,4] Panče Naumov,[3,4,5,6] Jeanette A. Krause,[1] Anna D. Gudmundsdottir[1]*

[1]*Department of Chemistry, University of Cincinnati, Cincinnati, Ohio 45221, USA*

[2]*School of Chemistry and Molecular Biosciences, The University of Queensland, St Lucia, Qld, Australia, 4072*

[3]*Smart Materials Lab, New York University Abu Dhabi, PO Box 129188, Abu Dhabi, UAE*

[4]*Center for Smart Engineering Materials, New York University Abu Dhabi, PO Box 129188, Abu Dhabi, UAE*

[5]*Research Center for Environment and Materials, Macedonian Academy of Sciences and Arts, Bul. Krste Misirkov 2, MK–1000 Skopje, Macedonia*

[6]*Molecular Design Institute, Department of Chemistry, New York University, 100 Washington Square East, New York, NY 10003, USA*

1. General Experimental Section S10
1.1. Preparation of 1a and 1b S10
1.1.1. Synthesis of (2Z,4E)- 4-methoxyphenyl 2-azidohexa-2,4-dienoate 1a S10
1.1.2. Synthesis of (2Z,4E)-Phenyl 2-azidohexa-2,4-dienoate 1b S10
1.2. Solid-State Photolysis S10
1.3. Preparative Photolysis of Crystalline 1 with 365 nm LED S11
1.4. White Light Irradiation of Crystals Monitored with ^{1}H NMR Spectroscopy as a Function of Irradiation Time. S11
1.4.1. White Light Irradiation of Crystals of Azide 1a S11
1.4.2. White Light Irradiation of Crystals of Azide 1b S11
1.5. Solution Photolysis S12
1.6. Quantum Chemical Calculations S12
1.7. Force Field Calculations S12
1.8. Video Microscopic Analysis of Crystals of 1a and 1b S12
1.9. Preparation of 1b nanocrystalline suspension for PXRD analysis S12
2. Characterizing the starting materials 1a/1b and photoproducts 2a/2b S13
Figure S1. ^{1}H NMR (400 MHz, $CDCl_3$) spectrum of 1a S13
Figure S2. IR (solid) spectrum of azide 1a S14
Figure S3. ^{1}H NMR (400 MHz, $CDCl_3$) spectrum of azide 1b S15
Figure S4. IR (solid) spectrum of 1b S16
Figure S5. ^{1}H NMR (400 MHz, $CDCl_3$) spectrum of photoproduct 2a S17
Figure S6. IR (solid) spectrum of photoproduct 2a S18
Figure S7. ^{1}H NMR (400 MHz, $CDCl_3$) spectrum of photoproduct 2b S19
Figure S8. IR (solid) spectrum of photoproduct 2b. S20
Figure S9. Diffuse reflectance UV spectra of 1a (A) and 1b (B) converted to the Kubelka–Munk function, $f(R)$ ... S21
3. Absorption Spectrum of 1 as a Function of irradiation times S22
Figure S10. Absorption spectra as a function of irradiating 1a in acetonitrile with 365 nm LED [0.003 mg/ mL] .. S22
Figure S11. Absorption spectra as a function of irradiating 1a in acetonitrile with 450 nm LED [0.006 mg/ mL] .. S22
Figure S12. Absorption spectra as a function of irradiating 1a in acetonitrile with 532 nm LED [0.001 mg/ mL] .. S23
Figure S13. Absorption spectra as a function of irradiating 1b in acetonitrile with 365 nm LED [0.001 mg/ mL] .. S23
Figure S14. Absorption spectra as a function of irradiating 1b in acetonitrile with 450 nm LED [0.001 mg/ mL] .. S24
Figure S15. Absorption spectra as a function of irradiating 1b in acetonitrile with 532 nm LED [0.002 mg/ mL] .. S24
Figure S16. Absorption spectra as a function of irradiating (395 nm LED) water nanosuspension of crystals 1a [0.035 mg/ mL] S25

Figure S17. Absorption spectra as a function of irradiating (395 nm LED) water nanosuspension of crystals **1b** [0.052 mg/mL] S25

4. ^{1}H-NMR spectra of Crystalline 1a as a Function of Irradiation Time S26

Figure S19. ^{1}H-NMR (400 MHz, $CDCl_3$) spectra of nanosuspension of crystalline 1a as a function of irradiation time - extension S27

Figure S20. ^{1}H-NMR (400 MHz, $CDCl_3$) spectra of nanosuspension of crystalline 1b as a function of irradiation time S28

Figure S21. ^{1}H-NMR (400 MHz, $CDCl_3$) spectra of nanosuspension of crystalline 1b as a function of irradiation time – extension S29

5. Preparative Product Studies S30

Figure S22. ^{1}H-NMR (400 MHz, $CDCl_3$) spectra after irradiating (365 nm LED, 18h) crystals of 1a (50 mg) S30

Figure S23. ^{1}H-NMR (400 MHz, $CDCl_3$) spectra after irradiating (365 nm LED, 16h) crystals of 1b (50 mg) S31

Figure S24. ^{1}H-NMR (400 MHz, $CDCl_3$) spectra after irradiating (365 nm LED, 12 min) 1a (10.8 mg) in $CDCl_3$ (0.4 mL) S32

Figure S25. ^{1}H-NMR (400 MHz, $CDCl_3$) spectra after irradiating(365 nm LED, 3 min) 1b (1.5 mg) in $CDCl_3$(0.4 mL) S33

6. ^{1}H NMR Spectra of Crystalline 1 after Exposure to Sunlight S34

Figure S26. ^{1}H-NMR (400 MHz, $CDCl_3$) spectra of crystalline 1a after exposure to sunlight for 22 hours S34

Figure S27. ^{1}H-NMR (400 MHz, $CDCl_3$) spectra of crystalline 1b after exposure to sunlight for 22 hours S35

7. Laser Flash Photolysis S36

7.1. Laser Flash Photolysis of 1b in acetonitrile S36

Figure S28. A) Transient Spectra obtained from laser flash photolysis of 1b in argon-saturated acetonitrile, B) extension S36

Figure S29. Kinetic traces from laser flash photolysis of 1b in argon-saturated acetonitrile at A) 400 nm and B) at 525 nm. S37

7.2. Laser Flash Photolysis of 1a in nanosuspension S38

Figure S30. Kinetic traces from laser flash photolysis of 1a in argon-saturated nanosuspension at 550 nm S38

Figure S31. Kinetic traces from laser flash photolysis of 1a in argon-saturated nanosuspension at 400 nm S38

Figure S32. Transient Spectra obtained from laser flash photolysis of 1a in argon-saturated nanosuspension S39

7.3. Laser Flash Photolysis of 1b in nanosuspension S40

Figure S33**.** Transient Spectra obtained by laser flash of 1b in argon-saturated nanosuspension using PMT S40

Figure S34. Kinetic trace at 340 nm obtained by laser flash of 1b in argon-saturated nanosuspension S40

Figure S35. Kinetic trace at 500 nm showing the growth, obtained by laser flash of 1b in argon-saturated nanosuspension S41

Figure S36. Kinetic trace at 500 nm showing the decay, obtained by laser flash of 1b in argon-saturated nanosuspension S41

8. TD-DFT Spectra S42

Figure S37. TD-DFT calculated electronic transition for A) T_A of 1a and B) nitrene ^31aN S42

Figure S38. TD-DFT calculated electronic transition for A) T_1 of 1b, B) ^{3}Br1b and C) ^{3}1Br2b S43

Figure S39. TD-DFT calculated electronic transition for A) T_A of 1a, and B) ^{3}1bN S44

9. X-ray Structures.. S45

9.1. X-ray Structures of Azide 1a.. S45

Figure S40. Crystal structure of azide 1a (CCDC-2352521).. S45

Table S1. Crystal data and structure refinement for $C_{13}H_{13}N_3O_3$ 1a at 150K. (CCDC-2352521)............................ S46

Table S2. Atomic coordinates [x104] and equivalent isotropic displacement parameters [$Å^2x10^3$] for 1a $C_{13}H_{13}N_3O_3$ at 150K. U(eq) is defined as one third of the trace of the orthogonalized Uij tensor...................................... S47

Table S3. Bond lengths [Å] and angles [°] for 1a $C_{13}H_{13}N_3O_3$ at 150K. S48

Table S4. Torsion angles [°] for 1a $C_{13}H_{13}N_3O_3$ at 150 K. S48

Table S5. Anisotropic displacement parameters [$Å^2x10^3$] for 1a $C_{13}H_{13}N_3O_3$ at 150K. The anisotropic displacement factor exponent takes the form: -2π2[h 2a*2U11+... +2hka*b*U12] S49

Figure S41. Crystal explorer energy lattice calculations of 1a (CCDC-2352521) in kJ/mol in B3LYP theory S51

Figure S42. Crystal Explorer lattice interaction energies for 1a (CCDC-2352521) in kJ/mol with B3LYP theory S52

9.2. X-ray Structure of Azide 1b S52

Figure S43. Crystal structure of 1b (CCDC-2352526) Polymorph I S52

Table S6. Crystal data and structure refinement for 1b $C_{13}H_{11}N_3O_2$ (CCDC-2352526)... S53

Table S7. Atomic coordinates [$x10^4$] and equivalent isotropic displacement parameters [$Å^2x10^3$] for **1b** $C_{13}H_{11}N_3O_2$ (CCDC-2352526). U(eq) is defined as one third of the trace of the orthogonalized U_{ij} tensor.......................... S54

Table S8. Bond lengths [Å] and angles [°] for 1b $C_{13}H_{11}N_3O_2$ (CCDC-2352526)... S56

Table S9. Anisotropic displacement parameters [$Å^2x10^3$] for 1b $C_{13}H_{11}N_3O_2$ (CCDC-2352526). The anisotropic displacement factor exponent takes the form: $-2\pi^2[h\ ^2a^{*2}U_{11}+...+2hka^*b^*U_{12}]$. S57

Table S10. Torsion angles [°] for 1b $C_{13}H_{11}N_3O_2$ (CCDC-2352526)... S58

Figure S44. Crystal explorer energy lattice calculations of 1b $C_{13}H_{11}N_3O_2$ (CCDC-2352526) in kJ/mol in B3LYP theory…….. S59

Figure S45. Crystal Explorer lattice interaction energies for 1b (CCDC- 2352526) in kJ/mol with B3LYP theory S60

Figure S46. Crystal structure of 1b (CCDC-2352525) Polymorph II S61

Table S11. Crystal data and structure refinement for 1b $C_{12}H_{11}N_3O_2$ (CCDC-2352525)....................................... S62

Table S12. Atomic coordinates [$x10^4$] and equivalent isotropic displacement parameters [$Å^2x10^3$] for 1b $C_{12}H_{11}N_3O_2$. U(eq) is (CCDC-2352525)defined as one third of the trace of the orthogonalized Uij tensor. S63

Table S13. Bond lengths [Å] and angles [°] for 1b $C_{12}H_{11}N_3O_2$ (CCDC-2352525).. S64

Table S14. Anisotropic displacement parameters [$Å^2x10^3$] for 1b $C_{12}H_{11}N_3O_2$ (CCDC-2352525). The anisotropic S64

Table S15. Torsion angles [°] for 1b $C_{12}H_{11}N_3O_2$ (CCDC-2352525). S65

Figure S47. Crystal explorer energy lattice calculations of 1b (CCDC- 2352525) in kJ/mol in B3LYP theory S66

Figure S48. Crystal Explorer lattice interaction energies for 1b (CCDC- 2352525) in kJ/mol with B3LYP theory S67

Figure S49. Crystal structure of 1b (Sjkc23f18) Polymorph 1 .... S68

Table S16. Crystal data and structure refinement for 1b (Sjkc23f18) S69

Table S17. Fractional Atomic Coordinates (×10^4) and Equivalent Isotropic Displacement Parameters (Å^2×10^3) for 1b (Sjkc23f18). Ueq is defined as 1/3 of the trace of the orthogonalised UIJ tensor. S70

Table S18. Anisotropic Displacement Parameters (Å^2×10^3) for 1b (Sjkc23f18). The Anisotropic displacement factor exponent takes the form: -2π2[h2a*2U11+2hka*b*U12+…]. S71

Table S19. Bond Lengths for 1b (Sjkc23f18). S72

Table S20. Bond Angles for 1b (Sjkc23f18) S72

Table S21. Torsion Angles for 1b (Sjkc23f18) S73

Table S22. Hydrogen Atom Coordinates (Å×10^4) and Isotropic Displacement Parameters (Å^2×10^3) for 1b (Sjkc23f18). S74

Figure S50. Refinement of 1b (cu-meoioazidedp_0min_DK) S74

9.3. Crystal Face Indexing of Azides 1a and 1b S75

Figure S51. Crystal face indexing for azide 1a (CCDC-2352521) S75

Figure S52. Crystal face indexing for azide 1a (cu-meoioazidedp_0min_DK) S75

Figure S53. Crystal face indexing for azide 1b (18075c) (CCDC-2352525) S76

Figure S54. Crystal face indexing for azide 1b (Sjkc23f20) S76

9.4. Effect of Temperature on the Crystal Structure of Azide 1a. S77

Figure S55. Crystals Structure of 1a obtained at different temperature to rule out pedal motion experiment on 1a. S77

Table S23. Crystal data and structure refinement for azide 1a ($C_{13}H_{13}N_3O_3$) at 150K. (CCDC 2352521) S78

Table S24. Atomic coordinates [x10^4] and equivalent isotropic displacement parameters [Å^{2}x10^3] for azide 1a ($C_{13}H_{13}N_3O_3$) at 150K (CCDC-2352521). U(eq) is defined as one third of the trace of the orthogonalized Uij tensor. S79

Table S25. Bond lengths [Å] and angles [°] for azide 1a ($C_{13}H_{13}N_3O_3$) at 150K (CCDC-2352521). S80

Table S26. Torsion angles [°] for azide 1a ($C_{13}H_{13}N_3O_3$) at 150K (CCDC-2352521). S80

Table S27. Anisotropic displacement parameters [Å^{2}x10^3] for azide 1a ($C_{13}H_{13}N_3O_3$) at 150K (CCDC-2352521). The anisotropic displacement factor exponent takes the form: $-2\pi^2[h^2a^{*2}U_{11}+ ... +2hka^*b^*U_{12}]$ S81

Table S28. Crystal data and structure refinement for azide 1a ($C_{13}H_{13}N_3O_3$) at 200K (CCDC-2352522) S82

Table S29. Atomic coordinates [x10^4] and equivalent isotropic displacement parameters [Å^{2}x10^3] for azide 1a ($C_{13}H_{13}N_3O_3$) at 200K CCDC-2352522. U(eq) is defined as one third of the trace of the orthogonalized Uij tensor. S83

Table S30. Bond lengths [Å] and angles [°] for azide 1a ($C_{13}H_{13}N_3O_3$) at 200K (CCDC-2352522). S84

Table S31. Torsion angles [°] for azide 1a ($C_{13}H_{13}N_3O_3$) at 200K (CCDC-2352522). S84

Table S32. Anisotropic displacement parameters [Å^{2}x10^3] for azide 1a ($C_{13}H_{13}N_3O_3$) at 200K (CCDC-2352522). The anisotropic displacement factor exponent takes the form: -2π2[h 2a*2U11+... +2hka*b*U12] S85

Table S33. Crystal data and structure refinement for azide 1a ($C_{13}H_{13}N_3O_3$) at 250K (CCDC-2352523). S85

Table S34. Atomic coordinates [x10^4] and equivalent isotropic displacement parameters [Å^{2}x10^3] for azide 1a ($C_{13}H_{13}N_3O_3$) at 250K (CCDC-2352523). U(eq) is defined as one third of the trace of the orthogonalized U_{ij} tensor. S86

Table S35. Bond lengths [Å] and angles [°] for azide 1a ($C_{13}H_{13}N_3O_3$) at 250K (CCDC-2352523). S87

Table S36. Torsion angles [°] for azide 1a ($C_{13}H_{13}N_3O_3$) at 250K (CCDC-2352523). S87

Table S37. Anisotropic displacement parameters [Å^{2}x10^3] for azide 1a ($C_{13}H_{13}N_3O_3$) at 250K (CCDC-2352523). The anisotropic displacement factor exponent takes the form: $-2\pi^2[h^2a^{*2}U_{11}+... +2hka^*b^*U_{12}]$ S88

Table S38. Crystal data and structure refinement for azide 1a ($C_{13}H_{13}N_3O_3$) at 295K (CCDC-2352524). S88

Table S39. Atomic coordinates [x10^4] and equivalent isotropic displacement parameters [Å^{2}x10^3] for azide 1a ($C_{13}H_{13}N_3O_3$) at 295K (CCDC-2352524). U(eq) is defined as one third of the trace of the orthogonalized Uij tensor. S89

Table S40. Bond lengths [Å] and angles [°] for azide 1a ($C_{13}H_{13}N_3O_3$) at 295K (CCDC-2352524). S90

Table S41. Torsion angles [°] for azide 1a ($C_{13}H_{13}N_3O_3$) at 295K (CCDC-2352524). S90

Table S42. Anisotropic displacement parameters [Å^{2}x10^3] for azide 1a ($C_{13}H_{13}N_3O_3$) at 295K (CCDC-2352524).. The anisotropic displacement factor exponent takes the form: -2π2[h 2a*2U11+... +2hka*b*U12] S91

9.5. X-ray Structure of Photoproduct 2a S91

Figure S56. Crystal Structure of Photoproduct 2a (CCDC-2352527) S91

Table S43. Crystal data and structure refinement for 2a $C_{13}H_{13}NO_3$ (CCDC-2352527). S92

Table S44. Atomic coordinates [x10^4] and equivalent isotropic displacement parameters [Å^{2}x10^3] for 2a $C_{13}H_{13}NO_3$ (CCDC-2352527). U(eq) is defined as one third of the trace of the orthogonalized Uij tensor. S93

Table S45. Bond lengths [Å] and angles [°] for 2a $C_{13}H_{13}NO_3$ (CCDC-2352527). S94

Table S46. Anisotropic displacement parameters [Å^{2}x10^3] for 2a $C_{13}H_{13}NO_3$ (CCDC-2352527). The anisotropic displacement factor exponent takes the form: $-2\pi^2[h^2a^{*2}U_{11}+... +2hka^*b^*U_{12}]$ S95

Table S47. Torsion angles [°] for 2a $C_{13}H_{13}NO_3$ (CCDC-2352527). S96

9.6. X-ray Structure of Photoproduct 2b S97

Figure S57. Crystal structure of photoproduct 2b (CCDC-2352528) S97

Table S48. Crystal data and structure refinement for 2b $C_{12}H_{11}NO_2$ (CCDC-2352528) S98

Table S49. Atomic coordinates [x10^4] and equivalent isotropic displacement parameters [Å^{2}x10^3] for 2b $C_{12}H_{11}NO_2$ (CCDC-2352528). U(eq) is defined as one third of the trace of the orthogonalized Uij tensor. S99

Table S50. Bond lengths [Å] and angles [°] for 2b $C_{12}H_{11}NO_2$ (CCDC-2352528). S100

Table S51. Anisotropic displacement parameters [Å^{2}x10^3] for 2b $C_{12}H_{11}NO_2$ (CCDC-2352528). The anisotropic displacement factor exponent takes the form: $-2\pi^2[h^2a^{*2}U_{11}+... +2hka^*b^*U_{12}]$ S101

Table S52. Torsion angles [°] for 2b $C_{12}H_{11}NO_2$ (CCDC-2352528). S102

Figure S58. Crystal structure of 2b (Sjkc23fw20) S102

Table S53. Crystal data and structure refinement for 2b S103

Table S54. Fractional Atomic Coordinates (×10^4) and Equivalent Isotropic Displacement Parameters (Å^2×10^3) for 2b (Sjkc23fw20). Ueq is defined as 1/3 of the trace of the orthogonalised UIJ tensor. S104

Table S55. Anisotropic Displacement Parameters ($Å^2×10^3$) for 2b (Sjkc23fw20). The Anisotropic displacement factor exponent takes the form: $-2\pi^2[h^2a^{*2}U_{11}+2hka^*b^*U_{12}+…]$. S105

Table S56. Bond Lengths for 2b (Sjkc23fw20)........ S106

Table S57.Bond Angles for 2b (Sjkc23fw20). S107

Table S58. Torsion Angles for 2b (Sjkc23fw20). S108

Table S59. Hydrogen Atom Coordinates ($Å×10^4$) and Isotropic Displacement Parameters ($Å^2×10^3$) for 2b (Sjkc23fw20). S109

10. Calculations........ S110

10.1. Calculations for 1b S110

10.1.1. Optimization of Azide 1b-A........ S110

10.1.2. Optimization of Azide 1b-B........ S111

10.1.3. Optimization of Azide 1b-C........ S112

10.1.4. Optimization of Azide 1b-D S113

10.1.5. Optimization of T of 1b-A S114

10.1.6. Optimization of T of 1b-B S115

10.1.7. Optimization of T of 1b-C S116

10.1.8. Optimization of T of 1b-D S117

10.1.9. Optimization of ^{3}Br1b-A S118

10.1.10. Optimization of ^{3}Br1b-B S118

10.1.11. Optimization of ^{3}Br2b-B S120

10.1.12. Optimization of ^{3}Br2b-B S121

10.1.13. Optimization of 2b-A S122

10.1.14. Optimization of 2b-B S123

10.1.15. Optimization of T_A of 1 .... S124

10.1.16. Optimization of Nitrene ^{3}1Nb-A........ S125

10.1.17. Optimization of ^{3}1Nb-B S126

10.1.18. Transition state barrier for T of 1b forming ^{3}Br1b S127

10.1.19. Transition state barrier for ^{3}Br1b forming ^{3}1Br2b S128

10.1.20. Transition state barrier for ^{3}1Nb forming ^{3}Br2b S129

10.1.21. Transition state barrier for T of 1b-D forming ^{3}1Nb S130

10.2. Calculations for 1a S131

10.2.1. Optimization of 1a-A........ S131

10.2.2. Optimization of 1a-B........ S132

10.2.3. Optimization of 1a-C........ S133

10.2.4. Optimization of 1a-D S134

10.2.5. Optimization of T of 1a-A S135

10.2.6. Optimization of T of 1a-B........ S136

10.2.7. Optimization of T of 1a-C........ S137

10.2.8. Optimization of T of 1a-D S138

10.2.9. Optimization of ^{3}Br1a-A........ S139

10.2.10. Optimization of ^{3}Br1a-B S140

10.2.11. Optimization of ^{3}Br2a-A........ S141

10.2.12. Optimization of ^{3}Br2a-B S142

10.2.13. Optimization of Nitrene ^{3}1Na-A........ S143

10.2.14. Optimization of T_A of 1a........ S144

10.2.15. Optimization of 2a-A........ S145

10.2.16. Optimization of 2a-B........ S146

10.3. Spin Density Calculations........ S147

10.3.1. Spin Density T of 1a-A........ S147

10.3.2. Spin Density T_A of 1a........ S148

10.3.2. Spin Density of ^{3}Br1a S149

10.3.2. Spin Density of ^{3}Br2a S150

10.3.3. Spin Density ^{3}1Na-A........ S151

10.3.4. Spin Density ^{3}1Na-B S152

10.3.5. Spin Density of T of 1b-A S153

10.3.5. Spin Density of T_A of 1b S154

10.3.6. Spin Density of ^{3}Br2b-A S155

10.3.7. Spin Density of ^{3}Br1b-A S156

10.3.7. Spin Density of ^{3}Br1b-B........ S157

10.3.7. Spin Density of ^{3}1Nb-A S158

10.4. Calculated Stationary Points on the Energy Diagram for 1........ S159

11. Emission Spectra of Light Sources S160

11.1. Emission spectrum of Microscope White Light........ S160

Figure S60. Keyence microscope white light emission for irradiation at 100% intensity with λmax at 450 and 550 nm. S160

11.2. Emission spectra of LEDs S161

Figure S61. Emission spectrum of LEXEON 395 nm LED........ S161

Figure S62. Emission spectrum of LEXEON Z 450 nm LED........ S162

Figure S63. Emission spectrum of LEXEON Z 532 nm LED........ S163

Figure S64. Emission spectrum of 365 nm LED S164

11.3. 254 nm UV Pen Emission Spectrum S165

Figure S65. Emission spectrum of 254 nm Analytik Jena (UVP) Pen-Ray 90001201 .... S165

12. Dynamic Light Scattering Data (DLS) S166

12.1. DLS analysis of azide 1a nanocrystalline suspension S166

Table S60. Summary of DLS analyses of azide 1a nanocrystalline suspension in water S166

Table S61. Percentiles of DLS analyses of azide 1a nanocrystalline suspension in water S166

Table S62. Raw data of DLS analyses of azide 1a nanocrystalline suspension in water S167

12.2. DLS analysis of azide 1b nanocrystalline suspension S168

Table S63. Summary of DLS analyses of azide 1b nanocrystalline suspension in water S168

Table S64. Percentiles of DLS analyses of azide 1b nanocrystalline suspension in water S168

Table S65. Raw data of DLS analyses of azide 1b nanocrystalline suspension in water S168

12.3. DLS analysis of azide 1b nanocrystalline suspension for PXRD analysis S170

Table S66. Summary of DLS analyses of azide 1b nanocrystalline suspension in water S170

Table S67. Raw data of DLS analyses of azide 1b nanocrystalline suspension in water S171

Figure S66. PXRD pattern of nanocrystalline 1b obtained from the water suspension S172

Table S68. Volume for unit cell of 1a, 1b, 2a and 2b from their crystal structures. S172

13. Video Descriptions S173

14. SEM and NMR analyses after Sequential Photoirradiation and PXRD Measurments S164

Figure S67. SEM images of the particles after sequential irradiations and PXRD analysis: (A, B) 2a; (C–E) 2b. S174

Figure S68. ^{1}H-NMR (400 MHz, $CDCl_3$) spectra after irradiating powdered 1a crystals (365 nm LED, 71.5 h) and subsequent PXRD measurements S175

Figure S69. ^{1}H-NMR (400 MHz, $CDCl_3$) spectra after irradiating powdered 1b crystals (365 nm LED, 41.5 h) and subsequent PXRD measurements S176

15. CCDC Structure Deposition S177

16. **PXRD Instrument S175**

17. References S189

1. General Experimental Section

1.1. Preparation of 1a and 1b

Azides **1a** and **1b** were synthesized in a single step from the condensation of methyl azidoacetate and corresponding aromatic cinnamaldehydes as described in the literature with minor modifications.[1]

1.1.1. Synthesis of (2Z,4E)- 4-methoxyphenyl 2-azidohexa-2,4-dienoate 1a

To a cooled solution (-22 ℃, obtained using dry ice and ortho-xylene mixture) of 25% sodium methoxide in methanol (7.5 mL, 32.81 mmol, 5 equiv), argon gas was purged for 5 minutes to ensure inert atmosphere. A mixture of 4-methoxy-*trans*-cinnamaldehyde (1.0349 g, 6.38 mmol, 1 equiv) and methyl azidoacetate (3 mL, 30.81 mmol, 4 equiv) was diluted in dry methanol and added dropwise into that cooled solution of 25% sodium methoxide in methanol over 45 minutes. After the addition, the resulting reaction mixture was warmed to -10 ℃ and stirred for 4 hours. Next, the mixture was diluted with 25 mL water. The resulting aqueous phase was extracted three times with diethyl ether (325 mL), and the combined organic phases washed with brine (25 mL), dried over anhydrous magnesium sulfate, the solvent removed under reduced pressure, and resulting oil was purified using flash column chromatography eluted with (0-25% ethyl acetate-hexane mixture) to yield pure azide **1a** (1.1078 g, 4.27 mmol, 67.05% yield). Azide **1a** was characterized with ^{1}H-NMR and IR spectroscopy which matched with previously reported data.[1b] mp: 60-67°C. ^{1}H NMR ($CDCl_3$, 400 MHz): ∂ 7.44-7.42 (d, *J* = 8 Hz, 2H), 7.04-7.00 (dd, J_1 = 4 Hz, J_2 = 8 Hz, 1H), 6.89-6.87 (d, *J* = 8 Hz, 2H), 6.79-6.74 (m, 2H), 3.87 (s, 3H), 3.83 (s, 3H) ppm; IR (neat): 2118, 1711, 1509, 1250, 1175, 970 cm^{-1}.

1.1.2. Synthesis of (2Z,4E)-Phenyl 2-azidohexa-2,4-dienoate 1b

The same procedure was followed to form **1b** as for **1a**. To a solution of 25% sodium methoxide in methanol (8 mL, 35 mmol) at -10°C, was added a mixture of cinnamaldehyde (1 mL, 7.94 mmol) and methyl azidoacetate (4 mL, 41.08 mmol) in dry methanol (12 mL) to yield oil that was purified to yield pale yellow solid of **1b** (1.0602 g, 4.62 mmol, 58.3% yield). The spectral data matched with previously reported data.[1b] mp: 62-70°C (lit.[2]; 63-65°C). ^{1}H NMR ($CDCl_3$, 400 MHz): 7.50-7.48 (d, J = 4 Hz, 2H), 7.37-7.28 (m, 3H), 7.20-7.14 (dd, J_1 = 4 Hz, J_2 = 12 Hz, 1H), 6.83-6.74 (m, 2H), 3.85 (s, 3H) ppm; IR (neat): 2124, 1714, 1371, 1234, 1074, 970 and 751 cm^{-1}.

1.2. Solid-State Photolysis

LED Irradiation of Solid Samples Crushed Between Two Microscopic Slides. Crystals of **1a** or **1b** (20 mg) were crushed between two 20 × 20 Pyrex slides to form powder. These two slides were taped on the edges and put in a Ziplock, which was purged with argon for 5 min, the bag was sealed under positive pressure. The bag was irradiated with 365 nm, 450 nm, or 532 nm LEDs (Section 11) in separate experiments. In addition, crystals were placed on top of the 20 × 20 Pyrex slide and were irradiated directly with 254 nm UV-Pen. The photolysis led to full depletion of the starting material and formation of corresponding pyrroles **2a** or **2b** as the only product as revealed with IR and ^{1}H-NMR spectroscopy.

Sunlight Irradiation of Solid Samples.

For the sunlight experiments, azides **1a** (20 mg, 0.077 mmol) and **1b** (20 mg, 0.087 mmol) were placed separately in Pyrex vials (27.5 × 57.5 mm) on the same days. The vials were exposed to sunlight on 1/28/2026, 1/29/2026, and 1/30/2026, with an average irradiance of 22000 – 28000 lux. The average temperatures on these days were −10 °C, −6 °C, and −7 °C, respectively. After 22 hours of irradiation, products **2a** (17.6 mg, 0.076 mmol, 99%) and 2b (17.2 mg, 0.085 mmol, 98%) were obtained from azides **1a** and **1b**, respectively.

LUX measurements were completed with a Light Meter Digital Illuminance Meter (MT-912) from URCERI.

LED Irradiation of Crystalline Nanosuspension Monitored with Absorption Spectroscopy. The photolysis of nanocrystalline suspensions of **1a** and **1b** was monitored by absorption spectroscopy. Nanocrystalline suspensions were prepared using the reprecipitation method, following the experimental procedure reported by Garcia-

Garibay and co-workers.[3] Either azide **1a** or **1b** was dissolved in 0.2 mL of acetone and added to vortexing water to generate the nanocrystalline suspensions. These nanocrystals adhered to the Pyrex glass walls within a few minutes of formation. To overcome this issue, the Pyrex surfaces were treated with Sigmacote (Sigma-Aldrich), a commercially available silanizing agent that renders the surface hydrophobic, thereby allowing the nanocrystals to remain dispersed in the aqueous suspension, as described by Hernández-Linares et al.[4]

The nanocrystalline suspensions were irradiated using a 395 nm LED (Section 11). The progress of the photoreaction was monitored at regular intervals using a JASCO V-750 UV–Vis spectrophotometer.

1.3. Preparative Photolysis of Crystalline 1 with 365 nm LED

Azide **1a** (50 mg, 0.19 mmol) was placed in a Pyrex vial and irradiated with a 365 nm LED light for 18 hours. After irradiation, both IR and ^{1}H NMR confirmed complete conversion of **1a** to **2a** (42 mg, 0.18 mmol, 95% yields). ^{1}H NMR (400 MHz, $CDCl_3$) δ 7.60 – 7.53 (m, 2H), 7.41 (dd, *J* = 8.5, 6.9 Hz, 2H), 7.34 – 7.28 (m, 1H), 6.96 (dd, *J* = 3.9, 2.4 Hz, 1H), 6.55 (dd, *J* = 3.9, 2.7 Hz, 1H), 3.88 (s, 3H) ppm.

Azide **1b** (50 mg, 0.218 mmol) was placed in a Pyrex vial and irradiated with a 365 nm LED light for 16 hours. After irradiation, both IR and ^{1}H NMR confirmed complete conversion of 1b to 2b (43.4 mg, 0.216 mmol, 99% yield). ^{1}H NMR (400 MHz, $CDCl_3$) δ 9.38 (s, 1H), 7.61 – 7.53 (m, 2H), 7.41 (t, *J* = 7.6 Hz, 2H), 7.34 – 7.28 (m, 1H), 6.96 (dd, *J* = 3.9, 2.5 Hz, 1H), 6.55 (t, *J* = 3.3 Hz, 1H), 3.88 (s, 3H).

1.4. White Light Irradiation of Crystals Monitored with ^{1}H NMR Spectroscopy as a Function of Irradiation Time

1.4.1. White Light Irradiation of Crystals of Azide 1a

Azide **1a** (70 mg, 0.27 mmol) was placed in a Pyrex vial and irradiated with white light from a microscope (Section 11). The progress of the photoreaction was monitored by periodically removing small aliquots of the sample and recording the ^{1}H NMR spectra of the reaction mixture (Figure S16 and Figure S17).
The mass of the irradiated compound used for each measurement and the corresponding irradiation times were as follows: (2 min, 2.7 mg), (4 min, 1.9 mg), (7 min, 1.0 mg), (15 min, 2.3 mg), (30 min, 2.1 mg), (45 min, 1.5 mg), (60 min, 1.3 mg), (75 min, 1.5 mg), (90 min, 2.2 mg), (105 min, 1.9 mg), (120 min, 1.0 mg), (240 min, 2.7 mg), (360 min, 2.4 mg), and (1380 min, 3.0 mg).
The mass of the fully converted compound remaining in the irradiated vial was 38.9 mg (0.17 mmol), and a total of 27.5 mg was used to follow the conversion of **1a** into **2a** over the course of the experiment.
2a; mp: 144-147 °C, (lit.;[1b] 144-146°C); ^{1}H NMR ($CDCl_3$, 400 MHz): δ 9.32 (br s, 1H), 7.50 (d, *J* = 8.5 Hz, 2H), 6.97-6.93 (m, 3H), 6.44-6.43 (m, 1H), 3.87 (s, 3H), 3.84 (s, 3H) ppm; IR (neat): 3323, 1686, 1477, 1275, 1006, 762 cm^{-1}.

1.4.2. White Light Irradiation of Crystals of Azide 1b

Azide **1b** (65 mg, 0.28 mmol) was placed in a Pyrex vial and irradiated with white light from a microscope. Subsequently, the reaction progress was monitored using ^{1}H NMR spectroscopy as a function of irradiation time The progress of the photoreaction was monitored by periodically removing small aliquots of the sample and recording the ^{1}H NMR spectra of the reaction mixture (Figure S18 and Figure S19).
The mass of the irradiated compound used for each measurement and the corresponding irradiation times were as follows: (2 min, 1.0 mg), (7 min, 2.2 mg), (15 min, 1.1 mg), (30 min, 2.0 mg), (120 min, 1.1 mg), (240 min, 1.1 mg), (360 min, 7.1 mg), and (1200 min, 5.8 mg). The mass of the fully converted compound remaining in the irradiated vial was found to be 39.4 mg, 0.20 mmol) whereas 21.4 mg were used to follow the conversion of **1b** to **2b**.

2b; mp: 142-146 ℃ (Lit.:[3] 142-144°C); ^{1}H NMR ($CDCl_3$, 400 MHz): δ 9.74 (br s, 1H), 7.61-7.60 (m, 2H), 7.41-7.38 (m, 2H), 7.31-7.25 (m, 1H), 6.97-6.96 (m, 1H), 6.55-6.53 (m, 1H), 3.88 (s, 3H) ppm; IR (neat): 3307, 1680, 1275, 1004 cm^{-1}.

1.5. Solution Photolysis

Azide **1a** (10.8 mg, 0.042 mmol) was dissolved $CDCl_4$ (0.4 mL) and placed in a Pyrex NMR tube, argon bubbled through the solution for 12 min, and the tube capped with rubber cap and parafilmed and irradiated with 365 LED light for 2.5 hours and ^{1}H NMR spectra of the irradiated crystals confirmed full conversion to **2a** (9.6 mg, 0.041 mmol, 98% yields).

Azide **1b** (1.5 mg, 0.0065 mmol) was dissolved $CDCl_4$ (0.4 mL) and placed in a Pyrex NMR tube argon bubbled through the solution for 3 min, and the tube capped with rubber cap and parafilmed and irradiated with 365 LED light for 3 min and ^{1}H NMR spectra of the irradiated crystals confirmed full conversion to **2b** (1.3 mg, 0.0064 mmol, 98% yields).

1.6. Quantum Chemical Calculations

All the geometries were optimized at the B3LYP-D3 level of theory with the 6-31G+(d,p) basis set as implemented in the Gaussian16 programs at the Ohio Supercomputer Center.[4] The absorption spectra were calculated using time-dependent density functional theory (TD-DFT).[5] Calculated IR spectra of the intermediates and products were obtained by frequency calculations at the B3LYP-D3 level of theory, using the 6-31G+(d,p) basis set. The transition states were confirmed to have one imaginary vibrational frequency by analytical determination of the second derivative of the energy with respect to the internal coordinates. Intrinsic reaction coordinate (IRC) calculations were used to verify that the transition state correlates with the products and the precursors.[6]

1.7. Force Field Calculations

Crystal Explorer 17.5 was used to generate energy framework, Hirschfeld surface and fingerprint plots for **1a** and **1b** with B3LYP/6-31G(d,p) level energy theory using experimental crystal structures (CIF files) as input files.[7] For energy framework calculation, interaction energies were calculated within 3.8 Å radius of a central molecule. For data presentation, width of the tube sizes was set as 60 and any interactions at 5 kJ/mol and above were shown.

1.8. Video Microscopic Analysis of Crystals of 1a and 1b

Divinylazide crystals **1** were grown by slow evaporation technique. 100 mg of sample was dissolved in 16 mL of (1:1) ethyl acetate and hexane mixture in a 20 mL glass vial. Vial was capped with a piece of aluminum foil. Foil was punctured with holes using a thin needle and the set up was refrigerated. After the desired crystals were formed, a Keyence VHX-1000 digital microscope was used to record dynamic responses of these crystals. The videos were recorded upon irradiation with LEDs for an hour. Obtained videos were sped up using Movie Maker software for convenience. Directional responses of these crystals were studied with a fiber optic cable attached to the LEDs. Nitrogen release was monitored by placing the crystals in a drop of mineral oil while irradiation.

1.9. Preparation of 1b Nanocrystalline Suspension for PXRD Analysis

A small aliquot of a 0.7 mg mL^{-1} solution of **1b** in acetone was added rapidly to water containing micellar concentrations of CTAB (0.1 mM) under sonication. The resulting nanocrystals exhibited an average size of 285 nm, as determined by dynamic light scattering (DLS) analysis. The nanosuspension was filtered through Whatman filter paper and dried overnight in a vacuum oven.

1.10. Diffuse Reflectance UV Measurements

Solid-state UV–vis spectra were collected on a Cary Agilent Series UV–Vis–NIR spectrophotometer equipped with a diffuse reflectance accessory. Finely ground crystalline samples of **1a** and **1b** were each diluted with spectroscopic-grade $BaSO_4$ and analyzed under ambient conditions. A $BaSO_4$ standard was used as the background/reference for all measurements.

2. Characterizing the starting materials 1a/1b and photoproducts 2a/2b

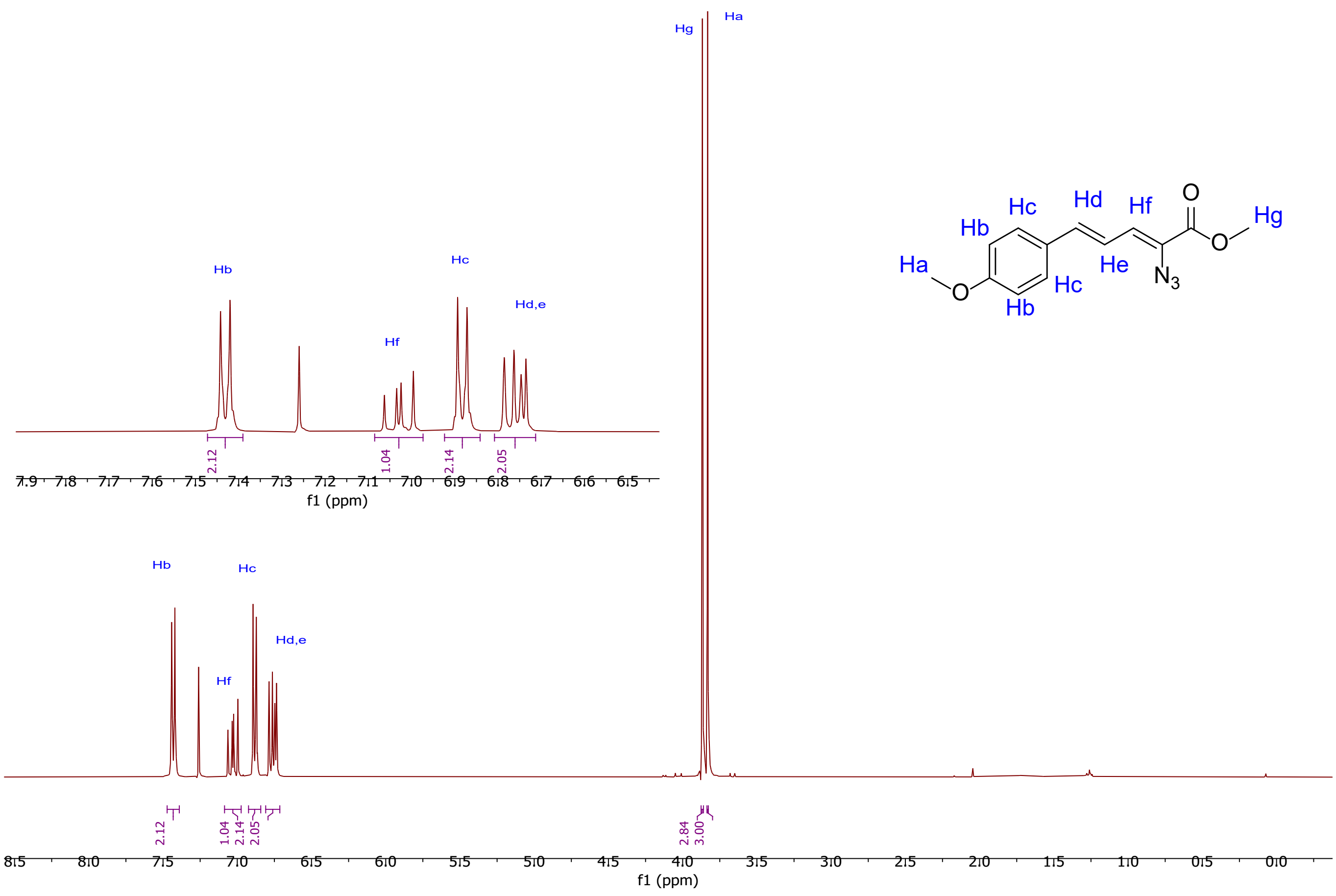

Figure S1. ^{1}H NMR (400 MHz, $CDCl_3$) spectrum of **1a**

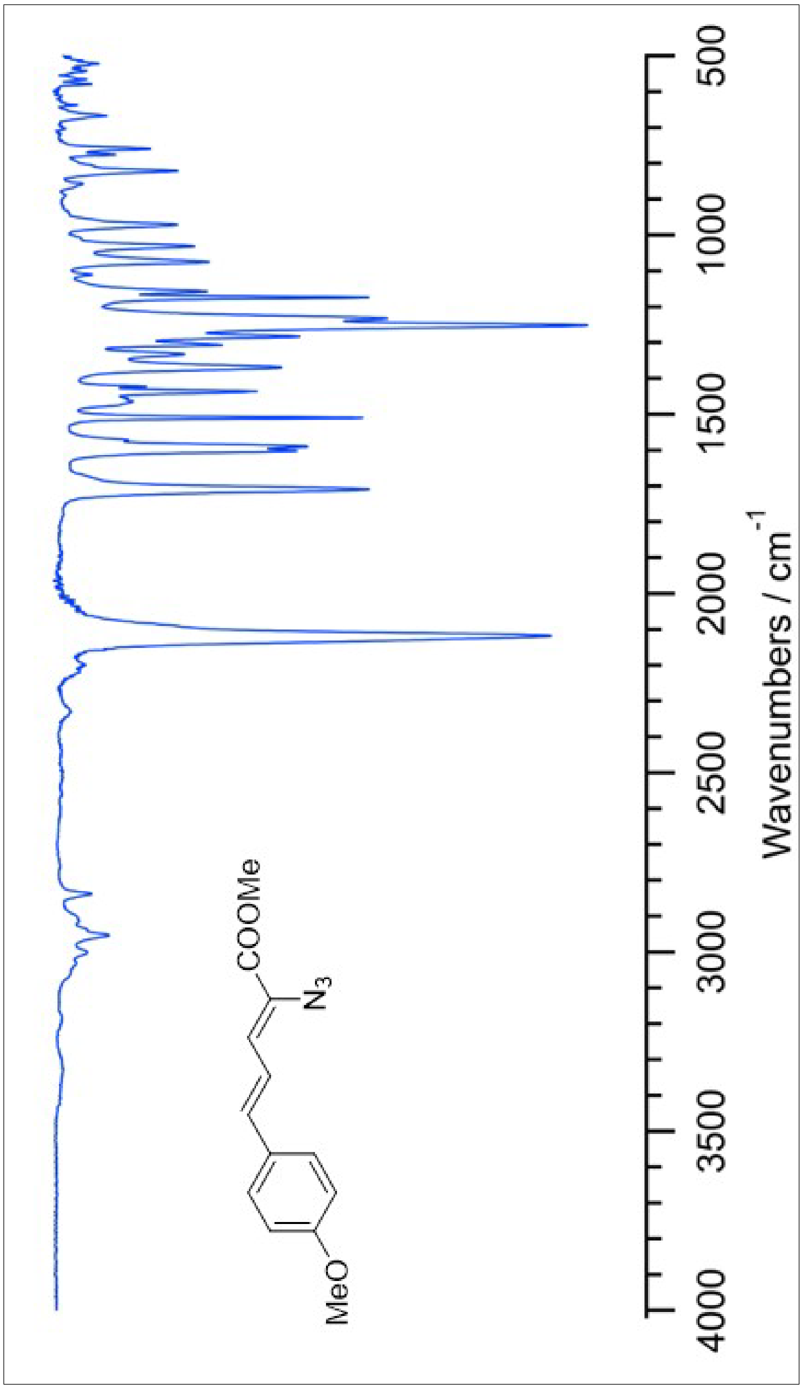

Figure S2. IR (solid) spectrum of azide **1a**

O

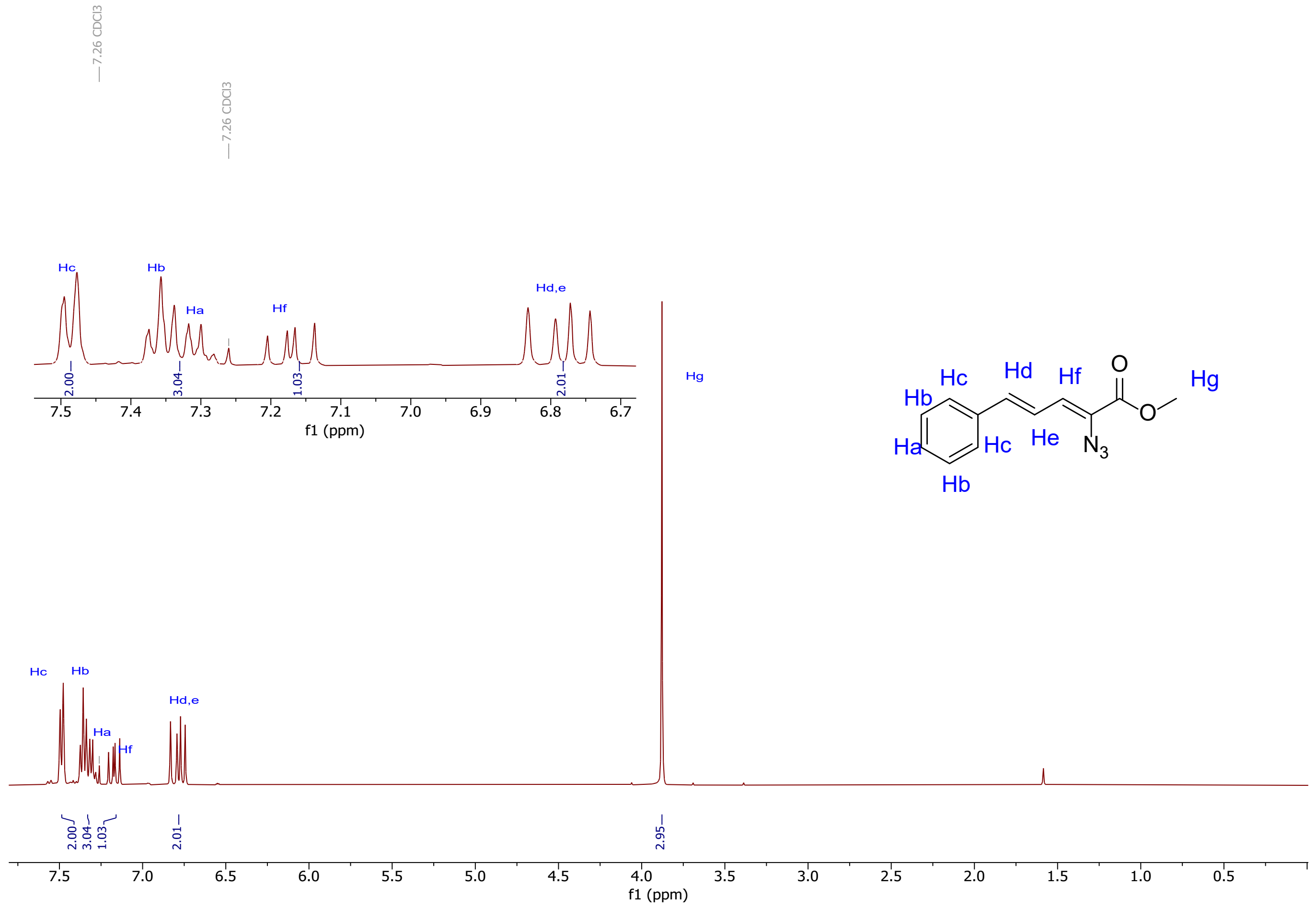

Figure S3. ^{1}H NMR (400 MHz, $CDCl_3$) spectrum of azide **1b**

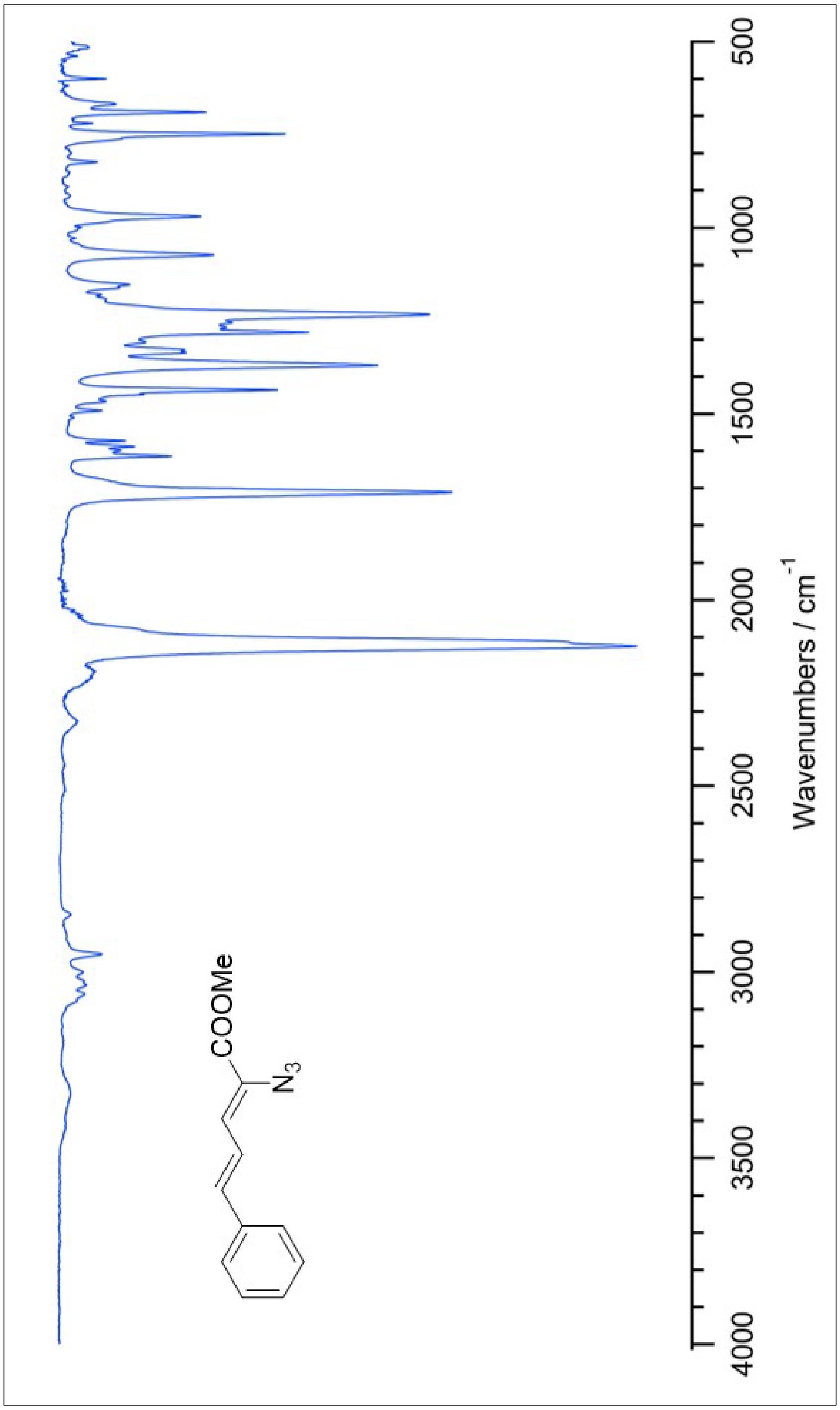

Figure S4. IR (solid) spectrum of **1b**

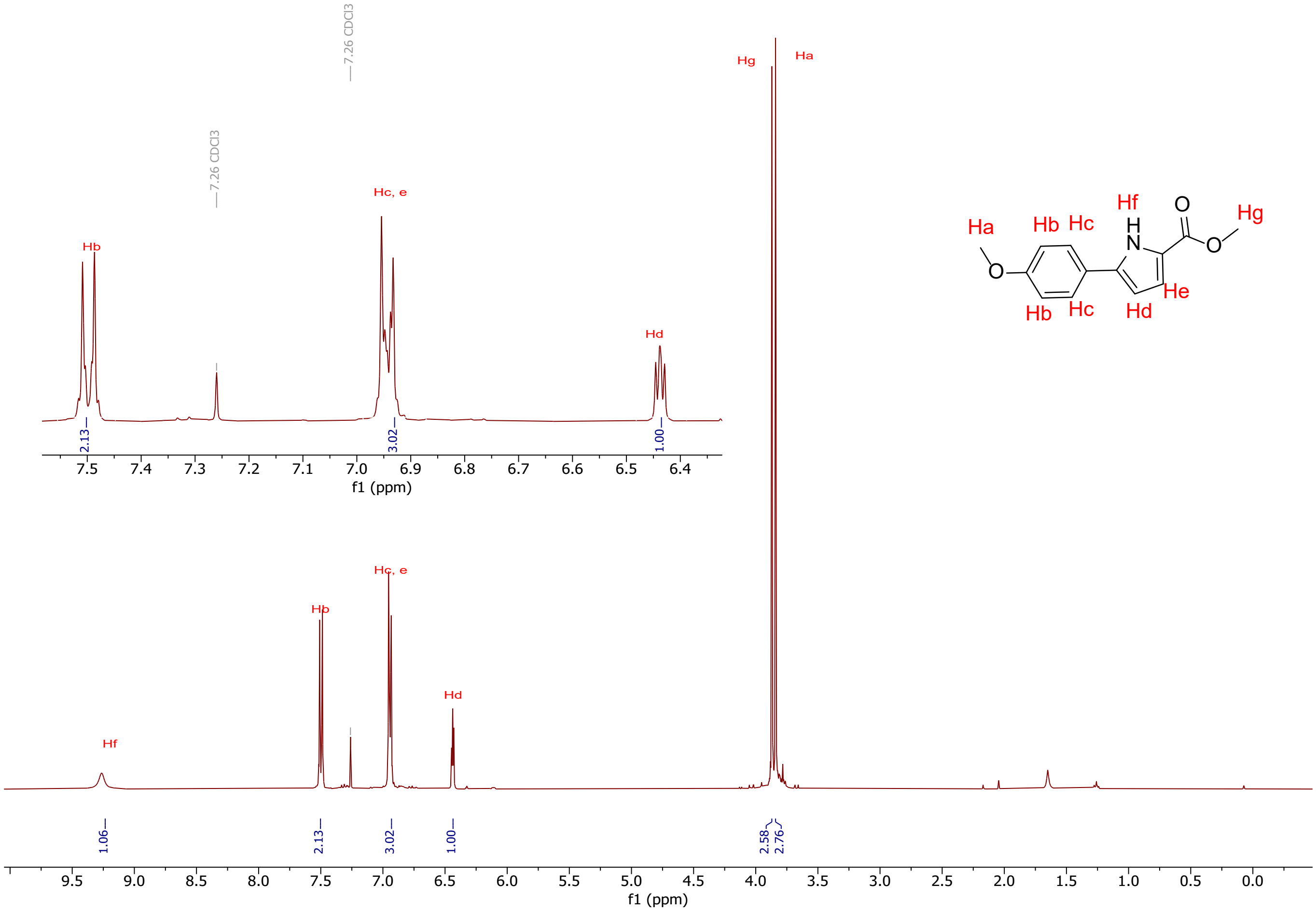

Figure S5. ^{1}H NMR (400 MHz, $CDCl_3$) spectrum of photoproduct **2a**

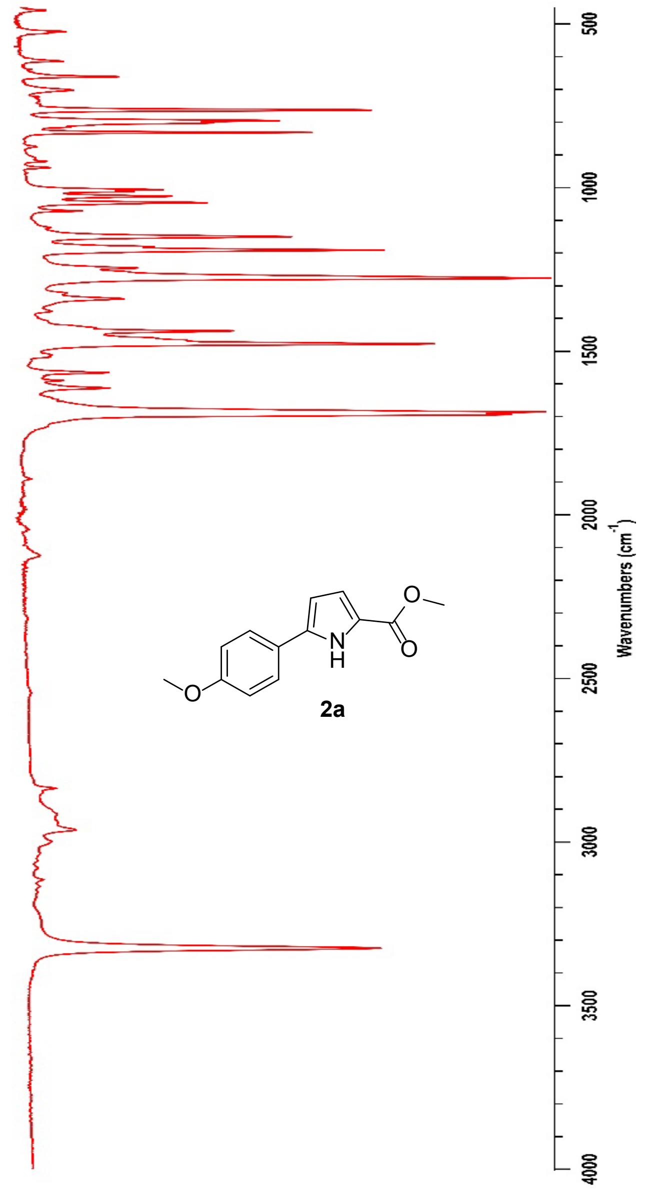

Figure S6. IR (solid) spectrum of photoproduct **2a**

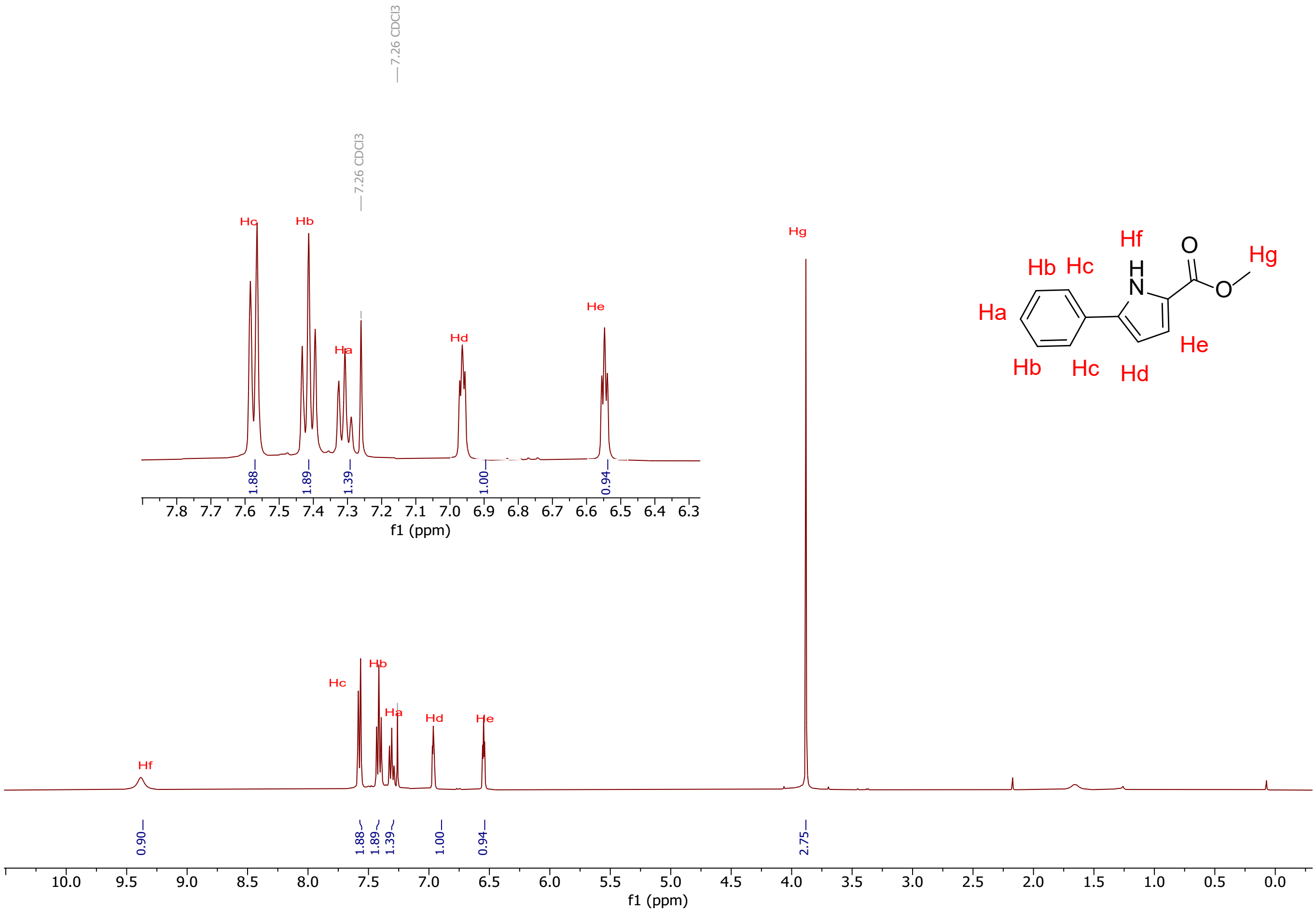

Figure S7. ^{1}H NMR (400 MHz, $CDCl_3$) spectrum of photoproduct **2b**

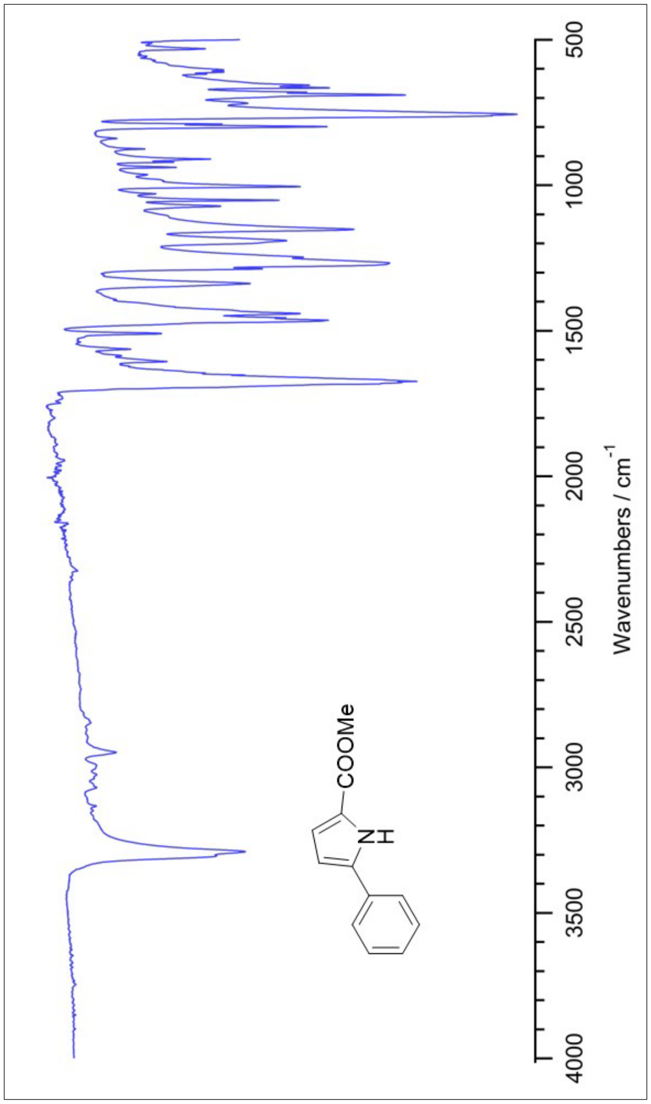

Figure S8. IR (solid) spectrum of photoproduct **2b**.

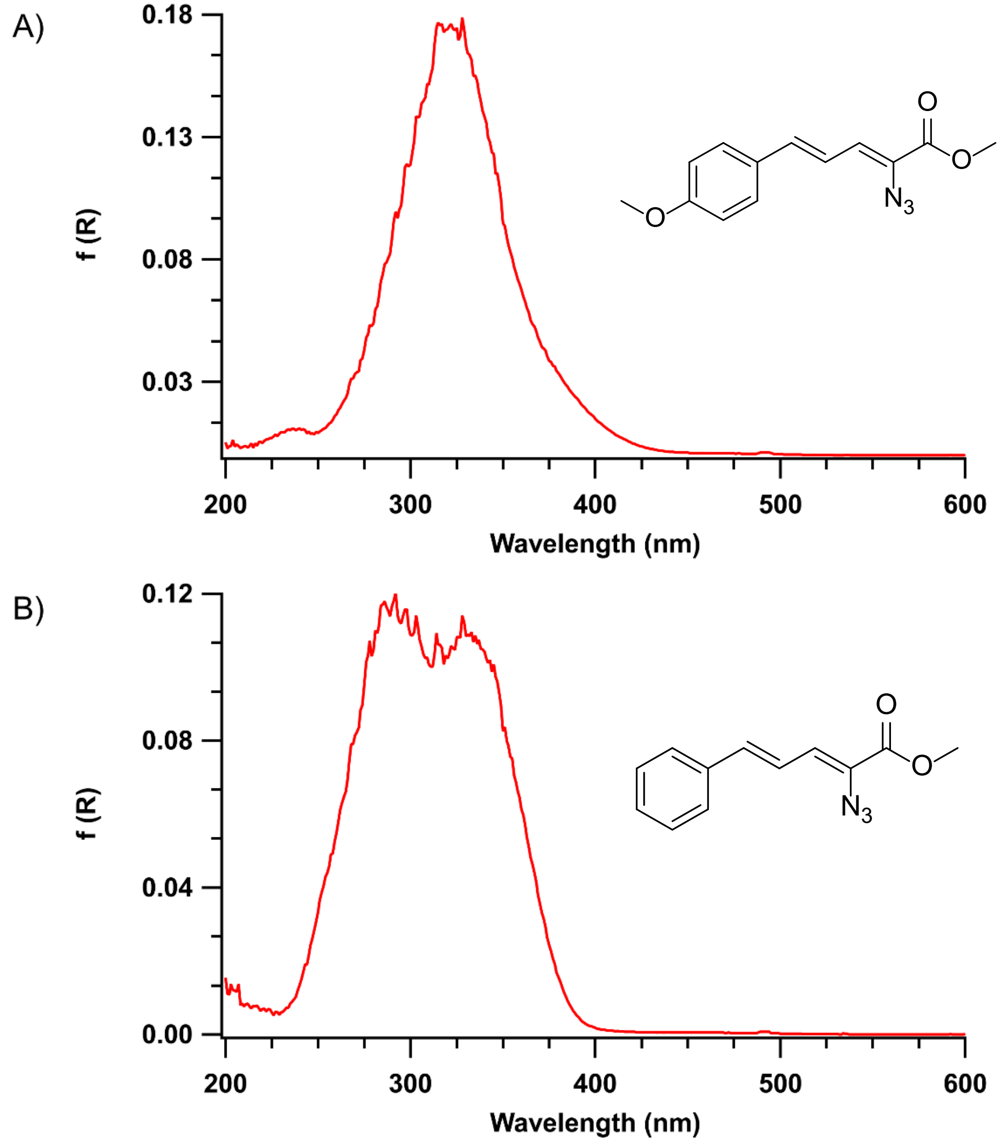

Figure S9. Diffuse reflectance UV spectra of **1a** (A) and **1b** (B) converted to the Kubelka–Munk function, f(R)

Both **1a** and **1b** display a detectable long-wavelength tail extending into the visible. At 532 nm, small but nonzero Kubelka–Munk values are observed (**1a**: 1.58×10^{-5}, **1b**: 2.35×10^{-5}), consistent with low but finite absorption at this wavelength.

3. Absorption Spectrum of 1 as a Function of irradiation times

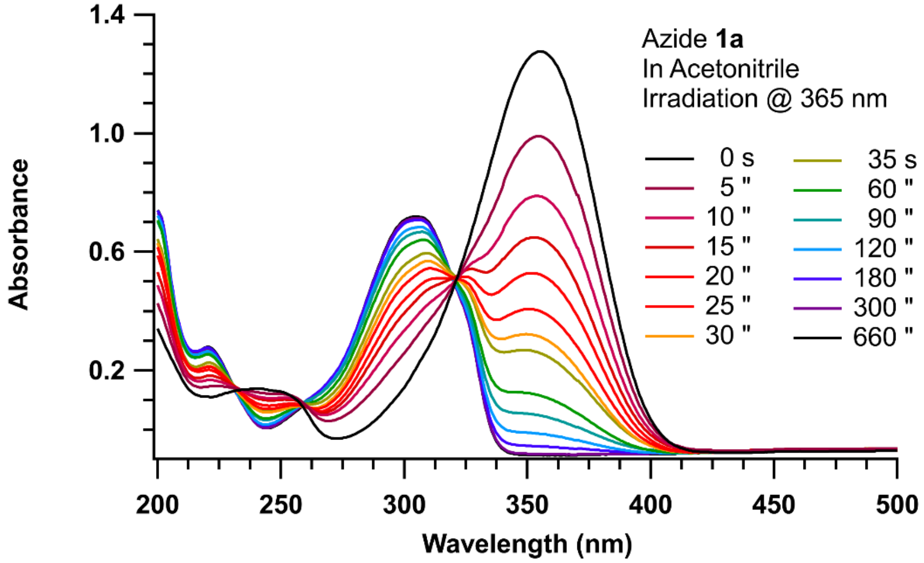

Figure S10. Absorption spectra as a function of irradiating **1a** in acetonitrile with 365 nm LED [0.003 mg/ mL]

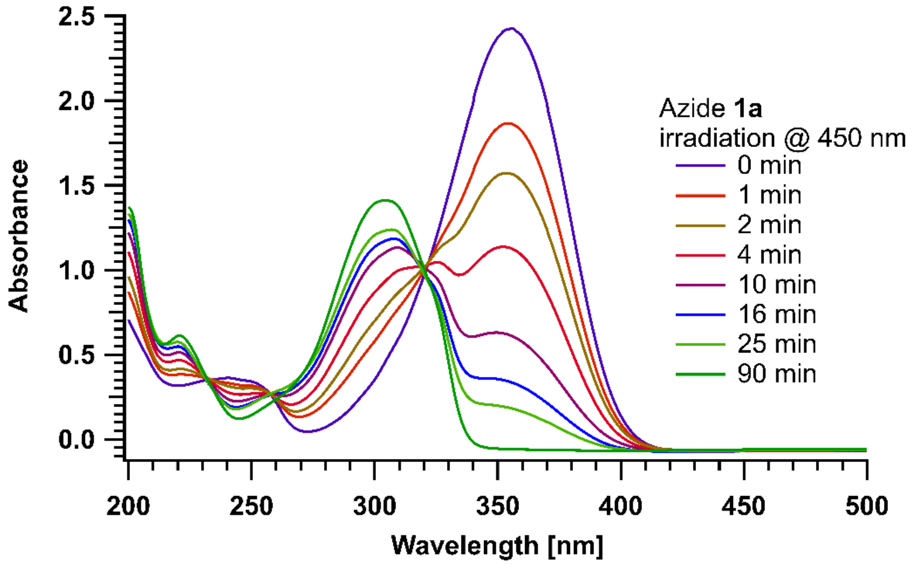

Figure S11. Absorption spectra as a function of irradiating **1a** in acetonitrile with 450 nm LED [0.006 mg/ mL]

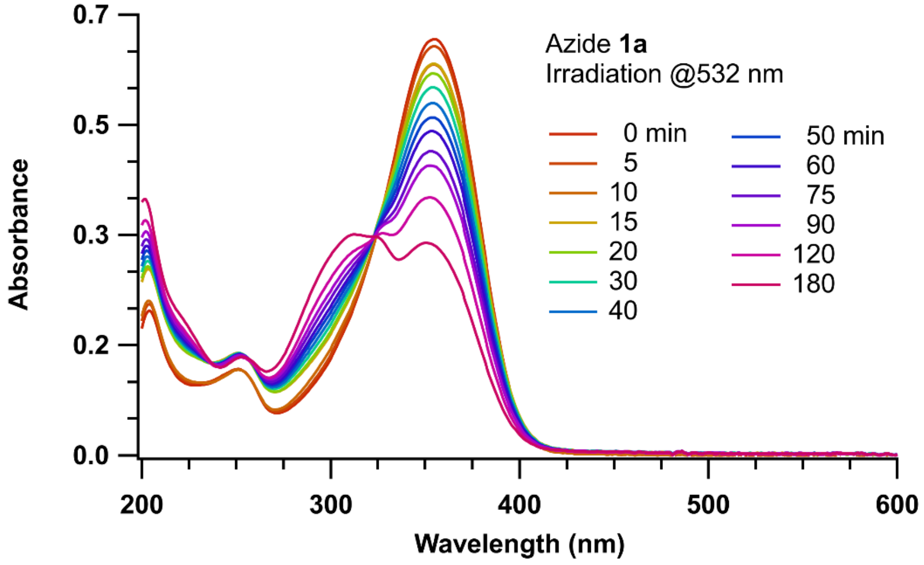

Figure S12. Absorption spectra as a function of irradiating **1a** in acetonitrile with 532 nm LED [0.001 mg/ mL]

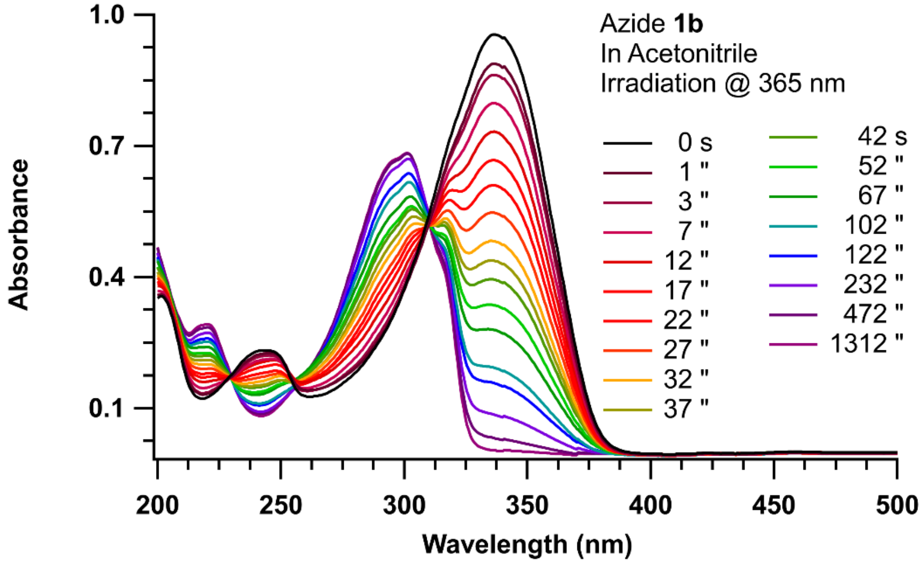

Figure S13. Absorption spectra as a function of irradiating **1b** in acetonitrile with 365 nm LED [0.001 mg/ mL]

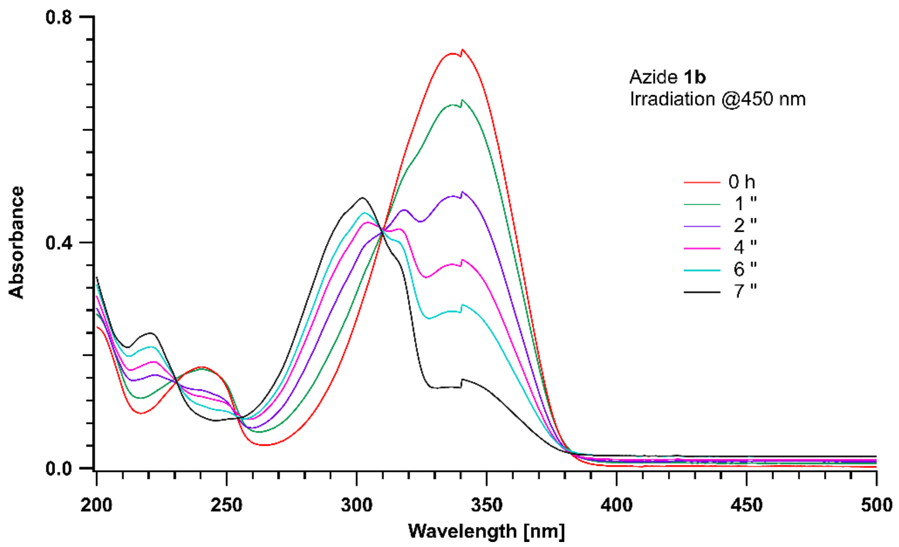

Figure S14. Absorption spectra as a function of irradiating **1b** in acetonitrile with 450 nm LED [0.001 mg/ mL]

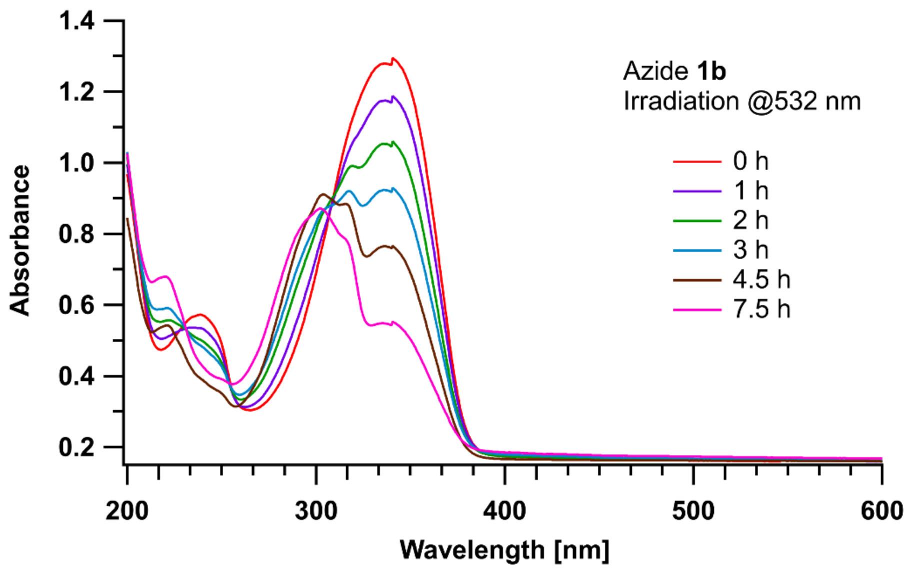

Figure S15. Absorption spectra as a function of irradiating **1b** in acetonitrile with 532 nm LED [0.002 mg/ mL]

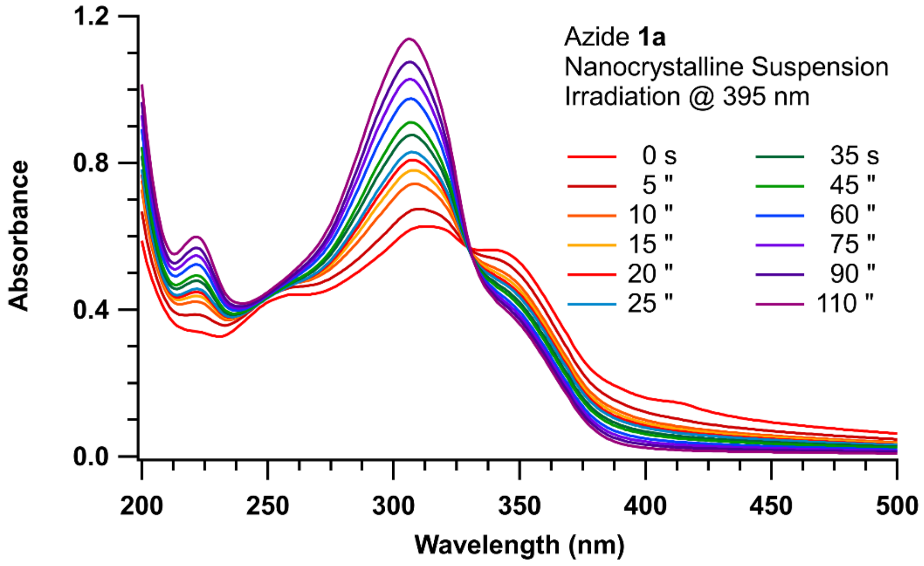

Figure S16. Absorption spectra as a function of irradiating (395 nm LED) water nanosuspension of crystals **1a** [0.035 mg/ mL]

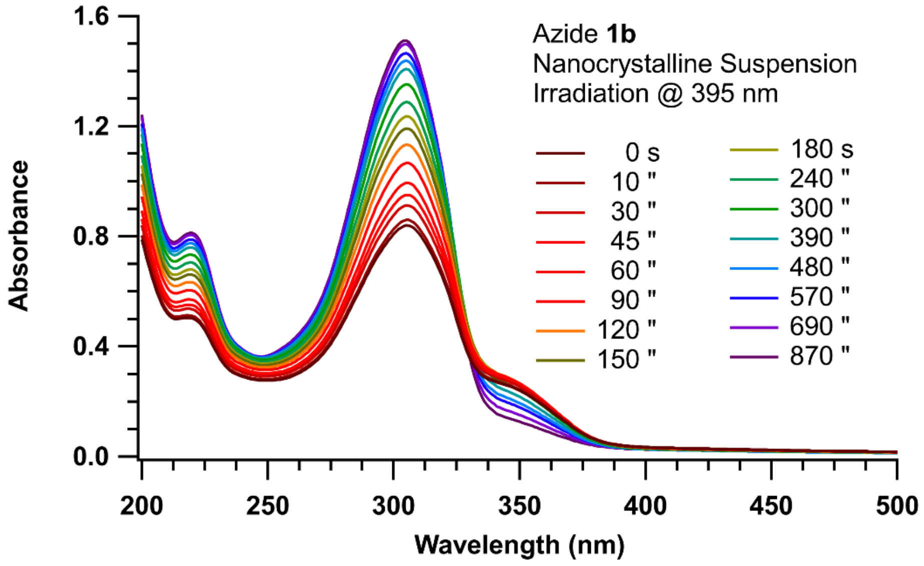

Figure S17. Absorption spectra as a function of irradiating (395 nm LED) water nanosuspension of crystals **1b** [0.052 mg/ mL]

4. ^{1}H-NMR spectra of Crystalline 1a as a Function of Irradiation Time

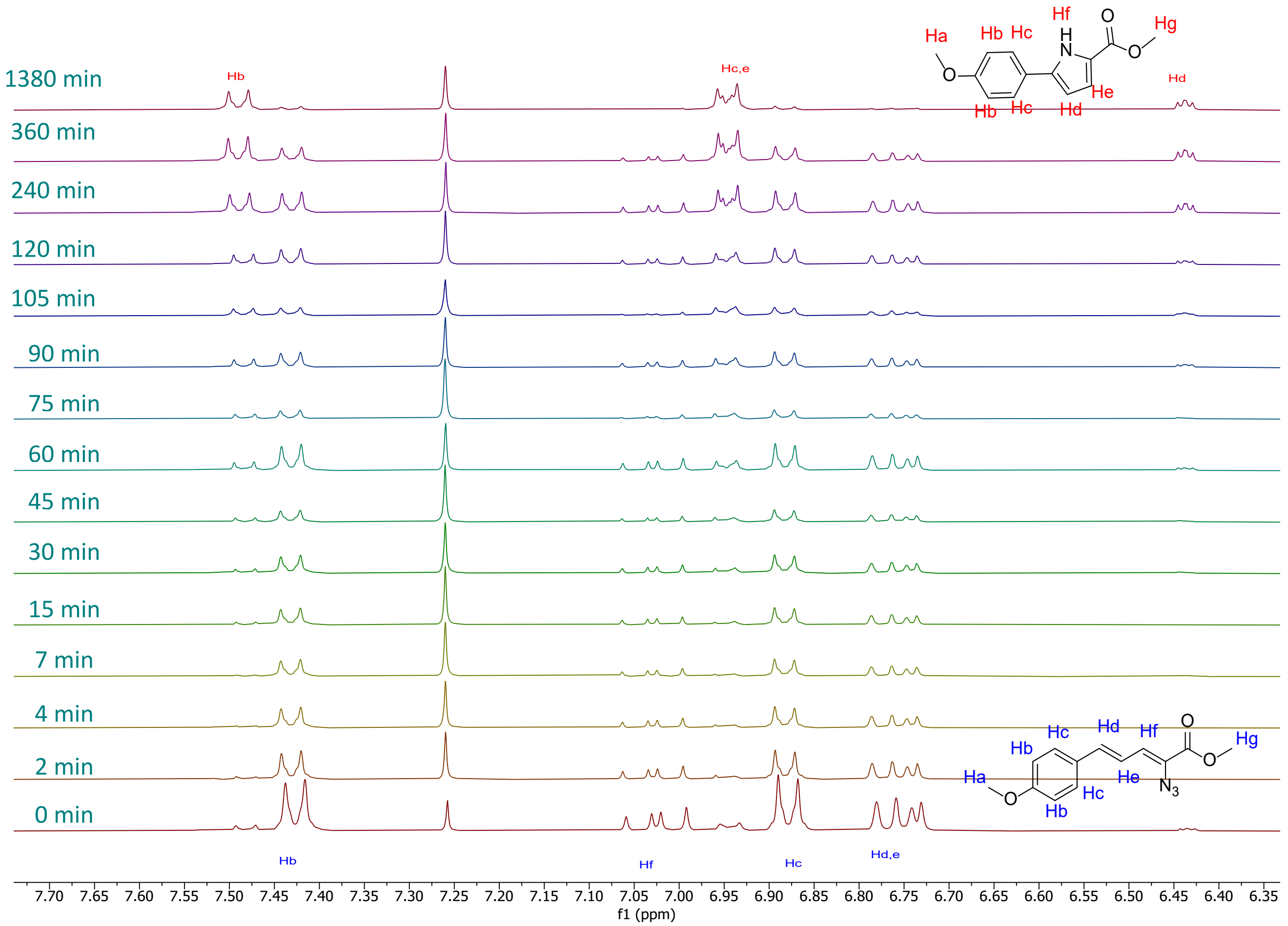

Figure S18. ^{1}H-NMR (400 MHz, $CDCl_3$) spectra of nanosuspension of crystalline **1a** as a function of irradiation time

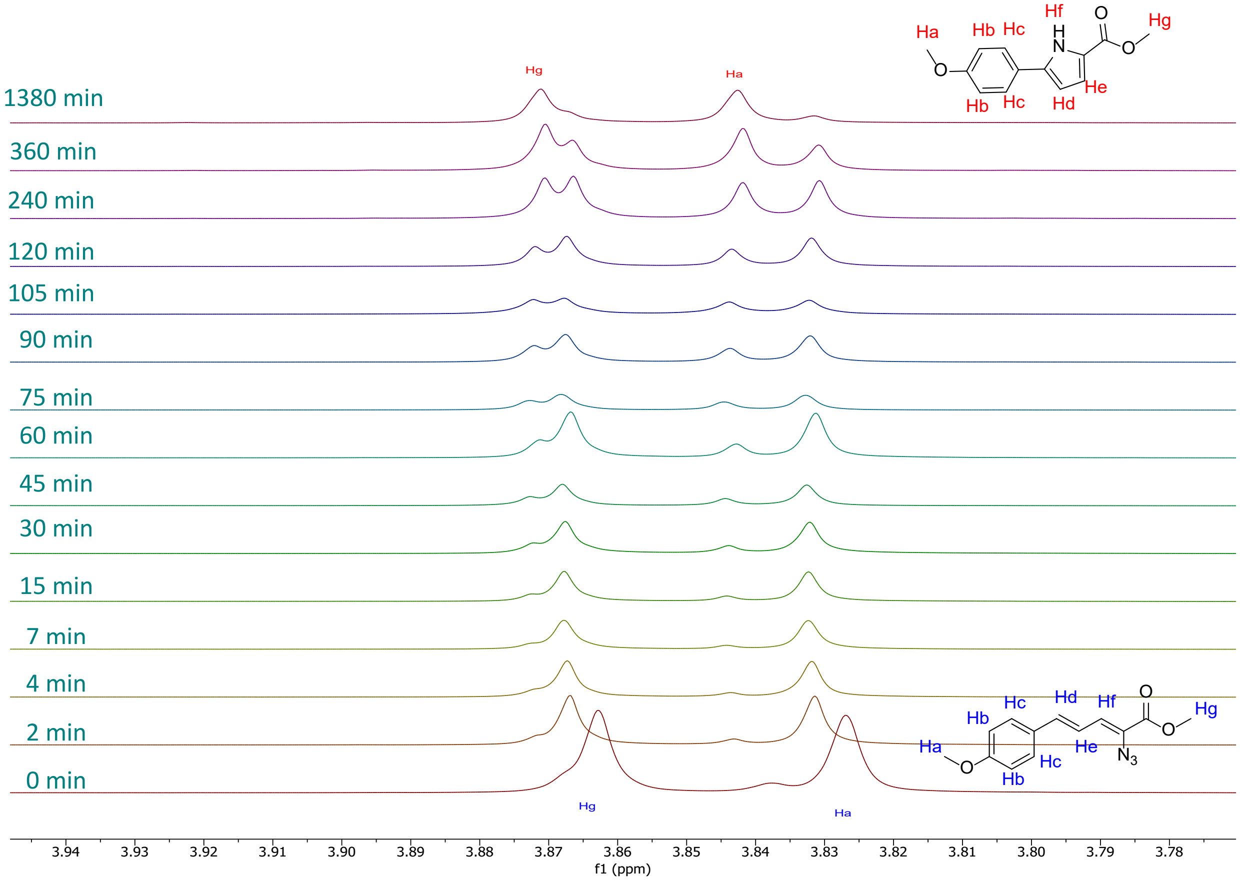

Figure S19. ^{1}H-NMR (400 MHz, $CDCl_3$) spectra of nanosuspension of crystalline **1a** as a function of irradiation time - extension

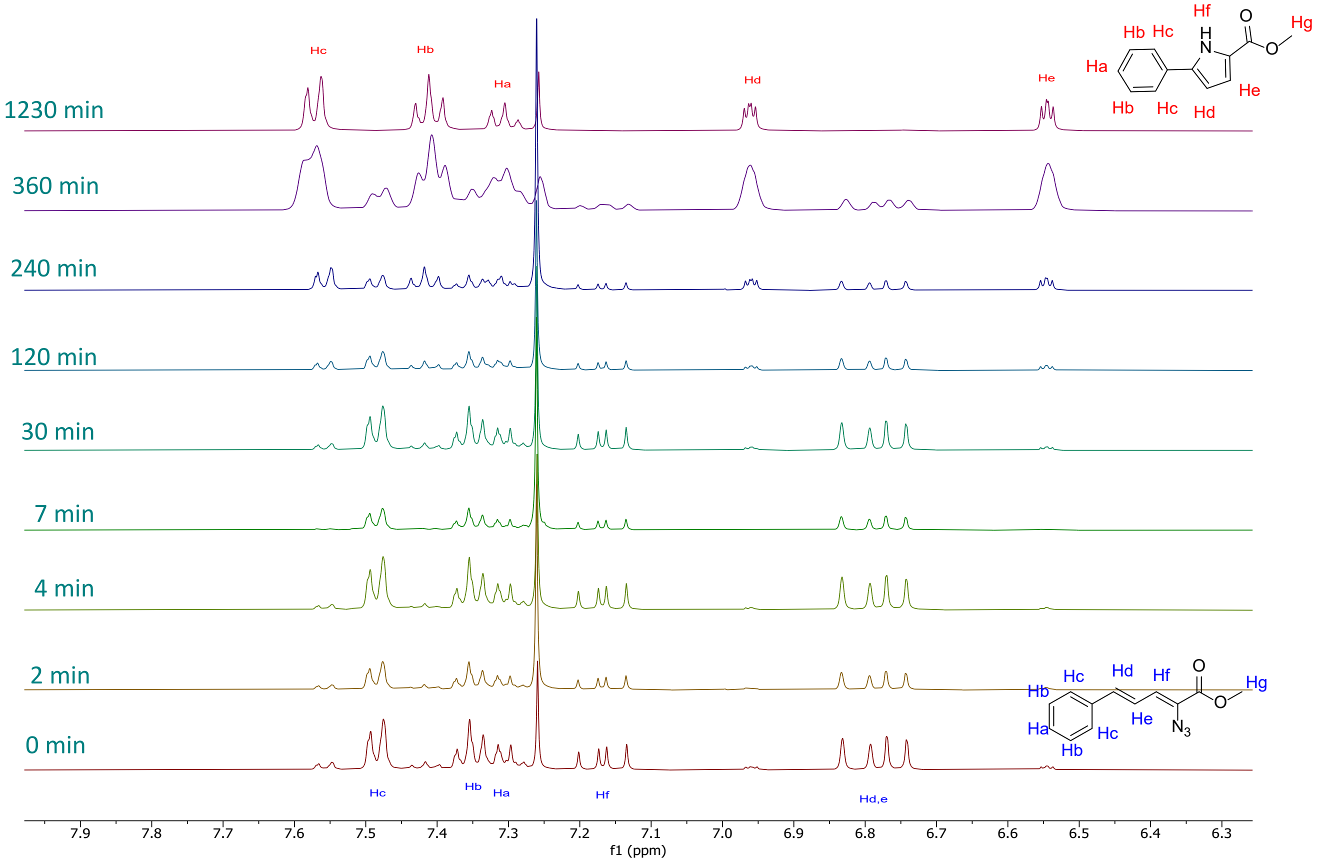

Figure S20. ^{1}H-NMR (400 MHz, $CDCl_3$) spectra of nanosuspension of crystalline **1b** as a function of irradiation time

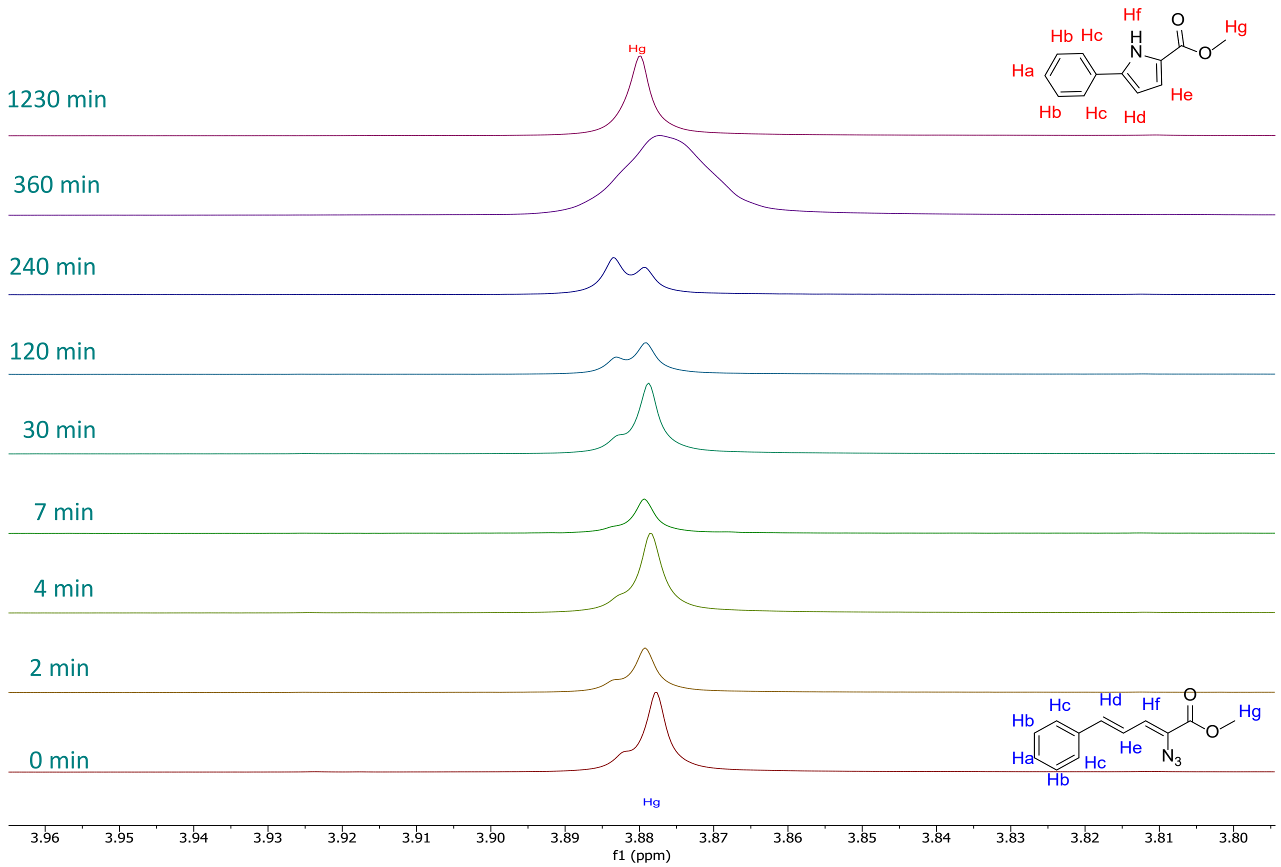

Figure S21. ^{1}H-NMR (400 MHz, $CDCl_3$) spectra of nanosuspension of crystalline **1b** as a function of irradiation time – extension

5. Preparative Product Studies

5.1. Preparative Product Studies in Crystals of **1a**

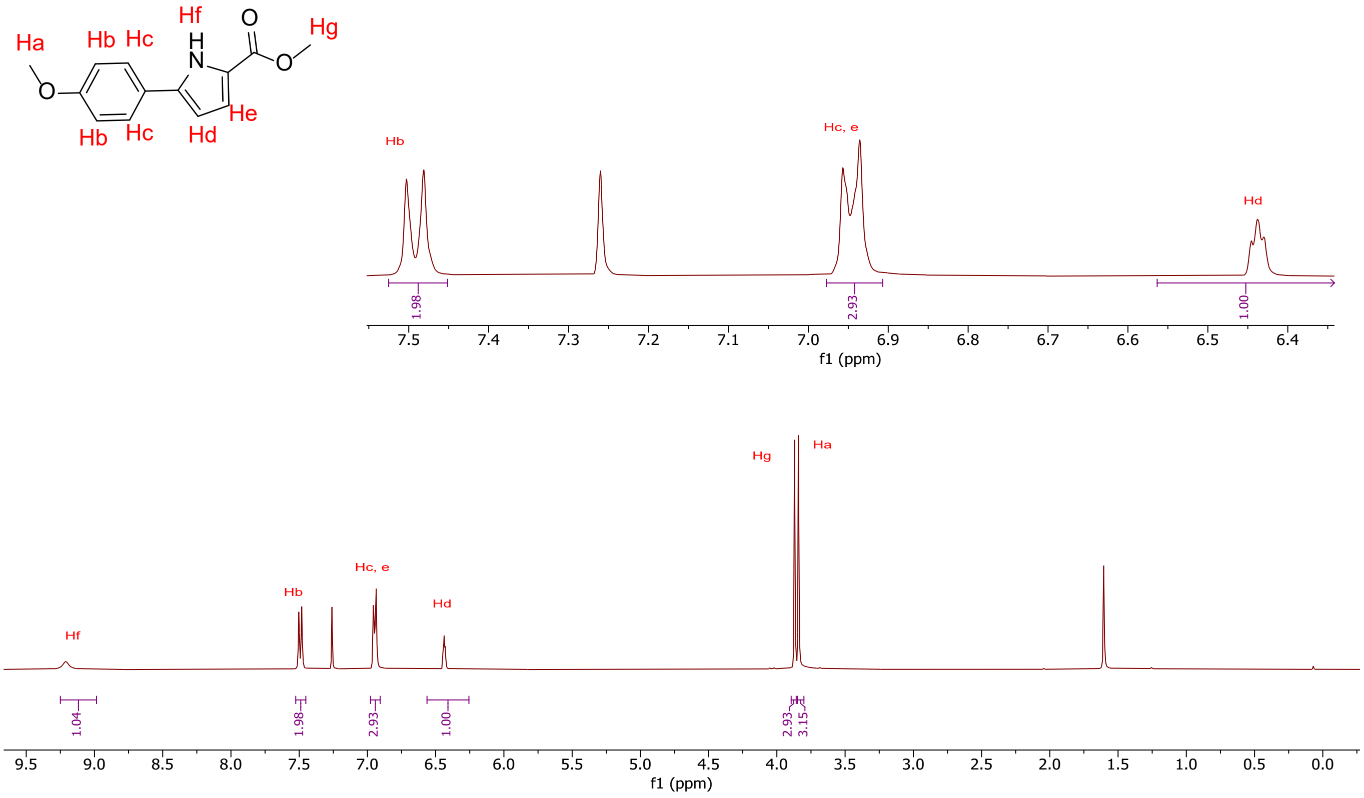

Figure S22. ^{1}H-NMR (400 MHz, $CDCl_3$) spectra after irradiating (365 nm LED, 18h) crystals of **1a** (50 mg)

5.2. Preparative Product Studies in Crystals of **1b**

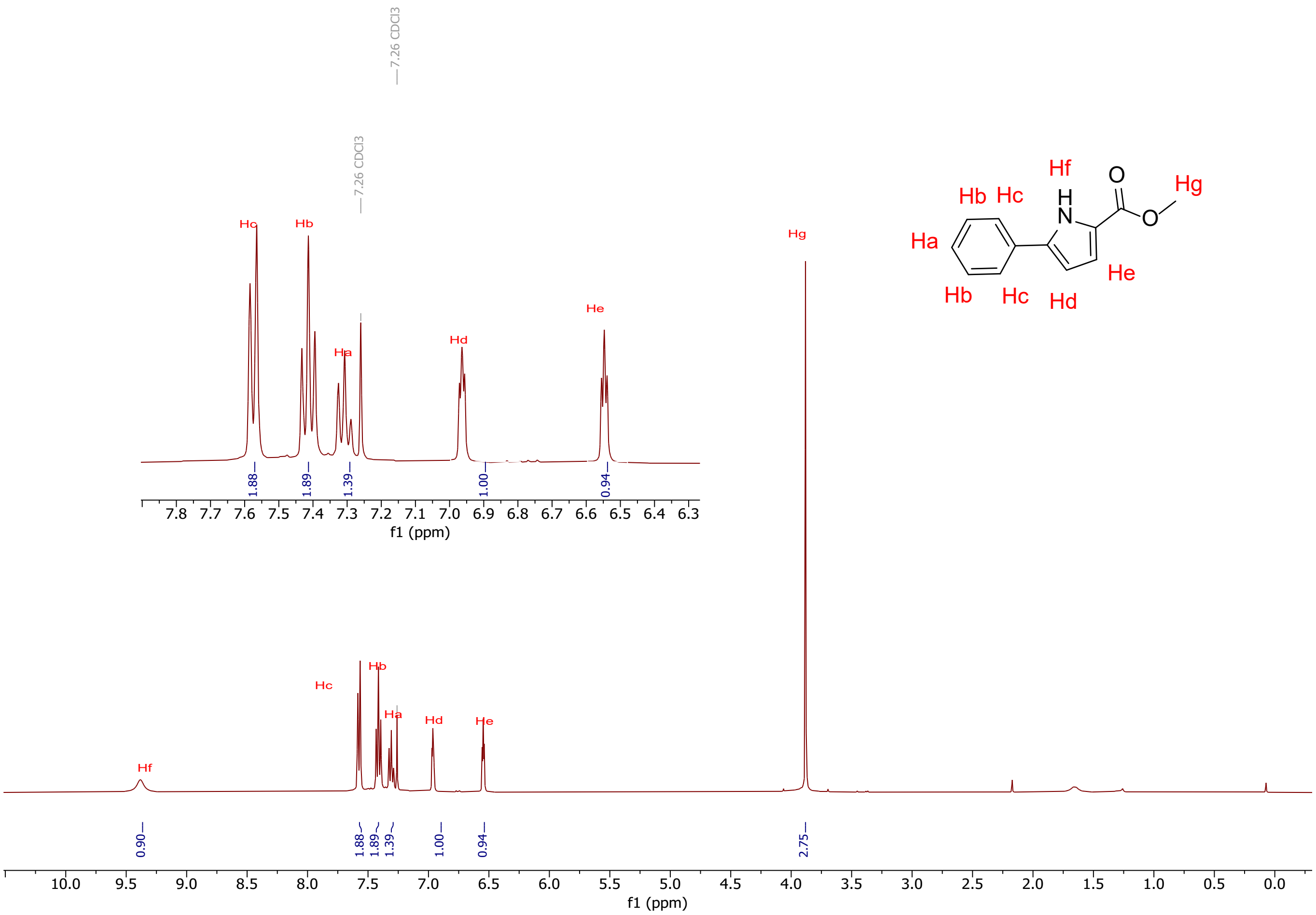

Figure S23. ^{1}H-NMR (400 MHz, $CDCl_3$) spectra after irradiating (365 nm LED, 16h) crystals of **1b** (50 mg)

5.3. Preparative Product Studies in Solution of **1a**

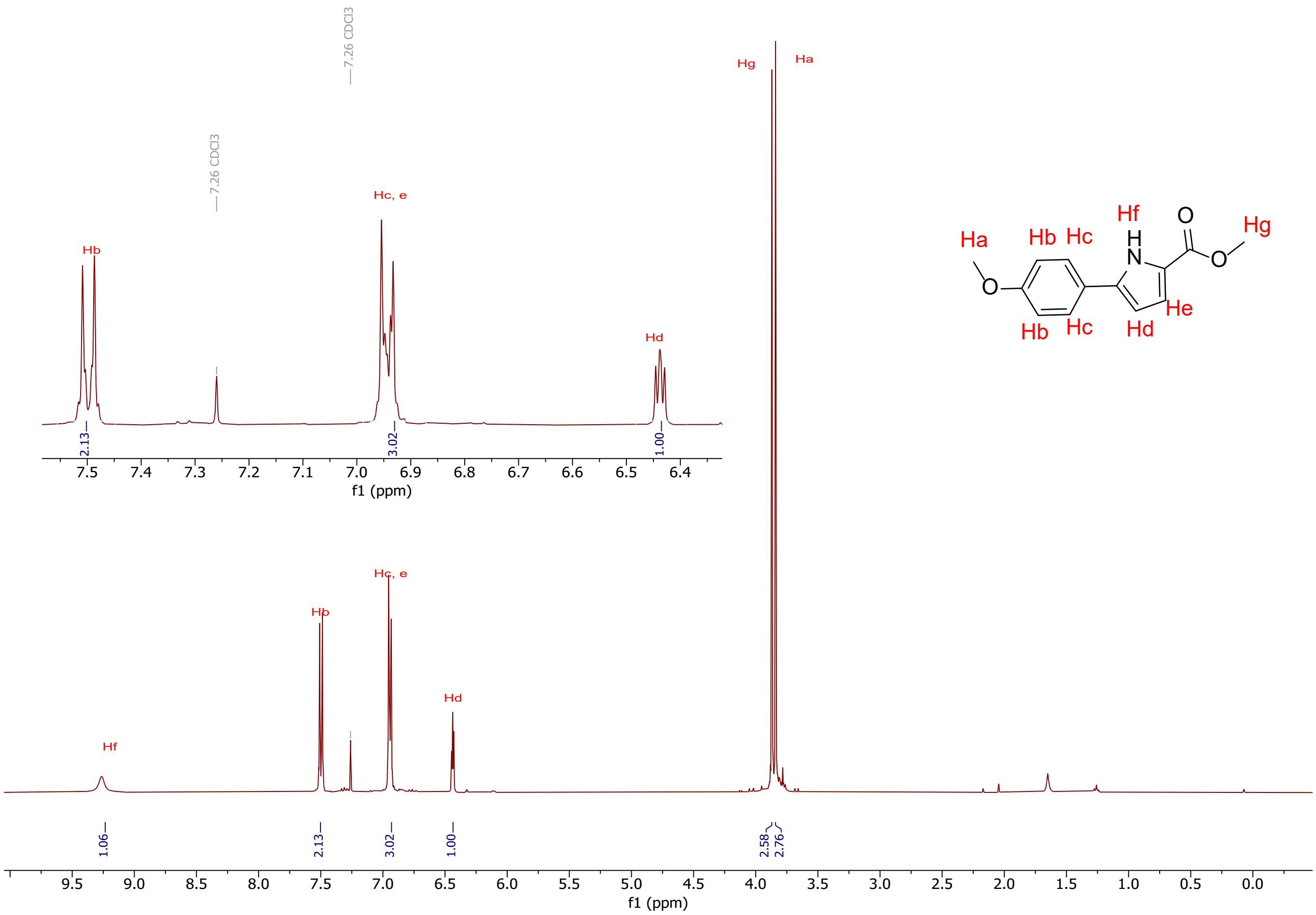

Figure S24. ^{1}H-NMR (400 MHz, $CDCl_3$) spectra after irradiating (365 nm LED, 12 min) **1a** (10.8 mg) in $CDCl_3$ (0.4 mL)

5.4. Preparative Product Studies of Azide **1b** in Solution

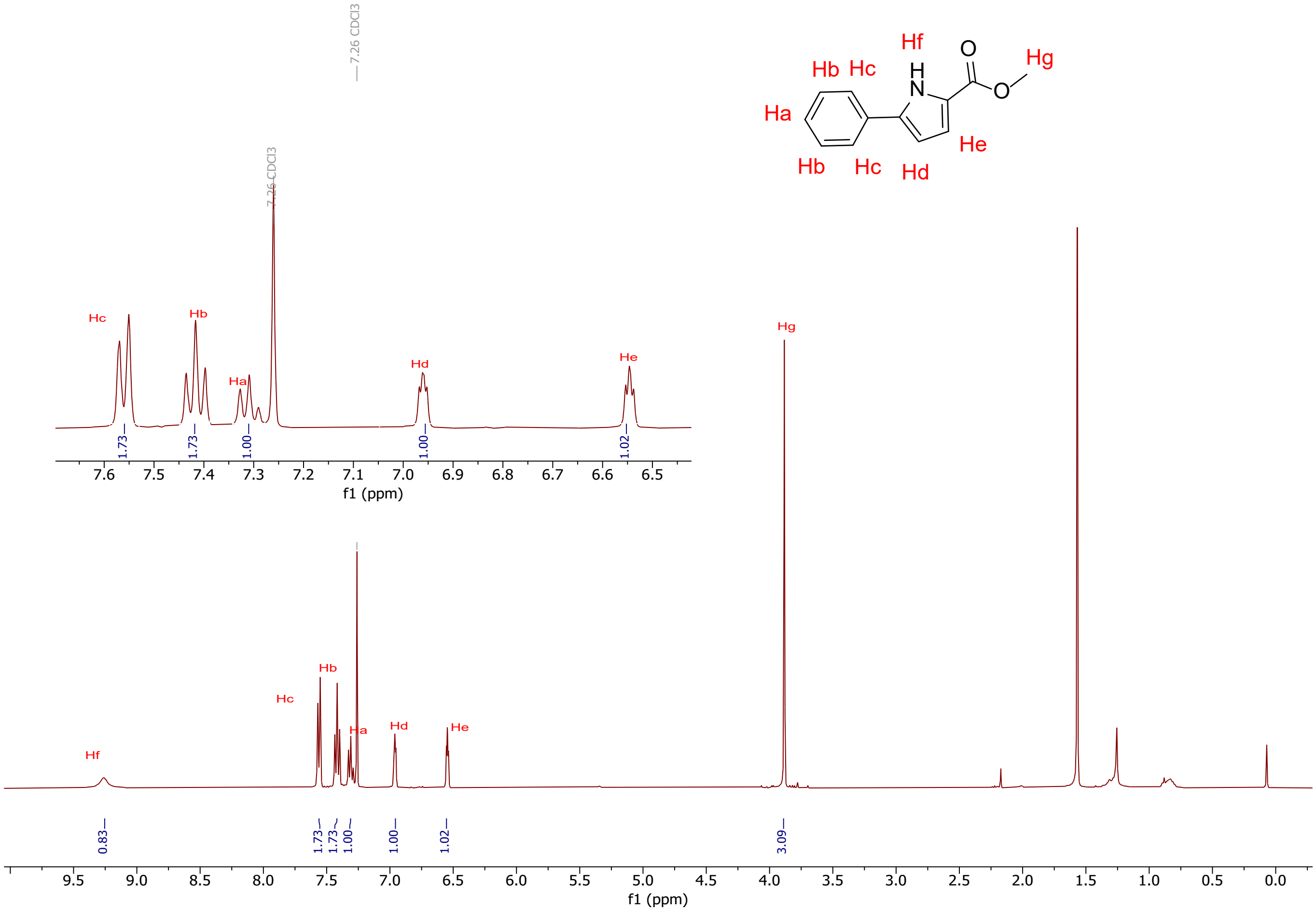

Figure S25. ^{1}H-NMR (400 MHz, $CDCl_3$) spectra after irradiating (365 nm LED, 3 min) **1b** (1.5 mg) in $CDCl_3$ (0.4 mL)

6. ^{1}H NMR Spectra of Crystalline 1 after Exposure to Sunlight

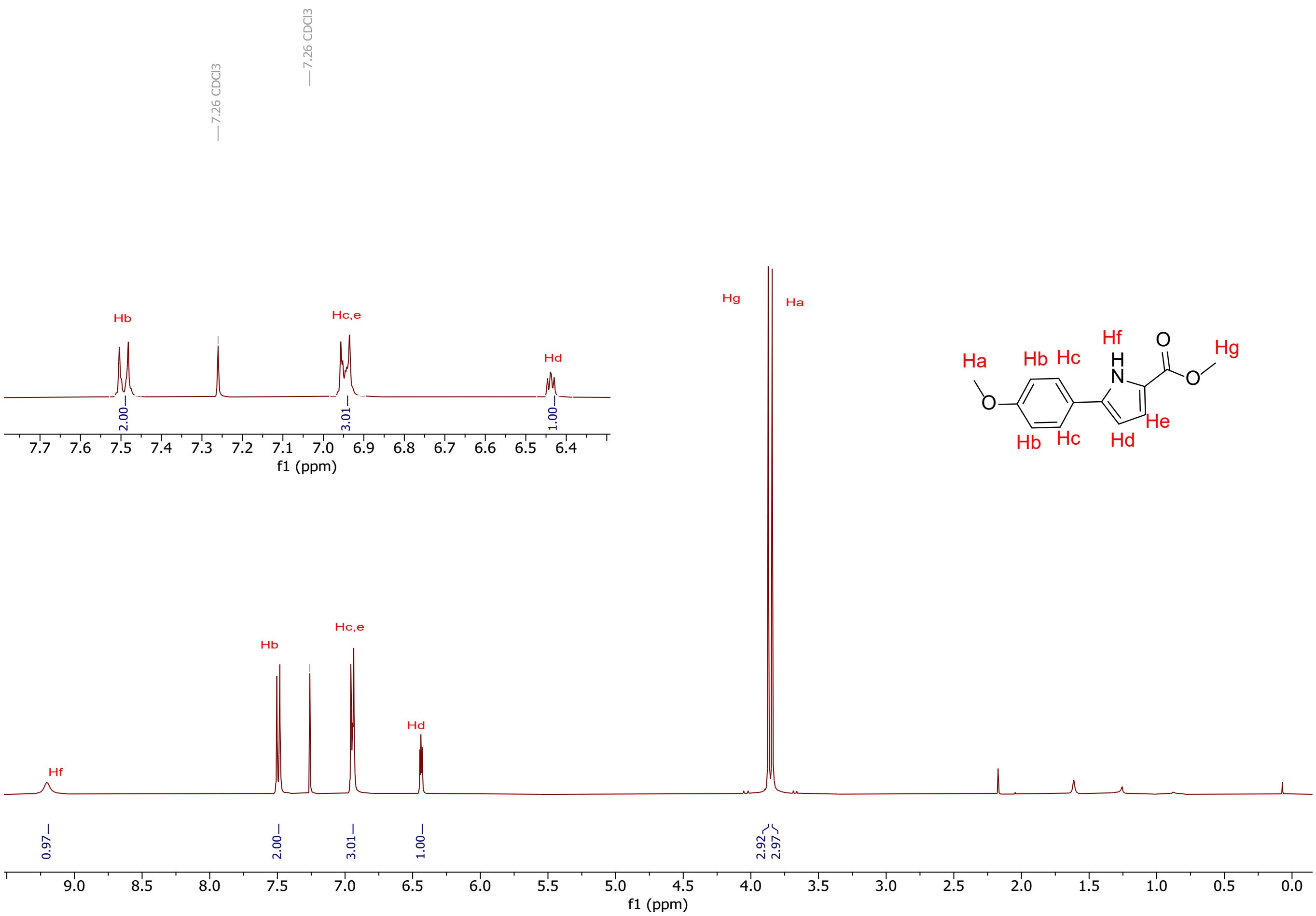

Figure S26. ^{1}H-NMR (400 MHz, $CDCl_3$) spectra of crystalline **1a** after exposure to sunlight for 22 hours

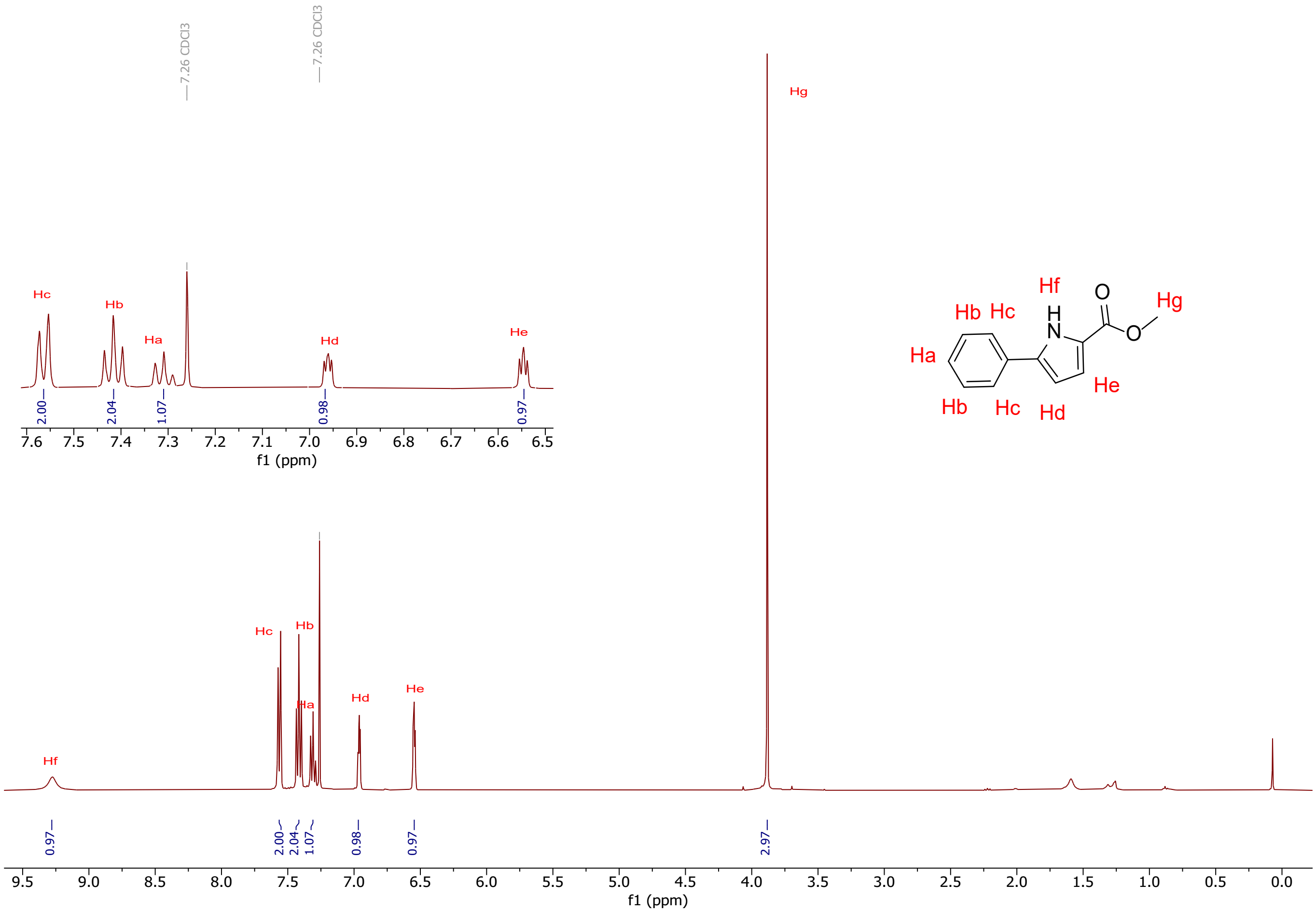

Figure S27. ^{1}H-NMR (400 MHz, $CDCl_3$) spectra of crystalline **1b** after exposure to sunlight for 22 hours

7. Laser Flash Photolysis

7.1. Laser Flash Photolysis of 1b in acetonitrile

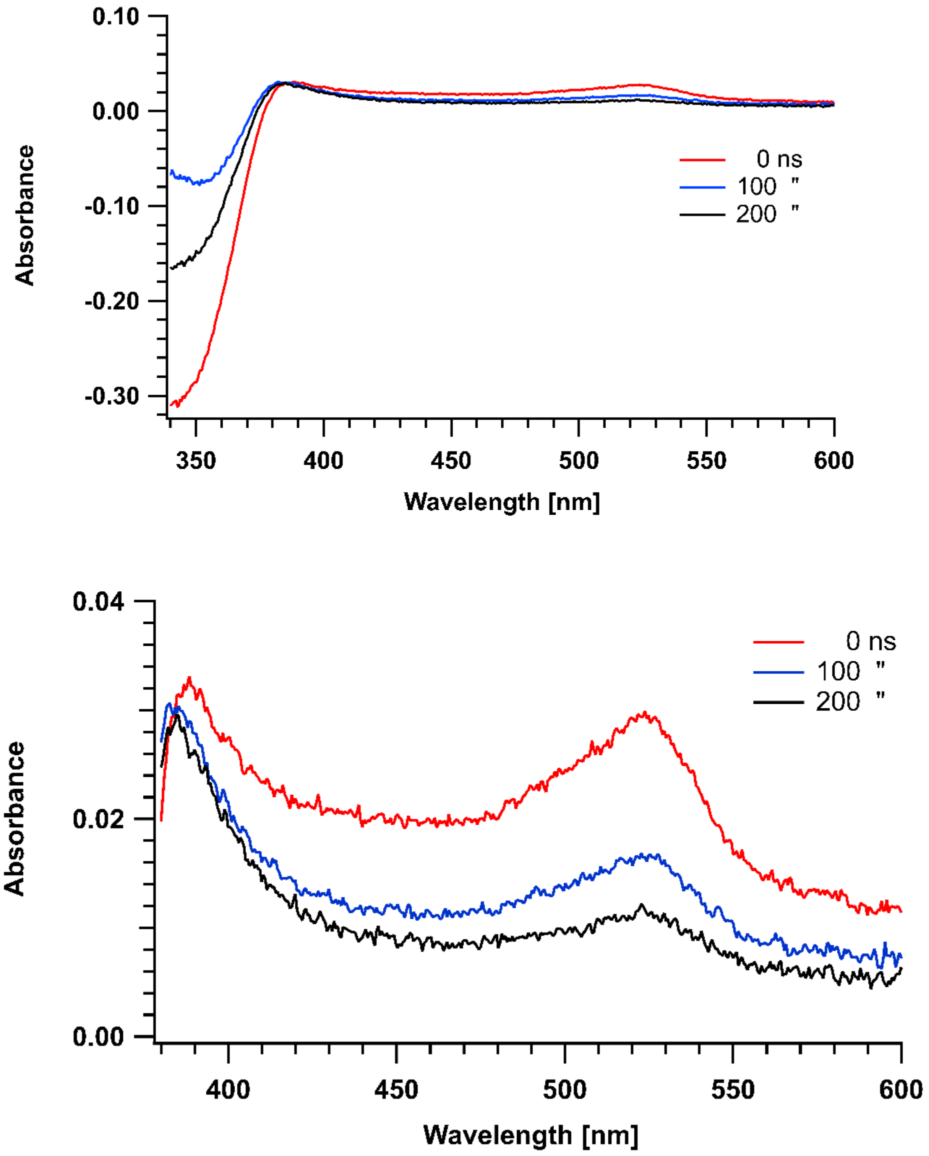

Figure S28. A) Transient Spectra obtained from laser flash photolysis of **1b** in argon-saturated acetonitrile, B) extension

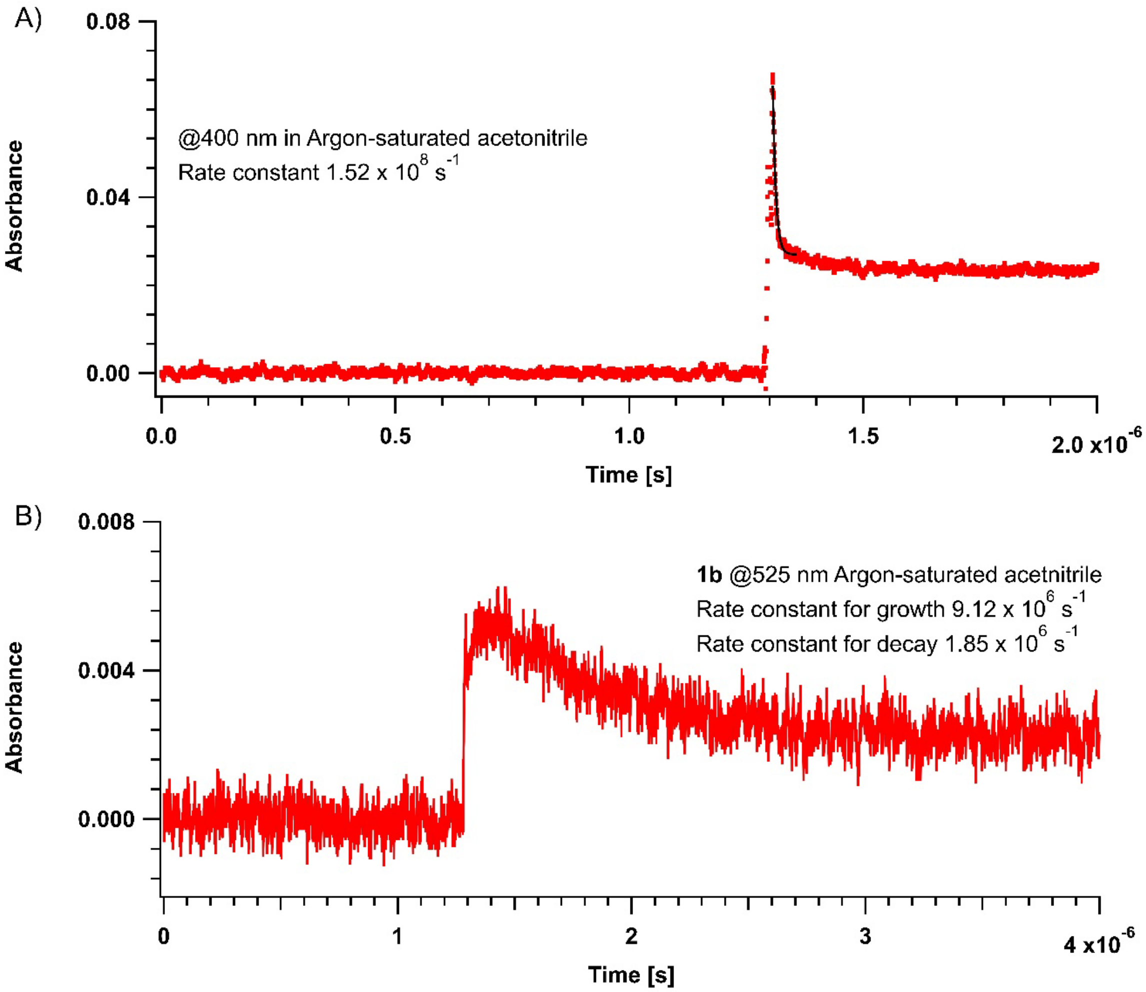

Figure S29. Kinetic traces from laser flash photolysis of **1b** in argon-saturated acetonitrile at A) 400 nm and B) at 525 nm.

7.2. Laser Flash Photolysis of 1a in nanosuspension

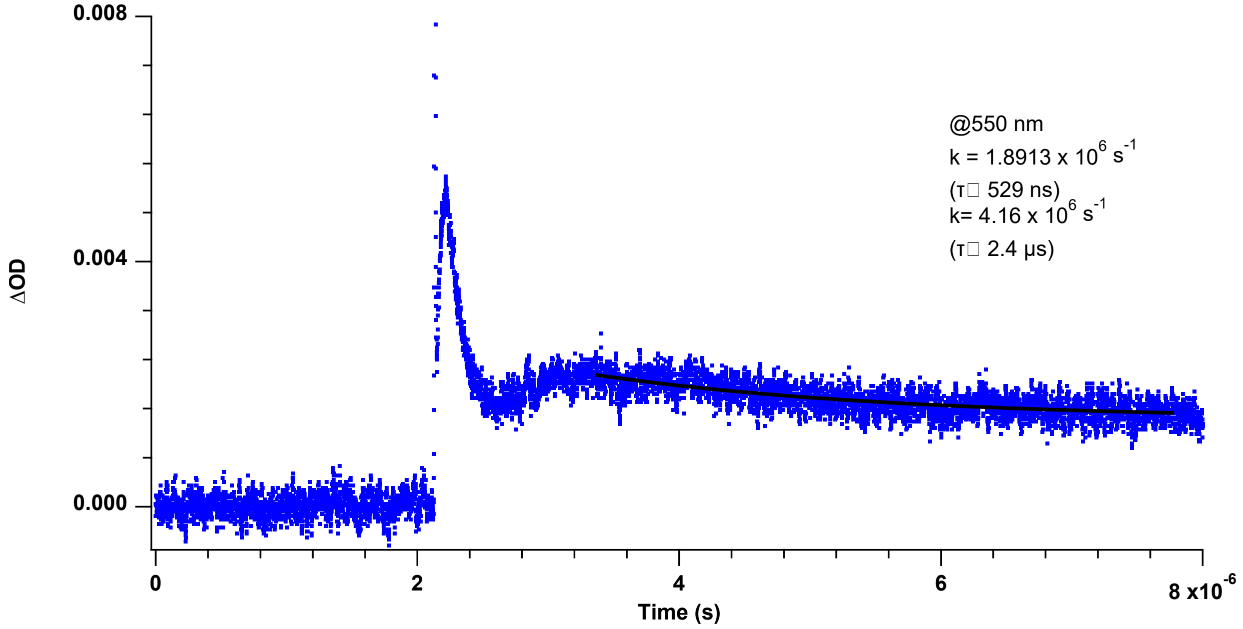

Figure S30. Kinetic traces from laser flash photolysis of **1a** in argon-saturated nanosuspension at 550 nm

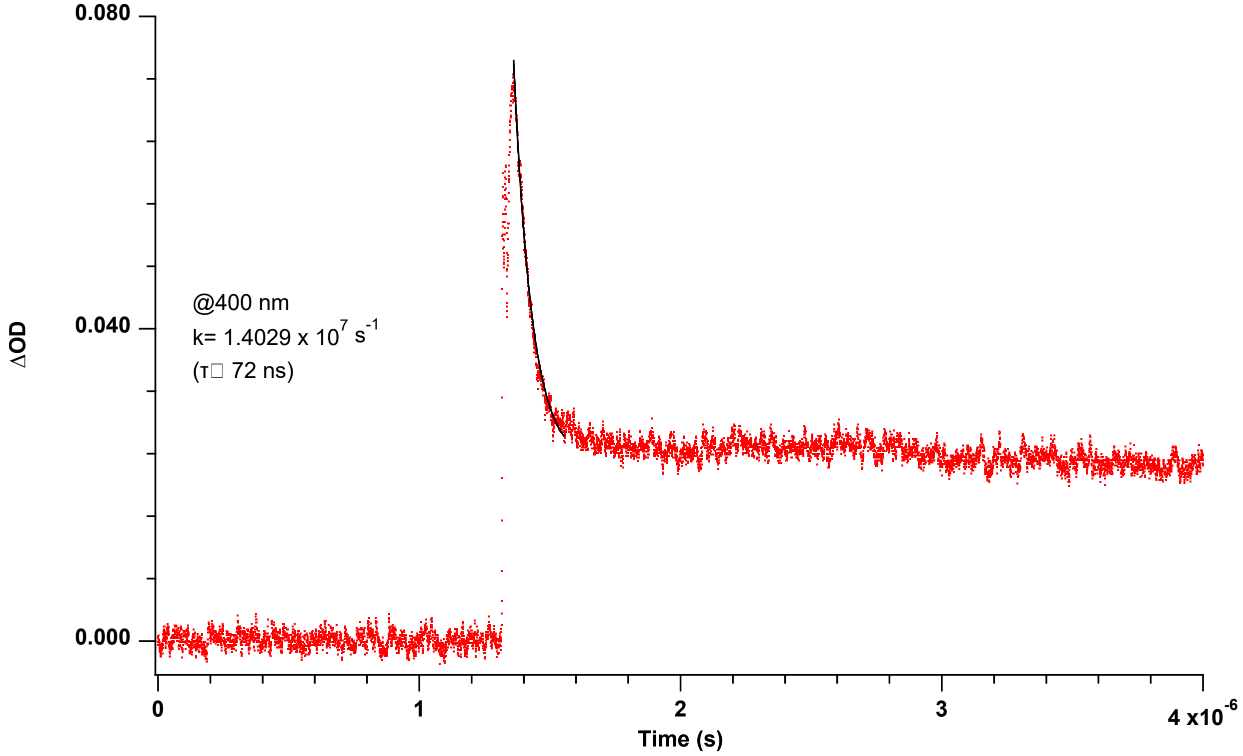

Figure S31. Kinetic traces from laser flash photolysis of **1a** in argon-saturated nanosuspension at 400 nm

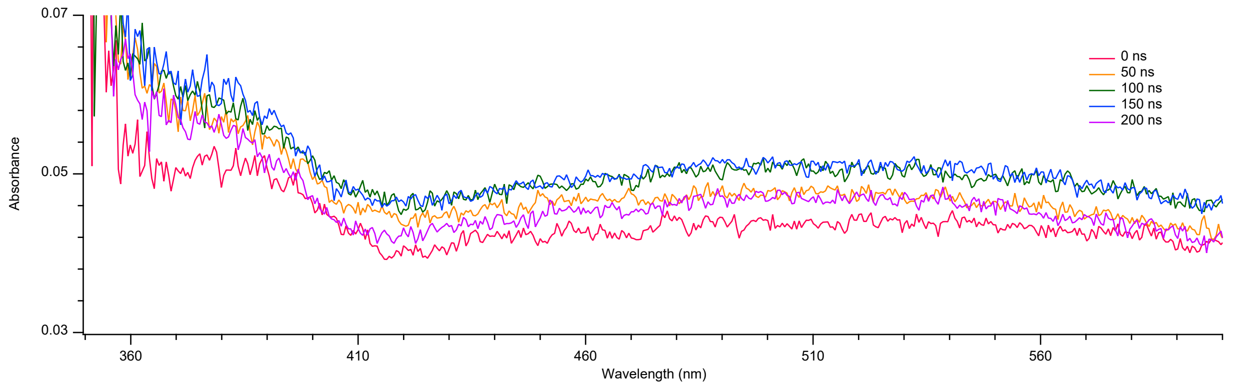

Figure S32. Transient Spectra obtained from laser flash photolysis of **1a** in argon-saturated nanosuspension

7.3. Laser Flash Photolysis of 1b in nanosuspension

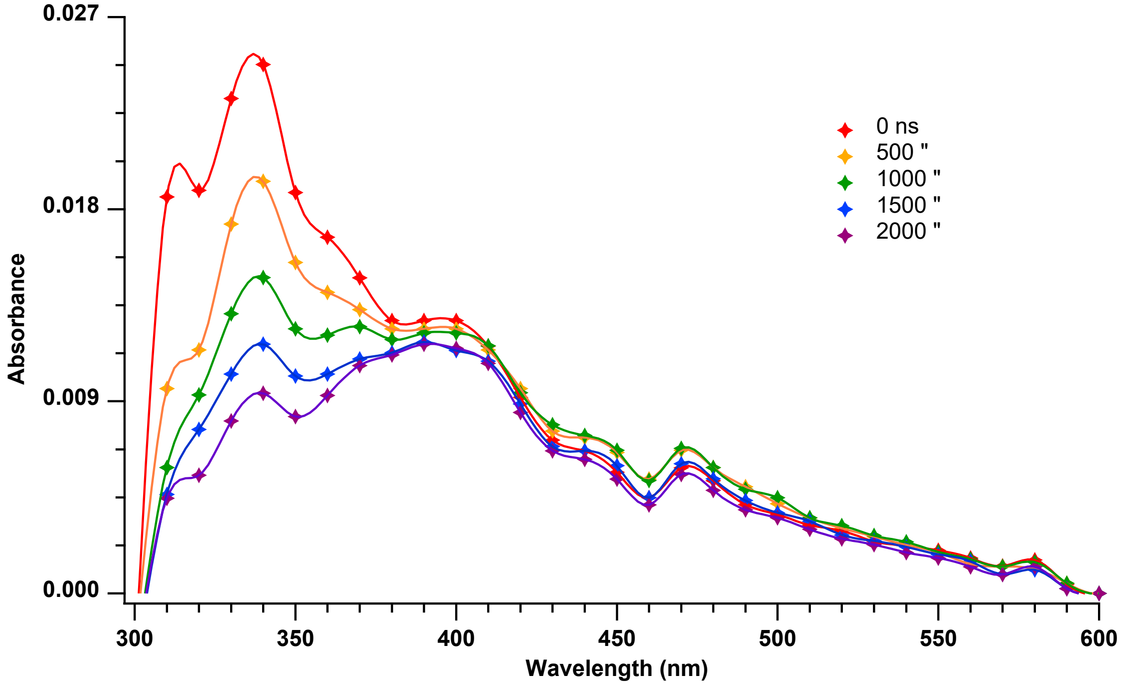

Figure S33. Transient Spectra obtained by laser flash of **1b** in argon-saturated nanosuspension using PMT

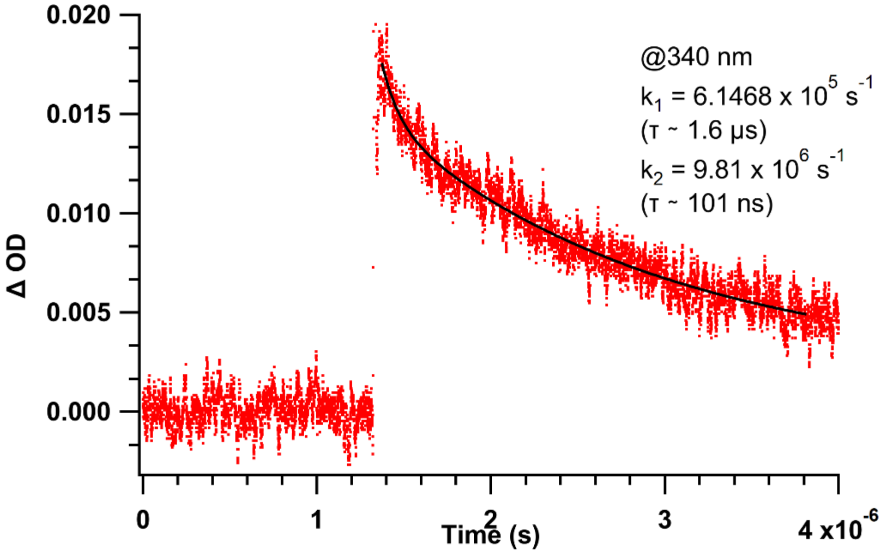

Figure **S**34. Kinetic trace at 340 nm obtained by laser flash of **1b** in argon-saturated nanosuspension

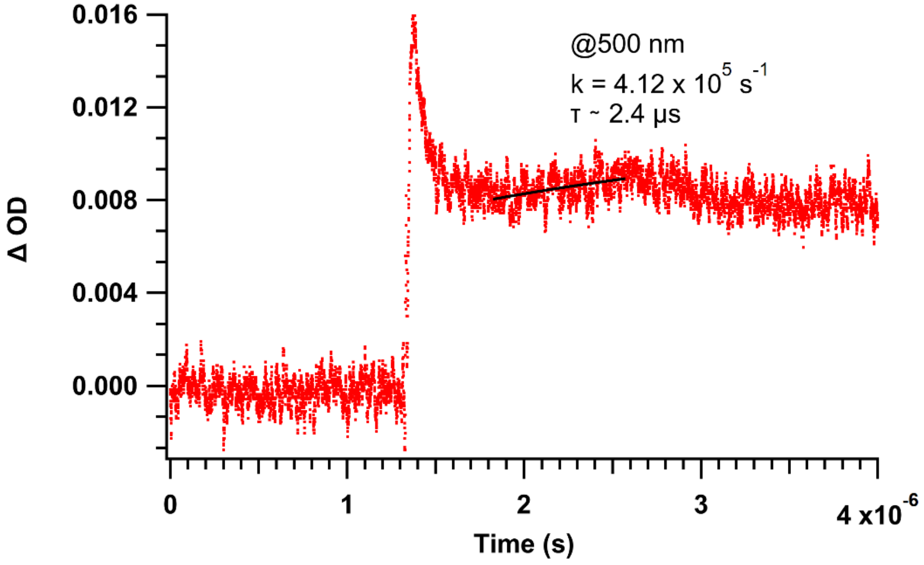

Figure S35**.** Kinetic trace at 500 nm showing the growth, obtained by laser flash of **1b** in argon-saturated nanosuspension

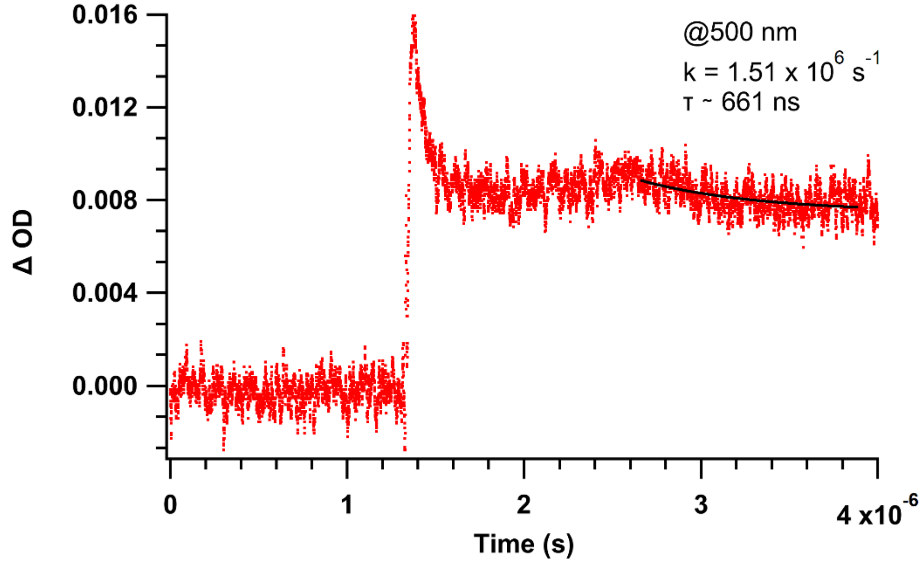

Figure S36. Kinetic trace at 500 nm showing the decay, obtained by laser flash of **1b** in argon-saturated nanosuspension

8. TD-DFT Spectra

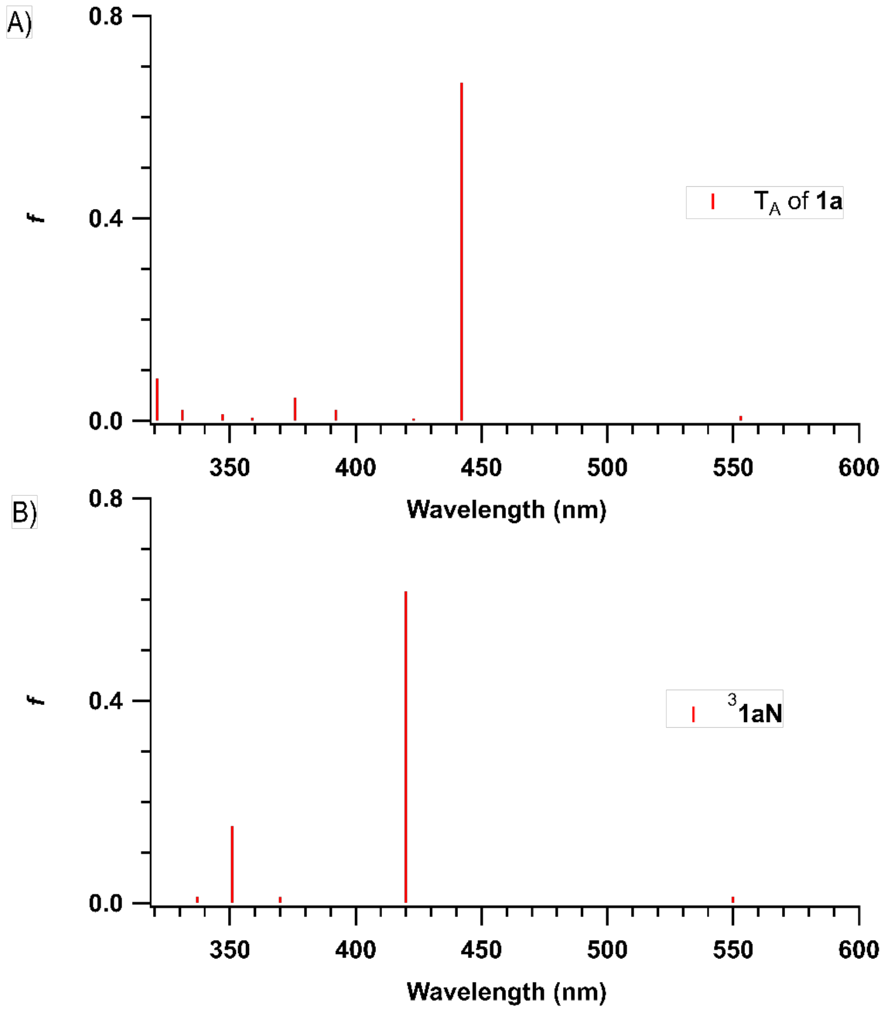

Figure S37. TD-DFT calculated electronic transition for A) TA of **1a** and B) nitrene ^{3}**1aN**

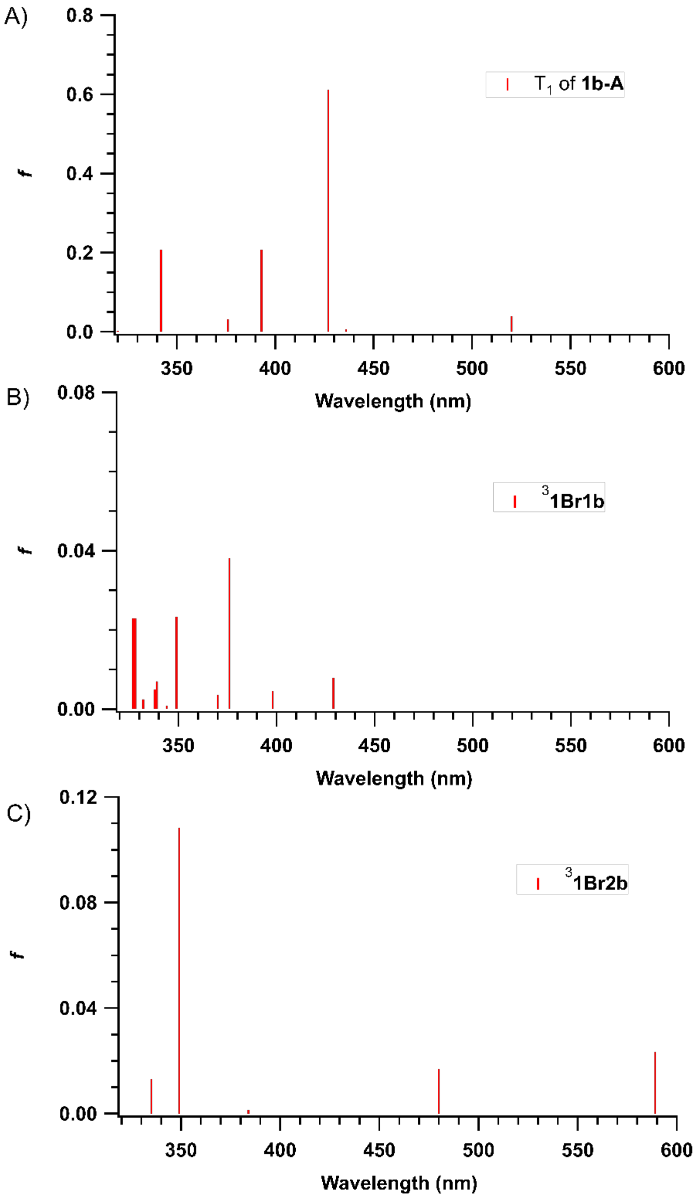

Figure S38. TD-DFT calculated electronic transition for A) T_1 of **1b**, B) ^{3}**Br1b** and C) ^{3}**1Br2b**

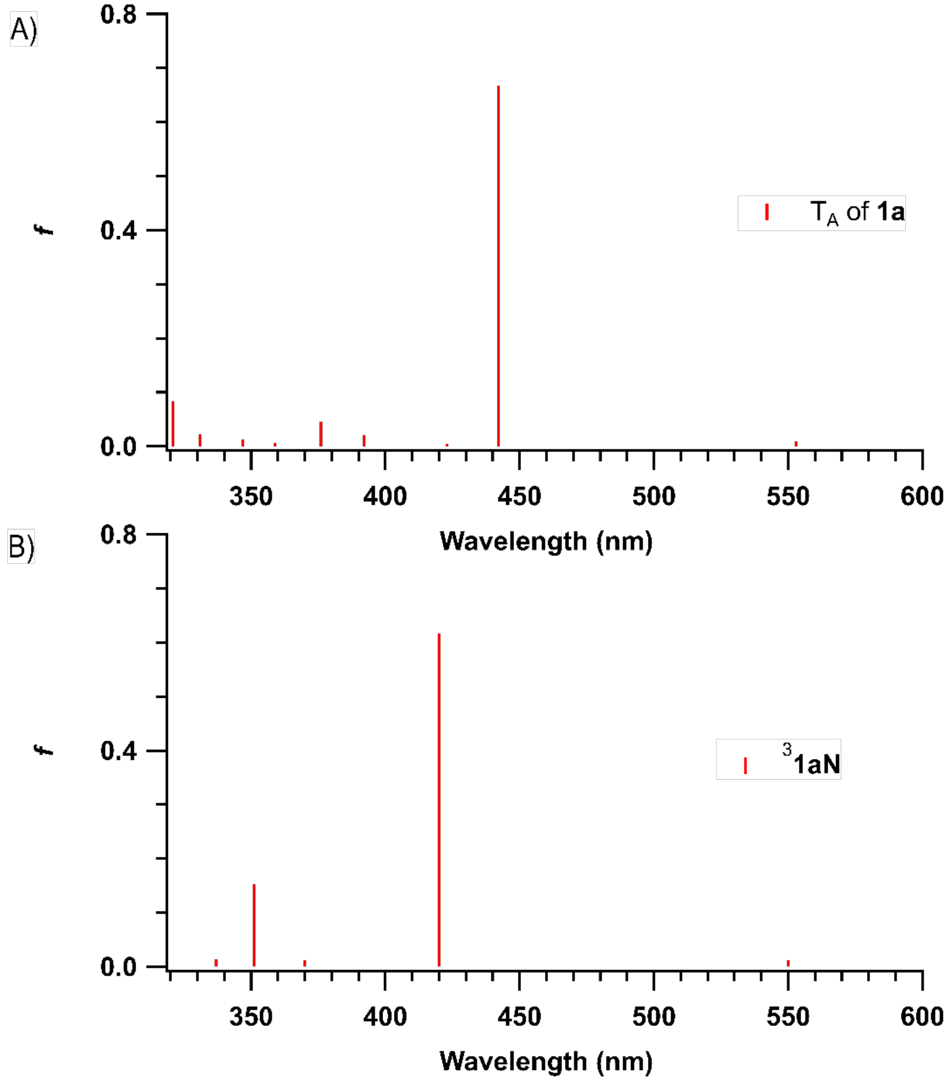

Figure S39. TD-DFT calculated electronic transition for A) T_A of **1a**, and B) ^{3}**1bN**

9. X-ray Structures

9.1. X-ray Structures of Azide 1a

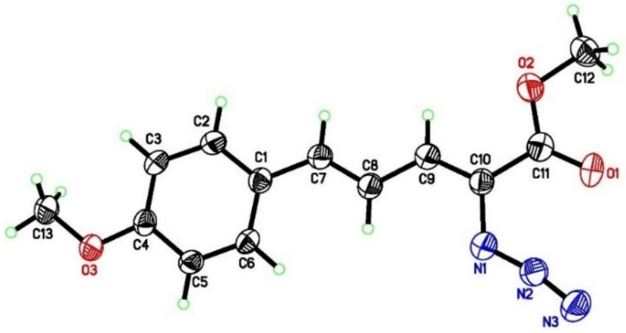

Figure S40. Crystal structure of azide **1a** (CCDC-2352521)

For X-ray examination and data collection, a suitable pale-yellow block-shaped crystal, with approximate dimensions 0.106 x 0.103 x 0.069 mm, was mounted in a loop with Paratone-N oil and transferred to the goniostat bathed in a cold stream. Intensity data were collected at 150K on a Bruker D8 Venture Mo-ImS Photon-II diffractometer, λ=0.71073Å. Data was measured in shutterless mode. The data frames were processed using the program SAINT. The data was corrected for decay, Lorentz and polarization effects as well as absorption and beam corrections based on the multi-scan technique.

The structure was solved by a combination of direct methods in SHELXTL and the difference Fourier technique and refined by full-matrix least squares on F^2 for reflections out to 0.83Å resolution. Non-hydrogen atoms were refined with anisotropic displacement parameters. H-atoms were located directly from the difference map and treated with a riding model. The H-atom isotropic displacement parameters were defined as a*U_{eq} (a=1.5 for methyl and 1.2 for all others) of the adjacent atom. The refinement converged with crystallographic agreement factors of R1=4.93%, wR2=12.62% for 1719 reflections with I>2σ(I) (R1=7.44%, wR2=14.77% for all data) and 174 variable parameters. (20093)

Table S1. Crystal data and structure refinement for $C_{13}H_{13}N_3O_3$ **1a** at 150K. (CCDC-2352521)

Empirical formula	$C_{13}H_{13}N_3O_3$ 259.26	
Formula weight		
**Temperature**	**150(2) K**	
Wavelength Crystal	0.71073 Å	
system Space group	Orthorhombic Pbca	
Unit cell dimensions	a = 13.0459(14) Å	α = 90°
	b = 8.3080(9) Å	β = 90°
	c = 23.886(3) Å	γ = 90°
Volume	2588.9(5) $Å^3$	
Z	8	
Density (calculated)	1.330 Mg/m^3	
Absorption coefficient	0.097 mm^{-1}	
F(000)	1088	
Crystal size	0.182 x 0.135 x 0.070 mm^3	
θ range for data collection	1.705 to 27.531°	
Index ranges	-16 ≤ h ≤ 16, -10 ≤ k ≤ 10, -31 ≤ l ≤ 31	
Reflections collected	33715	
Independent reflections	2977 [R_{int} = 0.0643]	
Completeness to θ = 25.242°	100.0 %	
Absorption correction	Numerical	
Max. and min. transmission	0.862 and 0.802	
Refinement method	Full-matrix least-squares on F^2	
Data / restraints / parameters	2977 / 0 / 174	
Goodness-of-fit on F^2	1.033	
Final R indices [I>2σ(I)]	R1 = 0.0401, wR2 = 0.0798	
R indices (all data)	R1 = 0.0783, wR2 = 0.0936	
Largest diff. peak and hole	0.199 and -0.189 $eÅ^{-3}$	

Table S2. Atomic coordinates [x104] and equivalent isotropic displacement parameters [$Å^2x10^3$] for **1a** $C_{13}H_{13}N_3O_3$ at 150K. U(eq) is defined as one third of the trace of the orthogonalized Uij tensor.

	x	y	Z	U(eq)
O(1)	974(1)	6686(1)	9065(1)	37(1)
O(2)	440(1)	7256(1)	8190(1)	33(1)
O(3)	5761(1)	1717(1)	5501(1)	36(1)
N(1)	2616(1)	4797(2)	8624(1)	34(1)
N(2)	2467(1)	4646(2)	9138(1)	33(1)
N(3)	2475(1)	4362(2)	9599(1)	42(1)
C(1)	1918(1)	5669(2)	8290(1)	27(1)
C(2)	2061(1)	5694(2)	7732(1)	28(1)
C(3)	2864(1)	4904(2)	7430(1)	28(1)
C(4)	2913(1)	4926(2)	6868(1)	28(1)
C(5)	3662(1)	4118(2)	6513(1)	26(1)
C(6)	3521(1)	4090(2)	5934(1)	31(1)
C(7)	4197(1)	3309(2)	5578(1)	30(1)
C(8)	5046(1)	2537(2)	5804(1)	27(1)
C(9)	5212(1)	2564(2)	6382(1)	28(1)
C(10)	4532(1)	3342(2)	6728(1)	26(1)
C(11)	1076(1)	6569(2)	8564(1)	28(1)
C(12)	-375(1)	8221(2)	8425(1)	40(1)
C(13)	5691(1)	1836(2)	4902(1)	43(1)
H(2A)	1581	6295	7518	34
H(3A)	3379	4346	7633	33
H(4A)	2402	5537	6681	34
H(6A)	2943	4624	5779	37
H(7A)	4081	3302	5185	36
H(9A)	5795	2044	6536	33
H(10A)	4654	3355	7120	32
H(12A)	-788	8680	8122	60
H(12B)	-79	9093	8649	60
H(12C)	-810	7546	8663	60
H(13A)	6258	1241	4731	64
H(13B)	5729	2970	4791	64
H(13C)	5037	1380	4777	64

Table S3. Bond lengths [Å] and angles [°] for **1a** $C_{13}H_{13}N_3O_3$ at 150K.

O(1)-C(11)	1.2078(18)	O(2)-C(11)	1.3456(19)
O(2)-C(12)	1.4451(19)	O(3)-C(8)	1.3632(18)
O(3)-C(13)	1.4356(18)	N(1)-N(2)	1.2490(17)
N(1)-C(1)	1.411(2)	N(2)-N(3)	1.1278(17)
C(1)-C(2)	1.346(2)	C(1)-C(11)	1.481(2)
C(2)-C(3)	1.431(2)	C(3)-C(4)	1.343(2)
C(4)-C(5)	1.457(2)	C(5)-C(6)	1.396(2)
C(5)-C(10)	1.403(2)	C(6)-C(7)	1.387(2)
C(7)-C(8)	1.390(2)	C(8)-C(9)	1.397(2)
C(9)-C(10)	1.373(2)		

C(11)-O(2)-C(12)	115.54(12)	C(8)-O(3)-C(13)	116.83(12)
N(2)-N(1)-C(1)	120.46(13)	N(3)-N(2)-N(1)	168.65(17)
C(2)-C(1)-N(1)	118.57(14)	C(2)-C(1)-C(11)	122.19(14)
N(1)-C(1)-C(11)	119.21(13)	C(1)-C(2)-C(3)	126.42(15)
C(4)-C(3)-C(2)	122.18(15)	C(3)-C(4)-C(5)	127.39(15)
C(6)-C(5)-C(10)	117.43(14)	C(6)-C(5)-C(4)	119.78(14)
C(10)-C(5)-C(4)	122.79(13)	C(7)-C(6)-C(5)	122.22(15)
C(6)-C(7)-C(8)	118.90(14)	O(3)-C(8)-C(7)	124.65(13)
O(3)-C(8)-C(9)	115.33(14)	C(7)-C(8)-C(9)	120.02(14)
C(10)-C(9)-C(8)	120.18(15)	C(9)-C(10)-C(5)	121.23(14)
O(1)-C(11)-O(2)	123.77(14)	O(1)-C(11)-C(1)	124.04(15)
O(2)-C(11)-C(1)	112.19(13)		

Table S4. Torsion angles [°] for **1a** $C_{13}H_{13}N_3O_3$ at 150 K.

C(1)-N(1)-N(2)-N(3)	178.3(8)	N(2)-N(1)-C(1)-C(2)	-174.66(15)
N(2)-N(1)-C(1)-C(11)	7.4(2)	N(1)-C(1)-C(2)-C(3)	0.3(2)
C(11)-C(1)-C(2)-C(3)	178.22(14)	C(1)-C(2)-C(3)-C(4)	176.32(16)
C(2)-C(3)-C(4)-C(5)	-177.23(15)	C(3)-C(4)-C(5)-C(6)	171.41(16)
C(3)-C(4)-C(5)-C(10)	-8.2(3)	C(10)-C(5)-C(6)-C(7)	1.1(2)
C(4)-C(5)-C(6)-C(7)	-178.54(15)	C(5)-C(6)-C(7)-C(8)	-0.4(2)
C(13)-O(3)-C(8)-C(7)	8.0(2)	C(13)-O(3)-C(8)-C(9)	-172.22(15)
C(6)-C(7)-C(8)-O(3)	179.37(15)	C(6)-C(7)-C(8)-C(9)	-0.4(2)
O(3)-C(8)-C(9)-C(10)	-179.27(14)	C(7)-C(8)-C(9)-C(10)	0.5(2)
C(8)-C(9)-C(10)-C(5)	0.2(2)	C(6)-C(5)-C(10)-C(9)	-0.9(2)
C(4)-C(5)-C(10)-C(9)	178.66(14)	C(12)-O(2)-C(11)-O(1)	2.2(2)
C(12)-O(2)-C(11)-C(1)	-177.15(13)	C(2)-C(1)-C(11)-O(1)	-173.48(16)
N(1)-C(1)-C(11)-O(1)	4.4(2)	C(2)-C(1)-C(11)-O(2)	5.9(2)
N(1)-C(1)-C(11)-O(2)	-176.23(13)		

Table S5. Anisotropic displacement parameters [Å^{2}x10^3] for **1a** $C_{13}H_{13}N_3O_3$ at 150K. The anisotropic displacement factor exponent takes the form: -2π2[h 2a*2U11+... +2hka*b*U12]

	U_{11}	U_{22}	U_{33}	U_{23}	U_{13}	U_{12}
O(1)	42(1)	42(1)	26(1)	-3(1)	6(1)	4(1)
O(2)	33(1)	35(1)	29(1)	1(1)	3(1)	6(1)
O(3)	41(1)	46(1)	21(1)	0(1)	3(1)	14(1)
N(1)	34(1)	46(1)	22(1)	-1(1)	2(1)	6(1)
N(2)	30(1)	38(1)	32(1)	0(1)	1(1)	-1(1)
N(3)	43(1)	54(1)	29(1)	5(1)	1(1)	0(1)
C(1)	27(1)	25(1)	28(1)	-2(1)	1(1)	-3(1)
C(2)	30(1)	27(1)	27(1)	-2(1)	1(1)	0(1)
C(3)	28(1)	26(1)	29(1)	-1(1)	2(1)	1(1)
C(4)	28(1)	30(1)	27(1)	1(1)	1(1)	1(1)
C(5)	28(1)	26(1)	24(1)	0(1)	2(1)	-3(1)
C(6)	30(1)	35(1)	27(1)	1(1)	-3(1)	3(1)
C(7)	36(1)	34(1)	20(1)	-1(1)	-3(1)	1(1)
C(8)	31(1)	27(1)	23(1)	-1(1)	2(1)	-1(1)
C(9)	28(1)	29(1)	26(1)	2(1)	-1(1)	0(1)
C(10)	30(1)	30(1)	19(1)	1(1)	-1(1)	-3(1)
C(11)	30(1)	26(1)	29(1)	-1(1)	3(1)	-4(1)
C(12)	40(1)	39(1)	41(1)	-1(1)	8(1)	13(1)
C(13)	51(1)	57(1)	21(1)	0(1)	5(1)	16(1)

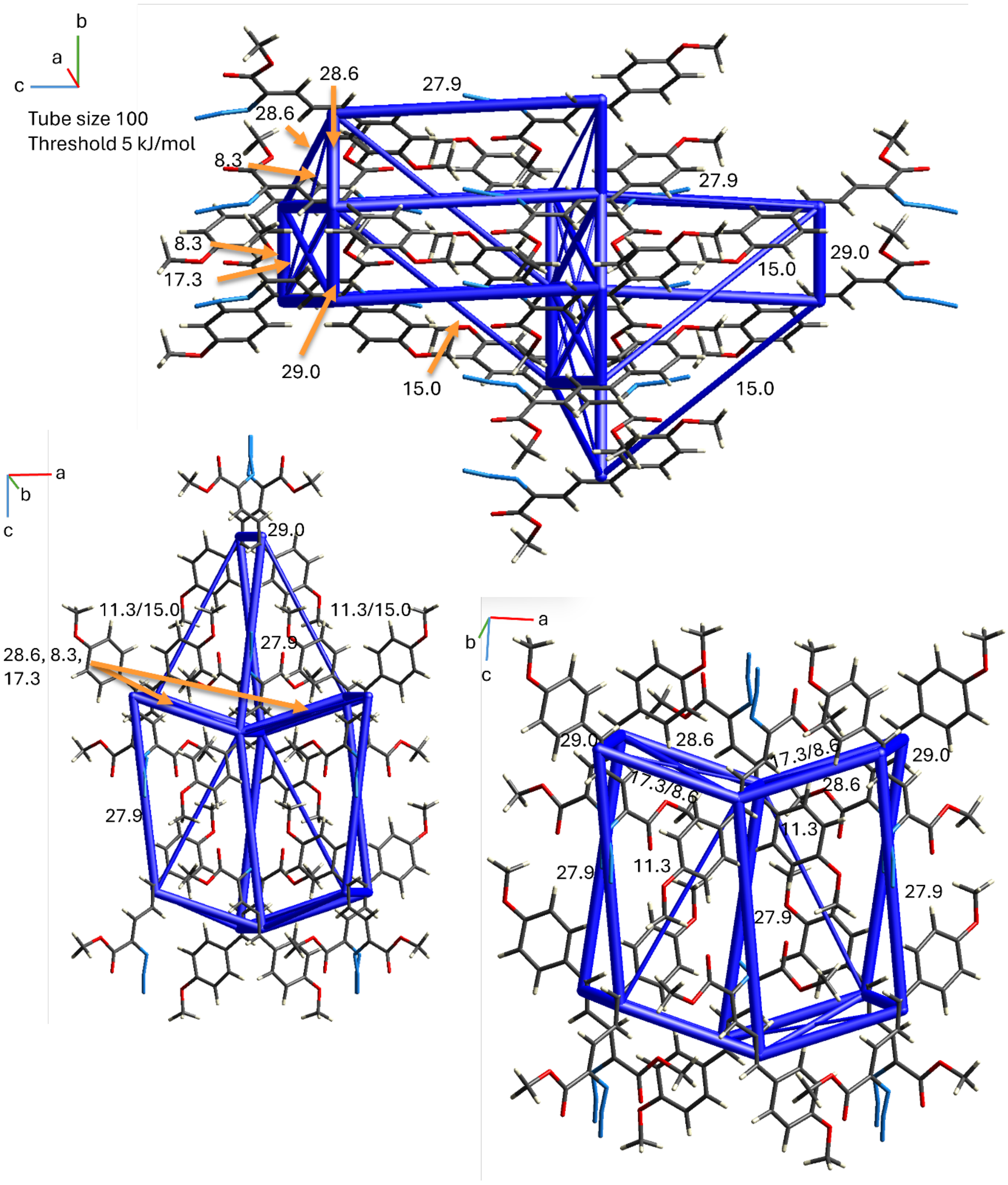
b
a
c
Tube size 100
Threshold 5 kJ/mol
28.6
27.9
28.6
8.3
27.9
8.3
17.3
29.0
15.0
29.0
15.0
15.0
a
b
c
29.0
11.3/15.0
11.3/15.0
27.9
28.6, 8.3,
17.3
27.9
a
b
c
29.0
28.6
17.3/8.6
29.0
17.3/8.6
28.6
11.3
11.3
27.9
27.9
27.9

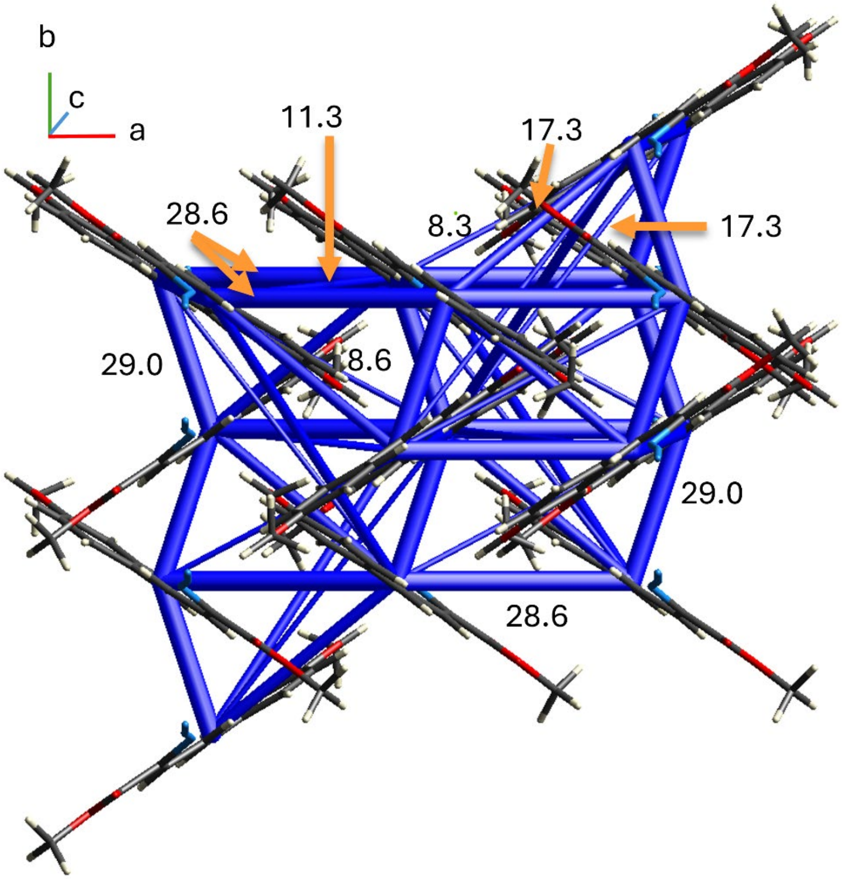

Figure S41. Crystal explorer energy lattice calculations of **1a** (CCDC-2352521) in kJ/mol in B3LYP theory

Interaction Energies (kJ/mol)
R is the distance between molecular centroids (mean atomic position) in Å.

Total energies, only reported for two benchmarked energy models, are the sum of the four energy components, scaled appropriately (see the scale factor table below)

	N	Symop	R	Electron Density	E_ele	E_pol	E_dis	E_rep	E_tot
	2	x+1/2, y, -z+1/2	10.64	B3LYP/6-31G(d,p)	-3.2	-0.5	-8.5	4.6	-8.3
	2	-x, y+1/2, -z+1/2	8.81	B3LYP/6-31G(d,p)	-2.5	-0.8	-10.5	5.9	-8.6
	2	x+1/2, y, -z+1/2	6.54	B3LYP/6-31G(d,p)	-7.6	-1.5	-35.7	19.0	-28.6
	2	-x+1/2, y+1/2, z	4.37	B3LYP/6-31G(d,p)	-7.0	-1.5	-41.6	25.4	-29.0
	2	-x+1/2, -y, z+1/2	12.05	B3LYP/6-31G(d,p)	-10.3	-3.8	-16.3	0.0	-27.9
	2	x, -y+1/2, z+1/2	12.52	B3LYP/6-31G(d,p)	-0.5	-0.5	-3.6	0.0	-4.0
	2	-x, y+1/2, -z+1/2	6.79	B3LYP/6-31G(d,p)	-8.4	-1.0	-17.3	11.9	-17.3
	1	-x, -y, -z	12.41	B3LYP/6-31G(d,p)	-0.2	-0.8	-12.0	0.0	-11.3
	2	x+1/2, -y+1/2, -z	13.46	B3LYP/6-31G(d,p)	0.3	-0.4	-4.1	0.0	-3.6
	1	-x, -y, -z	14.66	B3LYP/6-31G(d,p)	-3.7	-0.9	-12.0	0.0	-15.0

Scale factors for benchmarked energy models
See Mackenzie et al. IUCrJ (2017)

Energy Model	k_ele	k_pol	k_disp	k_rep
CE-HF ... HF/3-21G electron densities	1.019	0.651	0.901	0.811
CE-B3LYP ... B3LYP/6-31G(d,p) electron densities	1.057	0.740	0.871	0.618

Figure S42. Crystal Explorer lattice interaction energies for **1a** (CCDC-2352521) in kJ/mol with B3LYP theory

9.2. X-ray Structure of Azide 1b

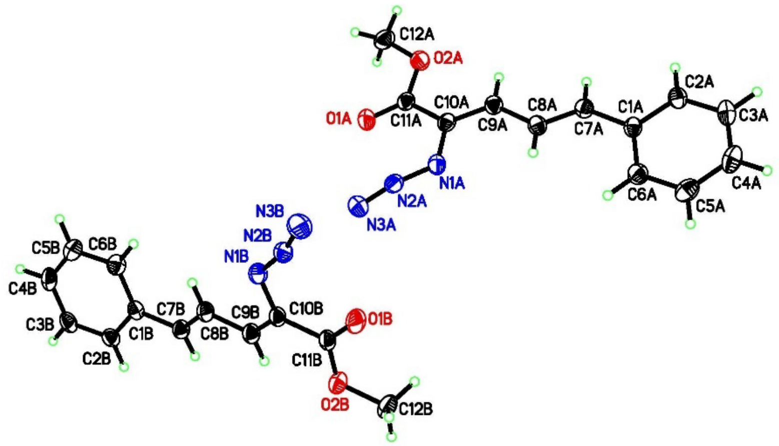

Figure S43. Crystal structure of **1b** (CCDC-2352526) Polymorph I

Crystals were obtained from ethyl acetate-hexane (1:1). For X-ray examination and data collection, a colorless blade-shaped crystal, approximate dimensions 0.332 x 0.092 x 0.066 mm, was mounted in a loop with Paratone-N oil and transferred to the goniostat bathed in a cold stream.

Intensity data were collected at 150K on a Bruker APEX-II CCD diffractometer using Mo Kα radiation, λ=0.71073Å (TRIUMPH curved-graphite monochromator). The data frames were processed using the program SAINT. The data were corrected for decay, Lorentz and polarization effects as well as absorption and beam corrections.

The structure was solved by a combination of direct methods and the difference Fourier technique as implemented in the SHELX suite of programs, and refined by full-matrix least squares on F^2 for reflections out to 0.75Å resolution. Non-hydrogen atoms were refined with anisotropic displacement parameters. The H-atom positions were located directly from the difference map, treated with a riding model. The H-atom isotropic displacement parameters were defined as a*U_{eq} of the adjacent atom (a=1.5 for methyl and 1.2 for all others). The refinement converged with crystallographic agreement factors of R1=4.05%, wR2=8.82% for 3998 reflections with I>2σ(I) (R1=6.97%, wR2=10.00% for all data) and 309 variable parameters.

Table S6. Crystal data and structure refinement for **1b** $C_{13}H_{11}N_3O_2$ (CCDC-2352526)

Empirical formula	$C_{13}H_{11}N_3O_2$	
Formula weight	229.24	
Temperature	150(2) K	
Wavelength	0.71073 Å	
Crystal system	Monoclinic	
Space group	$P2_1/c$	
Unit cell dimensions	a = 7.2493(5) Å	α = 90°
	b = 14.6977(9) Å	β = 91.2626(11)°
	c = 21.3712(13) Å	γ = 90°
Volume	2276.5(3) Å^3	
Z	8	
Density (calculated)	1.338 Mg/m^3	
Absorption coefficient	0.094 mm^{-1}	
F(000)	960	
Crystal size	0.332 x 0.092 x 0.066 mm^3	
θ range for data collection	1.682 to 28.302°	
Index ranges	-9 ≤ h ≤ 9, -19 ≤ k ≤ 19, -28 ≤ l ≤ 28	
Reflections collected	41897	
Independent reflections	5665 [R_{int} = 0.0475]	
Completeness to θ = 25.242°	100.0 %	
Absorption correction	Semi-empirical from equivalents	
Max. and min. transmission	0.862 and 0.820	
Refinement method	Full-matrix least-squares on F^2	
Data / restraints / parameters	5665 / 0 / 309	
Goodness-of-fit on F^2	1.003	
Final R indices [I>2σ(I)]	R1 = 0.0405, wR2 = 0.0882	
R indices (all data)	R1 = 0.0697, wR2 = 0.1000	
Largest diff. peak and hole	0.271 and -0.203 eÅ^{-3}	

Table S7. Atomic coordinates [x10^4] and equivalent isotropic displacement parameters [Å^{2}x10^3] for **1b** $C_{13}H_{11}N_3O_2$ (CCDC-2352526). U(eq) is defined as one third of the trace of the orthogonalized U_{ij} tensor.

	x	y	z	U(eq)
O(1A)	3181(1)	4404(1)	5224(1)	32(1)
O(2A)	2302(1)	5004(1)	6138(1)	31(1)
N(1A)	6604(2)	3852(1)	5800(1)	28(1)
N(2A)	6317(2)	3560(1)	5261(1)	26(1)
N(3A)	6335(2)	3233(1)	4785(1)	36(1)
C(1A)	9487(2)	4917(1)	7941(1)	24(1)
C(2A)	9496(2)	5230(1)	8560(1)	28(1)
C(3A)	11047(2)	5141(1)	8944(1)	33(1)
C(4A)	12635(2)	4751(1)	8716(1)	36(1)
C(5A)	12665(2)	4456(1)	8100(1)	34(1)
C(6A)	11115(2)	4537(1)	7716(1)	30(1)
C(7A)	7780(2)	4996(1)	7564(1)	25(1)
C(8A)	7401(2)	4587(1)	7015(1)	26(1)
C(9A)	5683(2)	4717(1)	6676(1)	25(1)
C(10A)	5268(2)	4384(1)	6101(1)	24(1)
C(11A)	3496(2)	4587(1)	5765(1)	24(1)
C(12A)	546(2)	5237(1)	5847(1)	35(1)
O(1B)	10194(1)	2125(1)	4261(1)	37(1)
O(2B)	11243(1)	2876(1)	3427(1)	30(1)
N(1B)	6848(2)	1725(1)	3619(1)	28(1)
N(2B)	7036(2)	1372(1)	4144(1)	28(1)
N(3B)	6912(2)	988(1)	4598(1)	40(1)
C(1B)	4211(2)	2903(1)	1509(1)	23(1)
C(2B)	4247(2)	3182(1)	883(1)	26(1)
C(3B)	2724(2)	3063(1)	489(1)	31(1)
C(4B)	1128(2)	2674(1)	712(1)	31(1)
C(5B)	1057(2)	2415(1)	1334(1)	31(1)
C(6B)	2573(2)	2529(1)	1732(1)	28(1)
C(7B)	5883(2)	3001(1)	1901(1)	25(1)
C(8B)	6184(2)	2607(1)	2460(1)	26(1)
C(9B)	7880(2)	2705(1)	2819(1)	26(1)
C(10B)	8223(2)	2289(1)	3372(1)	24(1)
C(11B)	9972(2)	2412(1)	3738(1)	25(1)
C(12B)	12954(2)	3026(1)	3778(1)	35(1)
H(2A)	8420	5508	8718	33
H(3A)	11022	5348	9365	39
H(4A)	13698	4686	8979	43
H(5A)	13759	4197	7941	41
H(6A)	11154	4332	7294	36
H(7A)	6846	5377	7725	30
H(8A)	8302	4196	6843	31
H(9A)	4761	5067	6873	30

H(12A)	-254	5512	6158	52
H(12B)	-40	4685	5678	52
H(12C)	740	5671	5506	52
H(2B)	5330	3456	727	32
H(3B)	2775	3248	64	37
H(4B)	89	2586	440	37
H(5B)	-43	2156	1490	37
H(6B)	2502	2352	2158	33
H(7B)	6837	3377	1746	30
H(8B)	5230	2245	2628	31
H(9B)	8814	3085	2655	31
H(12D)	13815	3366	3519	52
H(12E)	13502	2438	3894	52
H(12F)	12701	3374	4158	52

Table S8. Bond lengths [Å] and angles [°] for **1b** $C_{13}H_{11}N_3O_2$ (CCDC-2352526).

O(1A)-C(11A)	1.2040(15)	O(2A)-C(11A)	1.3375(16)
O(2A)-C(12A)	1.4458(16)	N(1A)-N(2A)	1.2427(15)
N(1A)-C(10A)	1.4098(17)	N(2A)-N(3A)	1.1273(15)
C(1A)-C(2A)	1.3993(18)	C(1A)-C(6A)	1.4010(19)
C(1A)-C(7A)	1.4663(18)	C(2A)-C(3A)	1.384(2)
C(3A)-C(4A)	1.385(2)	C(4A)-C(5A)	1.385(2)
C(5A)-C(6A)	1.383(2)	C(7A)-C(8A)	1.3414(18)
C(8A)-C(9A)	1.4397(18)	C(9A)-C(10A)	1.3491(18)
C(10A)-C(11A)	1.4882(18)	O(1B)-C(11B)	1.2016(16)
O(2B)-C(11B)	1.3348(16)	O(2B)-C(12B)	1.4525(16)
N(1B)-N(2B)	1.2422(16)	N(1B)-C(10B)	1.4076(17)
N(2B)-N(3B)	1.1278(16)	C(1B)-C(2B)	1.3994(18)
C(1B)-C(6B)	1.4015(19)	C(1B)-C(7B)	1.4651(18)
C(2B)-C(3B)	1.3852(19)	C(3B)-C(4B)	1.384(2)
C(4B)-C(5B)	1.386(2)	C(5B)-C(6B)	1.3843(19)
C(7B)-C(8B)	1.3415(18)	C(8B)-C(9B)	1.4417(18)
C(9B)-C(10B)	1.3498(18)	C(10B)-C(11B)	1.4859(17)

C(11A)-O(2A)-C(12A)	115.33(11)	N(2A)-N(1A)-C(10A)	120.77(11)
N(3A)-N(2A)-N(1A)	168.66(14)	C(2A)-C(1A)-C(6A)	118.00(12)
C(2A)-C(1A)-C(7A)	118.69(12)	C(6A)-C(1A)-C(7A)	123.32(12)
C(3A)-C(2A)-C(1A)	121.12(13)	C(2A)-C(3A)-C(4A)	120.04(13)
C(3A)-C(4A)-C(5A)	119.65(13)	C(6A)-C(5A)-C(4A)	120.54(14)
C(5A)-C(6A)-C(1A)	120.62(13)	C(8A)-C(7A)-C(1A)	126.86(13)
C(7A)-C(8A)-C(9A)	122.65(13)	C(10A)-C(9A)-C(8A)	125.65(13)
C(9A)-C(10A)-N(1A)	118.32(12)	C(9A)-C(10A)-C(11A)	122.59(12)
N(1A)-C(10A)-C(11A)	119.04(11)	O(1A)-C(11A)-O(2A)	124.18(12)
O(1A)-C(11A)-C(10A)	124.21(12)	O(2A)-C(11A)-C(10A)	111.60(11)
C(11B)-O(2B)-C(12B)	114.31(11)	N(2B)-N(1B)-C(10B)	121.31(11)
N(3B)-N(2B)-N(1B)	167.92(14)	C(2B)-C(1B)-C(6B)	118.23(12)
C(2B)-C(1B)-C(7B)	118.99(12)	C(6B)-C(1B)-C(7B)	122.77(12)
C(3B)-C(2B)-C(1B)	120.87(13)	C(4B)-C(3B)-C(2B)	120.24(13)
C(3B)-C(4B)-C(5B)	119.54(13)	C(6B)-C(5B)-C(4B)	120.65(13)
C(5B)-C(6B)-C(1B)	120.43(13)	C(8B)-C(7B)-C(1B)	125.64(13)
C(7B)-C(8B)-C(9B)	123.41(13)	C(10B)-C(9B)-C(8B)	124.01(13)
C(9B)-C(10B)-N(1B)	118.44(12)	C(9B)-C(10B)-C(11B)	122.99(13)
N(1B)-C(10B)-C(11B)	118.56(11)	O(1B)-C(11B)-O(2B)	124.22(12)
O(1B)-C(11B)-C(10B)	122.79(13)	O(2B)-C(11B)-C(10B)	112.99(11)

Table S9. Anisotropic displacement parameters [$Å^2 x10^3$] for **1b** $C_{13}H_{11}N_3O_2$ (CCDC-2352526). The anisotropic displacement factor exponent takes the form: $-2\pi^2[h^2a^{*2}U_{11}+ ... +2hka^*b^*U_{12}]$.

	U_{11}	U_{22}	U_{33}	U_{23}	U_{13}	U_{12}
O(1A)	29(1)	40(1)	27(1)	-5(1)	-5(1)	0(1)
O(2A)	26(1)	37(1)	29(1)	-3(1)	-1(1)	4(1)
N(1A)	26(1)	32(1)	26(1)	-5(1)	-3(1)	0(1)
N(2A)	22(1)	27(1)	29(1)	-1(1)	1(1)	-2(1)
N(3A)	34(1)	42(1)	31(1)	-10(1)	2(1)	0(1)
C(1A)	26(1)	21(1)	25(1)	-1(1)	-2(1)	-2(1)
C(2A)	31(1)	26(1)	26(1)	-3(1)	-2(1)	0(1)
C(3A)	40(1)	31(1)	27(1)	-2(1)	-9(1)	-2(1)
C(4A)	32(1)	31(1)	44(1)	5(1)	-15(1)	-2(1)
C(5A)	26(1)	30(1)	46(1)	1(1)	-1(1)	3(1)
C(6A)	30(1)	29(1)	30(1)	-3(1)	1(1)	1(1)
C(7A)	26(1)	24(1)	26(1)	-2(1)	0(1)	1(1)
C(8A)	28(1)	24(1)	24(1)	0(1)	0(1)	0(1)
C(9A)	26(1)	24(1)	25(1)	-1(1)	0(1)	-2(1)
C(10A)	24(1)	22(1)	26(1)	0(1)	0(1)	-4(1)
C(11A)	24(1)	24(1)	26(1)	1(1)	0(1)	-5(1)
C(12A)	23(1)	42(1)	39(1)	2(1)	-1(1)	2(1)
O(1B)	30(1)	56(1)	25(1)	6(1)	-10(1)	-2(1)
O(2B)	25(1)	34(1)	30(1)	0(1)	-7(1)	-2(1)
N(1B)	25(1)	38(1)	20(1)	2(1)	-5(1)	1(1)
N(2B)	23(1)	37(1)	24(1)	-2(1)	-3(1)	3(1)
N(3B)	41(1)	54(1)	26(1)	8(1)	-1(1)	-2(1)
C(1B)	26(1)	22(1)	21(1)	-1(1)	-4(1)	5(1)
C(2B)	28(1)	30(1)	21(1)	-1(1)	0(1)	3(1)
C(3B)	36(1)	37(1)	18(1)	-3(1)	-5(1)	6(1)
C(4B)	32(1)	32(1)	30(1)	-6(1)	-12(1)	2(1)
C(5B)	28(1)	29(1)	35(1)	1(1)	-4(1)	-2(1)
C(6B)	31(1)	29(1)	23(1)	4(1)	-4(1)	0(1)
C(7B)	24(1)	26(1)	24(1)	-1(1)	-3(1)	2(1)
C(8B)	27(1)	27(1)	23(1)	0(1)	-4(1)	1(1)
C(9B)	26(1)	28(1)	24(1)	-2(1)	-4(1)	2(1)
C(10B)	22(1)	27(1)	22(1)	-4(1)	-4(1)	4(1)
C(11B)	24(1)	27(1)	23(1)	-5(1)	-5(1)	5(1)
C(12B)	23(1)	38(1)	42(1)	-4(1)	-9(1)	-1(1)

Table S10. Torsion angles [°] for **1b** $C_{13}H_{11}N_3O_2$ (CCDC-2352526).

C(6A)-C(1A)-C(2A)-C(3A)	-2.0(2)	C(7A)-C(1A)-C(2A)-C(3A)	177.59(13)
C(1A)-C(2A)-C(3A)-C(4A)	1.1(2)	C(2A)-C(3A)-C(4A)-C(5A)	0.4(2)
C(3A)-C(4A)-C(5A)-C(6A)	-0.9(2)	C(4A)-C(5A)-C(6A)-C(1A)	-0.1(2)
C(2A)-C(1A)-C(6A)-C(5A)	1.5(2)	C(7A)-C(1A)-C(6A)-C(5A)	-178.08(13)
C(2A)-C(1A)-C(7A)-C(8A)	-166.36(13)	C(6A)-C(1A)-C(7A)-C(8A)	13.2(2)
C(1A)-C(7A)-C(8A)-C(9A)	-179.33(13)	C(7A)-C(8A)-C(9A)-C(10A)	174.62(13)
C(8A)-C(9A)-C(10A)-N(1A)	0.7(2)	C(8A)-C(9A)-C(10A)-C(11A)	-176.70(12)
N(2A)-N(1A)-C(10A)-C(9A)	-177.14(12)	N(2A)-N(1A)-C(10A)-C(11A)	0.35(18)
C(12A)-O(2A)-C(11A)-O(1A)	-0.12(19)	C(12A)-O(2A)-C(11A)-C(10A)	179.36(11)
C(9A)-C(10A)-C(11A)-O(1A)	169.66(13)	N(1A)-C(10A)-C(11A)-O(1A)	-7.7(2)
C(9A)-C(10A)-C(11A)-O(2A)	-9.81(18)	N(1A)-C(10A)-C(11A)-O(2A)	172.81(11)
C(6B)-C(1B)-C(2B)-C(3B)	-2.09(19)	C(7B)-C(1B)-C(2B)-C(3B)	177.02(12)
C(1B)-C(2B)-C(3B)-C(4B)	0.8(2)	C(2B)-C(3B)-C(4B)-C(5B)	0.8(2)
C(3B)-C(4B)-C(5B)-C(6B)	-0.9(2)	C(4B)-C(5B)-C(6B)-C(1B)	-0.5(2)
C(2B)-C(1B)-C(6B)-C(5B)	1.9(2)	C(7B)-C(1B)-C(6B)-C(5B)	-177.14(13)
C(2B)-C(1B)-C(7B)-C(8B)	-165.76(13)	C(6B)-C(1B)-C(7B)-C(8B)	13.3(2)
C(1B)-C(7B)-C(8B)-C(9B)	178.08(12)	C(7B)-C(8B)-C(9B)-C(10B)	-177.84(13)
C(8B)-C(9B)-C(10B)-N(1B)	-0.1(2)	C(8B)-C(9B)-C(10B)-C(11B)	-179.02(12)
N(2B)-N(1B)-C(10B)-C(9B)	-175.32(13)	N(2B)-N(1B)-C(10B)-C(11B)	3.67(19)
C(12B)-O(2B)-C(11B)-O(1B)	-0.84(19)	C(12B)-O(2B)-C(11B)-C(10B)	178.63(11)
C(9B)-C(10B)-C(11B)-O(1B)	171.39(14)	N(1B)-C(10B)-C(11B)-O(1B)	-7.5(2)
C(9B)-C(10B)-C(11B)-O(2B)	-8.09(18)	N(1B)-C(10B)-C(11B)-O(2B)	172.97(11)

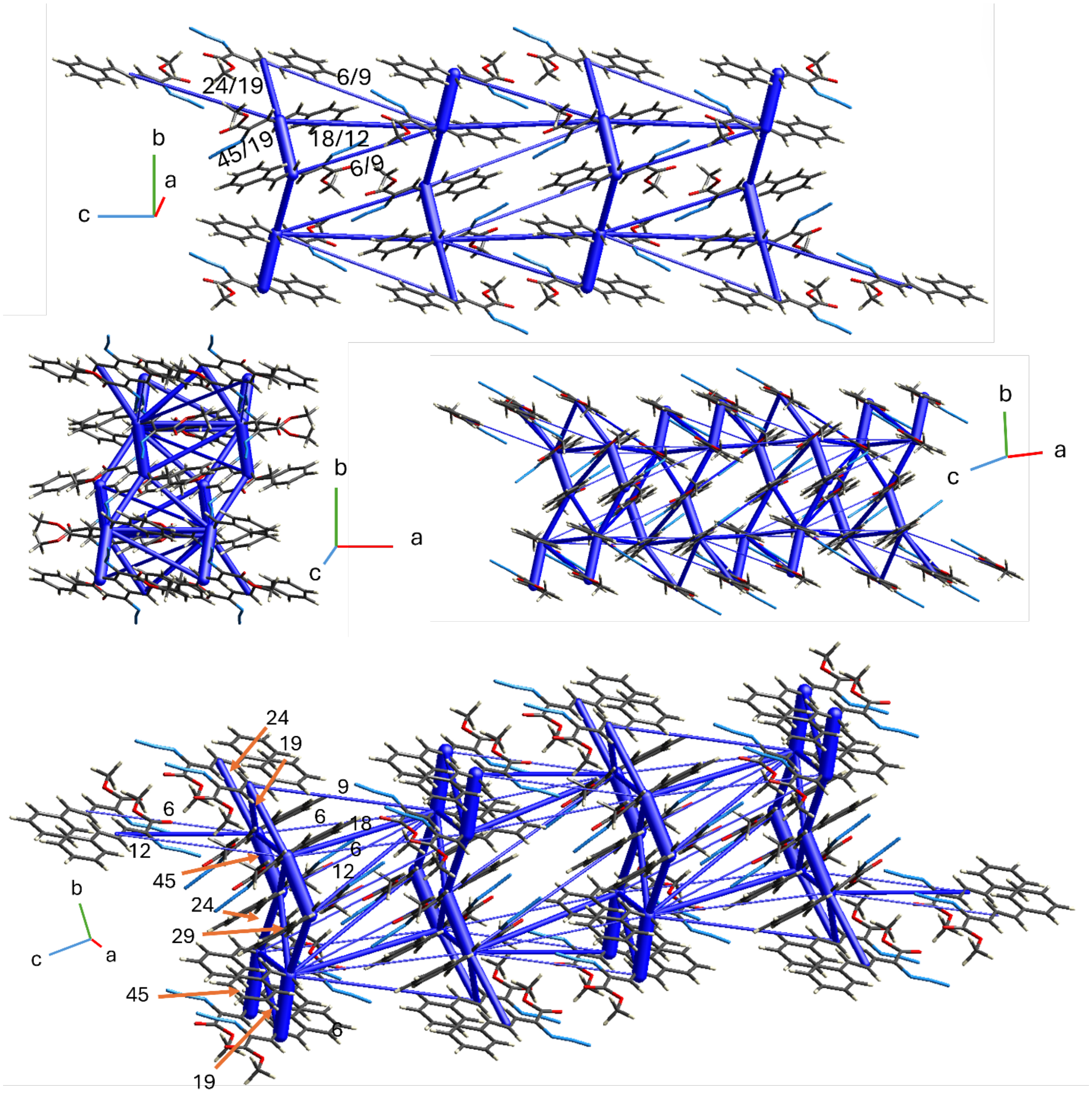

Figure S44. Crystal explorer energy lattice calculations of **1b** $C_{13}H_{11}N_3O_2$ (CCDC-2352526) in kJ/mol in B3LYP theory.

Interaction Energies (kJ/mol)
R is the distance between molecular centroids (mean atomic position) in Å.

Total energies, only reported for two benchmarked energy models, are the sum of the four energy components, scaled appropriately (see the scale factor table below)

	N	Symop	R	Electron Density	E_ele	E_pol	E_dis	E_rep	E_tot
	1	-	3.61	B3LYP/6-31G(d,p)	-6.7	-1.7	-63.5	31.0	-44.5
	1	-	9.32	B3LYP/6-31G(d,p)	-1.1	-0.8	-7.6	4.0	-5.9
	0	-	5.29	B3LYP/6-31G(d,p)	-5.6	-1.5	-37.8	25.1	-24.4
	1	-	7.82	B3LYP/6-31G(d,p)	-5.6	-1.0	-26.1	17.4	-18.7
	1	-	14.65	B3LYP/6-31G(d,p)	-1.1	-0.1	-2.8	0.0	-3.7
	0	-	6.52	B3LYP/6-31G(d,p)	-3.4	-1.1	-26.6	13.3	-19.4
	0	x, y, z	7.29	B3LYP/6-31G(d,p)	-4.2	-0.9	-21.8	10.8	-17.5
	0	x, -y+1/2, z+1/2	12.79	B3LYP/6-31G(d,p)	-6.0	-2.6	-11.2	0.0	-18.0
	0	x, -y+1/2, z+1/2	10.74	B3LYP/6-31G(d,p)	-2.9	-0.5	-4.7	2.8	-5.8
	0	-	13.33	B3LYP/6-31G(d,p)	-5.2	-0.7	-3.7	0.0	-9.3
	0	-	11.84	B3LYP/6-31G(d,p)	-7.8	-1.4	-7.2	4.9	-12.4
	0	-	14.27	B3LYP/6-31G(d,p)	-0.2	-0.1	-1.3	0.0	-1.3

Figure S45. Crystal Explorer lattice interaction energies for **1b** (CCDC- 2352526) in kJ/mol with B3LYP theory

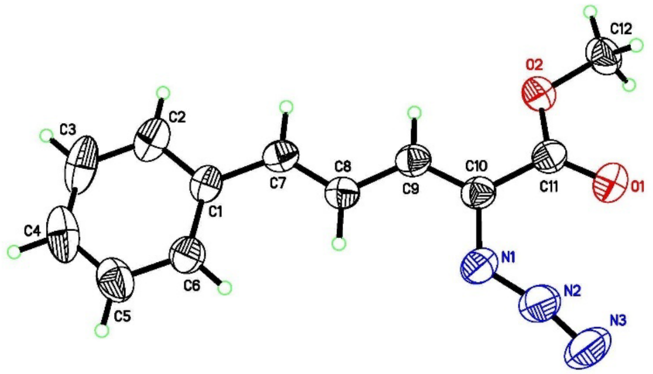

Figure S46. Crystal structure of **1b** (CCDC-2352525) Polymorph II

Crystals were obtained from ethyl acetate-hexane (1:1). For X-ray examination and data collection, a colorless blade-shaped crystal, approximate dimensions 0.332 x 0.092 x 0.066 mm, was mounted in a loop with Paratone-N oil and transferred to the goniostat bathed in a cold stream. Intensity data were collected at 150K on a Bruker APEX-II CCD diffractometer using Mo Kα radiation, λ=0.71073Å (TRIUMPH curved-graphite monochromator). The data frames were processed using the program SAINT. The data were corrected for decay, Lorentz and polarization effects as well as absorption and beam corrections. The structure was solved by a combination of direct methods and the difference Fourier technique as implemented in the SHELX suite of programs, and refined by full-matrix least squares on F2 for reflections out to 0.75Å resolution. Non-hydrogen atoms were refined with anisotropic displacement parameters. The H-atom positions were located directly from the difference map, treated with a riding model. The H-atom isotropic displacement parameters were defined as a*Ueq of the adjacent atom (a=1.5 for methyl and 1.2 for all others). The refinement converged with crystallographic agreement factors of R1=4.05%, wR2=8.82% for 3998 reflections with I>2σ(I) (R1=6.97%, wR2=10.00% for all data) and 309 variable parameters (18075c).

Table S11. Crystal data and structure refinement for **1b** $C_{12}H_{11}N_3O_2$ (CCDC-2352525).

Empirical formula	$C_{12}H_{11}N_3O_2$	
Formula weight	229.24	
Temperature	150(2) K	
Wavelength	0.71073 Å	
Crystal system	Monoclinic	
Space group	$P2_1/c$	
Unit cell dimensions	a = 7.9098(3) Å	α = 90°
	b = 13.1978(5) Å	β = 119.9924(14)°
	c = 13.0071(6) Å	γ = 90°
Volume	1176.01(8) $Å^3$	
Z	4	
Density (calculated)	1.295 Mg/m^3	
Absorption coefficient	0.091 mm^{-1}	
F(000)	480	
Crystal size	0.118 x 0.098 x 0.081 mm^3	
θ range for data collection	2.973 to 26.362°	
Index ranges	-9 ≤ h ≤ 9, -16 ≤ k ≤ 16, -14 ≤ l ≤ 16	
Reflections collected	11637	
Independent reflections	2411 [Rint = 0.0426]	
Completeness to θ = 25.242°	100.0 %	
Absorption correction	Numerical	
Max. and min. transmission	0.862 and 0.719	
Refinement method	Full-matrix least-squares on F^2	
Data / restraints / parameters	2411 / 0 / 187	
Goodness-of-fit on F^2	1.045	
Final R indices [I>2σ(I)]	R1 = 0.0434, wR2 = 0.0992	
R indices (all data)	R1 = 0.0644, wR2 = 0.1132	
Largest diff. peak and hole	0.159 and -0.175 $eÅ^{-3}$	

Table S12. Atomic coordinates [x10[4]] and equivalent isotropic displacement parameters [$Å^2$x10^3] for **1b** $C_{12}H_{11}N_3O_2$. U(eq) is (CCDC-2352525)defined as one third of the trace of the orthogonalized Uij tensor.

	x	y	z	U(eq)
O(1)	8968(2)	3026(1)	8497(1)	58(1)
O(2)	7006(2)	2518(1)	6624(1)	45(1)
N(1)	9877(2)	4823(1)	7678(1)	52(1)
N(2)	10950(2)	4656(1)	8757(1)	53(1)
N(3)	12040(3)	4667(1)	9738(2)	72(1)
C(1)	6875(2)	6191(1)	3354(1)	38(1)
C(2)	5682(3)	6207(1)	2120(2)	52(1)
C(3)	5855(4)	6992(2)	1463(2)	70(1)
C(4)	7166(4)	7760(2)	2021(2)	67(1)
C(5)	8311(3)	7762(2)	3232(2)	58(1)
C(6)	8176(3)	6989(1)	3896(2)	45(1)
C(7)	6705(2)	5340(1)	4017(1)	38(1)
C(8)	7829(2)	5155(1)	5180(1)	37(1)
C(9)	7561(2)	4284(1)	5750(2)	40(1)
C(10)	8535(2)	4094(1)	6920(1)	38(1)
C(11)	8218(2)	3168(1)	7446(1)	41(1)
C(12)	6576(3)	1611(1)	7076(2)	52(1)
H(2)	4770(30)	5667(16)	1765(17)	62
H(3)	5100(30)	6978(17)	690(20)	84
H(4)	7300(30)	8327(17)	1584(19)	80
H(5)	9210(30)	8315(16)	3617(18)	70
H(6)	9010(30)	6989(13)	4762(16)	54
H(7)	5680(30)	4851(13)	3569(14)	46
H(8)	8870(30)	5609(13)	5661(14)	45
H(9)	6570(30)	3808(13)	5254(14)	47
H(12A)	7770(30)	1206(17)	7522(18)	78
H(12B)	5650(30)	1228(17)	6360(20)	78
H(12C)	6160(30)	1808(17)	7613(19)	78

Table S13. Bond lengths [Å] and angles [°] for **1b** $C_{12}H_{11}N_3O_2$ (CCDC-2352525).

O(1)-C(11)	1.2021(18)	O(2)-C(11)	1.3316(19)	O(2)-
C(12)	1.448(2)	N(1)-N(2)	1.2436(19)	N(1)-
C(10)	1.406(2)	N(2)-N(3)	1.1277(19)	C(1)-
C(6)	1.393(2)	C(1)-C(2)	1.397(2)	C(1)-
C(7)	1.463(2)	C(2)-C(3)	1.392(3)	C(3)-
C(4)	1.370(3)	C(4)-C(5)	1.370(3)	C(5)-
C(6)	1.374(2)	C(7)-C(8)	1.339(2)	C(8)-
C(9)	1.439(2)	C(9)-C(10)	1.343(2)	C(10)-
C(11)	1.482(2)			

C(11)-O(2)-C(12)	115.27(14)	N(2)-N(1)-C(10)	121.36(15)	N(3)-
N(2)-N(1)	168.18(18)	C(6)-C(1)-C(2)	118.30(16)	C(6)-
C(1)-C(7)	122.97(14)	C(2)-C(1)-C(7)	118.74(15)	C(3)-
C(2)-C(1)	119.96(19)	C(4)-C(3)-C(2)	120.39(19)	C(5)-
C(4)-C(3)	120.01(19)	C(4)-C(5)-C(6)	120.5(2)	C(5)-
C(6)-C(1)	120.84(18)	C(8)-C(7)-C(1)	126.96(15)	C(7)-
C(8)-C(9)	122.78(15)	C(10)-C(9)-C(8)	125.79(15)	C(9)-
C(10)-N(1)	118.11(15)	C(9)-C(10)-C(11)	123.01(15)	N(1)-
C(10)-C(11)	118.86(14)	O(1)-C(11)-O(2)	124.15(15)	O(1)-
C(11)-C(10)	123.54(15)	O(2)-C(11)-C(10)	112.31(13)	

Table S14. Anisotropic displacement parameters [$Å^2x10^3$] for **1b** $C_{12}H_{11}N_3O_2$ (CCDC-2352525). The anisotropic displacement factor exponent takes the form: $-2\pi^2[h^2a^{*2}U_{11}+... +2hka^*b^*U_{12}]$

	U_{11}	U_{22}	U_{33}	U_{23}	U_{13}	U_{12}
O(1)	54(1)	64(1)	42(1)	12(1)	14(1)	-3(1)
O(2)	46(1)	41(1)	47(1)	4(1)	22(1)	-4(1)
N(1)	48(1)	50(1)	40(1)	3(1)	10(1)	-4(1)
N(2)	48(1)	49(1)	48(1)	-2(1)	15(1)	-2(1)
N(3)	70(1)	70(1)	44(1)	-5(1)	4(1)	-7(1)
C(1)	41(1)	37(1)	37(1)	2(1)	21(1)	11(1)
C(2)	64(1)	45(1)	38(1)	-3(1)	19(1)	16(1)
C(3)	104(2)	67(1)	42(1)	16(1)	38(1)	41(1)
C(4)	90(2)	55(1)	82(2)	28(1)	62(1)	29(1)
C(5)	55(1)	49(1)	79(2)	16(1)	39(1)	9(1)
C(6)	43(1)	43(1)	48(1)	3(1)	21(1)	4(1)
C(7)	36(1)	37(1)	39(1)	-7(1)	17(1)	1(1)
C(8)	35(1)	36(1)	41(1)	-2(1)	18(1)	1(1)
C(9)	34(1)	40(1)	42(1)	-1(1)	17(1)	1(1)
C(10)	33(1)	38(1)	41(1)	1(1)	16(1)	2(1)
C(11)	32(1)	44(1)	42(1)	4(1)	16(1)	4(1)
C(12)	56(1)	39(1)	61(1)	10(1)	30(1)	-1(1)

Table S15. Torsion angles [°] for **1b** $C_{12}H_{11}N_3O_2$ (CCDC-2352525).

C(10)-N(1)-N(2)-N(3)	179.8(9)	C(6)-C(1)-C(2)-C(3)	1.9(3)	C(7)-
C(1)-C(2)-C(3)	-178.05(16)	C(1)-C(2)-C(3)-C(4)	-1.1(3)	C(2)-
C(3)-C(4)-C(5)	-0.3(3)	C(3)-C(4)-C(5)-C(6)	1.0(3)	C(4)-
C(5)-C(6)-C(1)	-0.1(3)	C(2)-C(1)-C(6)-C(5)	-1.3(2)	C(7)-
C(1)-C(6)-C(5)	178.65(16)	C(6)-C(1)-C(7)-C(8)	-5.9(3)	C(2)-
C(1)-C(7)-C(8)	174.03(16)	C(1)-C(7)-C(8)-C(9)	-179.06(15)	C(7)-
C(8)-C(9)-C(10)	-174.68(16)	C(8)-C(9)-C(10)-N(1)	3.2(2)	C(8)-
C(9)-C(10)-C(11)	-178.55(15)	N(2)-N(1)-C(10)-C(9)	-173.57(16)	N(2)-
N(1)-C(10)-C(11)	8.1(2)	C(12)-O(2)-C(11)-O(1)	1.8(2)	C(12)-
O(2)-C(11)-C(10)	-177.90(14)	C(9)-C(10)-C(11)-O(1)	-174.02(15)	N(1)-
C(10)-C(11)-O(1)	4.3(2)	C(9)-C(10)-C(11)-O(2)	5.7(2)	N(1)-
C(10)-C(11)-O(2)	-176.04(13)			

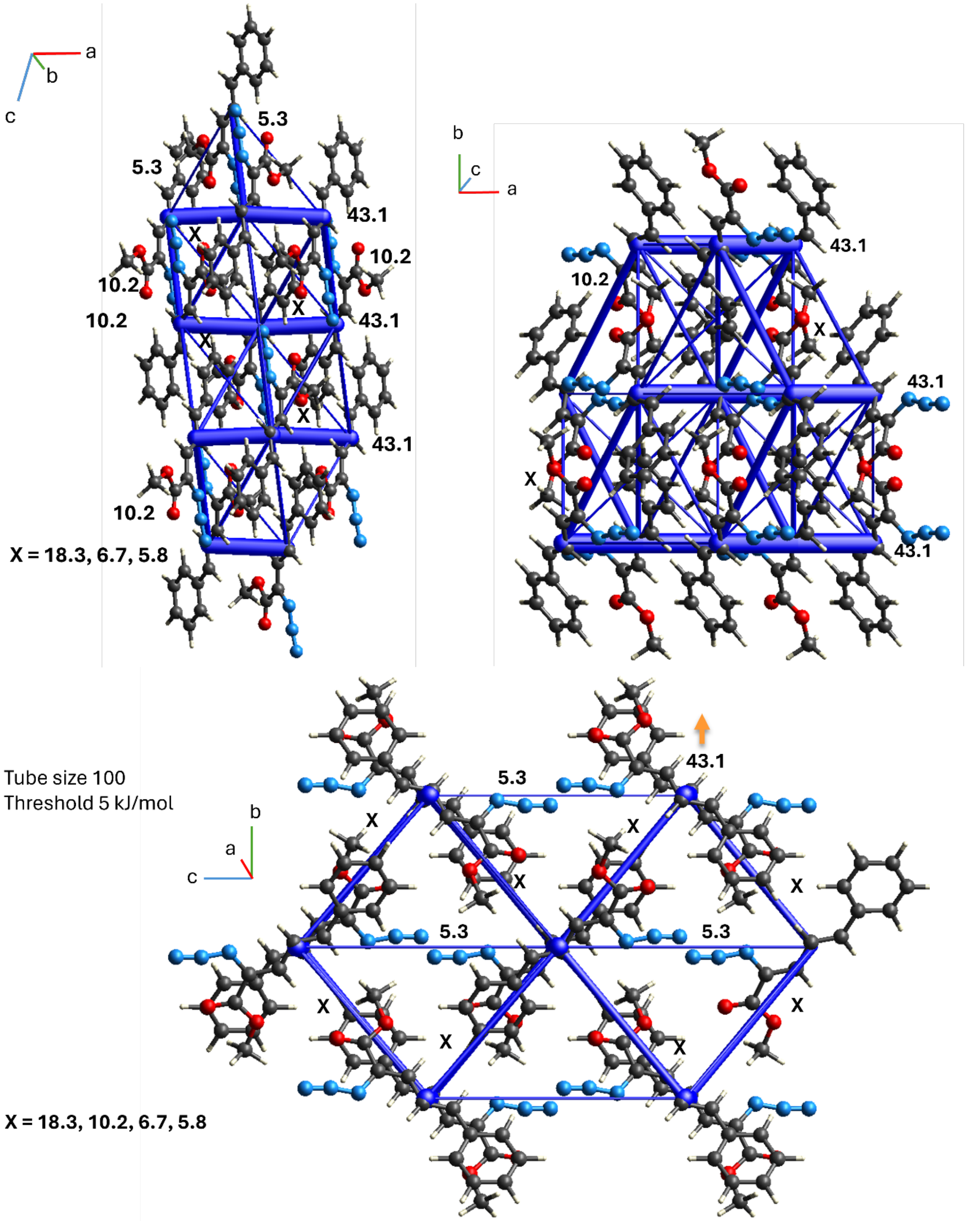

Figure S47. Crystal explorer energy lattice calculations of **1b** (CCDC- 2352525) in kJ/mol in B3LYP theory

----

Interaction Energies (kJ/mol)
R is the distance between molecular centroids (mean atomic position) in Å.

Total energies, only reported for two benchmarked energy models, are the sum of the four energy components, scaled appropriately (see the scale factor table below)

	N	Symop	R	Electron Density	E_ele	E_pol	E_dis	E_rep	E_tot
	2	-x, y+1/2, -z+1/2	7.87	B3LYP/6-31G(d,p)	-10.7	-2.7	-17.9	17.3	-18.3
	2	x, -y+1/2, z+1/2	9.13	B3LYP/6-31G(d,p)	0.6	-1.0	-11.7	5.8	-6.7
	2	-x, y+1/2, -z+1/2	9.67	B3LYP/6-31G(d,p)	-3.3	-0.5	-14.3	9.8	-10.2
	2	x, y, z	11.35	B3LYP/6-31G(d,p)	-3.5	-0.6	-4.1	3.9	-5.3
	2	x, -y+1/2, z+1/2	9.40	B3LYP/6-31G(d,p)	-3.5	-0.5	-15.4	9.3	-11.7
	1	-x, -y, -z	4.25	B3LYP/6-31G(d,p)	-13.3	-2.2	-56.9	35.9	-43.1
	1	-x, -y, -z	4.15	B3LYP/6-31G(d,p)	-7.9	-1.2	-61.2	31.4	-43.2
	2	x, -y+1/2, z+1/2	9.72	B3LYP/6-31G(d,p)	-3.9	-0.6	-3.4	2.7	-5.8
	1	-x, -y, -z	10.22	B3LYP/6-31G(d,p)	1.2	-0.5	-4.3	0.5	-2.6

Scale factors for benchmarked energy models
See Mackenzie et al. IUCrJ (2017)

Energy Model	k_ele	k_pol	k_disp	k_rep
CE-HF ... HF/3-21G electron densities	1.019	0.651	0.901	0.811
CE-B3LYP ... B3LYP/6-31G(d,p) electron densities	1.057	0.740	0.871	0.618

Figure S48. Crystal Explorer lattice interaction energies for **1b** (CCDC- 2352525) in kJ/mol with B3LYP theory

Additional structures were collected with almost exact structural information.

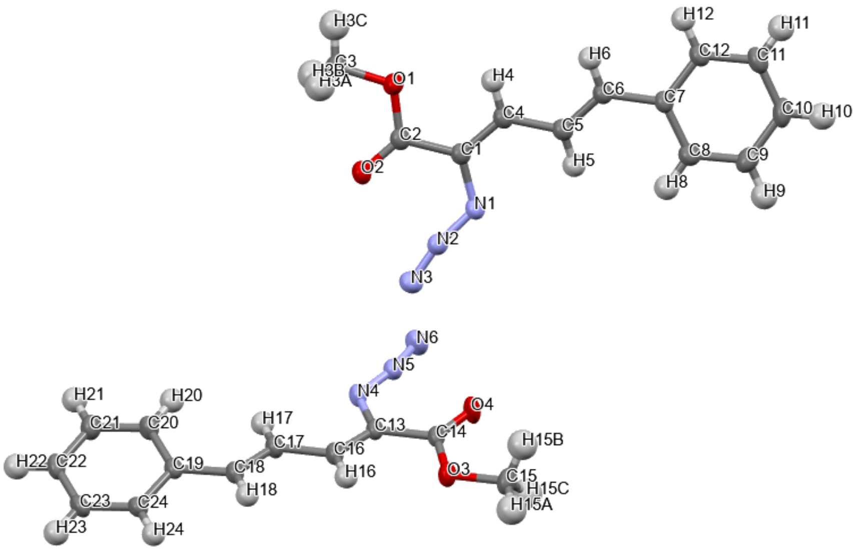

Figure S49. Crystal structure of **1b** (Sjkc23f18) Polymorph 1

Single crystals of $C_{24}H_{22}N_6O_4$ [**1b** (Sjkc23f20)] were [Grown in hexanes:ethylacetate 1:1]. A suitable crystal was selected and [Paratone oil on loop.] on a XtaLAB Synergy, Dualflex, Pilatus 300K diffractometer. The crystal was kept at 150.00(10) K during data collection. Using Olex2,[8] the structure was solved with the SHELXT[9] structure solution program using Intrinsic Phasing and refined with the SHELXL [10]refinement package using Least Squares minimisation.

Crystal structure determination of **1b** (Sjkc23f18)**: Crystal Data** for $C_{24}H_{22}N_6O_4$ (M =458.47 g/mol): monoclinic, space group P2$_1$/c (no. 14), a = 7.2429(4) Å, b = 14.6919(8) Å, c = 21.3492(11) Å, β = 91.260(5)°, V = 2271.3(2) Å^3, Z = 4, T = 150.00(10) K, μ(Mo Kα) = 0.095 mm^{-1}, $Dcalc$ = 1.341 g/cm^3, 41278 reflections measured (4.718° ≤ 2Θ ≤ 67.442°), 7703 unique (R_{int} = 0.0656, R_{sigma} = 0.0576) which were used in all calculations. The final R_1 was 0.0524 (I > 2σ(I)) and wR_2 was 0.1292 (all data) (Sjkc23f20).

Table S16. Crystal data and structure refinement for **1b** (Sjkc23f18)

Identification code	**1b** (Sjkc23f20)
Empirical formula	$C_{24}H_{22}N_6O_4$
Formula weight	458.47
Temperature/K	150.00(10)
Crystal system	monoclinic
Space group	$P2_1/c$
a/Å	7.2429(4)
b/Å	14.6919(8)
c/Å	21.3492(11)
α/°	90
β/°	91.260(5)
γ/°	90
Volume/Å^3	2271.3(2)
Z	8
ρ_{calc}g/cm^3	1.341
μ/mm^{-1}	0.095
F(000)	960.0
Crystal size/mm^3	0.672 × 0.174 × 0.1
Radiation	Mo Kα (λ = 0.71073)
2Θ range for data collection/°	4.718 to 67.442
Index ranges	-11 ≤ h ≤ 10, -21 ≤ k ≤ 20, -31 ≤ l ≤ 29
Reflections collected	41278
Independent reflections	7703 [R_{int} = 0.0656, R_{sigma} = 0.0576]
Data/restraints/parameters	7703/0/310
Goodness-of-fit on F^2	1.046
Final R indexes [I>=2σ (I)]	R_1 = 0.0524, wR_2 = 0.1161
Final R indexes [all data]	R_1 = 0.0951, wR_2 = 0.1292
Largest diff. peak/hole / e Å^{-3}	0.36/-0.22

Table S17. Fractional Atomic Coordinates (×10[4]) and Equivalent Isotropic Displacement Parameters (Å^2×10^3) for **1b** (Sjkc23f18). Ueq is defined as 1/3 of the trace of the orthogonalised UIJ tensor.

**Atom**	***x***	***y***	***z***	**U(eq)**
O1	2302.8(12)	4995.6(6)	6137.4(4)	32.4(2)
O2	3177.6(12)	5596.6(7)	5223.3(4)	33.1(2)
O3	11243.3(12)	7125.3(6)	3426.6(4)	31.1(2)
O4	10196.3(13)	7875.0(7)	4261.4(4)	38.7(2)
N2	6314.7(14)	6440.7(7)	5260.7(5)	27.8(2)
N4	6846.8(14)	8275.2(8)	3618.6(5)	29.5(2)
N5	7036.9(14)	8629.3(8)	4143.9(5)	29.4(2)
N1	6604.5(14)	6149.4(7)	5800.0(5)	29.3(2)
N3	6336.4(16)	6766.0(8)	4783.7(5)	37.0(3)
N6	6913.6(17)	9012.6(9)	4597.6(5)	40.6(3)
C19	4214.7(17)	7097.0(8)	1509.1(5)	24.7(2)
C13	8221.4(16)	7711.8(8)	3372.0(5)	24.9(2)
C2	3499.0(17)	5413.1(8)	5766.8(5)	26.0(2)
C14	9971.1(16)	7588.3(8)	3737.2(5)	26.4(2)
C1	5270.3(16)	5615.4(8)	6100.4(5)	25.3(2)
C7	9485.4(17)	5083.0(8)	7941.1(5)	25.0(2)
C24	4247.5(17)	6818.4(9)	881.0(5)	27.5(3)
C17	6188.6(17)	7395.3(9)	2458.5(5)	27.2(3)
C5	7401.1(17)	5413.0(8)	7014.7(5)	27.1(3)
C4	5686.3(17)	5283.6(8)	6676.2(5)	26.7(3)
C18	5881.1(17)	6997.4(9)	1899.2(5)	26.6(2)
C16	7882.3(17)	7295.7(8)	2817.0(5)	26.9(2)
C12	9496.0(18)	4769.5(8)	8560.2(6)	28.9(3)
C6	7786.2(17)	5003.7(8)	7564.1(5)	26.6(2)
C20	2572.4(17)	7473.2(9)	1730.9(6)	29.3(3)
C23	2722.2(19)	6934.3(9)	487.7(6)	32.0(3)
C21	1056.7(18)	7586.2(9)	1334.7(6)	32.1(3)
C8	11121.6(18)	5464.1(9)	7718.2(6)	31.0(3)
C22	1125.6(18)	7326.4(9)	711.0(6)	32.8(3)
C9	12665.9(19)	5545.1(9)	8101.4(7)	35.2(3)
C11	11046(2)	4855.8(9)	8944.8(6)	34.7(3)
C10	12634.6(19)	5248.0(9)	8718.4(7)	35.9(3)
C3	545.6(18)	4762.9(10)	5846.2(7)	36.4(3)
C15	12950.9(18)	6973.3(10)	3778.2(7)	36.0(3)

Table S18. Anisotropic Displacement Parameters (Å^2×10^3) for **1b** (Sjkc23f18). The Anisotropic displacement factor exponent takes the form: -2π2[h2a*2U11+2hka*b*U12+…].

Atom	U_{11}	U_{22}	U_{33}	U_{23}	U_{13}	U_{12}
O1	26.5(5)	40.8(5)	29.7(5)	3.1(4)	-2.0(4)	-5.0(4)
O2	29.0(5)	42.7(6)	27.4(4)	4.8(4)	-5.7(4)	0.3(4)
O3	25.6(4)	37.0(5)	30.2(4)	-0.1(4)	-7.5(4)	2.0(4)
O4	31.5(5)	58.6(6)	25.6(4)	-5.7(4)	-10.8(4)	2.9(4)
N2	24.2(5)	30.6(6)	28.7(5)	2.0(4)	0.4(4)	2.9(4)
N4	26.4(5)	39.9(6)	21.8(5)	-2.0(4)	-5.7(4)	-0.5(4)
N5	25.3(5)	38.7(6)	24.1(5)	2.5(4)	-2.4(4)	-1.8(4)
N1	27.1(5)	34.2(6)	26.6(5)	5.7(4)	-3.7(4)	0.2(4)
N3	34.5(6)	45.0(7)	31.4(6)	9.6(5)	1.2(5)	-0.4(5)
N6	39.1(7)	56.1(8)	26.6(5)	-7.4(5)	-1.3(5)	1.4(6)
C19	27.6(6)	24.2(6)	22.2(5)	1.3(4)	-3.7(5)	-4.1(4)
C13	22.9(6)	28.3(6)	23.4(5)	3.2(4)	-4.9(5)	-2.6(4)
C2	26.3(6)	25.6(6)	26.2(6)	-0.6(4)	-0.9(5)	3.9(5)
C14	23.8(6)	30.8(6)	24.3(6)	5.3(5)	-5.6(5)	-4.6(5)
C1	25.3(6)	25.1(6)	25.4(6)	0.4(4)	-0.9(5)	2.8(4)
C7	27.2(6)	23.0(6)	24.7(5)	0.8(4)	-3.3(5)	1.1(4)
C24	29.3(6)	32.1(7)	21.2(5)	1.1(5)	-0.5(5)	-3.2(5)
C17	27.3(6)	30.9(6)	23.3(5)	-0.6(5)	-4.6(5)	-0.7(5)
C5	29.3(6)	26.6(6)	25.4(6)	0.8(5)	-0.7(5)	1.0(5)
C4	28.0(6)	26.6(6)	25.6(6)	0.6(5)	-1.7(5)	1.9(5)
C18	26.5(6)	29.4(6)	23.8(5)	-0.8(5)	-3.8(5)	-2.1(5)
C16	26.6(6)	29.7(6)	24.1(5)	0.0(5)	-5.3(5)	-2.4(5)
C12	31.8(6)	28.7(6)	25.9(6)	2.9(5)	-3.1(5)	0.0(5)
C6	26.7(6)	27.6(6)	25.2(6)	1.8(5)	-1.5(5)	-0.2(5)
C20	31.4(6)	30.9(7)	25.4(6)	-5.9(5)	-3.8(5)	0.9(5)
C23	38.0(7)	38.7(7)	19.2(5)	2.5(5)	-4.9(5)	-5.6(6)
C21	28.9(6)	33.1(7)	34.2(7)	-1.7(5)	-4.9(5)	2.1(5)
C8	31.8(7)	30.8(7)	30.4(6)	4.3(5)	0.3(5)	-0.8(5)
C22	32.9(7)	34.6(7)	30.3(6)	6.2(5)	-11.8(5)	-1.7(5)
C9	28.0(7)	31.2(7)	46.3(8)	-0.7(6)	-1.3(6)	-3.2(5)
C11	41.6(8)	34.6(7)	27.4(6)	1.0(5)	-8.3(6)	1.9(6)
C10	33.1(7)	32.7(7)	41.4(7)	-4.3(6)	-14.7(6)	1.8(5)
C3	24.5(6)	44.9(8)	39.7(7)	-1.6(6)	-1.3(6)	-3.7(5)
C15	24.6(6)	39.6(8)	43.4(8)	2.7(6)	-7.9(6)	1.5(5)

Table S19. Bond Lengths for **1b** (Sjkc23f18).

Atom	Atom	Length/Å	Atom	Atom	Length/Å
O1	C2	1.3352(14)	C2	C1	1.4837(18)
O1	C3	1.4451(16)	C1	C4	1.3502(16)
O2	C2	1.2089(14)	C7	C12	1.3994(16)
O3	C14	1.3336(14)	C7	C6	1.4604(17)
O3	C15	1.4500(16)	C7	C8	1.4033(17)
O4	C14	1.2035(14)	C24	C23	1.3834(18)
N2	N1	1.2418(14)	C17	C18	1.3437(16)
N2	N3	1.1253(14)	C17	C16	1.4390(17)
N4	N5	1.2411(14)	C5	C4	1.4358(18)
N4	C13	1.4060(15)	C5	C6	1.3419(17)
N5	N6	1.1258(15)	C12	C11	1.3819(19)
N1	C1	1.4101(15)	C20	C21	1.3811(18)
C19	C24	1.4027(16)	C23	C22	1.3858(19)
C19	C18	1.4586(17)	C21	C22	1.3871(18)
C19	C20	1.4033(17)	C8	C9	1.3764(19)
C13	C14	1.4844(17)	C9	C10	1.388(2)
C13	C16	1.3510(16)	C11	C10	1.3837(19)

Table S20. Bond Angles for **1b** (Sjkc23f18)

Atom	Atom	Atom	Angle/˚	Atom	Atom	Atom	Angle/˚
C2	O1	C3	115.51(10)	C4	C1	C2	122.55(11)
C14	O3	C15	114.33(10)	C12	C7	C6	118.78(11)
N3	N2	N1	168.49(13)	C12	C7	C8	117.68(12)
N5	N4	C13	121.31(11)	C8	C7	C6	123.54(11)
N6	N5	N4	167.92(13)	C23	C24	C19	120.95(11)
N2	N1	C1	120.66(11)	C18	C17	C16	123.44(12)
C24	C19	C18	119.03(11)	C6	C5	C4	122.78(11)
C24	C19	C20	118.02(11)	C1	C4	C5	125.71(11)
C20	C19	C18	122.94(10)	C17	C18	C19	125.64(12)
N4	C13	C14	118.60(10)	C13	C16	C17	123.84(12)
C16	C13	N4	118.57(11)	C11	C12	C7	121.23(12)
C16	C13	C14	122.82(11)	C5	C6	C7	127.05(11)
O1	C2	C1	111.90(10)	C21	C20	C19	120.59(11)
O2	C2	O1	123.88(12)	C24	C23	C22	120.24(11)
O2	C2	C1	124.22(11)	C20	C21	C22	120.66(12)
O3	C14	C13	113.10(10)	C9	C8	C7	120.94(12)
O4	C14	O3	124.05(11)	C23	C22	C21	119.50(12)
O4	C14	C13	122.84(11)	C8	C9	C10	120.45(12)
N1	C1	C2	119.15(10)	C12	C11	C10	120.10(12)
C4	C1	N1	118.27(11)	C11	C10	C9	119.57(13)

Table S21. Torsion Angles for **1b** (Sjkc23f18)

A	B	C	D	Angle/˚	A	B	C	D	Angle/˚
O1	C2	C1	N1	-172.60(10)	C4	C5	C6	C7	179.42(11)
O1	C2	C1	C4	9.55(16)	C18	C19	C24	C23	-177.36(11)
O2	C2	C1	N1	7.96(18)	C18	C19	C20	C21	177.34(12)
O2	C2	C1	C4	-169.89(12)	C18	C17	C16	C13	178.23(12)
N2	N1	C1	C2	-0.71(17)	C16	C13	C14	O3	8.04(16)
N2	N1	C1	C4	177.24(11)	C16	C13	C14	O4	-171.49(12)
N4	C13	C14	O3	-172.93(10)	C16	C17	C18	C19	-178.12(11)
N4	C13	C14	O4	7.53(18)	C12	C7	C6	C5	166.29(12)
N4	C13	C16	C17	0.13(18)	C12	C7	C8	C9	-1.52(18)
N5	N4	C13	C14	-3.57(17)	C12	C11	C10	C9	-0.7(2)
N5	N4	C13	C16	175.50(11)	C6	C7	C12	C11	-177.76(11)
N1	C1	C4	C5	-1.05(18)	C6	C7	C8	C9	178.06(12)
N3	N2	N1	C1	-180(100)	C6	C5	C4	C1	-174.40(12)
C19	C24	C23	C22	-0.31(19)	C20	C19	C24	C23	1.76(18)
C19	C20	C21	C22	0.3(2)	C20	C19	C18	C17	-13.64(19)
C13	N4	N5	N6	-180(100)	C20	C21	C22	C23	1.2(2)
C2	C1	C4	C5	176.82(11)	C8	C7	C12	C11	1.84(18)
C14	C13	C16	C17	179.16(11)	C8	C7	C6	C5	-13.3(2)
C7	C12	C11	C10	-0.8(2)	C8	C9	C10	C11	1.0(2)
C7	C8	C9	C10	0.1(2)	C3	O1	C2	O2	0.21(18)
C24	C19	C18	C17	165.44(12)	C3	O1	C2	C1	-179.24(10)
C24	C19	C20	C21	-1.74(18)	C15	O3	C14	O4	1.08(17)
C24	C23	C22	C21	-1.18(19)	C15	O3	C14	C13	-178.45(10)

Table S22. Hydrogen Atom Coordinates (Å×10^4) and Isotropic Displacement Parameters (Å^2×10^3) for **1b** (Sjkc23f18).

**Atom**	***x***	***y***	***Z***	**U(eq)**
H24	5332.64	6546.06	723.51	33
H17	5237.98	7761.6	2626.1	33
H5	8301.21	5804.93	6842.36	33
H4	4763.6	4934.03	6874.74	32
H18	6831.1	6618.51	1744.43	32
H16	8816.71	6915.01	2653.82	32
H12	8417.69	4492.23	8718.99	35
H6	6852.17	4620.94	7725.21	32
H20	2502.61	7651.85	2157.37	35
H23	2769.52	6744.55	62.94	38
H21	-44.09	7844.38	1490.85	39
H8	11163.94	5669.03	7296.68	37
H22	86.09	7416.5	438.93	39
H9	13760.65	5806.12	7942.62	42
H11	11020.29	4645.41	9365.27	42
H10	13697.19	5313.58	8983.37	43
H3A	741.11	4330.89	5504.28	55
H3B	-43.7	5314.77	5678.99	55
H3C	-252.27	4485.17	6158.01	55
H15A	13812.43	6632.4	3519.48	54
H15B	12694.15	6624.25	4157.67	54
H15C	13500.41	7560.37	3895.16	54

Additional refinement for **1b** (cu meoioazidedp_0min_DK)

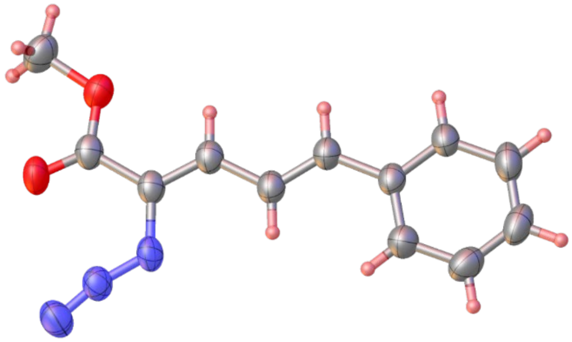

Figure S50. Refinement of **1b** (cu-meoioazidedp_0min_DK)

9.3. Crystal Face Indexing of Azides 1a and 1b

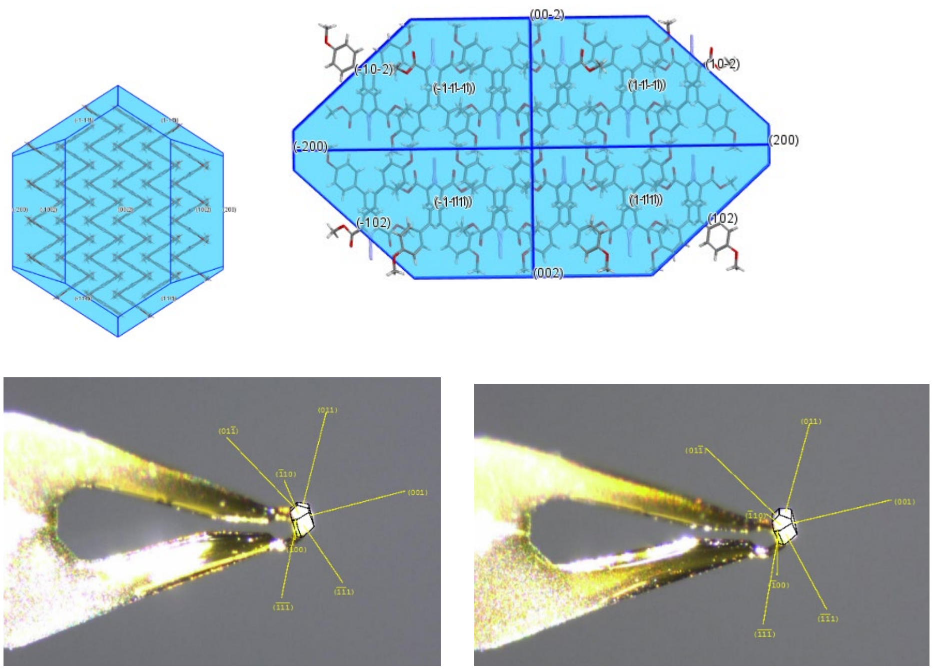

Figure S51. Crystal face indexing for azide **1a** (CCDC-2352521)

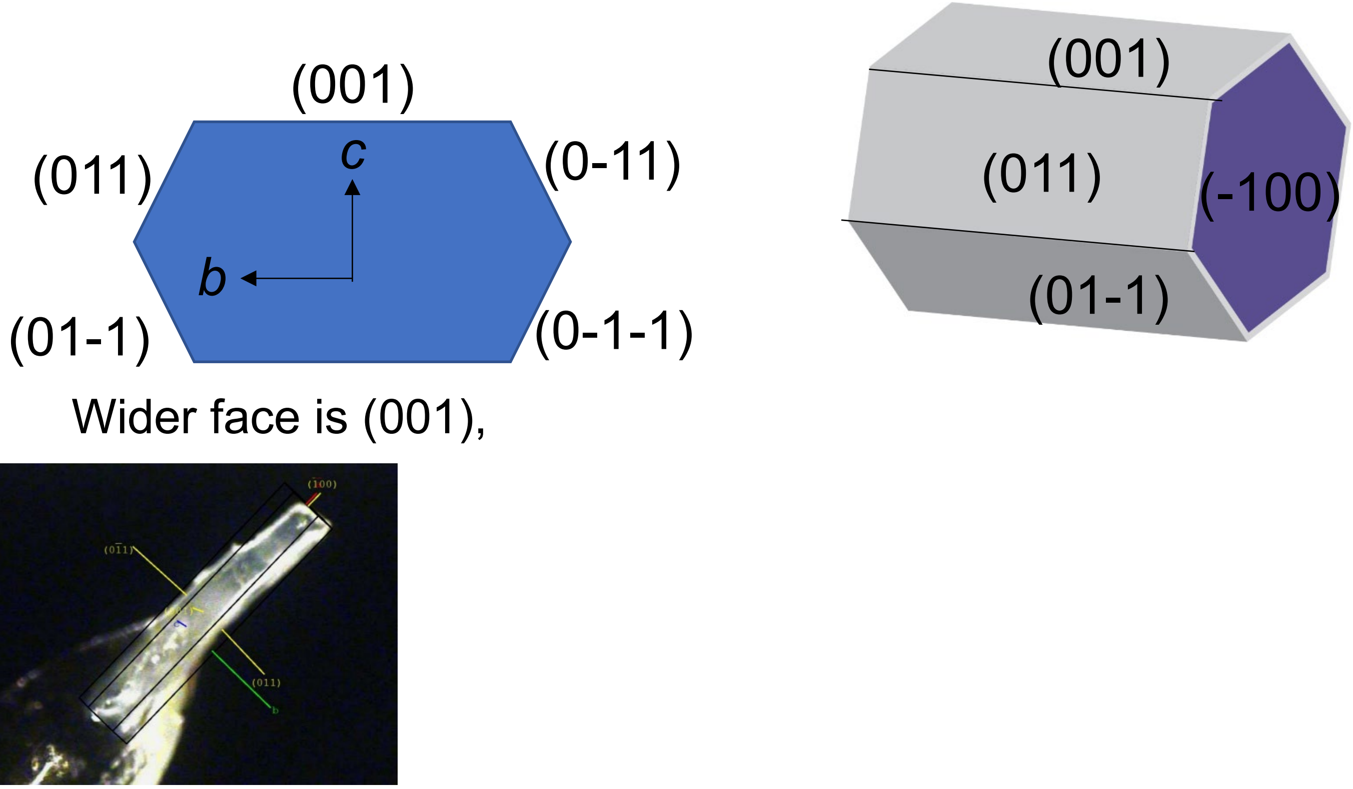

Figure S52. Crystal face indexing for azide **1a** (cu-meoioazidedp_0min_DK)

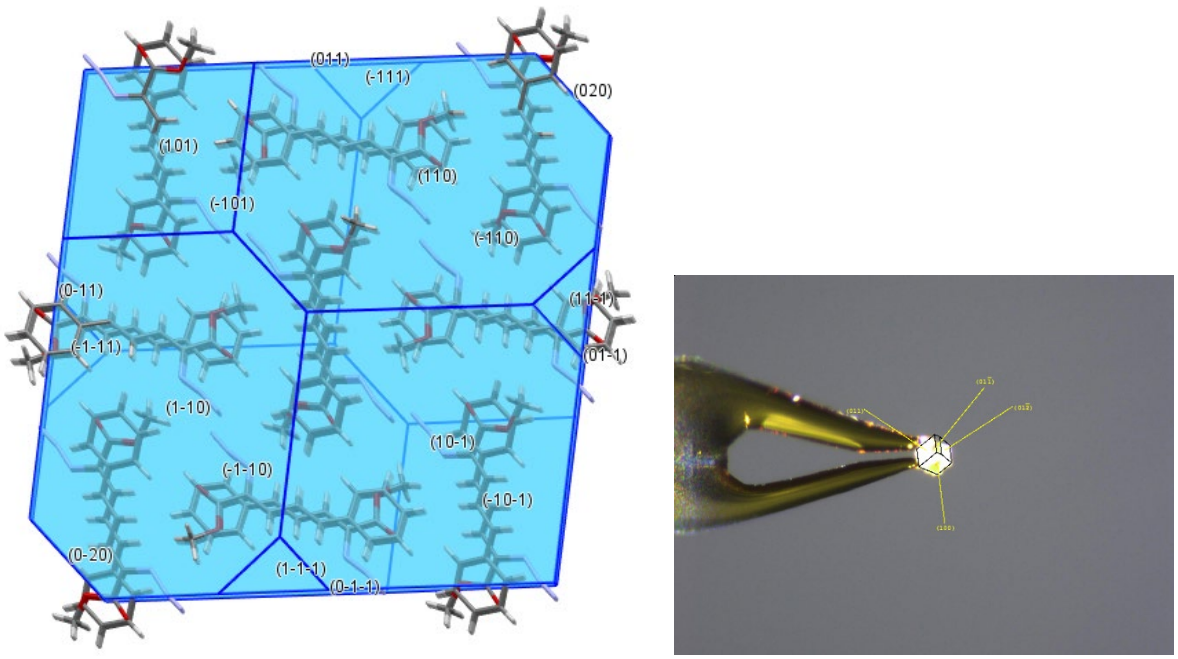

Figure S53. Crystal face indexing for azide **1b** (18075c) (CCDC-2352525).

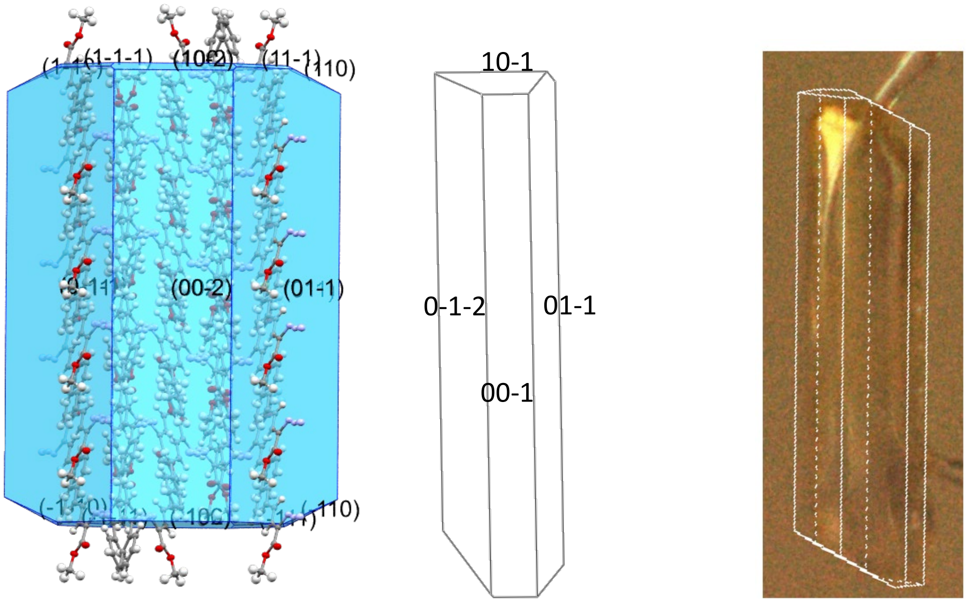

Figure S54. Crystal face indexing for azide **1b** (Sjkc23f20

9.4. Effect of Temperature on the Crystal Structure of Azide 1a.

150K

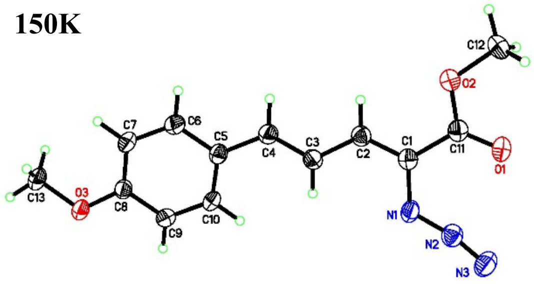

200K

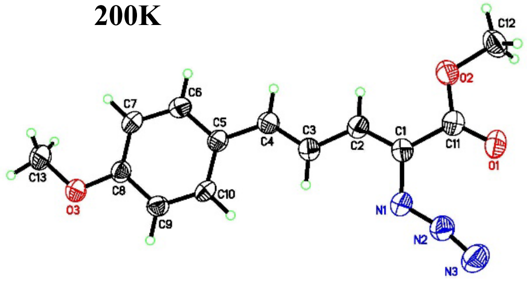

250K

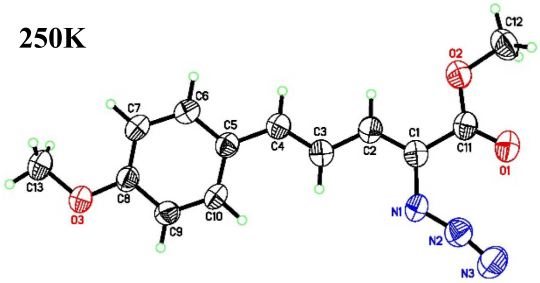

295K

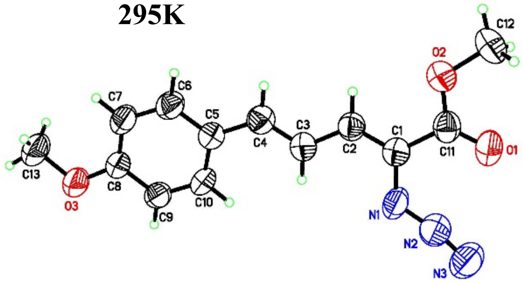

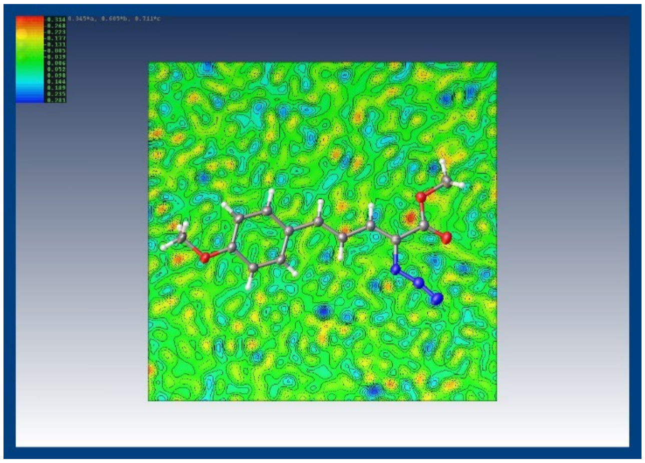

150K: Residual electron density indicating no alternate atom positions

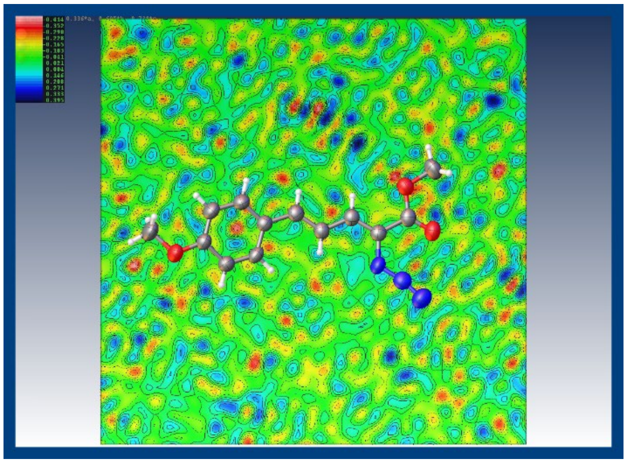

295K: Residual electron density indicating no alternate atom positions

Figure S55. Crystals Structure of 1a obtained at different temperature to rule out pedal motion experiment on **1a**.

Table S23. Crystal data and structure refinement for azide **1a** ($C_{13}H_{13}N_3O_3$) at 150K. (CCDC 2352521)

Empirical formula	$C_{13}H_{13}N_3O_3$	
Formula weight	259.26	
**Temperature**	**150(2) K**	
Wavelength	0.71073 Å	
Crystal system	Orthorhombic	
Space group	Pbca	
Unit cell dimensions	a = 13.0459(14) Å	α = 90°
	b = 8.3080(9) Å	β = 90°
	c = 23.886(3) Å	γ = 90°
Volume	2588.9(5) $Å^3$	
Z	8	
Density (calculated)	1.330 Mg/m^3	
Absorption coefficient	0.097 mm^{-1}	
F(000)	1088	
Crystal size	0.182 x 0.135 x 0.070 mm^3	
θ range for data collection	1.705 to 27.531°	
Index ranges	-16 ≤ h ≤ 16, -10 ≤ k ≤ 10, -31 ≤ l ≤ 31	
Reflections collected	33715	
Independent reflections	2977 [R_{int} = 0.0643]	
Completeness to θ = 25.242°	100.0 %	
Absorption correction	Numerical	
Max. and min. transmission	0.862 and 0.802	
Refinement method	Full-matrix least-squares on F^2	
Data / restraints / parameters	2977 / 0 / 174	
Goodness-of-fit on F^2	1.033	
Final R indices [I>2σ(I)]	R1 = 0.0401, wR2 = 0.0798	
R indices (all data)	R1 = 0.0783, wR2 = 0.0936	
Largest diff. peak and hole	0.199 and -0.189 $eÅ^{-3}$	

Table S24. Atomic coordinates [x10^4] and equivalent isotropic displacement parameters [$Å^2$x10^3] for azide **1a** ($C_{13}H_{13}N_3O_3$) at 150K (CCDC-2352521). U(eq) is defined as one third of the trace of the orthogonalized Uij tensor.

	x	y	z	U(eq)
O(1)	974(1)	6686(1)	9065(1)	37(1)
O(2)	440(1)	7256(1)	8190(1)	33(1)
O(3)	5761(1)	1717(1)	5501(1)	36(1)
N(1)	2616(1)	4797(2)	8624(1)	34(1)
N(2)	2467(1)	4646(2)	9138(1)	33(1)
N(3)	2475(1)	4362(2)	9599(1)	42(1)
C(1)	1918(1)	5669(2)	8290(1)	27(1)
C(2)	2061(1)	5694(2)	7732(1)	28(1)
C(3)	2864(1)	4904(2)	7430(1)	28(1)
C(4)	2913(1)	4926(2)	6868(1)	28(1)
C(5)	3662(1)	4118(2)	6513(1)	26(1)
C(6)	3521(1)	4090(2)	5934(1)	31(1)
C(7)	4197(1)	3309(2)	5578(1)	30(1)
C(8)	5046(1)	2537(2)	5804(1)	27(1)
C(9)	5212(1)	2564(2)	6382(1)	28(1)
C(10)	4532(1)	3342(2)	6728(1)	26(1)
C(11)	1076(1)	6569(2)	8564(1)	28(1)
C(12)	-375(1)	8221(2)	8425(1)	40(1)
C(13)	5691(1)	1836(2)	4902(1)	43(1)
H(2A)	1581	6295	7518	34
H(3A)	3379	4346	7633	33
H(4A)	2402	5537	6681	34
H(6A)	2943	4624	5779	37
H(7A)	4081	3302	5185	36
H(9A)	5795	2044	6536	33
H(10A)	4654	3355	7120	32
H(12A)	-788	8680	8122	60
H(12B)	-79	9093	8649	60
H(12C)	-810	7546	8663	60
H(13A)	6258	1241	4731	64
H(13B)	5729	2970	4791	64
H(13C)	5037	1380	4777	64

Table S25. Bond lengths [Å] and angles [°] for azide **1a** ($C_{13}H_{13}N_3O_3$) at 150K (CCDC-2352521).

O(1)-C(11)	1.2078(18)	O(2)-C(11)	1.3456(19)	O(2)-
C(12)	1.4451(19)	O(3)-C(8)	1.3632(18)	O(3)-
C(13)	1.4356(18)	N(1)-N(2)	1.2490(17)	N(1)-
C(1)	1.411(2)	N(2)-N(3)	1.1278(17)	C(1)-
C(2)	1.346(2)	C(1)-C(11)	1.481(2)	C(2)-
C(3)	1.431(2)	C(3)-C(4)	1.343(2)	C(4)-
C(5)	1.457(2)	C(5)-C(6)	1.396(2)	C(5)-
C(10)	1.403(2)	C(6)-C(7)	1.387(2)	C(7)-
C(8)	1.390(2)	C(8)-C(9)	1.397(2)	C(9)-
C(10)	1.373(2)			

C(11)-O(2)-C(12)	115.54(12)	C(8)-O(3)-C(13)	116.83(12)	N(2)-
N(1)-C(1)	120.46(13)	N(3)-N(2)-N(1)	168.65(17)	C(2)-
C(1)-N(1)	118.57(14)	C(2)-C(1)-C(11)	122.19(14)	N(1)-
C(1)-C(11)	119.21(13)	C(1)-C(2)-C(3)	126.42(15)	C(4)-
C(3)-C(2)	122.18(15)	C(3)-C(4)-C(5)	127.39(15)	C(6)-
C(5)-C(10)	117.43(14)	C(6)-C(5)-C(4)	119.78(14)	C(10)-
C(5)-C(4)	122.79(13)	C(7)-C(6)-C(5)	122.22(15)	C(6)-
C(7)-C(8)	118.90(14)	O(3)-C(8)-C(7)	124.65(13)	O(3)-
C(8)-C(9)	115.33(14)	C(7)-C(8)-C(9)	120.02(14)	C(10)-
C(9)-C(8)	120.18(15)	C(9)-C(10)-C(5)	121.23(14)	O(1)-
C(11)-O(2)	123.77(14)	O(1)-C(11)-C(1)	124.04(15)	O(2)-
C(11)-C(1)	112.19(13)			

Table S26. Torsion angles [°] for azide **1a** ($C_{13}H_{13}N_3O_3$) at 150K (CCDC-2352521).

C(1)-N(1)-N(2)-N(3)	178.3(8)	N(2)-N(1)-C(1)-C(2)	-174.66(15)	N(2)-
N(1)-C(1)-C(11)	7.4(2)	N(1)-C(1)-C(2)-C(3)	0.3(2)	C(11)-
C(1)-C(2)-C(3)	178.22(14)	C(1)-C(2)-C(3)-C(4)	176.32(16)	C(2)-
C(3)-C(4)-C(5)	-177.23(15)	C(3)-C(4)-C(5)-C(6)	171.41(16)	C(3)-
C(4)-C(5)-C(10)	-8.2(3)	C(10)-C(5)-C(6)-C(7)	1.1(2)	C(4)-
C(5)-C(6)-C(7)	-178.54(15)	C(5)-C(6)-C(7)-C(8)	-0.4(2)	C(13)-
O(3)-C(8)-C(7)	8.0(2)	C(13)-O(3)-C(8)-C(9)	-172.22(15)	C(6)-
C(7)-C(8)-O(3)	179.37(15)	C(6)-C(7)-C(8)-C(9)	-0.4(2)	O(3)-
C(8)-C(9)-C(10)	-179.27(14)	C(7)-C(8)-C(9)-C(10)	0.5(2)	C(8)-
C(9)-C(10)-C(5)	0.2(2)	C(6)-C(5)-C(10)-C(9)	-0.9(2)	C(4)-
C(5)-C(10)-C(9)	178.66(14)	C(12)-O(2)-C(11)-O(1)	2.2(2)	C(12)-
O(2)-C(11)-C(1)	-177.15(13)	C(2)-C(1)-C(11)-O(1)	-173.48(16)	N(1)-
C(1)-C(11)-O(1)	4.4(2)	C(2)-C(1)-C(11)-O(2)	5.9(2)	N(1)-
C(1)-C(11)-O(2)	-176.23(13)			

Table S27. Anisotropic displacement parameters [Å^{2}x10^3] for azide **1a** ($C_{13}H_{13}N_3O_3$) at 150K (CCDC-2352521). The anisotropic displacement factor exponent takes the form: $-2\pi^2[h^2a^{*2}U_{11}+ ... +2hka^*b^*U_{12}]$

	U_{11}	U_{22}	U_{33}	U_{23}	U_{13}	U_{12}
O(1)	42(1)	42(1)	26(1)	-3(1)	6(1)	4(1)
O(2)	33(1)	35(1)	29(1)	1(1)	3(1)	6(1)
O(3)	41(1)	46(1)	21(1)	0(1)	3(1)	14(1)
N(1)	34(1)	46(1)	22(1)	-1(1)	2(1)	6(1)
N(2)	30(1)	38(1)	32(1)	0(1)	1(1)	-1(1)
N(3)	43(1)	54(1)	29(1)	5(1)	1(1)	0(1)
C(1)	27(1)	25(1)	28(1)	-2(1)	1(1)	-3(1)
C(2)	30(1)	27(1)	27(1)	-2(1)	1(1)	0(1)
C(3)	28(1)	26(1)	29(1)	-1(1)	2(1)	1(1)
C(4)	28(1)	30(1)	27(1)	1(1)	1(1)	1(1)
C(5)	28(1)	26(1)	24(1)	0(1)	2(1)	-3(1)
C(6)	30(1)	35(1)	27(1)	1(1)	-3(1)	3(1)
C(7)	36(1)	34(1)	20(1)	-1(1)	-3(1)	1(1)
C(8)	31(1)	27(1)	23(1)	-1(1)	2(1)	-1(1)
C(9)	28(1)	29(1)	26(1)	2(1)	-1(1)	0(1)
C(10)	30(1)	30(1)	19(1)	1(1)	-1(1)	-3(1)
C(11)	30(1)	26(1)	29(1)	-1(1)	3(1)	-4(1)
C(12)	40(1)	39(1)	41(1)	-1(1)	8(1)	13(1)
C(13)	51(1)	57(1)	21(1)	0(1)	5(1)	16(1)

Table S28. Crystal data and structure refinement for azide **1a** ($C_{13}H_{13}N_3O_3$) at 200K (CCDC-2352522) .

Empirical formula	$C_{13}H_{13}N_3O_3$	
Formula weight	259.26	
Temperature	200(2) K	
Wavelength	0.71073 Å	
Crystal system	Orthorhombic	
Space group	Pbca	
Unit cell dimensions	a = 13.0646(8) Å	α = 90°
	b = 8.3471(5) Å	β = 90°
	c = 23.9088(15) Å	γ = 90°
Volume	2607.3(3) $Å^3$	
Z	8	
Density (calculated)	1.321 Mg/m^3	
Absorption coefficient	0.096 mm^{-1}	
F(000)	1088	
Crystal size	0.182 x 0.135 x 0.070 mm^3	
θ range for data collection	1.703 to 26.400°	
Index ranges	-16 ≤ h ≤ 16, -10 ≤ k ≤ 10, -29 ≤ l ≤ 29	
Reflections collected	31408	
Independent reflections	2682 [R_{int} = 0.0651]	
Completeness to θ = 25.242°	100.0 %	
Absorption correction	Semi-empirical from equivalents	
Max. and min. transmission	0.862 and 0.819	
Refinement method	Full-matrix least-squares on F^2	
Data / restraints / parameters	2682 / 0 / 174	
Goodness-of-fit on F^2	1.020	
Final R indices [I>2σ(I)]	R1 = 0.0393, wR2 = 0.0880	
R indices (all data)	R1 = 0.0762, wR2 = 0.1021	
Largest diff. peak and hole	0.172 and -0.172 $eÅ^{-3}$	

Table S29. Atomic coordinates [x10^4] and equivalent isotropic displacement parameters [$Å^2$x10^3] for azide **1a** ($C_{13}H_{13}N_3O_3$) at 200K CCDC-2352522. U(eq) is defined as one third of the trace of the orthogonalized Uij tensor.

	x	y	z	U(eq)
O(1)	978(1)	6687(2)	9060(1)	49(1)
O(2)	446(1)	7263(1)	8189(1)	43(1)
O(3)	5749(1)	1736(2)	5504(1)	47(1)
N(1)	2614(1)	4814(2)	8621(1)	45(1)
N(2)	2466(1)	4664(2)	9130(1)	42(1)
N(3)	2479(1)	4385(2)	9593(1)	55(1)
C(1)	1915(1)	5677(2)	8286(1)	33(1)
C(2)	2060(1)	5700(2)	7730(1)	35(1)
C(3)	2857(1)	4914(2)	7428(1)	35(1)
C(4)	2908(1)	4924(2)	6869(1)	36(1)
C(5)	3653(1)	4124(2)	6514(1)	33(1)
C(6)	3514(1)	4088(2)	5938(1)	39(1)
C(7)	4187(1)	3311(2)	5582(1)	39(1)
C(8)	5034(1)	2547(2)	5807(1)	34(1)
C(9)	5198(1)	2582(2)	6383(1)	35(1)
C(10)	4522(1)	3354(2)	6727(1)	34(1)
C(11)	1077(1)	6574(2)	8561(1)	36(1)
C(12)	-367(2)	8226(2)	8421(1)	52(1)
C(13)	5683(2)	1857(3)	4908(1)	57(1)
H(2A)	1583	6300	7516	42
H(3A)	3372	4363	7632	42
H(4A)	2395	5525	6681	43
H(6A)	2936	4615	5782	47
H(7A)	4071	3302	5190	47
H(9A)	5782	2068	6537	42
H(10A)	4645	3368	7118	40
H(12A)	-772	8692	8117	79
H(12B)	-73	9087	8649	79
H(12C)	-808	7554	8655	79
H(13A)	6259	1284	4737	85
H(13B)	5708	2988	4798	85
H(13C)	5038	1384	4780	85

Table S30. Bond lengths [Å] and angles [°] for azide **1a** ($C_{13}H_{13}N_3O_3$) at 200K (CCDC-2352522).

O(1)-C(11)	1.205(2)	O(2)-C(11)	1.342(2)	O(2)-
C(12)	1.443(2)	O(3)-C(8)	1.362(2)	O(3)-
C(13)	1.432(2)	N(1)-N(2)	1.2402(19)	N(1)-
C(1)	1.412(2)	N(2)-N(3)	1.1300(19)	C(1)-
C(2)	1.342(2)	C(1)-C(11)	1.480(2)	C(2)-
C(3)	1.427(2)	C(3)-C(4)	1.340(2)	C(4)-
C(5)	1.454(2)	C(5)-C(6)	1.390(2)	C(5)-
C(10)	1.401(2)	C(6)-C(7)	1.384(2)	C(7)-
C(8)	1.385(2)	C(8)-C(9)	1.393(2)	C(9)-
C(10)	1.368(2)			

C(11)-O(2)-C(12)	115.86(14)	C(8)-O(3)-C(13)	116.98(14)	N(2)-
N(1)-C(1)	120.49(15)	N(3)-N(2)-N(1)	168.49(19)	C(2)-
C(1)-N(1)	118.47(15)	C(2)-C(1)-C(11)	122.47(16)	N(1)-
C(1)-C(11)	119.01(14)	C(1)-C(2)-C(3)	126.73(16)	C(4)-
C(3)-C(2)	122.62(16)	C(3)-C(4)-C(5)	127.80(16)	C(6)-
C(5)-C(10)	117.13(15)	C(6)-C(5)-C(4)	120.05(15)	C(10)-
C(5)-C(4)	122.82(14)	C(7)-C(6)-C(5)	122.37(16)	C(6)-
C(7)-C(8)	119.01(15)	O(3)-C(8)-C(7)	124.73(14)	O(3)-
C(8)-C(9)	115.48(15)	C(7)-C(8)-C(9)	119.79(16)	C(10)-
C(9)-C(8)	120.30(16)	C(9)-C(10)-C(5)	121.39(15)	O(1)-
C(11)-O(2)	123.91(16)	O(1)-C(11)-C(1)	123.95(16)	O(2)-
C(11)-C(1)	112.13(14)			

Table S31. Torsion angles [°] for azide **1a** ($C_{13}H_{13}N_3O_3$) at 200K (CCDC-2352522).

C(1)-N(1)-N(2)-N(3)	179.8(9)	N(2)-N(1)-C(1)-C(2)	-174.65(17)	N(2)-
N(1)-C(1)-C(11)	7.8(2)	N(1)-C(1)-C(2)-C(3)	0.8(3)	C(11)-
C(1)-C(2)-C(3)	178.22(16)	C(1)-C(2)-C(3)-C(4)	175.87(18)	C(2)-
C(3)-C(4)-C(5)	-177.45(16)	C(3)-C(4)-C(5)-C(6)	171.54(18)	C(3)-
C(4)-C(5)-C(10)	-7.8(3)	C(10)-C(5)-C(6)-C(7)	1.0(3)	C(4)-
C(5)-C(6)-C(7)	-178.43(16)	C(5)-C(6)-C(7)-C(8)	-0.3(3)	C(13)-
O(3)-C(8)-C(7)	8.0(3)	C(13)-O(3)-C(8)-C(9)	-171.73(17)	C(6)-
C(7)-C(8)-O(3)	179.72(16)	C(6)-C(7)-C(8)-C(9)	-0.5(3)	O(3)-
C(8)-C(9)-C(10)	-179.55(15)	C(7)-C(8)-C(9)-C(10)	0.7(3)	C(8)-
C(9)-C(10)-C(5)	0.0(3)	C(6)-C(5)-C(10)-C(9)	-0.8(2)	C(4)-
C(5)-C(10)-C(9)	178.56(16)	C(12)-O(2)-C(11)-O(1)	2.0(2)	C(12)-
O(2)-C(11)-C(1)	-177.21(14)	C(2)-C(1)-C(11)-O(1)	-173.47(17)	N(1)-
C(1)-C(11)-O(1)	4.0(3)	C(2)-C(1)-C(11)-O(2)	5.8(2)	N(1)-
C(1)-C(11)-O(2)	-176.81(14)			

Table S32. Anisotropic displacement parameters [$Å^2 x10^3$] for azide **1a** ($C_{13}H_{13}N_3O_3$) at 200K (CCDC-2352522). The anisotropic displacement factor exponent takes the form: -2π2[h 2a*2U11+... +2hka*b*U12]

	U_{11}	U_{22}	U_{33}	U_{23}	U_{13}	U_{12}
O(1)	55(1)	57(1)	33(1)	-4(1)	8(1)	6(1)
O(2)	44(1)	46(1)	39(1)	2(1)	6(1)	9(1)
O(3)	53(1)	60(1)	28(1)	-1(1)	4(1)	18(1)
N(1)	44(1)	60(1)	30(1)	-1(1)	2(1)	9(1)
N(2)	39(1)	49(1)	39(1)	1(1)	1(1)	0(1)
N(3)	57(1)	70(1)	38(1)	7(1)	1(1)	0(1)
C(1)	34(1)	32(1)	35(1)	-2(1)	2(1)	-3(1)
C(2)	40(1)	34(1)	32(1)	0(1)	1(1)	0(1)
C(3)	37(1)	35(1)	35(1)	-1(1)	2(1)	1(1)
C(4)	36(1)	38(1)	34(1)	1(1)	1(1)	2(1)
C(5)	35(1)	33(1)	30(1)	1(1)	1(1)	-3(1)
C(6)	38(1)	46(1)	33(1)	1(1)	-3(1)	4(1)
C(7)	46(1)	47(1)	26(1)	-2(1)	-3(1)	2(1)
C(8)	39(1)	35(1)	28(1)	0(1)	2(1)	1(1)
C(9)	34(1)	38(1)	33(1)	3(1)	-1(1)	1(1)
C(10)	38(1)	39(1)	23(1)	2(1)	-2(1)	-4(1)
C(11)	40(1)	34(1)	36(1)	0(1)	4(1)	-5(1)
C(12)	50(1)	51(1)	56(1)	-1(1)	9(1)	18(1)
C(13)	69(1)	74(2)	27(1)	-2(1)	6(1)	21(1)

Table S33. Crystal data and structure refinement for azide **1a** ($C_{13}H_{13}N_3O_3$) at 250K (CCDC-2352523).

Empirical formula	$C_{13}H_{13}N_3O_3$	
Formula weight	259.26	
**Temperature**	**250(2) K**	
Wavelength	0.71073 Å	
Crystal system	Orthorhombic	
Space group	Pbca	
Unit cell dimensions	a = 13.1194(10) Å	α = 90°
	b = 8.3878(7) Å	β = 90°
	c = 23.9199(19) Å	γ = 90°
Volume	2632.2(4) $Å^3$	
Z	8	
Density (calculated)	1.308 Mg/m^3	
Absorption coefficient	0.095 mm^{-1}	
F(000)	1088	
Crystal size	0.182 x 0.135 x 0.070 mm^3	
θ range for data collection	1.703 to 26.418°	
Index ranges	-16 ≤ h ≤ 16, -10 ≤ k ≤ 10, -29 ≤ l ≤ 29	
Reflections collected	31763	
Independent reflections	2714 [R_{int} = 0.0754]	
Completeness to θ = 25.242°	100.0 %	
Absorption correction	Semi-empirical from equivalents	
Max. and min. transmission	0.862 and 0.811	
Refinement method	Full-matrix least-squares on F^2	
Data / restraints / parameters	2714 / 0 / 174	
Goodness-of-fit on F^2	1.003	
Final R indices [I>2σ(I)]	R1 = 0.0429, wR2 = 0.0901	
R indices (all data)	R1 = 0.1000, wR2 = 0.1096	
Largest diff. peak and hole	0.152 and -0.132 $eÅ^{-3}$	

Table S34. Atomic coordinates [x10[4]] and equivalent isotropic displacement parameters [$Å^2$x10^3] for azide 1a ($C_{13}H_{13}N_3O_3$) at 250K (CCDC-2352523). U(eq) is defined as one third of the trace of the orthogonalized U_{ij} tensor.

	x	y	z	U(eq)
O(1)	984(1)	6685(2)	9057(1)	63(1)
O(2)	454(1)	7253(2)	8189(1)	56(1)
O(3)	5734(1)	1759(2)	5511(1)	63(1)
N(1)	2612(1)	4825(2)	8617(1)	57(1)
N(2)	2474(1)	4680(2)	9127(1)	57(1)
N(3)	2490(2)	4409(3)	9590(1)	74(1)
C(1)	1917(1)	5675(2)	8286(1)	44(1)
C(2)	2056(2)	5691(2)	7729(1)	46(1)
C(3)	2849(1)	4906(2)	7429(1)	46(1)
C(4)	2900(2)	4916(2)	6871(1)	47(1)
C(5)	3642(1)	4122(2)	6517(1)	42(1)
C(6)	3503(2)	4075(2)	5944(1)	52(1)
C(7)	4176(2)	3307(3)	5590(1)	53(1)
C(8)	5020(2)	2561(2)	5814(1)	45(1)
C(9)	5183(2)	2601(2)	6387(1)	46(1)
C(10)	4508(2)	3363(2)	6730(1)	44(1)
C(11)	1087(2)	6567(2)	8559(1)	47(1)
C(12)	-358(2)	8216(3)	8418(1)	69(1)
C(13)	5675(2)	1875(3)	4915(1)	74(1)
H(2A)	1584	6277	7517	55
H(3A)	3355	4360	7631	55
H(4A)	2395	5509	6686	56
H(6A)	2930	4585	5789	62
H(7A)	4061	3293	5202	63
H(9A)	5760	2101	6540	55
H(10A)	4629	3378	7117	53
H(12A)	-753	8677	8116	104
H(12B)	-69	9062	8645	104
H(12C)	-797	7556	8648	104
H(13A)	6240	1300	4749	112
H(13B)	5708	2987	4805	112
H(13C)	5037	1417	4787	112

Table S35. Bond lengths [Å] and angles [°] for azide **1a** ($C_{13}H_{13}N_3O_3$) at 250K (CCDC-2352523).

O(1)-C(11)	1.203(2)	O(2)-C(11)	1.344(2)	O(2)-
C(12)	1.445(2)	O(3)-C(8)	1.362(2)	O(3)-
C(13)	1.431(2)	N(1)-N(2)	1.239(2)	N(1)-
C(1)	1.402(2)	N(2)-N(3)	1.130(2)	C(1)-
C(2)	1.344(2)	C(1)-C(11)	1.475(3)	C(2)-
C(3)	1.426(3)	C(3)-C(4)	1.335(2)	C(4)-
C(5)	1.452(3)	C(5)-C(6)	1.384(2)	C(5)-
C(10)	1.398(3)	C(6)-C(7)	1.383(3)	C(7)-
C(8)	1.380(3)	C(8)-C(9)	1.387(3)	C(9)-
C(10)	1.365(3)			

C(11)-O(2)-C(12)	116.40(16)	C(8)-O(3)-C(13)	117.36(16)	N(2)-
N(1)-C(1)	120.71(17)	N(3)-N(2)-N(1)	168.9(2)	C(2)-
C(1)-N(1)	118.47(17)	C(2)-C(1)-C(11)	122.31(19)	N(1)-
C(1)-C(11)	119.18(16)	C(1)-C(2)-C(3)	126.43(19)	C(4)-
C(3)-C(2)	122.49(19)	C(3)-C(4)-C(5)	127.82(19)	C(6)-
C(5)-C(10)	117.05(18)	C(6)-C(5)-C(4)	120.18(18)	C(10)-
C(5)-C(4)	122.77(16)	C(7)-C(6)-C(5)	122.35(19)	C(8)-
C(7)-C(6)	119.11(18)	O(3)-C(8)-C(7)	124.66(17)	O(3)-
C(8)-C(9)	115.59(18)	C(7)-C(8)-C(9)	119.74(19)	C(10)-
C(9)-C(8)	120.33(19)	C(9)-C(10)-C(5)	121.42(17)	O(1)-
C(11)-O(2)	123.25(19)	O(1)-C(11)-C(1)	124.3(2)	O(2)-
C(11)-C(1)	112.44(16)			

Table S36. Torsion angles [°] for azide **1a** ($C_{13}H_{13}N_3O_3$) at 250K (CCDC-2352523).

C(1)-N(1)-N(2)-N(3)	179.4(11)	N(2)-N(1)-C(1)-C(2)	-174.7(2)	N(2)-
N(1)-C(1)-C(11)	7.6(3)	N(1)-C(1)-C(2)-C(3)	0.6(3)	C(11)-
C(1)-C(2)-C(3)	178.23(18)	C(1)-C(2)-C(3)-C(4)	176.4(2)	C(2)-
C(3)-C(4)-C(5)	-177.62(18)	C(3)-C(4)-C(5)-C(6)	171.1(2)	C(3)-
C(4)-C(5)-C(10)	-8.1(3)	C(10)-C(5)-C(6)-C(7)	0.6(3)	C(4)-
C(5)-C(6)-C(7)	-178.67(19)	C(5)-C(6)-C(7)-C(8)	-0.2(3)	C(13)-
O(3)-C(8)-C(7)	8.5(3)	C(13)-O(3)-C(8)-C(9)	-171.50(19)	C(6)-
C(7)-C(8)-O(3)	179.65(19)	C(6)-C(7)-C(8)-C(9)	-0.4(3)	O(3)-
C(8)-C(9)-C(10)	-179.50(18)	C(7)-C(8)-C(9)-C(10)	0.6(3)	C(8)-
C(9)-C(10)-C(5)	-0.1(3)	C(6)-C(5)-C(10)-C(9)	-0.5(3)	C(4)-
C(5)-C(10)-C(9)	178.79(18)	C(12)-O(2)-C(11)-O(1)	2.3(3)	C(12)-
O(2)-C(11)-C(1)	-177.19(17)	C(2)-C(1)-C(11)-O(1)	-173.9(2)	N(1)-
C(1)-C(11)-O(1)	3.7(3)	C(2)-C(1)-C(11)-O(2)	5.5(3)	N(1)-
C(1)-C(11)-O(2)	-176.86(17)			

Table S37. Anisotropic displacement parameters [Å^{2}x10^3] for azide **1a** ($C_{13}H_{13}N_3O_3$) at 250K (CCDC-2352523). The anisotropic displacement factor exponent takes the form: $-2\pi^2[h^2a^{*2}U_{11}+ ... +2hka^*b^*U_{12}]$

	U_{11}	U_{22}	U_{33}	U_{23}	U_{13}	U_{12}
O(1)	70(1)	74(1)	45(1)	-4(1)	10(1)	9(1)
O(2)	56(1)	60(1)	52(1)	1(1)	7(1)	10(1)
O(3)	70(1)	82(1)	37(1)	-2(1)	5(1)	25(1)
N(1)	56(1)	80(1)	35(1)	-1(1)	4(1)	11(1)
N(2)	51(1)	66(1)	53(1)	1(1)	1(1)	-1(1)
N(3)	74(1)	97(2)	51(1)	9(1)	1(1)	1(1)
C(1)	42(1)	43(1)	45(1)	-4(1)	3(1)	-4(1)
C(2)	51(1)	44(1)	42(1)	-1(1)	4(1)	0(1)
C(3)	47(1)	45(1)	46(1)	-2(1)	3(1)	-1(1)
C(4)	48(1)	49(1)	43(1)	2(1)	2(1)	0(1)
C(5)	44(1)	46(1)	38(1)	1(1)	2(1)	-3(1)
C(6)	52(1)	61(1)	42(1)	1(1)	-5(1)	6(1)
C(7)	61(1)	63(1)	35(1)	-2(1)	-4(1)	5(1)
C(8)	53(1)	47(1)	35(1)	-1(1)	3(1)	1(1)
C(9)	46(1)	50(1)	42(1)	5(1)	-1(1)	3(1)
C(10)	51(1)	52(1)	31(1)	1(1)	0(1)	-4(1)
C(11)	50(1)	47(1)	43(1)	-2(1)	5(1)	-6(1)
C(12)	66(2)	68(2)	74(2)	-1(1)	13(1)	22(1)
C(13)	89(2)	97(2)	37(1)	-1(1)	8(1)	25(2)

Table S38. Crystal data and structure refinement for azide **1a** ($C_{13}H_{13}N_3O_3$) at 295K (CCDC-2352524).

Empirical formula	$C_{13}H_{13}N_3O_3$	
Formula weight	259.26	
Temperature	295(2) K	
Wavelength	0.71073 Å	
Crystal system	Orthorhombic	
Space group	Pbca	
Unit cell dimensions	a = 13.1408(16) Å	α = 90°
	b = 8.4392(10) Å	β = 90°
	c = 23.972(3) Å	γ = 90°
Volume	2658.4(5) Å^3	
Z	8	
Density (calculated)	1.296 Mg/m^3	
Absorption coefficient	0.094 mm^{-1}	
F(000)	1088	
Crystal size	0.182 x 0.135 x 0.070 mm^3	
θ range for data collection	1.699 to 26.391°	
Index ranges	$-16 \le h \le 16$, $-10 \le k \le 10$, $-29 \le l \le 29$	
Reflections collected	31003	
Independent reflections	2734 [R_{int} = 0.1385]	
Completeness to θ = 25.242°	100.0 %	
Absorption correction	Semi-empirical from equivalents	
Max. and min. transmission	0.862 and 0.772	
Refinement method	Full-matrix least-squares on F^2	
Data / restraints / parameters	2734 / 0 / 174	
Goodness-of-fit on F^2	1.005	
Final R indices [I>2σ(I)]	R1 = 0.0560, wR2 = 0.0955	
R indices (all data)	R1 = 0.1597, wR2 = 0.1254	
Largest diff. peak and hole	0.159 and -0.125 eÅ^{-3}	

Table S39. Atomic coordinates [x10[4]] and equivalent isotropic displacement parameters [Å^{2}x10^3] for azide **1a** ($C_{13}H_{13}N_3O_3$) at 295K (CCDC-2352524). U(eq) is defined as one third of the trace of the orthogonalized Uij tensor.

	x	y	z	U(eq)
O(1)	986(2)	6679(3)	9055(1)	79(1)
O(2)	454(2)	7247(2)	8190(1)	68(1)
O(3)	5721(2)	1778(3)	5519(1)	81(1)
N(1)	2609(2)	4841(4)	8611(1)	72(1)
N(2)	2479(2)	4695(3)	9120(1)	71(1)
N(3)	2501(2)	4432(4)	9584(1)	97(1)
C(1)	1916(2)	5686(3)	8284(1)	54(1)
C(2)	2050(2)	5690(3)	7732(1)	56(1)
C(3)	2838(2)	4905(3)	7431(1)	56(1)
C(4)	2890(2)	4909(3)	6876(1)	58(1)
C(5)	3626(2)	4122(3)	6521(1)	52(1)
C(6)	3491(2)	4066(4)	5950(1)	65(1)
C(7)	4166(2)	3307(4)	5597(1)	66(1)
C(8)	5009(2)	2572(4)	5820(1)	56(1)
C(9)	5168(2)	2614(3)	6390(1)	58(1)
C(10)	4493(2)	3371(3)	6733(1)	55(1)
C(11)	1087(2)	6571(4)	8559(1)	58(1)
C(12)	-357(3)	8206(4)	8419(1)	88(1)
C(13)	5663(3)	1902(5)	4922(1)	98(1)
H(2A)	1581	6264	7523	67
H(3A)	3338	4367	7630	68
H(4A)	2390	5490	6693	70
H(6A)	2924	4559	5796	78
H(7A)	4052	3292	5214	80
H(9A)	5739	2124	6541	70
H(10A)	4613	3386	7115	66
H(12A)	-761	8629	8120	131
H(12B)	-71	9061	8632	131
H(12C)	-778	7565	8656	131
H(13A)	6223	1340	4758	147
H(13B)	5694	2997	4816	147
H(13C)	5034	1451	4795	147

Table S40. Bond lengths [Å] and angles [°] for azide **1a** ($C_{13}H_{13}N_3O_3$) at 295K (CCDC-2352524).

O(1)-C(11)	1.200(3)	O(2)-C(11)	1.340(3)	O(2)-
C(12)	1.446(3)	O(3)-C(8)	1.359(3)	O(3)-
C(13)	1.435(3)	N(1)-N(2)	1.238(3)	N(1)-
C(1)	1.398(3)	N(2)-N(3)	1.134(3)	C(1)-
C(2)	1.334(3)	C(1)-C(11)	1.476(4)	C(2)-
C(3)	1.427(4)	C(3)-C(4)	1.332(3)	C(4)-
C(5)	1.450(4)	C(5)-C(6)	1.381(4)	C(5)-
C(10)	1.400(4)	C(6)-C(7)	1.382(4)	C(7)-
C(8)	1.377(4)	C(8)-C(9)	1.382(4)	C(9)-
C(10)	1.367(4)			

C(11)-O(2)-C(12)	116.5(2)	C(8)-O(3)-C(13)	117.2(2)	N(2)-
N(1)-C(1)	120.9(3)	N(3)-N(2)-N(1)	169.1(4)	C(2)-
C(1)-N(1)	118.1(3)	C(2)-C(1)-C(11)	122.6(3)	N(1)-
C(1)-C(11)	119.3(2)	C(1)-C(2)-C(3)	126.6(3)	C(4)-
C(3)-C(2)	122.8(3)	C(3)-C(4)-C(5)	128.3(3)	C(6)-
C(5)-C(10)	116.7(3)	C(6)-C(5)-C(4)	120.8(3)	C(10)-
C(5)-C(4)	122.5(3)	C(5)-C(6)-C(7)	122.7(3)	C(8)-
C(7)-C(6)	119.2(3)	O(3)-C(8)-C(7)	124.7(3)	O(3)-
C(8)-C(9)	115.7(3)	C(7)-C(8)-C(9)	119.5(3)	C(10)-
C(9)-C(8)	120.6(3)	C(9)-C(10)-C(5)	121.4(3)	O(1)-
C(11)-O(2)	123.5(3)	O(1)-C(11)-C(1)	124.2(3)	O(2)-
C(11)-C(1)	112.3(3)			

Table S41. Torsion angles [°] for azide **1a** ($C_{13}H_{13}N_3O_3$) at 295K (CCDC-2352524).

C(1)-N(1)-N(2)-N(3)	-178.8(18)	N(2)-N(1)-C(1)-C(2)	-174.4(3)	N(2)-
N(1)-C(1)-C(11)	7.3(5)	N(1)-C(1)-C(2)-C(3)	0.3(5)	C(11)-
C(1)-C(2)-C(3)	178.5(3)	C(1)-C(2)-C(3)-C(4)	176.8(3)	C(2)-
C(3)-C(4)-C(5)	-177.9(3)	C(3)-C(4)-C(5)-C(6)	171.0(3)	C(3)-
C(4)-C(5)-C(10)	-8.2(5)	C(10)-C(5)-C(6)-C(7)	0.3(5)	C(4)-
C(5)-C(6)-C(7)	-178.9(3)	C(5)-C(6)-C(7)-C(8)	0.0(5)	C(13)-
O(3)-C(8)-C(7)	9.0(5)	C(13)-O(3)-C(8)-C(9)	-171.2(3)	C(6)-
C(7)-C(8)-O(3)	179.4(3)	C(6)-C(7)-C(8)-C(9)	-0.4(5)	O(3)-
C(8)-C(9)-C(10)	-179.4(3)	C(7)-C(8)-C(9)-C(10)	0.4(5)	C(8)-
C(9)-C(10)-C(5)	0.0(4)	C(6)-C(5)-C(10)-C(9)	-0.3(4)	C(4)-
C(5)-C(10)-C(9)	178.9(3)	C(12)-O(2)-C(11)-O(1)	2.8(4)	C(12)-
O(2)-C(11)-C(1)	-177.1(2)	C(2)-C(1)-C(11)-O(1)	-174.7(3)	N(1)-
C(1)-C(11)-O(1)	3.5(5)	C(2)-C(1)-C(11)-O(2)	5.2(4)	N(1)-
C(1)-C(11)-O(2)	-176.6(3)			

Table S42. Anisotropic displacement parameters [Å^{2}x10^3] for azide **1a** ($C_{13}H_{13}N_3O_3$) at 295K (CCDC-2352524).. The anisotropic displacement factor exponent takes the form: -2π2[h 2a*2U11+... +2hka*b*U12]

	U_{11}	U_{22}	U_{33}	U_{23}	U_{13}	U_{12}
O(1)	84(2)	97(2)	57(1)	-6(1)	14(1)	11(1)
O(2)	64(1)	74(2)	66(1)	2(1)	8(1)	13(1)
O(3)	90(2)	104(2)	50(1)	-1(1)	7(1)	32(2)
N(1)	72(2)	102(2)	42(1)	-1(2)	5(1)	12(2)
N(2)	61(2)	85(2)	68(2)	0(2)	-2(2)	0(2)
N(3)	95(2)	128(3)	66(2)	13(2)	2(2)	7(2)
C(1)	53(2)	55(2)	54(2)	-2(2)	0(2)	-2(2)
C(2)	58(2)	55(2)	54(2)	0(2)	4(2)	1(2)
C(3)	56(2)	58(2)	55(2)	-2(2)	4(2)	1(2)
C(4)	55(2)	62(2)	58(2)	1(2)	2(2)	-2(2)
C(5)	53(2)	54(2)	50(2)	0(2)	0(2)	-2(2)
C(6)	60(2)	82(2)	52(2)	-1(2)	-6(2)	7(2)
C(7)	73(2)	81(2)	45(2)	-4(2)	-4(2)	5(2)
C(8)	63(2)	62(2)	45(2)	-1(2)	2(2)	5(2)
C(9)	58(2)	64(2)	52(2)	4(2)	-1(2)	4(2)
C(10)	63(2)	65(2)	38(2)	2(2)	-1(2)	-6(2)
C(11)	60(2)	57(2)	58(2)	-3(2)	10(2)	-7(2)
C(12)	79(2)	90(3)	93(3)	-4(2)	17(2)	30(2)
C(13)	114(3)	132(4)	48(2)	1(2)	10(2)	34(3)

9.5. X-ray Structure of Photoproduct 2a

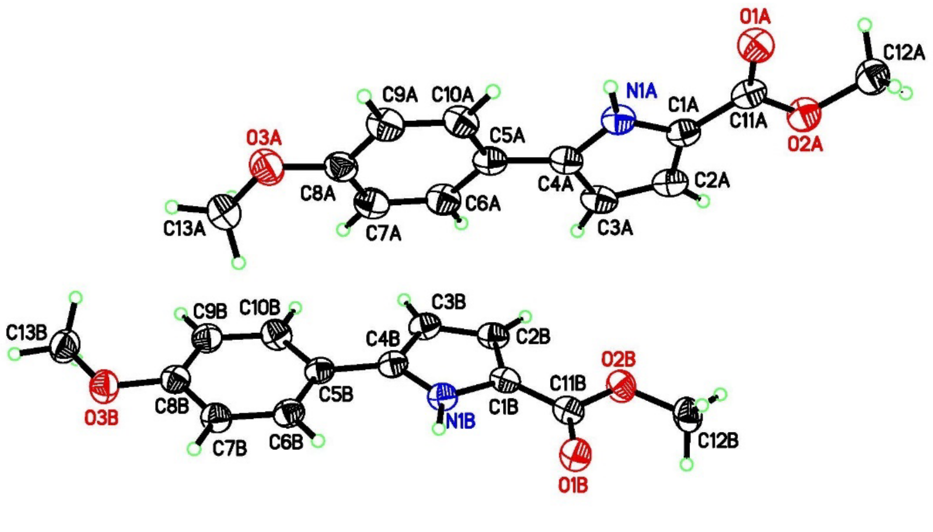

Figure S56. Crystal Structure of Photoproduct **2a** (CCDC-2352527)

Crystals were obtained from ethyl acetate-hexane (1:1). For X-ray examination and data collection, a pale yellow block-shaped crystal, approximate dimensions 0.190 x 0.150 x 0.130 mm, was mounted in a loop with Paratone-N oil and transferred to the goniostat bathed in a cold stream.

Intensity data were collected at 150K on a Bruker APEX-II CCD diffractometer using Mo Kα radiation, λ=0.71073Å (TRIUMPH curved-graphite monochromator). The data frames were processed using the program SAINT. The data were corrected for decay, Lorentz and polarization effects as well as absorption and beam corrections.

The structure was solved by a combination of direct methods and the difference Fourier technique as implemented in the SHELX suite of programs, and refined by full-matrix least squares on F^2 for reflections out to 0.85Å resolution (data intensity declined rapidly beyond 0.95Å resolution). Non-hydrogen atoms were refined with anisotropic displacement parameters. The N-bound H-atom positions were located directly from the difference map and refined. The remaining H-atom positions were calculated and treated with a riding model. All H-atom isotropic displacement parameters were defined as a*U_{eq} of the adjacent atom (a=1.5 for methyl and 1.2 for all others). The refinement converged with crystallographic agreement factors of R1=5.74%, wR2=14.47% for 3053 reflections with I>2σ(I) (R1=7.62%, wR2=15.31% for all data) and 317 variable parameters.

Table S43. Crystal data and structure refinement for **2a** $C_{13}H_{13}NO_3$ (CCDC-2352527).

Empirical formula	$C_{13}H_{13}NO_3$	
Formula weight	231.24	
Temperature	150(2) K	
Wavelength	0.71073 Å	
Crystal system	Monoclinic	
Space group	$P2_1/c$	
Unit cell dimensions	a = 15.838(3) Å	α = 90°
	b = 19.892(4) Å	β = 92.814(3)°
	c = 7.3156(14) Å	γ = 90°
Volume	2301.9(8) Å^3	
Z	8	
Density (calculated)	1.334 Mg/m^3	
Absorption coefficient	0.095 mm^{-1}	
F(000)	976	
Crystal size	0.190 x 0.150 x 0.130 mm^3	
θ range for data collection	1.287 to 24.713°	
Index ranges	-18 ≤ h ≤ 18, -23 ≤ k ≤ 23, -8 ≤ l ≤ 8	
Reflections collected	36351	
Independent reflections	3908 [R_{int} = 0.0498]	
Completeness to θ = 24.713°	99.6 %	
Absorption correction	Semi-empirical from equivalents	
Max. and min. transmission	0.862 and 0.766	
Refinement method	Full-matrix least-squares on F^2	
Data / restraints / parameters	3908 / 0 / 317	
Goodness-of-fit on F^2	1.190	
Final R indices [I>2σ(I)]	R1 = 0.0574, wR2 = 0.1447	
R indices (all data)	R1 = 0.0762, wR2 = 0.1531	
Largest diff. peak and hole	0.276 and -0.215 eÅ^{-3}	

Table S44. Atomic coordinates [x10^4] and equivalent isotropic displacement parameters [$Å^2$x10^3] for **2a** $C_{13}H_{13}NO_3$ (CCDC-2352527). U(eq) is defined as one third of the trace of the orthogonalized Uij tensor.

	x	y	z	U(eq)
O(1A)	71(1)	5778(1)	10961(3)	37(1)
O(2A)	727(1)	6783(1)	10834(3)	38(1)
O(3A)	1425(2)	3211(1)	1220(3)	43(1)
N(1A)	840(2)	5416(1)	7634(4)	32(1)
C(1A)	953(2)	6021(2)	8528(4)	32(1)
C(2A)	1478(2)	6407(2)	7500(5)	35(1)
C(3A)	1676(2)	6033(2)	5966(5)	36(1)
C(4A)	1266(2)	5414(2)	6058(4)	31(1)
C(5A)	1291(2)	4847(2)	4784(4)	32(1)
C(6A)	1700(2)	4910(2)	3160(5)	38(1)
C(7A)	1760(2)	4385(2)	1939(5)	40(1)
C(8A)	1399(2)	3768(2)	2329(5)	35(1)
C(9A)	976(2)	3693(2)	3933(5)	38(1)
C(10A)	921(2)	4224(2)	5143(5)	35(1)
C(11A)	540(2)	6159(2)	10195(4)	32(1)
C(12A)	319(2)	6977(2)	12467(5)	43(1)
C(13A)	1900(2)	3271(2)	-382(5)	47(1)
O(1B)	4760(1)	5764(1)	6016(3)	35(1)
O(2B)	3944(1)	6693(1)	5654(3)	37(1)
O(3B)	3948(1)	3131(1)	-4177(3)	37(1)
N(1B)	4074(2)	5313(1)	2502(4)	29(1)
C(1B)	3866(2)	5904(1)	3346(4)	30(1)
C(2B)	3303(2)	6246(2)	2166(4)	33(1)
C(3B)	3180(2)	5854(2)	584(4)	34(1)
C(4B)	3670(2)	5276(1)	810(4)	29(1)
C(5B)	3751(2)	4719(1)	-477(4)	29(1)
C(6B)	4268(2)	4159(2)	-110(4)	32(1)
C(7B)	4321(2)	3642(2)	-1365(4)	33(1)
C(8B)	3858(2)	3667(2)	-3021(4)	31(1)
C(9B)	3341(2)	4217(2)	-3423(4)	36(1)
C(10B)	3294(2)	4730(2)	-2157(4)	36(1)
C(11B)	4239(2)	6091(1)	5107(4)	31(1)
C(12B)	4296(2)	6938(2)	7383(5)	40(1)
C(13B)	3448(2)	3144(2)	-5862(5)	43(1)
H(1)	520(20)	5103(18)	7940(40)	38
H(2A)	1671	6849	7787	42
H(3A)	2027	6174	5024	43
H(6A)	1948	5330	2878	45
H(7A)	2047	4445	840	48
H(9A)	722	3274	4202	46
H(10A)	627	4166	6233	42
H(12A)	459	7446	12764	64
H(12B)	514	6686	13483	64
H(12C)	-295	6932	12266	64
H(13A)	1858	2851	-1083	70
H(13B)	2494	3360	-25	70
H(13C)	1674	3642	-1139	70
H(2)	4450(20)	5047(17)	3000(40)	35
H(2B)	3048	6668	2391	40
H(3B)	2826	5964	-459	40
H(6B)	4588	4134	1021	39
H(7B)	4677	3268	-1089	39
H(9B)	3023	4240	-4556	43
H(10B)	2939	5104	-2442	43
H(12D)	4047	7375	7651	60
H(12G)	4910	6986	7320	60
H(12E)	4171	6618	8353	60
H(13G)	3547	2731	-6552	64
H(13D)	3608	3534	-6585	64
H(13E)	2848	3175	-5601	64

Table S45. Bond lengths [Å] and angles [°] for **2a** $C_{13}H_{13}NO_3$ (CCDC-2352527).

O(1A)-C(11A)	1.217(4)	O(2A)-C(11A)	1.353(4)
O(2A)-C(12A)	1.439(4)	O(3A)-C(8A)	1.374(4)
O(3A)-C(13A)	1.428(4)	N(1A)-C(4A)	1.364(4)
N(1A)-C(1A)	1.378(4)	C(1A)-C(2A)	1.382(4)
C(1A)-C(11A)	1.438(5)	C(2A)-C(3A)	1.395(5)
C(3A)-C(4A)	1.395(4)	C(4A)-C(5A)	1.465(4)
C(5A)-C(6A)	1.386(4)	C(5A)-C(10A)	1.400(4)
C(6A)-C(7A)	1.380(5)	C(7A)-C(8A)	1.390(5)
C(8A)-C(9A)	1.387(5)	C(9A)-C(10A)	1.384(5)
O(1B)-C(11B)	1.221(4)	O(2B)-C(11B)	1.353(4)
O(2B)-C(12B)	1.441(4)	O(3B)-C(8B)	1.372(4)
O(3B)-C(13B)	1.431(4)	N(1B)-C(4B)	1.367(4)
N(1B)-C(1B)	1.374(4)	C(1B)-C(2B)	1.388(4)
C(1B)-C(11B)	1.440(4)	C(2B)-C(3B)	1.401(4)
C(3B)-C(4B)	1.392(4)	C(4B)-C(5B)	1.464(4)
C(5B)-C(10B)	1.395(4)	C(5B)-C(6B)	1.400(4)
C(6B)-C(7B)	1.384(4)	C(7B)-C(8B)	1.386(4)
C(8B)-C(9B)	1.389(4)	C(9B)-C(10B)	1.383(4)

C(11A)-O(2A)-C(12A)	115.7(2)	C(8A)-O(3A)-C(13A)	116.7(3)
C(4A)-N(1A)-C(1A)	110.2(3)	N(1A)-C(1A)-C(2A)	107.2(3)
N(1A)-C(1A)-C(11A)	121.1(3)	C(2A)-C(1A)-C(11A)	131.8(3)
C(1A)-C(2A)-C(3A)	107.8(3)	C(4A)-C(3A)-C(2A)	108.0(3)
N(1A)-C(4A)-C(3A)	106.8(3)	N(1A)-C(4A)-C(5A)	124.7(3)
C(3A)-C(4A)-C(5A)	128.4(3)	C(6A)-C(5A)-C(10A)	117.3(3)
C(6A)-C(5A)-C(4A)	120.3(3)	C(10A)-C(5A)-C(4A)	122.5(3)
C(7A)-C(6A)-C(5A)	122.4(3)	C(6A)-C(7A)-C(8A)	119.5(3)
O(3A)-C(8A)-C(9A)	116.3(3)	O(3A)-C(8A)-C(7A)	124.4(3)
C(9A)-C(8A)-C(7A)	119.3(3)	C(10A)-C(9A)-C(8A)	120.5(3)
C(9A)-C(10A)-C(5A)	121.0(3)	O(1A)-C(11A)-O(2A)	122.6(3)
O(1A)-C(11A)-C(1A)	125.8(3)	O(2A)-C(11A)-C(1A)	111.6(3)
C(11B)-O(2B)-C(12B)	115.8(2)	C(8B)-O(3B)-C(13B)	116.6(2)
C(4B)-N(1B)-C(1B)	109.9(3)	N(1B)-C(1B)-C(2B)	107.5(3)
N(1B)-C(1B)-C(11B)	121.8(3)	C(2B)-C(1B)-C(11B)	130.7(3)
C(1B)-C(2B)-C(3B)	107.4(3)	C(4B)-C(3B)-C(2B)	107.9(3)
N(1B)-C(4B)-C(3B)	107.2(3)	N(1B)-C(4B)-C(5B)	124.8(3)
C(3B)-C(4B)-C(5B)	128.0(3)	C(10B)-C(5B)-C(6B)	117.1(3)
C(10B)-C(5B)-C(4B)	119.7(3)	C(6B)-C(5B)-C(4B)	123.2(3)
C(7B)-C(6B)-C(5B)	121.2(3)	C(6B)-C(7B)-C(8B)	120.5(3)
O(3B)-C(8B)-C(7B)	116.4(3)	O(3B)-C(8B)-C(9B)	124.0(3)
C(7B)-C(8B)-C(9B)	119.6(3)	C(10B)-C(9B)-C(8B)	119.4(3)
C(9B)-C(10B)-C(5B)	122.4(3)	O(1B)-C(11B)-O(2B)	122.9(3)
O(1B)-C(11B)-C(1B)	125.9(3)	O(2B)-C(11B)-C(1B)	111.2(3)

Table S46. Anisotropic displacement parameters [$Å^2x10^3$] for **2a** $C_{13}H_{13}NO_3$ (CCDC-2352527). The anisotropic displacement factor exponent takes the form: $-2\pi^2[h^2a^{*2}U_{11}+... +2hka^*b^*U_{12}]$

	U_{11}	U_{22}	U_{33}	U_{23}	U_{13}	U_{12}
O(1A)	41(1)	27(1)	43(1)	5(1)	6(1)	-5(1)
O(2A)	44(1)	27(1)	45(1)	0(1)	5(1)	-9(1)
O(3A)	45(1)	38(1)	48(1)	-3(1)	11(1)	0(1)
N(1A)	31(1)	24(1)	39(2)	6(1)	3(1)	-3(1)
C(1A)	27(2)	27(2)	40(2)	6(1)	-2(1)	1(1)
C(2A)	28(2)	28(2)	49(2)	7(2)	1(1)	0(1)
C(3A)	30(2)	29(2)	49(2)	11(2)	7(1)	0(1)
C(4A)	28(2)	28(2)	36(2)	8(1)	2(1)	4(1)
C(5A)	27(2)	29(2)	38(2)	7(1)	-1(1)	4(1)
C(6A)	37(2)	33(2)	44(2)	6(2)	6(2)	-3(1)
C(7A)	35(2)	43(2)	41(2)	4(2)	7(2)	-1(2)
C(8A)	29(2)	35(2)	42(2)	1(2)	1(1)	5(1)
C(9A)	38(2)	26(2)	52(2)	7(2)	8(2)	-1(1)
C(10A)	35(2)	32(2)	40(2)	6(1)	8(1)	2(1)
C(11A)	30(2)	25(2)	42(2)	6(1)	-4(1)	-1(1)
C(12A)	49(2)	33(2)	47(2)	-5(2)	6(2)	-9(2)
C(13A)	44(2)	50(2)	46(2)	-7(2)	7(2)	1(2)
O(1B)	40(1)	26(1)	37(1)	2(1)	-2(1)	5(1)
O(2B)	41(1)	27(1)	44(1)	-4(1)	0(1)	7(1)
O(3B)	42(1)	32(1)	37(1)	-3(1)	-1(1)	2(1)
N(1B)	29(1)	22(1)	36(2)	4(1)	1(1)	2(1)
C(1B)	26(2)	24(2)	39(2)	4(1)	6(1)	1(1)
C(2B)	29(2)	27(2)	44(2)	4(1)	3(1)	2(1)
C(3B)	30(2)	30(2)	40(2)	6(1)	-2(1)	0(1)
C(4B)	28(2)	26(2)	33(2)	5(1)	1(1)	-3(1)
C(5B)	27(2)	27(2)	33(2)	4(1)	2(1)	-5(1)
C(6B)	30(2)	31(2)	35(2)	3(1)	-2(1)	-1(1)
C(7B)	32(2)	27(2)	39(2)	2(1)	2(1)	2(1)
C(8B)	31(2)	28(2)	35(2)	3(1)	4(1)	-4(1)
C(9B)	34(2)	38(2)	36(2)	0(1)	-2(1)	2(1)
C(10B)	38(2)	34(2)	37(2)	5(1)	-1(2)	7(1)
C(11B)	31(2)	23(2)	39(2)	5(1)	6(1)	1(1)
C(12B)	48(2)	30(2)	41(2)	-6(1)	2(2)	8(2)
C(13B)	45(2)	43(2)	40(2)	-6(2)	-6(2)	1(2)

Table S47. Torsion angles [°] for **2a** $C_{13}H_{13}NO_3$ (CCDC-2352527).

C(4A)-N(1A)-C(1A)-C(2A)	-1.1(3)	C(4A)-N(1A)-C(1A)-C(11A)	177.6(3)
N(1A)-C(1A)-C(2A)-C(3A)	0.6(3)	C(11A)-C(1A)-C(2A)-C(3A)	-177.9(3)
C(1A)-C(2A)-C(3A)-C(4A)	0.1(4)	C(1A)-N(1A)-C(4A)-C(3A)	1.1(3)
C(1A)-N(1A)-C(4A)-C(5A)	179.2(3)	C(2A)-C(3A)-C(4A)-N(1A)	-0.7(3)
C(2A)-C(3A)-C(4A)-C(5A)	-178.7(3)	N(1A)-C(4A)-C(5A)-C(6A)	176.0(3)
C(3A)-C(4A)-C(5A)-C(6A)	-6.3(5)	N(1A)-C(4A)-C(5A)-C(10A)	-4.7(5)
C(3A)-C(4A)-C(5A)-C(10A)	172.9(3)	C(10A)-C(5A)-C(6A)-C(7A)	-1.1(5)
C(4A)-C(5A)-C(6A)-C(7A)	178.3(3)	C(5A)-C(6A)-C(7A)-C(8A)	0.2(5)
C(13A)-O(3A)-C(8A)-C(9A)	-176.5(3)	C(13A)-O(3A)-C(8A)-C(7A)	3.8(5)
C(6A)-C(7A)-C(8A)-O(3A)	-179.6(3)	C(6A)-C(7A)-C(8A)-C(9A)	0.7(5)
O(3A)-C(8A)-C(9A)-C(10A)	179.6(3)	C(7A)-C(8A)-C(9A)-C(10A)	-0.6(5)
C(8A)-C(9A)-C(10A)-C(5A)	-0.2(5)	C(6A)-C(5A)-C(10A)-C(9A)	1.1(5)
C(4A)-C(5A)-C(10A)-C(9A)	-178.2(3)	C(12A)-O(2A)-C(11A)-O(1A)	-1.4(4)
C(12A)-O(2A)-C(11A)-C(1A)	177.9(3)	N(1A)-C(1A)-C(11A)-O(1A)	0.0(5)
C(2A)-C(1A)-C(11A)-O(1A)	178.3(3)	N(1A)-C(1A)-C(11A)-O(2A)	-179.2(3)
C(2A)-C(1A)-C(11A)-O(2A)	-0.9(5)	C(4B)-N(1B)-C(1B)-C(2B)	0.9(3)
C(4B)-N(1B)-C(1B)-C(11B)	-176.5(3)	N(1B)-C(1B)-C(2B)-C(3B)	-0.5(3)
C(11B)-C(1B)-C(2B)-C(3B)	176.7(3)	C(1B)-C(2B)-C(3B)-C(4B)	-0.1(3)
C(1B)-N(1B)-C(4B)-C(3B)	-1.0(3)	C(1B)-N(1B)-C(4B)-C(5B)	179.5(3)
C(2B)-C(3B)-C(4B)-N(1B)	0.6(3)	C(2B)-C(3B)-C(4B)-C(5B)	-179.8(3)
N(1B)-C(4B)-C(5B)-C(10B)	178.8(3)	C(3B)-C(4B)-C(5B)-C(10B)	-0.6(5)
N(1B)-C(4B)-C(5B)-C(6B)	-0.8(5)	C(3B)-C(4B)-C(5B)-C(6B)	179.7(3)
C(10B)-C(5B)-C(6B)-C(7B)	0.0(4)	C(4B)-C(5B)-C(6B)-C(7B)	179.7(3)
C(5B)-C(6B)-C(7B)-C(8B)	-0.2(5)	C(13B)-O(3B)-C(8B)-C(7B)	177.9(3)
C(13B)-O(3B)-C(8B)-C(9B)	-2.1(4)	C(6B)-C(7B)-C(8B)-O(3B)	-179.7(3)
C(6B)-C(7B)-C(8B)-C(9B)	0.3(5)	O(3B)-C(8B)-C(9B)-C(10B)	179.8(3)
C(7B)-C(8B)-C(9B)-C(10B)	-0.2(5)	C(8B)-C(9B)-C(10B)-C(5B)	0.1(5)
C(6B)-C(5B)-C(10B)-C(9B)	0.0(5)	C(4B)-C(5B)-C(10B)-C(9B)	-179.6(3)
C(12B)-O(2B)-C(11B)-O(1B)	1.0(4)	C(12B)-O(2B)-C(11B)-C(1B)	-178.3(3)
N(1B)-C(1B)-C(11B)-O(1B)	0.2(5)	C(2B)-C(1B)-C(11B)-O(1B)	-176.7(3)
N(1B)-C(1B)-C(11B)-O(2B)	179.4(3)	C(2B)-C(1B)-C(11B)-O(2B)	2.6(5)

9.6. X-ray Structure of Photoproduct 2b

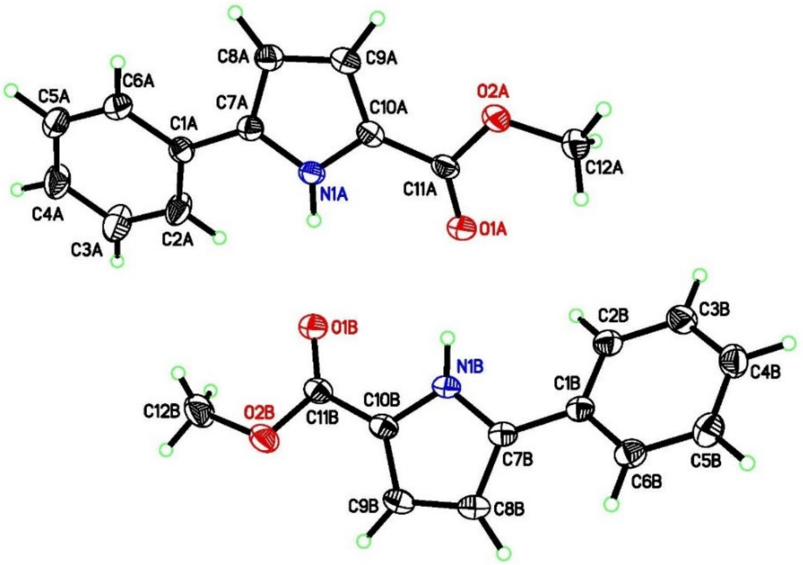

Figure S57. Crystal structure of photoproduct **2b** (CCDC-2352528)

For X-ray examination and data collection, a suitable **colorless** tablet-shaped crystal, approximate dimensions 0.180 x 0.124 x 0.099 mm, was mounted in a loop with Paratone-N oil and transferred to the goniostat bathed in a cold stream.

Intensity data were collected at 150K on a Bruker D8 Venture Mo-I[illegible]S Photon-II diffractometer, λ=0.71073Å. For data collection frames were measured in shutterless mode. The data frames were processed using the program SAINT. The data were corrected for decay, Lorentz and polarization effects as well as absorption and beam corrections based on the numerical technique.

The structure was solved by a combination of direct methods in SHELXTL and the difference Fourier technique and refined by full-matrix least squares on F^2 for reflections out to 0.83Å resolution. Non-hydrogen atoms were refined with anisotropic displacement parameters. H-atoms were located directly from the difference map and the coordinates refined. The H-atom isotropic displacement parameters were defined as a*U_{eq} (a=1.5 for methyl and 1.2 for all others) of the adjacent atom. The refinement converged with crystallographic agreement factors of R1=3.73%, wR2=8.63% for 3097 reflections with I>2σ(I) (R1=4.71%, wR2=9.33% for all data) and 337 variable parameters.

Table S48. Crystal data and structure refinement for **2b** $C_{12}H_{11}NO_2$ (CCDC-2352528)

Empirical formula	$C_{12}H_{11}NO_2$	
Formula weight	201.22	
Temperature	150(2) K	
Wavelength	0.71073 Å	
Crystal system	Monoclinic	
Space group	$P2_1/c$	
Unit cell dimensions	a = 9.9019(3) Å	α = 90°
	b = 20.0776(5) Å	β = 104.4454(9)°
	c = 10.4531(3) Å	γ = 90°
Volume	2012.44(10) $Å^3$	
Z	8	
Density (calculated)	1.328 Mg/m^3	
Absorption coefficient	0.091 mm^{-1}	
F(000)	848	
Crystal size	0.180 x 0.124 x 0.099 mm^3	
θ range for data collection	2.354 to 25.348°	
Index ranges	-11 ≤ h ≤ 11, -24 ≤ k ≤ 24, -12 ≤ l ≤ 12	
Reflections collected	19169	
Independent reflections	3674 [R_{int} = 0.0345]	
Completeness to θ = 25.242°	99.9 %	
Absorption correction	Numerical	
Max. and min. transmission	0.862 and 0.780	
Refinement method	Full-matrix least-squares on F^2	
Data / restraints / parameters	3674 / 0 / 337	
Goodness-of-fit on F^2	1.046	
Final R indices [I>2σ(I)]	R1 = 0.0373, wR2 = 0.0863	
R indices (all data)	R1 = 0.0471, wR2 = 0.0933	
Largest diff. peak and hole	0.151 and -0.234 $eÅ^{-3}$	

Table S49. Atomic coordinates [x10^4] and equivalent isotropic displacement parameters [Å^{2}x10^3] for **2b** $C_{12}H_{11}NO_2$ (CCDC-2352528). U(eq) is defined as one third of the trace of the orthogonalized Uij tensor.

	x	y	z	U(eq)
O(1A)	2701(1)	4283(1)	7177(1)	32(1)
O(2A)	3265(1)	4414(1)	5230(1)	36(1)
N(1A)	1125(1)	5496(1)	6611(1)	25(1)
C(1A)	-386(1)	6485(1)	6649(1)	25(1)
C(2A)	-1009(2)	6252(1)	7621(2)	30(1)
C(3A)	-1784(2)	6675(1)	8202(2)	34(1)
C(4A)	-1954(2)	7336(1)	7829(2)	35(1)
C(5A)	-1355(2)	7574(1)	6856(2)	35(1)
C(6A)	-574(2)	7154(1)	6274(2)	30(1)
C(7A)	442(1)	6049(1)	6016(1)	24(1)
C(8A)	680(2)	6090(1)	4766(2)	29(1)
C(9A)	1534(2)	5558(1)	4617(2)	29(1)
C(10A)	1802(2)	5194(1)	5770(1)	26(1)
C(11A)	2613(2)	4592(1)	6156(1)	27(1)
C(12A)	4069(2)	3806(1)	5461(2)	45(1)
O(1B)	1475(1)	5269(1)	-615(1)	33(1)
O(2B)	2012(1)	5639(1)	1488(1)	35(1)
N(1B)	3831(1)	4386(1)	124(1)	24(1)
C(1B)	5607(2)	3486(1)	260(1)	25(1)
C(2B)	5021(2)	3209(1)	-970(2)	39(1)
C(3B)	5662(2)	2681(1)	-1441(2)	49(1)
C(4B)	6895(2)	2421(1)	-690(2)	41(1)
C(5B)	7498(2)	2697(1)	528(2)	37(1)
C(6B)	6871(2)	3226(1)	993(2)	32(1)
C(7B)	4926(1)	4015(1)	829(1)	25(1)
C(8B)	5176(2)	4213(1)	2137(2)	29(1)
C(9B)	4225(2)	4715(1)	2223(2)	30(1)
C(10B)	3401(2)	4820(1)	965(1)	25(1)
C(11B)	2217(2)	5254(1)	505(1)	27(1)
C(12B)	828(2)	6083(1)	1162(2)	40(1)
H(1)	1207(16)	5381(8)	7437(17)	30
H(2A)	-908(17)	5785(9)	7865(16)	36
H(3A)	-2212(18)	6509(8)	8874(18)	41
H(4A)	-2481(18)	7637(9)	8257(18)	42
H(5A)	-1462(17)	8057(9)	6589(17)	42
H(6A)	-151(17)	7333(8)	5575(17)	36
H(8A)	263(17)	6427(8)	4131(17)	34
H(9A)	1869(17)	5445(8)	3845(17)	35
H(12A)	4500(20)	3767(10)	4720(20)	67
H(12B)	3470(20)	3416(11)	5500(20)	67
H(12C)	4800(20)	3832(11)	6260(20)	67
H(2)	3469(16)	4372(8)	-752(17)	29
H(2B)	4136(19)	3378(9)	-1494(18)	47
H(3B)	5240(20)	2502(10)	-2310(20)	59
H(4B)	7336(19)	2037(9)	-1000(18)	49
H(5B)	8380(19)	2509(9)	1089(18)	45
H(6B)	7298(17)	3427(8)	1865(17)	38
H(8B)	5870(17)	4022(8)	2856(17)	34
H(9B)	4121(17)	4941(8)	3002(17)	35
H(12D)	780(20)	6318(10)	330(20)	60
H(12E)	1010(20)	6393(10)	1910(20)	60
H(12F)	-20(20)	5834(10)	1080(20)	60

Table S50. Bond lengths [Å] and angles [°] for **2b** $C_{12}H_{11}NO_2$ (CCDC-2352528).

O(1A)-C(11A)	1.2189(17)	O(2A)-C(11A)	1.3396(17)
O(2A)-C(12A)	1.444(2)	N(1A)-C(7A)	1.3656(19)
N(1A)-C(10A)	1.3732(18)	C(1A)-C(2A)	1.395(2)
C(1A)-C(6A)	1.400(2)	C(1A)-C(7A)	1.465(2)
C(2A)-C(3A)	1.382(2)	C(3A)-C(4A)	1.381(2)
C(4A)-C(5A)	1.384(2)	C(5A)-C(6A)	1.384(2)
C(7A)-C(8A)	1.386(2)	C(8A)-C(9A)	1.396(2)
C(9A)-C(10A)	1.378(2)	C(10A)-C(11A)	1.451(2)
O(1B)-C(11B)	1.2175(18)	O(2B)-C(11B)	1.3405(17)
O(2B)-C(12B)	1.445(2)	N(1B)-C(7B)	1.3691(19)
N(1B)-C(10B)	1.3786(18)	C(1B)-C(2B)	1.388(2)
C(1B)-C(6B)	1.395(2)	C(1B)-C(7B)	1.462(2)
C(2B)-C(3B)	1.387(2)	C(3B)-C(4B)	1.379(2)
C(4B)-C(5B)	1.380(2)	C(5B)-C(6B)	1.380(2)
C(7B)-C(8B)	1.385(2)	C(8B)-C(9B)	1.397(2)
C(9B)-C(10B)	1.380(2)	C(10B)-C(11B)	1.443(2)

C(11A)-O(2A)-C(12A)	116.73(13)	C(7A)-N(1A)-C(10A)	109.45(12)
C(2A)-C(1A)-C(6A)	118.28(14)	C(2A)-C(1A)-C(7A)	121.91(13)
C(6A)-C(1A)-C(7A)	119.81(13)	C(3A)-C(2A)-C(1A)	120.63(14)
C(4A)-C(3A)-C(2A)	120.50(15)	C(3A)-C(4A)-C(5A)	119.72(15)
C(6A)-C(5A)-C(4A)	120.08(15)	C(5A)-C(6A)-C(1A)	120.79(14)
N(1A)-C(7A)-C(8A)	107.27(13)	N(1A)-C(7A)-C(1A)	123.28(13)
C(8A)-C(7A)-C(1A)	129.45(13)	C(7A)-C(8A)-C(9A)	108.03(14)
C(10A)-C(9A)-C(8A)	107.35(13)	N(1A)-C(10A)-C(9A)	107.89(13)
N(1A)-C(10A)-C(11A)	120.98(13)	C(9A)-C(10A)-C(11A)	131.12(14)
O(1A)-C(11A)-O(2A)	123.71(14)	O(1A)-C(11A)-C(10A)	125.55(13)
O(2A)-C(11A)-C(10A)	110.75(12)	C(11B)-O(2B)-C(12B)	116.35(13)
C(7B)-N(1B)-C(10B)	109.22(12)	C(2B)-C(1B)-C(6B)	117.95(14)
C(2B)-C(1B)-C(7B)	122.67(14)	C(6B)-C(1B)-C(7B)	119.32(14)
C(3B)-C(2B)-C(1B)	120.82(16)	C(4B)-C(3B)-C(2B)	120.44(17)
C(3B)-C(4B)-C(5B)	119.37(16)	C(6B)-C(5B)-C(4B)	120.33(16)
C(5B)-C(6B)-C(1B)	121.06(15)	N(1B)-C(7B)-C(8B)	107.36(13)
N(1B)-C(7B)-C(1B)	124.03(13)	C(8B)-C(7B)-C(1B)	128.51(14)
C(7B)-C(8B)-C(9B)	108.21(14)	C(10B)-C(9B)-C(8B)	107.26(13)
N(1B)-C(10B)-C(9B)	107.94(13)	N(1B)-C(10B)-C(11B)	121.57(13)
C(9B)-C(10B)-C(11B)	130.36(13)	O(1B)-C(11B)-O(2B)	123.53(14)
O(1B)-C(11B)-C(10B)	125.46(13)	O(2B)-C(11B)-C(10B)	110.99(13)

Table S51. Anisotropic displacement parameters [Å^{2}x10^3] for **2b** $C_{12}H_{11}NO_2$ (CCDC-2352528). The anisotropic displacement factor exponent takes the form: $-2\pi^2[h^2a^{*2}U_{11}+ ... +2hka^*b^*U_{12}]$

	U_{11}	U_{22}	U_{33}	U_{23}	U_{13}	U_{12}
O(1A)	38(1)	34(1)	25(1)	1(1)	9(1)	4(1)
O(2A)	46(1)	34(1)	31(1)	-1(1)	17(1)	7(1)
N(1A)	29(1)	28(1)	19(1)	0(1)	6(1)	-3(1)
C(1A)	21(1)	29(1)	22(1)	-1(1)	2(1)	-4(1)
C(2A)	31(1)	30(1)	30(1)	5(1)	8(1)	1(1)
C(3A)	31(1)	42(1)	31(1)	4(1)	11(1)	2(1)
C(4A)	31(1)	39(1)	36(1)	-4(1)	8(1)	4(1)
C(5A)	34(1)	29(1)	40(1)	2(1)	7(1)	1(1)
C(6A)	30(1)	31(1)	30(1)	3(1)	7(1)	-3(1)
C(7A)	25(1)	24(1)	22(1)	0(1)	3(1)	-5(1)
C(8A)	34(1)	29(1)	22(1)	3(1)	5(1)	-3(1)
C(9A)	34(1)	33(1)	22(1)	-3(1)	9(1)	-6(1)
C(10A)	27(1)	29(1)	21(1)	-4(1)	5(1)	-6(1)
C(11A)	29(1)	31(1)	22(1)	-5(1)	7(1)	-5(1)
C(12A)	57(1)	38(1)	46(1)	0(1)	26(1)	12(1)
O(1B)	42(1)	36(1)	22(1)	2(1)	10(1)	9(1)
O(2B)	48(1)	35(1)	25(1)	-1(1)	13(1)	14(1)
N(1B)	28(1)	26(1)	20(1)	0(1)	7(1)	0(1)
C(1B)	25(1)	25(1)	27(1)	1(1)	8(1)	-2(1)
C(2B)	31(1)	42(1)	38(1)	-12(1)	-3(1)	11(1)
C(3B)	41(1)	53(1)	44(1)	-24(1)	-6(1)	15(1)
C(4B)	38(1)	40(1)	44(1)	-11(1)	5(1)	11(1)
C(5B)	32(1)	42(1)	35(1)	0(1)	3(1)	10(1)
C(6B)	32(1)	37(1)	25(1)	-2(1)	5(1)	1(1)
C(7B)	24(1)	24(1)	26(1)	1(1)	7(1)	-3(1)
C(8B)	29(1)	33(1)	23(1)	1(1)	5(1)	0(1)
C(9B)	34(1)	31(1)	24(1)	-4(1)	10(1)	-2(1)
C(10B)	31(1)	24(1)	23(1)	-1(1)	11(1)	-1(1)
C(11B)	37(1)	26(1)	22(1)	2(1)	15(1)	0(1)
C(12B)	54(1)	37(1)	33(1)	4(1)	16(1)	17(1)

Table S52. Torsion angles [°] for **2b** $C_{12}H_{11}NO_2$ (CCDC-2352528).

C(6A)-C(1A)-C(2A)-C(3A)	-0.5(2)	C(7A)-C(1A)-C(2A)-C(3A)	179.95(14)
C(1A)-C(2A)-C(3A)-C(4A)	0.0(2)	C(2A)-C(3A)-C(4A)-C(5A)	0.6(3)
C(3A)-C(4A)-C(5A)-C(6A)	-0.9(3)	C(4A)-C(5A)-C(6A)-C(1A)	0.4(2)
C(2A)-C(1A)-C(6A)-C(5A)	0.2(2)	C(7A)-C(1A)-C(6A)-C(5A)	179.82(14)
C(10A)-N(1A)-C(7A)-C(8A)	0.80(16)	C(10A)-N(1A)-C(7A)-C(1A)	179.91(13)
C(2A)-C(1A)-C(7A)-N(1A)	-26.5(2)	C(6A)-C(1A)-C(7A)-N(1A)	153.88(14)
C(2A)-C(1A)-C(7A)-C(8A)	152.38(15)	C(6A)-C(1A)-C(7A)-C(8A)	-27.2(2)
N(1A)-C(7A)-C(8A)-C(9A)	-0.73(16)	C(1A)-C(7A)-C(8A)-C(9A)	-179.77(14)
C(7A)-C(8A)-C(9A)-C(10A)	0.39(17)	C(7A)-N(1A)-C(10A)-C(9A)	-0.56(16)
C(7A)-N(1A)-C(10A)-C(11A)	-179.97(12)	C(8A)-C(9A)-C(10A)-N(1A)	0.09(17)
C(8A)-C(9A)-C(10A)-C(11A)	179.42(15)	C(12A)-O(2A)-C(11A)-O(1A)	2.2(2)
C(12A)-O(2A)-C(11A)-C(10A)	-177.62(14)	N(1A)-C(10A)-C(11A)-O(1A)	4.9(2)
C(9A)-C(10A)-C(11A)-O(1A)	-174.39(15)	N(1A)-C(10A)-C(11A)-O(2A)	-175.29(12)
C(9A)-C(10A)-C(11A)-O(2A)	5.5(2)	C(6B)-C(1B)-C(2B)-C(3B)	1.2(3)
C(7B)-C(1B)-C(2B)-C(3B)	-176.23(16)	C(1B)-C(2B)-C(3B)-C(4B)	0.2(3)
C(2B)-C(3B)-C(4B)-C(5B)	-1.0(3)	C(3B)-C(4B)-C(5B)-C(6B)	0.3(3)
C(4B)-C(5B)-C(6B)-C(1B)	1.2(3)	C(2B)-C(1B)-C(6B)-C(5B)	-1.9(2)
C(7B)-C(1B)-C(6B)-C(5B)	175.62(14)	C(10B)-N(1B)-C(7B)-C(8B)	0.77(16)
C(10B)-N(1B)-C(7B)-C(1B)	177.43(12)	C(2B)-C(1B)-C(7B)-N(1B)	-14.8(2)
C(6B)-C(1B)-C(7B)-N(1B)	167.76(13)	C(2B)-C(1B)-C(7B)-C(8B)	161.11(16)
C(6B)-C(1B)-C(7B)-C(8B)	-16.3(2)	N(1B)-C(7B)-C(8B)-C(9B)	-0.50(16)
C(1B)-C(7B)-C(8B)-C(9B)	-176.96(14)	C(7B)-C(8B)-C(9B)-C(10B)	0.05(17)
C(7B)-N(1B)-C(10B)-C(9B)	-0.74(16)	C(7B)-N(1B)-C(10B)-C(11B)	-177.08(12)
C(8B)-C(9B)-C(10B)-N(1B)	0.42(16)	C(8B)-C(9B)-C(10B)-C(11B)	176.32(15)
C(12B)-O(2B)-C(11B)-O(1B)	0.7(2)	C(12B)-O(2B)-C(11B)-C(10B)	-178.23(13)
N(1B)-C(10B)-C(11B)-O(1B)	2.4(2)	C(9B)-C(10B)-C(11B)-O(1B)	-173.03(15)
N(1B)-C(10B)-C(11B)-O(2B)	-178.73(12)	C(9B)-C(10B)-C(11B)-O(2B)	5.8(2)

Additional structure for **2b** (UnsubProd_JK)

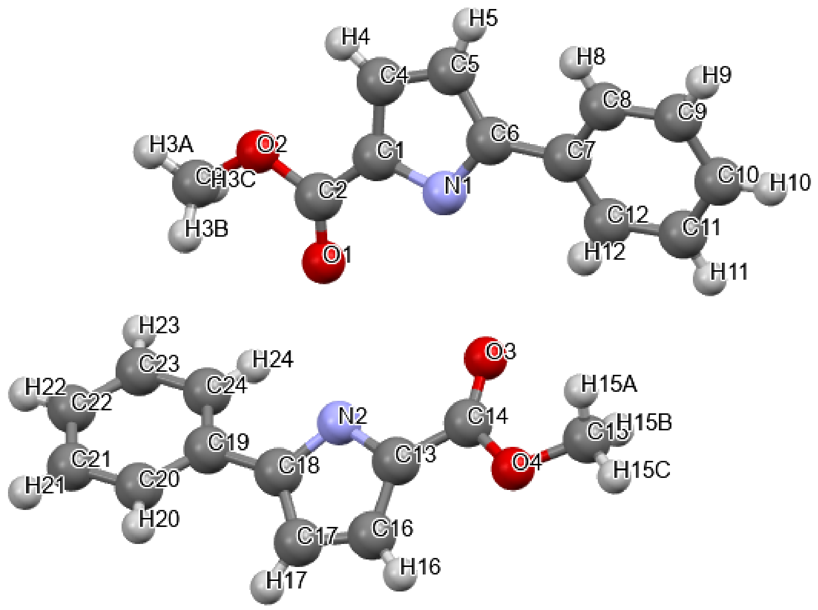

Figure S58. Crystal structure of **2b** (Sjkc23fw20)

Table S53. Crystal data and structure refinement for **2b**.

Identification code	**2b** (UnsubProd_JK)
Empirical formula	$C_{26}H_{22}N_2O_4$
Formula weight	401.94
Temperature/K	150(2)
Crystal system	Monoclinic
Space group	$P2_1/c$
a/Å	9.8829(6)
b/Å	20.0887(12)
c/Å	10.4493(7)
α/°	90
β/°	104.419(7)
γ/°	90
Volume/Å^3	2009.2(2)
Z	8
ρ_{calc}g/cm^3	1.329
μ/mm^{-1}	0.089
F(000)	847.0
Crystal size/mm^3	0.575 × 0.198 × 0.108
Radiation	MoKα (λ = 0.71073)
2Θ range for data collection/°	4.506 to 66.716
Index ranges	-14 ≤ h ≤ 14, -27 ≤ k ≤ 28, -14 ≤ l ≤ 15
Reflections collected	32477
Independent reflections	6618 [R_{int} = 0.0595, R_{sigma} = 0.0531]
Data/restraints/parameters	6618/0/281
Goodness-of-fit on F^2	1.050
Final R indexes [I>=2σ (I)]	R_1 = 0.0519, wR_2 = 0.1173
Final R indexes [all data]	R_1 = 0.0854, wR_2 = 0.1292
Largest diff. peak/hole / e Å^{-3}	0.31/-0.28

Table S54. Fractional Atomic Coordinates (×10^4) and Equivalent Isotropic Displacement Parameters (Å^2×10^3) for **2b (**Sjkc23fw20). Ueq is defined as 1/3 of the trace of the orthogonalised UIJ tensor.

**Atom**	***x***	***Y***	***z***	**U(eq)**
O3	7299.3(10)	5715.8(5)	7823.1(9)	30.9(2)
O2	7989.7(10)	4362.0(5)	3512.3(9)	33.8(2)
O4	6732.9(11)	5585.8(5)	9772.3(10)	34.4(2)
O1	8525.3(10)	4730.7(5)	5615.0(9)	31.9(2)
N2	8875.1(11)	4503.4(5)	8386.7(11)	23.6(2)
N1	6169.9(11)	5613.7(5)	4878.6(11)	23.0(2)
C7	4390.2(12)	6514.1(6)	4741.8(13)	23.7(2)
C6	5073.8(12)	5985.7(6)	4174.1(12)	22.7(2)
C18	9558.7(12)	3950.6(6)	8984.3(12)	22.8(2)
C1	6596.8(13)	5180.7(6)	4032.6(12)	24.0(2)
C13	8197.1(13)	4805.7(6)	9226.5(12)	24.4(2)
C19	10384.9(12)	3515.1(6)	8349.2(12)	23.0(2)
C2	7784.4(14)	4745.7(6)	4497.1(13)	25.5(3)
C14	7387.8(13)	5408.6(6)	8845.2(12)	25.8(3)
C17	9322.6(13)	3906.8(6)	10236.2(13)	26.9(3)
C4	5774.4(13)	5285.4(6)	2778.4(13)	27.8(3)
C5	4820.8(13)	5788.6(6)	2860.4(13)	26.7(3)
C20	10574.3(13)	2846.8(6)	8728.5(14)	28.7(3)
C16	8464.6(14)	4443.3(6)	10385.8(13)	27.6(3)
C24	11011.8(13)	3748.4(7)	7379.0(14)	28.2(3)
C8	3123.3(13)	6774.0(7)	4001.8(14)	29.9(3)
C22	11955.0(14)	2663.3(7)	7171.4(15)	33.9(3)
C23	11787.6(14)	3323.7(7)	6794.8(14)	32.2(3)
C21	11354.8(14)	2425.1(7)	8144.5(15)	33.3(3)
C9	2497.0(15)	7305.4(7)	4470.9(15)	35.8(3)
C12	4981.5(15)	6789.9(7)	5971.8(15)	37.1(3)
C10	3101.9(15)	7580.1(8)	5685.6(16)	39.8(4)
C3	9173.0(16)	3916.8(7)	3837.2(15)	38.4(3)
C15	5929.0(18)	6192.1(8)	9542.4(17)	42.9(4)
C11	4335.3(16)	7317.3(8)	6442.8(17)	47.6(4)

Table S55. Anisotropic Displacement Parameters ($Å^2 \times 10^3$) for **2b** (Sjkc23fw20). The Anisotropic displacement factor exponent takes the form: $-2\pi^2[h^2a^{*2}U_{11}+2hka^*b^*U_{12}+…]$.

**Atom**	**U_{11}**	**U_{22}**	**U_{33}**	**U_{23}**	**U_{13}**	**U_{12}**
O3	37.1(5)	33.3(5)	22.9(5)	1.4(4)	8.3(4)	4.7(4)
O2	46.3(6)	34.2(5)	22.4(5)	-0.7(4)	11.6(4)	14.6(4)
O4	45.1(6)	34.2(5)	28.0(5)	-1.4(4)	16.7(4)	5.3(4)
O1	41.1(5)	36.4(5)	19.0(5)	2.3(4)	9.3(4)	9.3(4)
N2	27.6(5)	26.3(5)	17.2(5)	-0.2(4)	6.2(4)	-2.2(4)
N1	27.3(5)	23.9(5)	19.0(5)	-0.3(4)	7.8(4)	0.6(4)
C7	24.2(6)	23.4(6)	24.5(6)	0.0(4)	7.9(5)	-0.8(4)
C6	22.5(5)	23.7(6)	22.2(6)	0.4(4)	6.3(4)	-1.9(4)
C18	24.4(6)	23.2(6)	20.0(6)	-0.1(4)	3.8(4)	-4.6(4)
C1	30.8(6)	23.0(6)	20.7(6)	-1.4(4)	11.1(5)	0.1(4)
C13	27.2(6)	27.3(6)	18.7(6)	-3.6(5)	5.6(5)	-3.2(4)
C19	22.4(5)	25.3(6)	20.3(6)	-0.8(4)	3.3(4)	-3.8(4)
C2	35.4(7)	23.4(6)	21.5(6)	1.5(4)	14.0(5)	1.1(5)
C14	28.6(6)	29.2(6)	19.8(6)	-5.3(5)	6.1(5)	-3.9(5)
C17	33.1(6)	27.1(6)	20.0(6)	1.3(5)	5.9(5)	-4.6(5)
C4	33.4(7)	30.0(6)	20.6(6)	-3.9(5)	7.9(5)	-0.7(5)
C5	27.2(6)	31.4(6)	20.3(6)	-1.3(5)	3.3(5)	0.3(5)
C20	29.6(6)	28.3(6)	28.3(7)	3.5(5)	7.6(5)	-1.9(5)
C16	33.4(7)	31.3(6)	19.4(6)	-2.4(5)	9.0(5)	-4.1(5)
C24	29.3(6)	29.3(6)	27.5(7)	4.9(5)	9.8(5)	1.2(5)
C8	29.2(6)	36.0(7)	22.8(7)	-1.2(5)	3.2(5)	3.7(5)
C22	30.7(7)	37.0(7)	34.9(8)	-3.5(6)	10.1(6)	5.1(5)
C23	30.8(6)	39.6(7)	28.8(7)	3.6(6)	12.1(5)	2.0(5)
C21	33.0(7)	27.9(6)	38.4(8)	3.4(6)	7.9(6)	2.0(5)
C9	31.1(7)	41.1(8)	33.0(8)	-0.9(6)	3.6(6)	11.4(6)
C12	30.5(7)	40.5(8)	34.1(8)	-12.4(6)	-3.5(6)	11.0(6)
C10	36.0(7)	39.3(8)	41.1(9)	-12.1(7)	3.9(6)	11.2(6)
C3	52.8(9)	34.9(7)	31.0(8)	3.0(6)	17.0(7)	18.0(6)
C15	55.5(9)	36.6(8)	43.4(9)	1.1(7)	25.1(8)	13.2(7)
C11	39.0(8)	52.8(9)	42.0(9)	-23.6(7)	-6.7(7)	16.1(7)

Table S56. Bond Lengths for **2b** (Sjkc23fw20).

Atom	Atom	Length/Å	Atom	Atom	Length/Å
O3	C14	1.2177(16)	C1	C2	1.4467(18)
O2	C2	1.3409(15)	C1	C4	1.3754(18)
O2	C3	1.4440(16)	C13	C14	1.4514(18)
O4	C14	1.3402(15)	C13	C16	1.3813(18)
O4	C15	1.4413(17)	C19	C20	1.3992(17)
O1	C2	1.2148(16)	C19	C24	1.3943(17)
N2	C18	1.3673(16)	C17	C16	1.4038(18)
N2	C13	1.3713(16)	C4	C5	1.3991(18)
N1	C6	1.3699(16)	C20	C21	1.3854(19)
N1	C1	1.3791(15)	C24	C23	1.3862(19)
C7	C6	1.4610(17)	C8	C9	1.3835(19)
C7	C8	1.3986(17)	C22	C23	1.382(2)
C7	C12	1.3879(18)	C22	C21	1.384(2)
C6	C5	1.3902(17)	C9	C10	1.377(2)
C18	C19	1.4636(17)	C12	C11	1.3889(19)
C18	C17	1.3877(17)	C10	C11	1.382(2)

Table S57.Bond Angles for **2b** (Sjkc23fw20).

Atom	Atom	Atom	Angle/˚	Atom	Atom	Atom	Angle/˚
C2	O2	C3	116.36(11)	C24	C19	C20	118.43(12)
C14	O4	C15	116.70(11)	O2	C2	C1	110.79(11)
C18	N2	C13	109.40(11)	O1	C2	O2	123.61(12)
C6	N1	C1	109.08(11)	O1	C2	C1	125.59(11)
C8	C7	C6	119.18(11)	O3	C14	O4	123.86(12)
C12	C7	C6	122.37(11)	O3	C14	C13	125.31(12)
C12	C7	C8	118.39(12)	O4	C14	C13	110.83(11)
N1	C6	C7	124.18(11)	C18	C17	C16	107.72(11)
N1	C6	C5	107.40(11)	C1	C4	C5	107.42(11)
C5	C6	C7	128.33(11)	C6	C5	C4	107.93(11)
N2	C18	C19	123.12(11)	C21	C20	C19	120.79(12)
N2	C18	C17	107.58(11)	C13	C16	C17	107.22(11)
C17	C18	C19	129.30(11)	C23	C24	C19	120.53(12)
N1	C1	C2	121.20(11)	C9	C8	C7	120.67(13)
C4	C1	N1	108.16(11)	C23	C22	C21	119.89(13)
C4	C1	C2	130.51(11)	C22	C23	C24	120.37(13)
N2	C13	C14	121.17(11)	C22	C21	C20	119.99(13)
N2	C13	C16	108.08(11)	C10	C9	C8	120.40(13)
C16	C13	C14	130.74(12)	C7	C12	C11	120.53(13)
C20	C19	C18	119.70(11)	C9	C10	C11	119.56(13)
C24	C19	C18	121.86(11)	C10	C11	C12	120.42(14)

Table S58. Torsion Angles for **2b** (Sjkc23fw20).

A	B	C	D	Angle/˚	A	B	C	D	Angle/˚
N2	C18	C19	C20	-154.03(12)	C19	C18	C17	C16	179.80(12)
N2	C18	C19	C24	26.80(18)	C19	C20	C21	C22	-0.2(2)
N2	C18	C17	C16	0.60(13)	C19	C24	C23	C22	-0.3(2)
N2	C13	C14	O3	-4.5(2)	C2	C1	C4	C5	-176.34(12)
N2	C13	C14	O4	175.20(11)	C14	C13	C16	C17	-179.28(12)
N2	C13	C16	C17	-0.23(14)	C17	C18	C19	C20	26.88(19)
N1	C6	C5	C4	0.52(14)	C17	C18	C19	C24	-152.29(13)
N1	C1	C2	O2	178.86(11)	C4	C1	C2	O2	-5.66(19)
N1	C1	C2	O1	-2.48(19)	C4	C1	C2	O1	172.99(13)
N1	C1	C4	C5	-0.41(14)	C20	C19	C24	C23	0.86(19)
C7	C6	C5	C4	177.11(11)	C16	C13	C14	O3	174.48(13)
C7	C8	C9	C10	-1.0(2)	C16	C13	C14	O4	-5.85(19)
C7	C12	C11	C10	-0.8(3)	C24	C19	C20	C21	-0.64(19)
C6	N1	C1	C2	177.13(11)	C8	C7	C6	N1	-167.85(12)
C6	N1	C1	C4	0.75(14)	C8	C7	C6	C5	16.08(19)
C6	C7	C8	C9	-175.50(12)	C8	C7	C12	C11	-0.9(2)
C6	C7	C12	C11	176.30(14)	C8	C9	C10	C11	-0.6(3)
C18	N2	C13	C14	179.78(11)	C23	C22	C21	C20	0.7(2)
C18	N2	C13	C16	0.62(14)	C21	C22	C23	C24	-0.5(2)
C18	C19	C20	C21	-179.84(12)	C9	C10	C11	C12	1.5(3)
C18	C19	C24	C23	-179.96(12)	C12	C7	C6	N1	14.98(19)
C18	C17	C16	C13	-0.23(14)	C12	C7	C6	C5	-161.09(14)
C1	N1	C6	C7	-177.55(11)	C12	C7	C8	C9	1.8(2)
C1	N1	C6	C5	-0.78(13)	C3	O2	C2	O1	-0.43(19)
C1	C4	C5	C6	-0.07(14)	C3	O2	C2	C1	178.26(11)
C13	N2	C18	C19	179.99(10)	C15	O4	C14	O3	-2.54(19)
C13	N2	C18	C17	-0.76(13)	C15	O4	C14	C13	177.79(12)

Table S59. Hydrogen Atom Coordinates (Å×10^4) and Isotropic Displacement Parameters (Å^2×10^3) for **2b** **(**Sjkc23fw20).

**Atom**	***X***	***y***	***z***	**U(eq)**
H17	9677.76	3573.69	10877.11	32
H4	5841.62	5057.92	1999.61	33
H5	4123.37	5964.5	2144.7	32
H20	10162.87	2680.16	9394.41	34
H16	8130.26	4539.15	11142.98	33
H24	10906.5	4202.07	7116.29	34
H8	2688.46	6583.78	3168.53	36
H22	12481.22	2373.29	6762.99	41
H23	12206.5	3487.52	6131.82	39
H21	11477.96	1972.56	8412	40
H9	1644.28	7481.79	3951.54	43
H12	5834.89	6616.48	6495.81	44
H10	2674.37	7947.82	6001.27	48
H3A	9095.29	3588.03	3129.67	58
H3B	9189.65	3687.57	4668.95	58
H3C	10036.97	4172.51	3933.63	58
H15A	5216.7	6158	8704.14	64
H15B	6549.42	6567.89	9502.07	64
H15C	5474.39	6262.72	10264.6	64
H11	4743.74	7498.54	7290.71	57
H1	6501(16)	5627(8)	5752(18)	37(4)
H2	8786(17)	4632(8)	7538(19)	40(4)

10. Calculations

10.1. Calculations for 1b

10.1.1. Optimization of Azide 1b-A

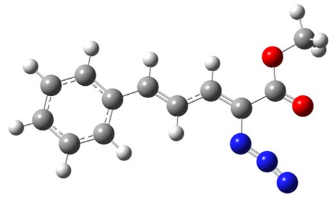

Energy (B3LYP-D/6-31+G(d,p)): = -778.592489 Hartrees
Standard orientation:

Center Number	Atomic Number	Atomic Type	Coordinates (Angstroms) X	Y	Z
1	6	0	5.390266	-1.009838	-0.000250
2	6	0	4.041546	-1.365069	-0.000191
3	6	0	3.027494	-0.385383	-0.000114
4	6	0	3.418437	0.971098	-0.000099
5	6	0	4.764584	1.325100	-0.000158
6	6	0	5.758570	0.337997	-0.000234
7	1	0	6.152183	-1.783851	-0.000309
8	1	0	3.762707	-2.415887	-0.000204
9	1	0	2.666100	1.753143	-0.000041
10	1	0	5.043001	2.374992	-0.000145
11	1	0	6.807425	0.619242	-0.000280
12	6	0	1.632182	-0.817318	-0.000054
13	6	0	0.525228	-0.030220	0.000022
14	6	0	-0.803368	-0.573511	0.000069
15	6	0	-1.953796	0.161513	0.000150
16	6	0	-3.299077	-0.470880	0.000216
17	6	0	-4.534362	-2.482487	-0.000145
18	8	0	-4.340348	0.163661	0.000167
19	8	0	-3.253192	-1.820284	-0.000018
20	1	0	-4.306489	-3.547916	-0.000360
21	1	0	-5.104653	-2.207115	0.890584
22	1	0	-5.104665	-2.206758	-0.890755
23	7	0	-1.855416	1.562284	0.000166
24	7	0	-2.849798	2.306070	0.000234
25	7	0	-3.603016	3.160060	0.000320
26	1	0	1.483138	-1.897513	-0.000076
27	1	0	0.615211	1.052212	0.000050
28	1	0	-0.914250	-1.652483	0.000044

10.1.2. Optimization of Azide 1b-B

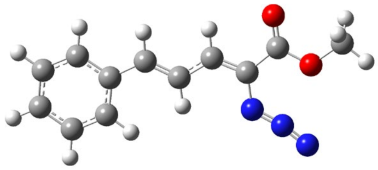

Energy (B3LYP-D/6-31+G(d,p)): -778.591587 Hartrees
Standard orientation:

Center Number	Atomic Number	Atomic Type	Coordinates (Angstroms) X	Y	Z
1	6	0	5.524495	-0.868268	-0.035028
2	6	0	4.196591	-1.294404	-0.053177
3	6	0	3.132125	-0.371094	-0.005364
4	6	0	3.450145	1.002621	0.062188
5	6	0	4.775500	1.427435	0.080244
6	6	0	5.820434	0.495644	0.031654
7	1	0	6.326515	-1.599637	-0.072909
8	1	0	3.973832	-2.357203	-0.105208
9	1	0	2.657120	1.742344	0.101600
10	1	0	4.997886	2.489316	0.132692
11	1	0	6.852857	0.831978	0.046066
12	6	0	1.761940	-0.876579	-0.027139
13	6	0	0.614334	-0.150608	0.003704
14	6	0	-0.680899	-0.766775	-0.026370
15	6	0	-1.874060	-0.107601	0.005560
16	6	0	-3.125933	-0.917936	-0.015593
17	6	0	-5.488450	-0.856416	0.149812
18	8	0	-3.161848	-2.125165	-0.169855
19	8	0	-4.226991	-0.157559	0.163464
20	1	0	-6.245385	-0.085286	0.287822
21	1	0	-5.623905	-1.371340	-0.804382
22	1	0	-5.522108	-1.587143	0.961591
23	7	0	-1.860659	1.298092	0.126391
24	7	0	-2.851069	2.006094	-0.088727
25	7	0	-3.648013	2.807297	-0.240204
26	1	0	1.670209	-1.961925	-0.074875
27	1	0	0.645157	0.934205	0.050818
28	1	0	-0.740612	-1.850019	-0.077258

10.1.3. Optimization of Azide 1b-C

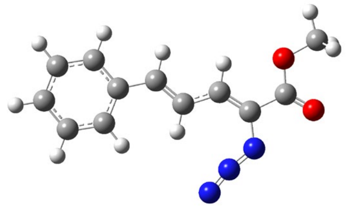

Energy (B3LYP-D/6-31+G(d,p)): = -778.584834 Hartrees
Standard orientation:

Center Number	Atomic Number	Atomic Type	Coordinates (Angstroms) X	Y	Z
1	6	0	-5.024934	-1.429407	0.035415
2	6	0	-3.649668	-1.650138	0.104826
3	6	0	-2.734383	-0.579729	0.039585
4	6	0	-3.251123	0.727951	-0.086901
5	6	0	-4.623898	0.947339	-0.155967
6	6	0	-5.518518	-0.128907	-0.096972
7	1	0	-5.709847	-2.270754	0.085366
8	1	0	-3.272445	-2.664600	0.206916
9	1	0	-2.578704	1.579265	-0.121416
10	1	0	-5.000621	1.961522	-0.251182
11	1	0	-6.588668	0.047664	-0.149355
12	6	0	-1.305252	-0.872277	0.108995
13	6	0	-0.279295	0.005139	-0.042744
14	6	0	1.095107	-0.408867	0.040267
15	6	0	2.216741	0.359812	-0.071132
16	6	0	3.586796	-0.235793	-0.068709
17	6	0	4.853123	-2.225786	0.046670
18	8	0	4.614286	0.407044	-0.145281
19	8	0	3.561785	-1.587579	0.026302
20	1	0	4.646318	-3.292392	0.131375
21	1	0	5.399445	-2.008361	-0.874681
22	1	0	5.438708	-1.874693	0.900296
23	7	0	2.295621	1.756849	-0.251863
24	7	0	1.386600	2.509087	0.106541
25	7	0	0.653796	3.341402	0.383715
26	1	0	-1.053298	-1.915957	0.298204
27	1	0	-0.505027	1.043748	-0.255194
28	1	0	1.275274	-1.468553	0.192762

10.1.4. Optimization of Azide 1b-D

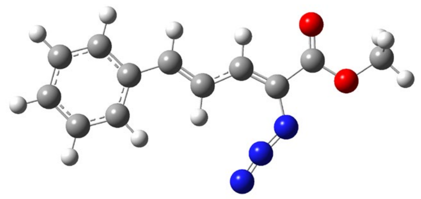

Energy (B3LYP-D/6-31+G(d,p)): = -778.586368 Hartrees
Standard orientation:

Center Number	Atomic Number	Atomic Type	Coordinates (Angstroms) X	Y	Z
1	6	0	-5.206929	-1.230963	-0.023292
2	6	0	-3.850272	-1.545967	-0.094618
3	6	0	-2.863345	-0.540849	-0.034126
4	6	0	-3.288784	0.799505	0.091295
5	6	0	-4.643055	1.113033	0.161957
6	6	0	-5.609682	0.100705	0.106732
7	1	0	-5.948136	-2.023364	-0.069859
8	1	0	-3.543797	-2.584094	-0.195155
9	1	0	-2.559288	1.602518	0.124510
10	1	0	-4.949016	2.150863	0.256260
11	1	0	-6.665064	0.350568	0.160683
12	6	0	-1.458354	-0.931772	-0.107314
13	6	0	-0.372984	-0.124958	0.025146
14	6	0	0.966379	-0.636124	-0.062340
15	6	0	2.145221	0.041772	0.037278
16	6	0	3.410940	-0.751547	0.008108
17	6	0	5.764578	-0.691207	0.095560
18	8	0	3.447418	-1.966083	-0.095988
19	8	0	4.510359	0.017726	0.108029
20	1	0	6.530096	0.078179	0.191371
21	1	0	5.812639	-1.394399	0.931094
22	1	0	5.880831	-1.241500	-0.841757
23	7	0	2.346848	1.427578	0.213238
24	7	0	1.487281	2.255052	-0.100747
25	7	0	0.814401	3.147076	-0.342562
26	1	0	-1.279153	-1.992455	-0.283370
27	1	0	-0.524583	0.930165	0.222157
28	1	0	1.081260	-1.707348	-0.208081

10.1.5. Optimization of T of 1b-A

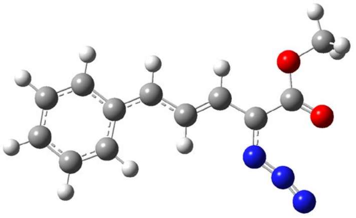

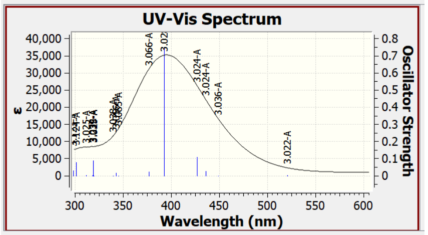

Energy (B3LYP-D/6-31+G(d,p)) = -778.536441 Hartree
Standard orientation:

Center Number	Atomic Number	Atomic Type	Coordinates (Angstroms) X	Y	Z
1	6	0	5.424590	-0.959728	0.000262
2	6	0	4.102107	-1.374691	0.000574
3	6	0	3.025547	-0.434853	0.000170
4	6	0	3.373577	0.951284	-0.000597
5	6	0	4.701377	1.354769	-0.000900
6	6	0	5.739281	0.409568	-0.000472
7	1	0	6.220914	-1.698580	0.000584
8	1	0	3.867889	-2.436151	0.001145
9	1	0	2.593283	1.704553	-0.000968
10	1	0	4.937632	2.415156	-0.001482
11	1	0	6.775264	0.733934	-0.000708
12	6	0	1.688774	-0.908486	0.000535
13	6	0	0.499769	-0.101729	0.000298
14	6	0	-0.758930	-0.626242	0.000510
15	6	0	-1.986639	0.154012	0.000341
16	6	0	-3.317488	-0.444431	-0.000124
17	6	0	-4.572439	-2.446860	-0.000686
18	8	0	-4.358273	0.207876	-0.000173
19	8	0	-3.285065	-1.801321	-0.000463
20	1	0	-4.359259	-3.515542	-0.000906
21	1	0	-5.140782	-2.164823	0.889617
22	1	0	-5.140650	-2.164496	-0.890969
23	7	0	-1.848524	1.518222	0.000713
24	7	0	-2.820754	2.304245	0.000314
25	7	0	-3.527687	3.198706	0.000046
26	1	0	1.547752	-1.988049	0.001052
27	1	0	0.602998	0.979886	-0.000044
28	1	0	-0.896731	-1.702222	0.000789

10.1.6. Optimization of T of 1b-B

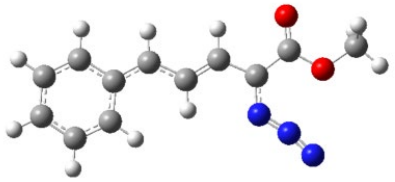

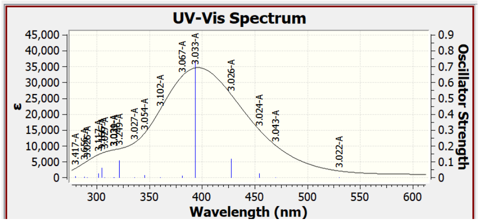

Energy (B3LYP-D/6-31+G(d,p)): = -778.535001 Hartree
Standard orientation:

Center Number	Atomic Number	Atomic Type	Coordinates (Angstroms) X	Y	Z
1	6	0	5.556990	-0.807555	-0.000333
2	6	0	4.260053	-1.296294	-0.000404
3	6	0	3.132070	-0.418498	-0.000174
4	6	0	3.401893	0.985159	0.000140
5	6	0	4.704845	1.462685	0.000209
6	6	0	5.794283	0.577317	-0.000027
7	1	0	6.393587	-1.500467	-0.000515
8	1	0	4.085773	-2.369169	-0.000642
9	1	0	2.580514	1.693412	0.000338
10	1	0	4.881179	2.534687	0.000452
11	1	0	6.810398	0.959406	0.000028
12	6	0	1.824569	-0.966943	-0.000262
13	6	0	0.591484	-0.228112	-0.000099
14	6	0	-0.634716	-0.823136	-0.000074
15	6	0	-1.906429	-0.118460	0.000070
16	6	0	-3.142735	-0.900143	0.000032
17	6	0	-5.513241	-0.827114	0.000001
18	8	0	-3.174378	-2.124900	-0.000184
19	8	0	-4.257328	-0.122273	0.000087
20	1	0	-6.279135	-0.052047	0.000386
21	1	0	-5.595321	-1.455050	-0.890891
22	1	0	-5.595079	-1.455721	0.890438
23	7	0	-1.845449	1.254521	0.000233
24	7	0	-2.831977	2.011059	0.000343
25	7	0	-3.593876	2.861379	0.000476
26	1	0	1.743665	-2.052483	-0.000467
27	1	0	0.634523	0.857770	0.000001
28	1	0	-0.721749	-1.905100	-0.000175

10.1.7. Optimization of T of 1b-C

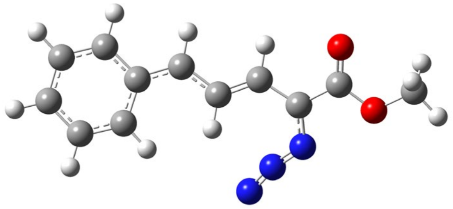

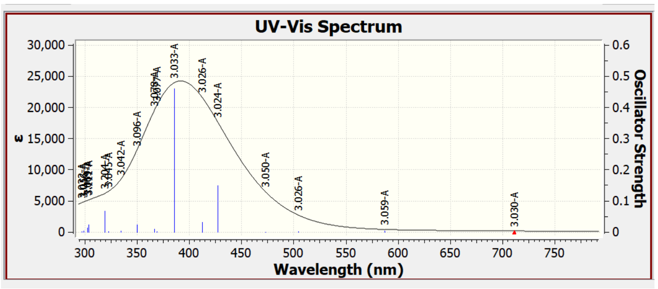

Energy (B3LYP-D/6-31+G(d,p)): = -778.530930 Hartree
Standard orientation:

Center Number	Atomic Number	Atomic Type	Coordinates (Angstroms) X	Y	Z
1	6	0	5.227655	-1.122522	-0.160978
2	6	0	3.902021	-1.476159	-0.364865
3	6	0	2.837778	-0.572013	-0.072124
4	6	0	3.195653	0.712884	0.435220
5	6	0	4.525702	1.057505	0.633466
6	6	0	5.553201	0.147686	0.340474
7	1	0	6.016263	-1.832984	-0.391585
8	1	0	3.658519	-2.461414	-0.753899
9	1	0	2.424424	1.439692	0.667118
10	1	0	4.770718	2.043093	1.018901
11	1	0	6.590785	0.424503	0.499604
12	6	0	1.495497	-0.984056	-0.297652
13	6	0	0.317701	-0.209340	-0.048016
14	6	0	-0.942588	-0.657675	-0.320915
15	6	0	-2.187932	0.072196	-0.105437
16	6	0	-3.421688	-0.700252	0.075089
17	6	0	-5.753773	-0.665347	0.418173
18	8	0	-3.440813	-1.926398	0.064362
19	8	0	-4.524694	0.062850	0.243095
20	1	0	-6.523616	0.095779	0.543429
21	1	0	-5.960347	-1.284333	-0.459206
22	1	0	-5.696126	-1.307427	1.301324
23	7	0	-2.327531	1.432066	-0.087474
24	7	0	-1.397175	2.211031	-0.368360
25	7	0	-0.670502	3.078896	-0.555646
26	1	0	1.346755	-1.981280	-0.707658
27	1	0	0.450890	0.772548	0.395132
28	1	0	-1.083111	-1.671176	-0.687076

10.1.8. Optimization of T of 1b-D

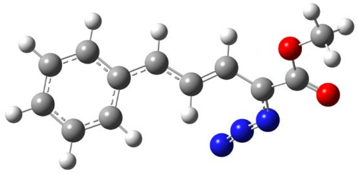

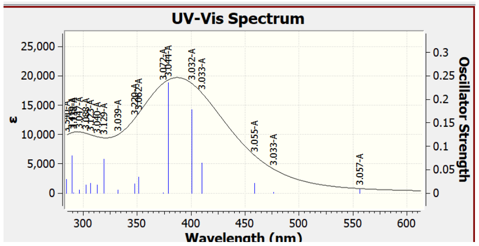

Energy (B3LYP-D/6-31+G(d,p)): = -778.529612 Hartree
Standard orientation:

Center Number	Atomic Number	Atomic Type	Coordinates (Angstroms) X	Y	Z
1	6	0	-5.044478	-1.217139	-0.328397
2	6	0	-3.708005	-1.429127	-0.638125
3	6	0	-2.687720	-0.560332	-0.155396
4	6	0	-3.095595	0.538290	0.654564
5	6	0	-4.435305	0.743818	0.957602
6	6	0	-5.420818	-0.128776	0.472668
7	1	0	-5.800430	-1.897538	-0.709680
8	1	0	-3.424923	-2.274302	-1.260267
9	1	0	-2.356968	1.232739	1.040495
10	1	0	-4.719918	1.590534	1.575620
11	6	0	-1.328970	-0.824479	-0.501807
12	6	0	-0.190404	-0.070165	-0.098852
13	6	0	1.086036	-0.353516	-0.499728
14	6	0	2.274289	0.417954	-0.121417
15	6	0	3.564074	-0.213702	0.177122
16	6	0	4.683462	-2.287483	0.376827
17	8	0	4.600153	0.372021	0.447048
18	8	0	3.465609	-1.570672	0.105552
19	1	0	4.426380	-3.342312	0.281130
20	1	0	5.459069	-2.010506	-0.342437
21	1	0	5.040124	-2.065699	1.386262
22	7	0	2.315139	1.783997	-0.044949
23	7	0	1.341442	2.483883	-0.381507
24	7	0	0.537631	3.270840	-0.600885
25	1	0	-1.145093	-1.681284	-1.147824
26	1	0	-0.350209	0.771099	0.571046
27	1	0	1.272979	-1.234535	-1.108690
28	1	0	-6.465983	0.037911	0.714571

10.1.9. Optimization of ^{3}Br1b-A

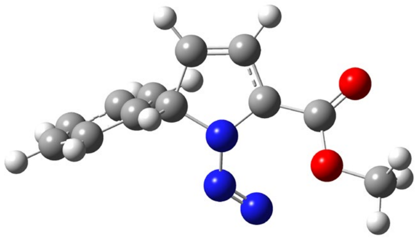

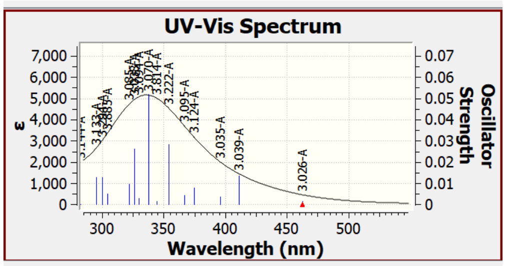

Energy (B3LYP-D/6-31+G(d,p)): = -778.542621 Hartree
Standard orientation:

Center Number	Atomic Number	Atomic Type	Coordinates (Angstroms) X	Y	Z
1	6	0	-1.437511	0.730739	-0.007374
2	6	0	-0.942592	2.063122	0.070012
3	6	0	0.284951	2.139513	-0.515781
4	6	0	0.720357	0.779734	-1.002466
5	7	0	-0.476169	-0.041911	-0.663366
6	1	0	-1.520268	2.871148	0.499235
7	6	0	1.999803	0.271807	-0.353968
8	6	0	2.088738	0.168947	1.041197
9	6	0	3.261545	-0.290762	1.638429
10	6	0	4.361413	-0.642522	0.847093
11	6	0	4.278786	-0.537150	-0.542607
12	6	0	3.098237	-0.084803	-1.140688
13	1	0	1.235553	0.444891	1.654800
14	1	0	3.319575	-0.373990	2.719664
15	1	0	5.275205	-0.999054	1.313204
16	1	0	5.126381	-0.813684	-1.162737
17	1	0	3.030387	-0.018226	-2.223601
18	6	0	-2.807855	0.351960	0.320021
19	6	0	-4.548858	-1.213206	-0.007976
20	8	0	-3.504183	0.998277	1.092548
21	8	0	-3.231606	-0.741078	-0.348120
22	1	0	-4.599201	-1.458323	1.056131
23	1	0	-5.298244	-0.451416	-0.238194
24	1	0	-4.700119	-2.104256	-0.616204
25	7	0	-0.265973	-1.414611	-0.567672
26	7	0	-0.954597	-2.167987	0.045340
27	1	0	0.902500	3.018883	-0.638634
28	1	0	0.849634	0.773714	-2.094564

10.1.10. Optimization of ^{3}Br1b-B

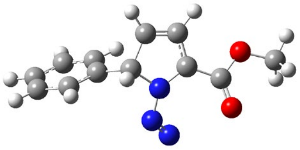

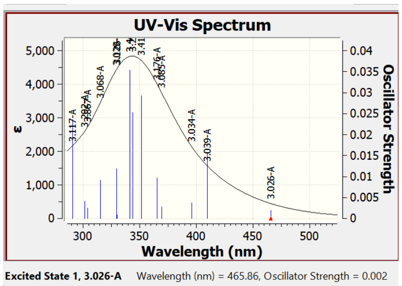

Energy (B3LYP-D/6-31+G(d,p)): = - 778.541696 Hartree
Standard orientation:

Center Number	Atomic Number	Atomic Type	Coordinates (Angstroms) X	Y	Z
1	6	0	1.451770	-0.396267	-0.224784
2	6	0	1.111897	-1.781442	-0.194383
3	6	0	-0.138320	-1.965223	-0.702507
4	6	0	-0.748955	-0.639512	-1.083454
5	7	0	0.370586	0.287660	-0.774806
6	1	0	1.796860	-2.544161	0.150454
7	6	0	-2.027460	-0.309422	-0.325942
8	6	0	-2.019168	-0.244363	1.074118
9	6	0	-3.190931	0.047038	1.770912
10	6	0	-4.385566	0.266871	1.075057
11	6	0	-4.399047	0.199177	-0.319333
12	6	0	-3.220614	-0.084231	-1.017308
13	1	0	-1.091671	-0.416510	1.613295
14	1	0	-3.174120	0.103033	2.855342
15	1	0	-5.298094	0.493238	1.618584
16	1	0	-5.320842	0.375365	-0.865859
17	1	0	-3.229660	-0.120321	-2.103742
18	6	0	2.774089	0.186260	-0.032481
19	6	0	4.912296	-0.192968	0.902552
20	8	0	3.161389	1.246273	-0.496236
21	8	0	3.557760	-0.642804	0.711674
22	1	0	5.421730	-0.092750	-0.059842
23	1	0	4.924750	0.773145	1.413846
24	1	0	5.388369	-0.959735	1.513475
25	7	0	0.039626	1.636066	-0.648513
26	7	0	0.694261	2.439156	-0.062480
27	1	0	-0.666615	-2.900304	-0.830229
28	1	0	-0.955158	-0.594437	-2.162921

10.1.11. Optimization of ^{3}Br2b-B

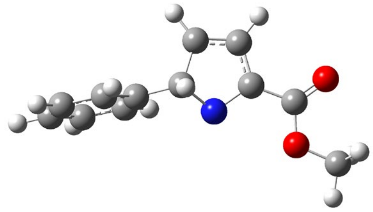

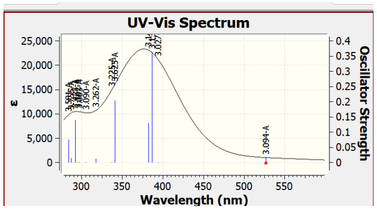

Energy (B3LYP-D/6-31+G(d,p)): = -669.047231 Hartrees
Standard orientation:

Center Number	Atomic Number	Atomic Type	Coordinates (Angstroms) X	Y	Z
1	6	0	1.659451	0.551645	-0.101493
2	6	0	1.205374	1.794849	-0.526726
3	6	0	-0.164000	1.870848	-0.238862
4	6	0	-0.548064	0.637782	0.573532
5	7	0	0.544366	-0.273604	0.194733
6	1	0	1.816195	2.546296	-1.012330
7	6	0	-1.929729	0.099629	0.271826
8	6	0	-2.986140	0.330180	1.160858
9	6	0	-4.275783	-0.119179	0.863446
10	6	0	-4.517434	-0.809709	-0.327151
11	6	0	-3.464728	-1.047668	-1.217331
12	6	0	-2.179130	-0.591823	-0.921791
13	1	0	-2.799804	0.859613	2.092467
14	1	0	-5.086637	0.063791	1.562609
15	1	0	-5.517763	-1.164106	-0.558388
16	1	0	-3.645561	-1.589883	-2.141019
17	1	0	-1.356905	-0.784633	-1.604227
18	6	0	3.047637	0.102707	-0.099216
19	6	0	4.524822	-1.675409	0.378814
20	8	0	3.989567	0.803579	-0.448121
21	8	0	3.178114	-1.169772	0.341010
22	1	0	4.963524	-1.669472	-0.622788
23	1	0	5.144840	-1.065647	1.041869
24	1	0	4.440133	-2.694139	0.756547
25	1	0	-0.871604	2.619792	-0.572340
26	1	0	-0.472077	0.860044	1.655915

10.1.12. Optimization of ^{3}Br2b-B

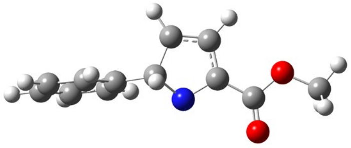

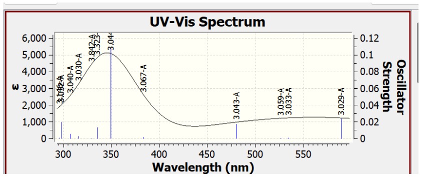

Energy (B3LYP-D/6-31+G(d,p)): = -669.045845 Hartrees
Standard orientation:

Center Number	Atomic Number	Atomic Type	Coordinates (Angstroms) X	Y	Z
1	6	0	1.616268	0.189816	-0.019174
2	6	0	1.303211	1.307522	-0.785562
3	6	0	-0.061865	1.574657	-0.614448
4	6	0	-0.595704	0.644261	0.470429
5	7	0	0.413888	-0.423147	0.418016
6	1	0	1.996989	1.848593	-1.417082
7	6	0	-2.012322	0.165958	0.235804
8	6	0	-3.076307	0.750184	0.933263
9	6	0	-4.392671	0.352457	0.683681
10	6	0	-4.654355	-0.640304	-0.264441
11	6	0	-3.594700	-1.231794	-0.960301
12	6	0	-2.281260	-0.828113	-0.714977
13	1	0	-2.875386	1.516050	1.678961
14	1	0	-5.209429	0.811107	1.233526
15	1	0	-5.676027	-0.955074	-0.456319
16	1	0	-3.791609	-2.009794	-1.692291
17	1	0	-1.454808	-1.294292	-1.242631
18	6	0	2.915126	-0.418184	0.244504
19	6	0	5.243574	-0.236932	-0.136327
20	8	0	3.099928	-1.417886	0.918764
21	8	0	3.920174	0.284388	-0.352530
22	1	0	5.482541	-0.244854	0.930730
23	1	0	5.322667	-1.255998	-0.524863
24	1	0	5.912055	0.434117	-0.675780
25	1	0	-0.680084	2.258999	-1.182345
26	1	0	-0.538918	1.143971	1.457409

10.1.13. Optimization of 2b-A

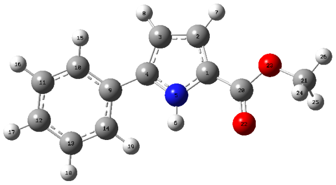

Energy (B3LYP-D/6-31+G(d,p)): = -669.181765 Hartrees
Standard orientation:

Center Number	Atomic Number	Atomic Type	Coordinates (Angstroms) X	Y	Z
1	6	0	1.649781	0.325623	-0.054141
2	6	0	1.374474	1.672586	-0.268742
3	6	0	-0.028890	1.813303	-0.287555
4	6	0	-0.592755	0.547948	-0.086716
5	7	0	0.445214	-0.334422	0.044989
6	1	0	0.383003	-1.318192	0.266272
7	1	0	2.117068	2.445081	-0.403454
8	1	0	-0.585407	2.723058	-0.461727
9	6	0	-1.996330	0.134361	-0.032448
10	6	0	-2.991238	1.058033	0.339051
11	6	0	-4.331944	0.679474	0.389056
12	6	0	-4.708184	-0.631924	0.080252
13	6	0	-3.729187	-1.559204	-0.287474
14	6	0	-2.387814	-1.180062	-0.348806
15	1	0	-2.705643	2.070497	0.606999
16	1	0	-5.083995	1.406806	0.680917
17	1	0	-5.752238	-0.926873	0.124468
18	1	0	-4.010391	-2.577672	-0.539146
19	1	0	-1.647039	-1.904890	-0.674912
20	6	0	2.881722	-0.435428	0.083701
21	6	0	5.242352	-0.355162	0.092203
22	8	0	2.915546	-1.643138	0.287361
23	8	0	3.985807	0.336168	-0.036080
24	1	0	5.320828	-0.826784	1.075385
25	1	0	5.337238	-1.122940	-0.680291
26	1	0	6.007349	0.411339	-0.029966

10.1.14. Optimization of 2b-B

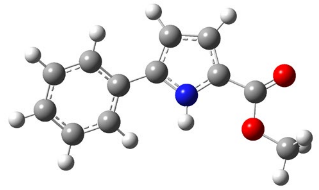

Energy (B3LYP-D/6-31+G(d,p)): = -669.179949 Hartree
Standard orientation:

Center Number	Atomic Number	Atomic Type	Coordinates (Angstroms) X	Y	Z
1	6	0	1.701445	0.686089	-0.076147
2	6	0	1.290888	2.005671	-0.228147
3	6	0	-0.119218	2.009156	-0.229272
4	6	0	-0.554377	0.687987	-0.081201
5	7	0	0.566502	-0.095546	0.002219
6	1	0	0.587896	-1.087437	0.184891
7	1	0	1.957295	2.849116	-0.334115
8	1	0	-0.764047	2.866488	-0.358372
9	6	0	-1.909577	0.135997	-0.032488
10	6	0	-2.983115	0.936451	0.400938
11	6	0	-4.279395	0.425844	0.447010
12	6	0	-4.531605	-0.898037	0.072283
13	6	0	-3.473731	-1.703807	-0.357883
14	6	0	-2.177042	-1.191803	-0.415421
15	1	0	-2.792466	1.956436	0.719835
16	1	0	-5.093424	1.059408	0.787140
17	1	0	-5.541028	-1.295956	0.113321
18	1	0	-3.659204	-2.729892	-0.662044
19	1	0	-1.375348	-1.821090	-0.792320
20	6	0	3.048319	0.140924	0.004837
21	6	0	4.318336	-1.847107	0.262305
22	8	0	4.078084	0.788841	-0.061158
23	8	0	3.027513	-1.215953	0.169494
24	1	0	4.895161	-1.671356	-0.649494
25	1	0	4.874652	-1.453954	1.117246
26	1	0	4.114655	-2.910227	0.390791

10.1.15. Optimization of T_A of 1

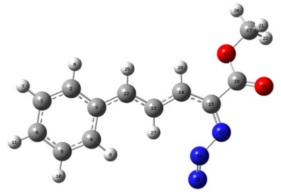

Energy (B3LYP-D/6-31+G(d,p)): -778.538515 Hartrees
Standard orientation:

Center Number	Atomic Number	Atomic Type	Coordinates (Angstroms) X	Y	Z
1	6	0	-5.030413	-1.404601	-0.113075
2	6	0	-3.661507	-1.633941	-0.215211
3	6	0	-2.729362	-0.577217	-0.078710
4	6	0	-3.233289	0.725444	0.160363
5	6	0	-4.601431	0.949793	0.261415
6	6	0	-5.507743	-0.111134	0.126423
7	1	0	-5.726338	-2.231261	-0.220189
8	1	0	-3.295510	-2.640343	-0.401401
9	1	0	-2.551917	1.563380	0.263213
10	1	0	-4.967658	1.955559	0.444322
11	1	0	-6.575196	0.071038	0.205786
12	6	0	-1.319655	-0.873666	-0.189580
13	6	0	-0.270599	0.024952	-0.057060
14	6	0	1.064195	-0.375585	-0.178200
15	6	0	2.243602	0.433929	-0.112654
16	6	0	3.589431	-0.281253	-0.109895
17	6	0	4.707981	-2.292994	0.423661
18	8	0	4.628896	0.201034	-0.495706
19	8	0	3.480035	-1.535146	0.384805
20	1	0	4.437575	-3.259590	0.847330
21	1	0	5.113900	-2.407590	-0.584587
22	1	0	5.444297	-1.784519	1.050760
23	7	0	2.406085	1.736356	-0.058102
24	7	0	1.195663	2.506675	-0.244653
25	7	0	0.976855	3.498218	0.352329
26	1	0	-1.067237	-1.912326	-0.401541
27	1	0	-0.491107	1.063291	0.151737
28	1	0	1.250251	-1.435857	-0.320105

10.1.16. Optimization of Nitrene ^{3}1Nb-A

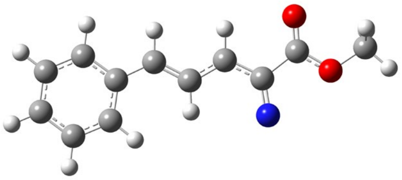

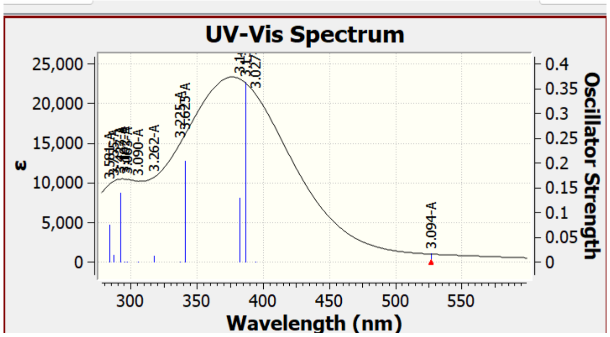

Energy (B3LYP-D/6-31+G(d,p)) = -669.070375 Hartrees
Standard orientation:

Center Number	Atomic Number	Atomic Type	Coordinates (Angstroms) X	Y	Z
1	6	0	-5.057338	-0.979497	0.000069
2	6	0	-3.704086	-1.308094	0.000163
3	6	0	-2.705703	-0.306019	-0.000005
4	6	0	-3.127046	1.045584	-0.000274
5	6	0	-4.479418	1.370259	-0.000367
6	6	0	-5.453048	0.362202	-0.000197
7	1	0	-5.804127	-1.768031	0.000203
8	1	0	-3.403456	-2.352716	0.000370
9	1	0	-2.392421	1.843974	-0.000410
10	1	0	-4.780080	2.413917	-0.000574
11	1	0	-6.507375	0.621844	-0.000271
12	6	0	-1.313786	-0.706768	0.000103
13	6	0	-0.206276	0.121686	-0.000032
14	6	0	1.104384	-0.368706	0.000090
15	6	0	2.273855	0.469466	-0.000045
16	6	0	3.615990	-0.263076	0.000190
17	6	0	5.960372	-0.018652	0.000109
18	8	0	3.718507	-1.473055	0.000345
19	8	0	4.649392	0.586575	0.000010
20	1	0	6.659650	0.816370	-0.000061
21	1	0	6.091529	-0.636842	0.891726
22	1	0	6.091506	-0.637185	-0.891274
23	7	0	2.262356	1.757172	-0.000251
24	1	0	-1.131028	-1.781084	0.000315
25	1	0	-0.338961	1.200759	-0.000244
26	1	0	1.287680	-1.439685	0.000304

10.1.17. Optimization of ^{3}1Nb-B

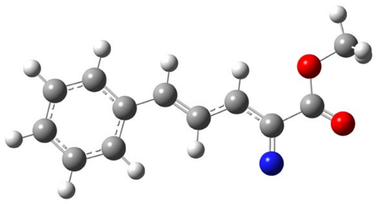

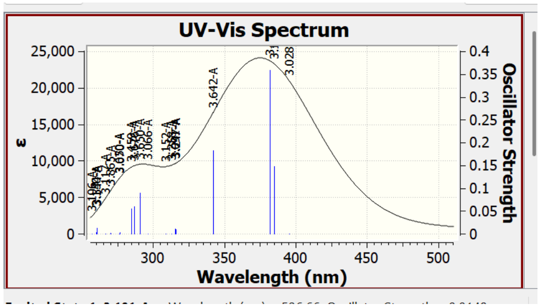

Energy (B3LYP-D/6-31+G(d,p)): -669.065860 Hartrees
Standard orientation:

Center Number	Atomic Number	Atomic Type	Coordinates (Angstroms) X	Y	Z
1	6	0	-4.886882	-1.171979	0.000056
2	6	0	-3.512515	-1.396705	0.000013
3	6	0	-2.593443	-0.321606	0.000000
4	6	0	-3.116044	0.994019	0.000027
5	6	0	-4.489176	1.214939	0.000069
6	6	0	-5.383298	0.135760	0.000084
7	1	0	-5.571633	-2.014934	0.000066
8	1	0	-3.133443	-2.415528	-0.000009
9	1	0	-2.444271	1.845951	0.000012
10	1	0	-4.868241	2.232706	0.000088
11	1	0	-6.454294	0.314511	0.000116
12	6	0	-1.174688	-0.615083	-0.000042
13	6	0	-0.134092	0.294790	-0.000020
14	6	0	1.211989	-0.094296	-0.000059
15	6	0	2.309542	0.838359	-0.000038
16	6	0	3.748149	0.327946	-0.000210
17	6	0	5.127822	-1.589358	0.000111
18	8	0	4.725275	1.041580	0.000197
19	8	0	3.800083	-1.021453	-0.000139
20	1	0	4.979544	-2.668649	0.000324
21	1	0	5.674394	-1.270240	-0.890646
22	1	0	5.674239	-1.269857	0.890824
23	7	0	2.156327	2.120910	-0.000145
24	1	0	-0.911508	-1.672736	-0.000091
25	1	0	-0.349075	1.360394	0.000036
26	1	0	1.462956	-1.149731	-0.000117

10.1.18. Transition state barrier for T of 1b forming ^{3}Br1b

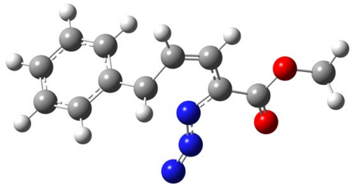

Energy (B3LYP-D/6-31+G(d,p)): -778.510649 Hartrees
Imaginary Frequency -449 cm^{-1} (439)

Center Number	Atomic Number	Atomic Type	Coordinates (Angstroms) X	Y	Z
1	6	0	1.566133	-0.197622	-0.325615
2	6	0	1.202469	-1.492971	-0.797064
3	6	0	-0.069447	-1.640698	-1.265394
4	6	0	-0.959442	-0.459750	-1.284825
5	7	0	0.530760	0.695761	-0.439507
6	1	0	1.921094	-2.301063	-0.756740
7	6	0	-2.153650	-0.374892	-0.465585
8	6	0	-2.330358	-1.199227	0.669460
9	6	0	-3.474187	-1.088213	1.455078
10	6	0	-4.465777	-0.153040	1.132781
11	6	0	-4.303363	0.673403	0.013183
12	6	0	-3.162677	0.565835	-0.775759
13	1	0	-1.558611	-1.918084	0.928366
14	1	0	-3.594544	-1.728579	2.324154
15	1	0	-5.355673	-0.067310	1.749076
16	1	0	-5.067379	1.402575	-0.239868
17	1	0	-3.035715	1.215062	-1.638240
18	6	0	2.866527	0.169925	0.237467
19	6	0	5.038070	-0.605245	0.760035
20	8	0	3.158262	1.276644	0.664183
21	8	0	3.730087	-0.880745	0.224270
22	1	0	5.527590	0.185343	0.185089
23	1	0	4.964786	-0.292526	1.805074
24	1	0	5.588202	-1.542144	0.674625
25	7	0	0.619831	1.942562	-0.131527
26	7	0	0.051131	2.959552	-0.062804
27	1	0	-0.446211	-2.601018	-1.610499
28	1	0	-0.988176	0.110384	-2.214365

10.1.19. Transition state barrier for ^{3}Br1b forming ^{31}Br2b

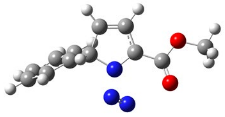

Energy (B3LYP-D/6-311+G(d,p)): -778.537101 Hartrees
Imaginary Frequency: -460 cm^{-1} (46)
Standard Orientation

Center Number	Atomic Number	Atomic Type	Coordinates (Angstroms) X	Y	Z
1	6	0	-1.439602	0.487822	-0.256099
2	6	0	-1.140017	1.834484	0.093946
3	6	0	0.105644	2.158665	-0.364353
4	6	0	0.747387	0.957886	-1.020426
5	7	0	-0.400848	0.011461	-1.052868
6	1	0	-1.840995	2.485237	0.599700
7	6	0	1.972672	0.434171	-0.277477
8	6	0	1.914451	0.179589	1.100019
9	6	0	3.031577	-0.314285	1.772343
10	6	0	4.223681	-0.549080	1.077044
11	6	0	4.289065	-0.291138	-0.293480
12	6	0	3.164829	0.196178	-0.967696
13	1	0	0.990414	0.363766	1.640497
14	1	0	2.974559	-0.514633	2.838263
15	1	0	5.093989	-0.931537	1.602150
16	1	0	5.209396	-0.473788	-0.840468
17	1	0	3.213723	0.383010	-2.037497
18	6	0	-2.710606	-0.207515	-0.109588
19	6	0	-4.810411	-0.156785	0.976942
20	8	0	-3.047327	-1.214764	-0.713096
21	8	0	-3.511846	0.433590	0.788574
22	1	0	-5.370584	-0.159804	0.037761
23	1	0	-4.714448	-1.184990	1.336151
24	1	0	-5.307370	0.466772	1.720141
25	7	0	0.083374	-1.552558	-0.921998
26	7	0	-0.469815	-2.296575	-0.244293
27	1	0	0.602415	3.117914	-0.298181
28	1	0	1.041293	1.181206	-2.055290

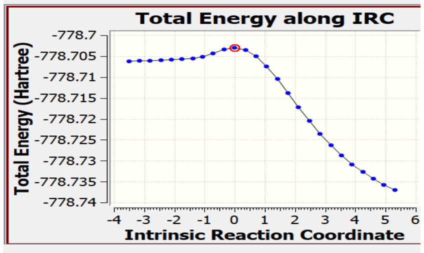

10.1.20. Transition state barrier for ^{3}1Nb forming ^{3}Br2b

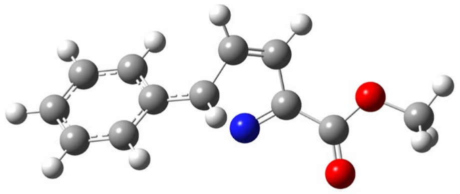

Energy (B3LYP-D/6-31+G(d,p)): -669.008374 Hartrees
Imaginary Frequency: -761 cm^{-1} (1240)
Standard Orientation

Center Number	Atomic Number	Atomic Type	Coordinates (Angstroms) X	Y	Z
1	6	0	1.636557	0.497678	-0.263782
2	6	0	1.144210	1.804818	0.155346
3	6	0	-0.062300	1.711810	0.766905
4	6	0	-0.661656	0.353625	0.907394
5	7	0	0.637311	-0.321397	-0.525646
6	1	0	1.718211	2.717325	0.039578
7	6	0	-1.981764	0.043978	0.420599
8	6	0	-2.579714	0.803405	-0.614143
9	6	0	-3.843117	0.474796	-1.096110
10	6	0	-4.541004	-0.615920	-0.562508
11	6	0	-3.959171	-1.384099	0.456907
12	6	0	-2.696243	-1.062915	0.939482
13	1	0	-2.035308	1.640733	-1.040388
14	1	0	-4.286461	1.065238	-1.892661
15	1	0	-5.527595	-0.868919	-0.939043
16	1	0	-4.495275	-2.233866	0.869301
17	1	0	-2.244393	-1.663716	1.724452
18	6	0	3.053602	0.122788	-0.130230
19	6	0	4.625645	-1.637170	-0.230958
20	8	0	3.949864	0.931587	0.070336
21	8	0	3.254848	-1.203648	-0.303817
22	1	0	5.220664	-1.154708	-1.011170
23	1	0	5.054373	-1.394879	0.745410
24	1	0	4.594808	-2.716024	-0.382073
25	1	0	-0.586538	2.576345	1.167947
26	1	0	-0.321637	-0.218024	1.772605

10.1.21. Transition state barrier for T of 1b-D forming ^{3}1Nb

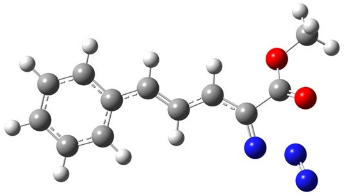

Energy (B3LYP-D/6-31+G(d,p)): -778.538363 Hartrees
Imaginary Frequency: -311 cm^{-1} (29)
Standard Orientation

Center Number	Atomic Number	Atomic Type	Coordinates (Angstroms) X	Y	Z
1	6	0	-5.029102	-1.407860	-0.127086
2	6	0	-3.660369	-1.633872	-0.239329
3	6	0	-2.728577	-0.578946	-0.088002
4	6	0	-3.232877	0.718473	0.176902
5	6	0	-4.600807	0.939640	0.287921
6	6	0	-5.506770	-0.119515	0.137761
7	1	0	-5.724603	-2.233263	-0.246073
8	1	0	-3.294304	-2.636390	-0.445308
9	1	0	-2.551832	1.555060	0.292120
10	1	0	-4.967140	1.941583	0.490615
11	1	0	-6.574035	0.060074	0.225095
12	6	0	-1.318906	-0.871903	-0.210972
13	6	0	-0.269996	0.025309	-0.068188
14	6	0	1.064304	-0.370749	-0.202308
15	6	0	2.247968	0.434852	-0.120796
16	6	0	3.584852	-0.301698	-0.141057
17	6	0	4.702607	-2.284407	0.494012
18	8	0	4.605177	0.129981	-0.625080
19	8	0	3.485147	-1.508523	0.459273
20	1	0	4.442040	-3.207288	1.010955
21	1	0	5.049469	-2.489470	-0.521908
22	1	0	5.480036	-1.740091	1.035506
23	7	0	2.436061	1.728567	-0.033387
24	7	0	1.161054	2.540795	-0.208857
25	7	0	1.004270	3.553085	0.347967
26	1	0	-1.066736	-1.906587	-0.441840
27	1	0	-0.489399	1.059985	0.159319
28	1	0	1.250248	-1.428353	-0.365241

10.2. Calculations for 1a

10.2.1. Optimization of 1a-A

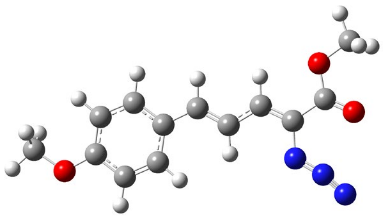

Energy (B3LYP-D/6-31+G(d,p)): -893.126945 Hartrees
Standard Orientation

Center Number	Atomic Number	Atomic Type	Coordinates (Angstroms) X	Y	Z
1	6	0	-4.477420	-1.086663	0.000160
2	6	0	-3.120488	-1.414632	0.000150
3	6	0	-2.112934	-0.433519	0.000044
4	6	0	-2.527373	0.919982	-0.000051
5	6	0	-3.868429	1.261945	-0.000042
6	6	0	-4.858216	0.261444	0.000063
7	1	0	-5.215375	-1.879476	0.000242
8	1	0	-2.837423	-2.464279	0.000225
9	1	0	-1.787963	1.714098	-0.000133
10	1	0	-4.184040	2.300047	-0.000115
11	6	0	-0.716591	-0.846701	0.000039
12	6	0	0.384277	-0.048193	-0.000060
13	6	0	1.716097	-0.579436	-0.000051
14	6	0	2.862825	0.162928	-0.000148
15	6	0	4.210508	-0.458282	-0.000131
16	6	0	5.462250	-2.460113	0.000441
17	8	0	5.248625	0.182411	-0.000015
18	8	0	4.175838	-1.809658	0.000179
19	1	0	5.244153	-3.527690	0.000671
20	1	0	6.030798	-2.180149	-0.890043
21	1	0	6.030646	-2.179737	0.890892
22	7	0	2.753086	1.564257	-0.000249
23	7	0	3.740004	2.316643	-0.000354
24	7	0	4.486520	3.177202	-0.000488
25	1	0	-0.554666	-1.925254	0.000126
26	1	0	0.283978	1.033316	-0.000150
27	1	0	1.835408	-1.657623	0.000039
28	8	0	-6.145716	0.706393	0.000063
29	6	0	-7.200675	-0.251026	0.000168
30	1	0	-7.165100	-0.883059	0.896606
31	1	0	-7.165150	-0.883202	-0.896171
32	1	0	-8.125485	0.326717	0.000148

10.2.2. Optimization of 1a-B

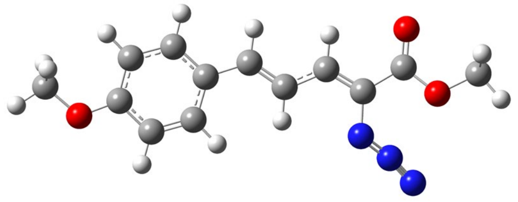

Energy (B3LYP/6-31+G(d)): -893.126155 Hartrees
Standard Orientation

Center Number	Atomic Number	Atomic Type	Coordinates (Angstroms) X	Y	Z
1	6	0	4.593451	-1.031551	-0.042864
2	6	0	3.251312	-1.414942	-0.059412
3	6	0	2.204298	-0.477132	-0.012180
4	6	0	2.562264	0.890802	0.054228
5	6	0	3.888044	1.287541	0.071415
6	6	0	4.918159	0.329664	0.022962
7	1	0	5.363669	-1.792164	-0.081209
8	1	0	3.011567	-2.474062	-0.110669
9	1	0	1.790517	1.652587	0.093253
10	1	0	4.160787	2.336512	0.122413
11	6	0	0.826566	-0.947640	-0.032512
12	6	0	-0.306682	-0.196467	0.002402
13	6	0	-1.612890	-0.785098	-0.027950
14	6	0	-2.794834	-0.104548	0.007939
15	6	0	-4.060097	-0.889050	-0.012462
16	6	0	-6.420907	-0.781619	0.156111
17	8	0	-4.122392	-2.095578	-0.169572
18	8	0	-5.147059	-0.107327	0.170572
19	1	0	-7.163232	0.003348	0.296255
20	1	0	-6.567365	-1.292052	-0.798973
21	1	0	-6.468826	-1.513634	0.966147
22	7	0	-2.754733	1.301421	0.137285
23	7	0	-3.726314	2.029765	-0.092110
24	7	0	-4.505900	2.846633	-0.253614
25	1	0	0.708986	-2.030611	-0.082947
26	1	0	-0.252472	0.887319	0.052895
27	1	0	-1.694046	-1.866949	-0.082176
28	8	0	6.186074	0.826532	0.045079
29	6	0	7.279253	-0.085982	-0.001885
30	1	0	7.267482	-0.674331	-0.928177
31	1	0	7.271255	-0.762030	0.862529
32	1	0	8.179687	0.528448	0.026329

10.2.3. Optimization of 1a-C

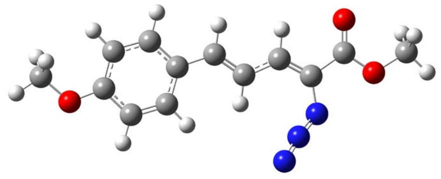

Energy (B3LYP-D/6-31+G(d,p) = -893.121120 Hartree
Standard Orientation

Center Number	Atomic Number	Atomic Type	Coordinates (Angstroms) X	Y	Z
1	6	0	4.329480	-1.203950	0.094516
2	6	0	2.969693	-1.511823	0.156891
3	6	0	1.973730	-0.523313	0.060590
4	6	0	2.402498	0.816982	-0.095576
5	6	0	3.746877	1.138725	-0.158650
6	6	0	4.724974	0.130647	-0.066132
7	1	0	5.058717	-2.001095	0.171525
8	1	0	2.674566	-2.550731	0.280618
9	1	0	1.673618	1.618472	-0.160500
10	1	0	4.074221	2.166519	-0.275547
11	6	0	0.574251	-0.917075	0.126384
12	6	0	-0.517358	-0.117591	-0.017232
13	6	0	-1.851850	-0.637817	0.066616
14	6	0	-3.035414	0.031305	-0.044435
15	6	0	-4.295261	-0.766315	-0.023641
16	6	0	-6.648689	-0.717198	-0.129639
17	8	0	-4.329465	-1.981478	0.082064
18	8	0	-5.398852	-0.001906	-0.132903
19	1	0	-7.417873	0.047894	-0.231534
20	1	0	-6.687370	-1.421148	-0.965154
21	1	0	-6.769768	-1.268152	0.806780
22	7	0	-3.236457	1.417875	-0.232433
23	7	0	-2.397637	2.247035	0.128142
24	7	0	-1.738243	3.138943	0.406743
25	1	0	0.398231	-1.977329	0.309635
26	1	0	-0.373014	0.937828	-0.219166
27	1	0	-1.959966	-1.709015	0.218523
28	8	0	6.016279	0.555103	-0.139186
29	6	0	7.060932	-0.409736	-0.046150
30	1	0	7.002589	-1.139748	-0.863353
31	1	0	7.033963	-0.933896	0.917530
32	1	0	7.991566	0.152627	-0.127580

10.2.4. Optimization of 1a-D

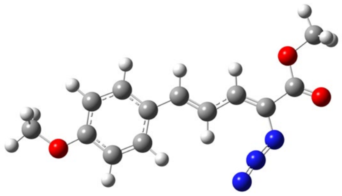

Energy (B3LYP-D/6-31+G(d,p) = -893.119561 Hartree
Standard Orientation

Center Number	Atomic Number	Atomic Type	Coordinates (Angstroms) X	Y	Z
1	6	0	4.187086	-1.277090	0.101126
2	6	0	2.813733	-1.518208	0.161390
3	6	0	1.867415	-0.482409	0.062939
4	6	0	2.361210	0.835538	-0.091035
5	6	0	3.719775	1.091234	-0.152189
6	6	0	4.647425	0.036652	-0.058766
7	1	0	4.876548	-2.108777	0.179223
8	1	0	2.468221	-2.541613	0.284294
9	1	0	1.672452	1.671819	-0.154974
10	1	0	4.096980	2.101998	-0.267194
11	6	0	0.449784	-0.806841	0.124097
12	6	0	-0.599963	0.043036	-0.037398
13	6	0	-1.961414	-0.407732	0.041721
14	6	0	-3.103477	0.329808	-0.079854
15	6	0	-4.455762	-0.298402	-0.085423
16	6	0	-5.673859	-2.318705	0.028595
17	8	0	-5.500142	0.316480	-0.171854
18	8	0	-4.398537	-1.650499	0.014595
19	1	0	-5.442725	-3.380021	0.118342
20	1	0	-6.273987	-1.979316	0.876971
21	1	0	-6.219661	-2.117763	-0.896902
22	7	0	-3.210128	1.725714	-0.271474
23	7	0	-2.336859	2.499138	0.126968
24	7	0	-1.634389	3.347024	0.435458
25	1	0	0.221847	-1.855348	0.318461
26	1	0	-0.402061	1.086851	-0.252933
27	1	0	-2.113615	-1.471127	0.199855
28	8	0	5.957905	0.397714	-0.129565
29	6	0	6.954379	-0.616533	-0.033947
30	1	0	6.862389	-1.343331	-0.850933
31	1	0	6.900127	-1.138184	0.929956
32	1	0	7.911322	-0.099979	-0.113768

10.2.5. Optimization of T of 1a-A

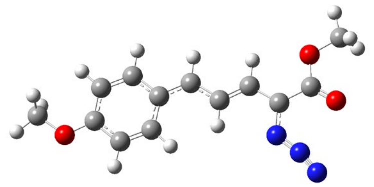

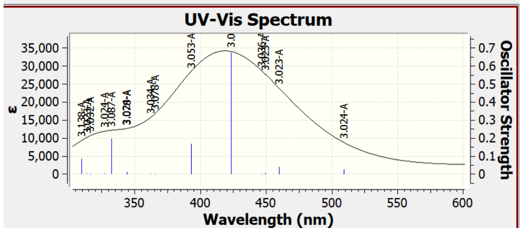

Energy (B3LYP-D/6-31+G(d,p) = -893.070993 Hartree
Standard Orientation

Center Number	Atomic Number	Atomic Type	Coordinates (Angstroms) X	Y	Z
1	6	0	-4.508009	-1.090181	0.000179
2	6	0	-3.174704	-1.474444	0.000214
3	6	0	-2.106740	-0.528205	0.000084
4	6	0	-2.483642	0.853961	-0.000088
5	6	0	-3.807991	1.240373	-0.000124
6	6	0	-4.838721	0.277645	0.000009
7	1	0	-5.279065	-1.851382	0.000283
8	1	0	-2.932374	-2.533892	0.000347
9	1	0	-1.718750	1.622607	-0.000192
10	1	0	-4.085728	2.289516	-0.000254
11	6	0	-0.765530	-0.980892	0.000129
12	6	0	0.408776	-0.154662	0.000011
13	6	0	1.680204	-0.651031	0.000069
14	6	0	2.887556	0.156373	-0.000048
15	6	0	4.231377	-0.409818	0.000029
16	6	0	5.534335	-2.381216	0.000286
17	8	0	5.258123	0.265398	-0.000078
18	8	0	4.231944	-1.768554	0.000212
19	1	0	5.348416	-3.455127	0.000433
20	1	0	6.096172	-2.085479	-0.889859
21	1	0	6.096184	-2.085238	0.890344
22	7	0	2.719214	1.519098	-0.000241
23	7	0	3.671639	2.327432	-0.000356
24	7	0	4.358346	3.238174	-0.000483
25	1	0	-0.607471	-2.058054	0.000268
26	1	0	0.284979	0.925086	-0.000133
27	1	0	1.841910	-1.723611	0.000213
28	8	0	-6.107985	0.771779	-0.000041
29	6	0	-7.200733	-0.142510	0.000086
30	1	0	-7.190423	-0.775245	0.896606
31	1	0	-7.190442	-0.775473	-0.896273
32	1	0	-8.101537	0.472035	0.000018

10.2.6. Optimization of T of 1a-B

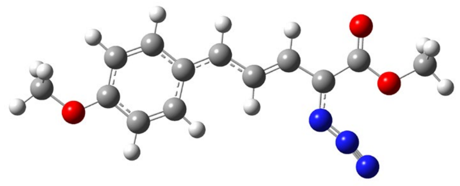

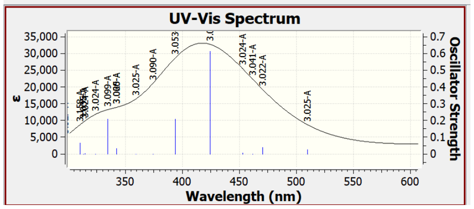

Energy (B3LYP-D/6-31+G(d)): - 893.069639 Hartrees

Standard Orientation

Center Number	Atomic Number	Atomic Type	Coordinates (Angstroms) X	Y	Z
1	6	0	-4.624242	-1.026583	0.000207
2	6	0	-3.309500	-1.469904	0.000244
3	6	0	-2.200228	-0.572077	0.000111
4	6	0	-2.515375	0.825611	-0.000063
5	6	0	-3.821244	1.270612	-0.000100
6	6	0	-4.893754	0.354640	0.000034
7	1	0	-5.428455	-1.752699	0.000313
8	1	0	-3.114441	-2.539016	0.000378
9	1	0	-1.716956	1.559397	-0.000169
10	1	0	-4.052156	2.331065	-0.000232
11	6	0	-0.881003	-1.084584	0.000156
12	6	0	0.329551	-0.311545	0.000034
13	6	0	1.576558	-0.865017	0.000086
14	6	0	2.821224	-0.118068	-0.000033
15	6	0	4.083769	-0.854940	0.000046
16	6	0	6.450264	-0.698004	-0.000003
17	8	0	4.161268	-2.078205	0.000211
18	8	0	5.171303	-0.037640	-0.000076
19	1	0	7.189034	0.103110	-0.000118
20	1	0	6.554645	-1.323237	0.890588
21	1	0	6.554624	-1.323474	-0.890430
22	7	0	2.714179	1.254054	-0.000215
23	7	0	3.673114	2.043996	-0.000335
24	7	0	4.405426	2.920422	-0.000464
25	1	0	-0.770554	-2.167492	0.000294
26	1	0	0.253427	0.772858	-0.000108
27	1	0	1.699863	-1.943380	0.000227
28	8	0	-6.139971	0.904663	-0.000017
29	6	0	-7.271893	0.039329	0.000111
30	1	0	-7.289407	-0.593250	0.896610
31	1	0	-7.289433	-0.593481	-0.896225
32	1	0	-8.144786	0.692925	0.000040

10.2.7. Optimization of T of 1a-C

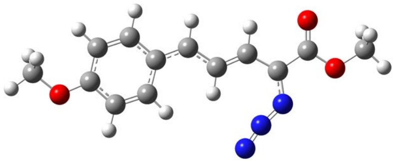

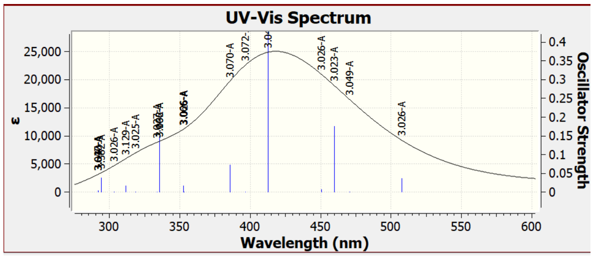

Energy (B3LYP-D/6-31+G(d,p)): -893.065450 Hartrees
Standard Orientation

Center Number	Atomic Number	Atomic Type	Coordinates (Angstroms) X	Y	Z
1	6	0	4.329480	-1.203950	0.094516
2	6	0	2.969693	-1.511823	0.156891
3	6	0	1.973730	-0.523313	0.060590
4	6	0	2.402498	0.816982	-0.095576
5	6	0	3.746877	1.138725	-0.158650
6	6	0	4.724974	0.130647	-0.066132
7	1	0	5.058717	-2.001095	0.171525
8	1	0	2.674566	-2.550731	0.280618
9	1	0	1.673618	1.618472	-0.160500
10	1	0	4.074221	2.166519	-0.275547
11	6	0	0.574251	-0.917075	0.126384
12	6	0	-0.517358	-0.117591	-0.017232
13	6	0	-1.851850	-0.637817	0.066616
14	6	0	-3.035414	0.031305	-0.044435
15	6	0	-4.295261	-0.766315	-0.023641
16	6	0	-6.648689	-0.717198	-0.129639
17	8	0	-4.329465	-1.981478	0.082064
18	8	0	-5.398852	-0.001906	-0.132903
19	1	0	-7.417873	0.047894	-0.231534
20	1	0	-6.687370	-1.421148	-0.965154
21	1	0	-6.769768	-1.268152	0.806780
22	7	0	-3.236457	1.417875	-0.232433
23	7	0	-2.397637	2.247035	0.128142
24	7	0	-1.738243	3.138943	0.406743
25	1	0	0.398231	-1.977329	0.309635
26	1	0	-0.373014	0.937828	-0.219166
27	1	0	-1.959966	-1.709015	0.218523
28	8	0	6.016279	0.555103	-0.139186
29	6	0	7.060932	-0.409736	-0.046150
30	1	0	7.002589	-1.139748	-0.863353
31	1	0	7.033963	-0.933896	0.917530
32	1	0	7.991566	0.152627	-0.127580

10.2.8. Optimization of T of 1a-D

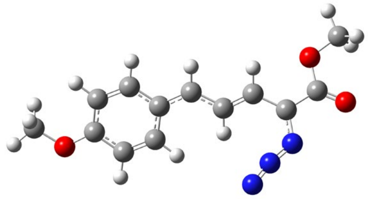

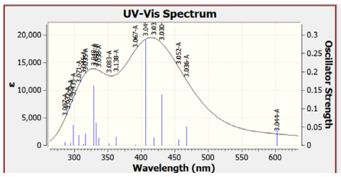

Energy (B3LYP-D/6-31+G(d,p)): -893.064423 Hatrees
Standard Orientation

Center Number	Atomic Number	Atomic Type	Coordinates (Angstroms) X	Y	Z
1	6	0	4.197498	-1.119314	0.480531
2	6	0	2.852923	-1.359361	0.735067
3	6	0	1.823496	-0.502546	0.256744
4	6	0	2.241078	0.633329	-0.502541
5	6	0	3.575347	0.879128	-0.757589
6	6	0	4.571319	0.007523	-0.272172
7	1	0	4.941255	-1.805487	0.867487
8	1	0	2.576713	-2.233385	1.319012
9	1	0	1.504194	1.329148	-0.888876
10	1	0	3.885973	1.745042	-1.333135
11	6	0	0.464293	-0.804949	0.550314
12	6	0	-0.675845	-0.054415	0.142055
13	6	0	-1.961074	-0.372939	0.486134
14	6	0	-3.149166	0.389150	0.097886
15	6	0	-4.430378	-0.250870	-0.218474
16	6	0	-5.535129	-2.329423	-0.444119
17	8	0	-5.469180	0.328027	-0.495031
18	8	0	-4.324278	-1.608564	-0.156271
19	1	0	-5.273624	-3.383711	-0.353268
20	1	0	-6.319617	-2.061865	0.269194
21	1	0	-5.883719	-2.102863	-1.455406
22	7	0	-3.204658	1.755677	0.023625
23	7	0	-2.250550	2.469335	0.384563
24	7	0	-1.465557	3.270457	0.622821
25	1	0	0.277351	-1.689483	1.156860
26	1	0	-0.507386	0.814933	-0.488699
27	1	0	-2.153193	-1.282481	1.049352
28	8	0	5.852687	0.344349	-0.584293
29	6	0	6.913445	-0.489078	-0.124585
30	1	0	6.827354	-1.504103	-0.532463
31	1	0	6.937649	-0.533987	0.971500
32	1	0	7.831725	-0.027950	-0.489372

10.2.9. Optimization of ^{3}Br1a-A

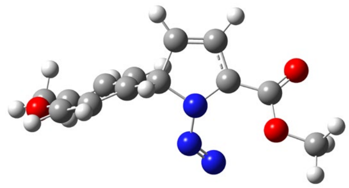

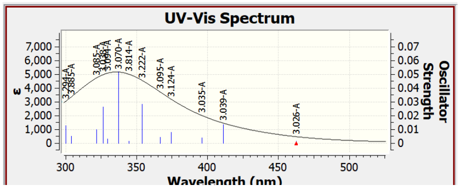

Energy (B3LYP-D/6-31+G(d,p)): -893.076393 Hartrees
Standard Orientation

Center Number	Atomic Number	Atomic Type	Coordinates (Angstroms) X	Y	Z
1	6	0	-2.192720	0.622900	0.313300
2	6	0	-1.811762	1.873563	0.877058
3	6	0	-0.687573	2.337928	0.263826
4	6	0	-0.202464	1.350297	-0.767632
5	7	0	-1.266989	0.305282	-0.682725
6	1	0	-2.395035	2.363663	1.645483
7	6	0	1.192339	0.807439	-0.511014
8	6	0	1.519608	0.244767	0.726000
9	6	0	2.793316	-0.270305	0.972618
10	6	0	3.768290	-0.214318	-0.035059
11	6	0	3.451997	0.353587	-1.277770
12	6	0	2.174509	0.852931	-1.508808
13	1	0	0.769001	0.199947	1.510440
14	1	0	3.010595	-0.705629	1.940253
15	1	0	4.217351	0.383205	-2.046077
16	1	0	1.936272	1.275549	-2.481543
17	6	0	-3.464329	-0.037781	0.585267
18	6	0	-5.086867	-1.575672	-0.182986
19	8	0	-4.101890	0.154242	1.613228
20	8	0	-3.877983	-0.832033	-0.424558
21	1	0	-4.965724	-2.220363	0.691514
22	1	0	-5.926285	-0.895696	-0.015640
23	1	0	-5.241948	-2.171219	-1.081961
24	7	0	-0.919671	-0.956616	-1.156074
25	7	0	-1.444125	-1.974550	-0.829828
26	1	0	-0.174073	3.271427	0.450386
27	1	0	-0.230515	1.790340	-1.775279
28	8	0	5.043192	-0.682170	0.095253
29	6	0	5.425340	-1.291409	1.324607
30	1	0	6.465665	-1.592111	1.195578
31	1	0	5.351778	-0.584551	2.161057
32	1	0	4.813760	-2.177255	1.538351

10.2.10. Optimization of ^{3}Br1a-B

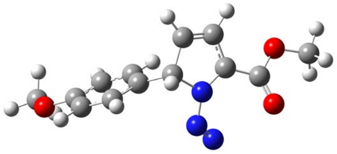

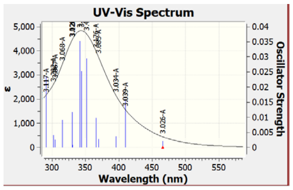

Energy (B3LYP-D/6-31+G(d,p)): - 893.075373 Hartrees
Standard Orientation

Center Number	Atomic Number	Atomic Type	Coordinates (Angstroms) X	Y	Z
1	6	0	2.195693	0.341994	-0.244959
2	6	0	1.922826	1.039516	-1.459556
3	6	0	0.776998	1.764429	-1.332054
4	6	0	0.167546	1.550065	0.030789
5	7	0	1.182151	0.656755	0.655756
6	1	0	2.582632	1.002719	-2.315805
7	6	0	-1.227277	0.948200	0.006492
8	6	0	-1.471914	-0.250848	-0.668661
9	6	0	-2.745956	-0.820624	-0.693631
10	6	0	-3.804697	-0.174334	-0.037479
11	6	0	-3.570973	1.032062	0.638156
12	6	0	-2.292511	1.579963	0.660587
13	1	0	-0.655657	-0.755290	-1.178653
14	1	0	-2.898505	-1.755026	-1.219637
15	1	0	-4.400480	1.513571	1.145104
16	1	0	-2.120007	2.506277	1.202560
17	6	0	3.440784	-0.318965	0.124883
18	6	0	5.422506	-1.280872	-0.734229
19	8	0	3.844504	-0.498278	1.262355
20	8	0	4.138242	-0.681982	-0.988251
21	1	0	6.070034	-0.584572	-0.194085
22	1	0	5.308088	-2.192257	-0.141468
23	1	0	5.835294	-1.508513	-1.716956
24	7	0	0.766031	-0.060448	1.776351
25	7	0	1.277843	-1.062987	2.163700
26	1	0	0.321964	2.411533	-2.069711
27	1	0	0.135655	2.491508	0.599012
28	8	0	-5.087910	-0.636564	0.001812
29	6	0	-5.389156	-1.871883	-0.639571
30	1	0	-6.452456	-2.043389	-0.468196
31	1	0	-5.198479	-1.819026	-1.719244
32	1	0	-4.812150	-2.698392	-0.205504

10.2.11. Optimization of ^{3}Br2a-A

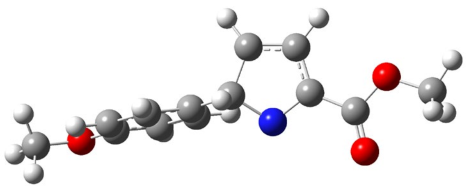

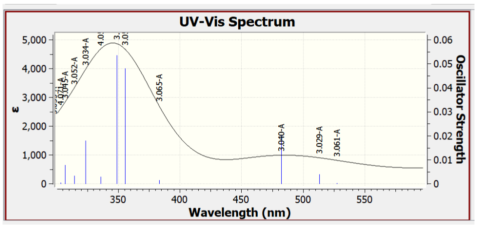

Energy (B3LYP-D/6-31+G(d,p)): -783.579423 Hartrees
Standard Orientation

Center Number	Atomic Number	Atomic Type	Coordinates (Angstroms) X	Y	Z
1	6	0	2.489658	0.179876	-0.021508
2	6	0	2.233930	1.261174	-0.859362
3	6	0	0.889426	1.618022	-0.698452
4	6	0	0.307431	0.791215	0.444336
5	7	0	1.260687	-0.331979	0.466653
6	1	0	2.952956	1.720242	-1.526642
7	6	0	-1.127135	0.368001	0.237432
8	6	0	-2.158234	0.933878	0.988237
9	6	0	-3.496055	0.581245	0.775059
10	6	0	-3.808152	-0.364885	-0.209525
11	6	0	-2.778464	-0.945901	-0.968732
12	6	0	-1.458366	-0.579086	-0.747313
13	1	0	-1.924456	1.662632	1.760904
14	1	0	-4.268962	1.039401	1.380005
15	1	0	-3.041282	-1.683182	-1.720174
16	1	0	-0.665149	-1.041939	-1.326684
17	6	0	3.755535	-0.478095	0.276901
18	6	0	6.086480	-0.453673	-0.131122
19	8	0	3.894249	-1.435966	1.019929
20	8	0	4.792176	0.123753	-0.375858
21	1	0	6.336235	-0.398503	0.932133
22	1	0	6.105491	-1.500933	-0.445343
23	1	0	6.785593	0.138382	-0.721944
24	1	0	0.309164	2.304650	-1.302498
25	1	0	0.390048	1.350546	1.396718
26	8	0	-5.071639	-0.789839	-0.504847
27	6	0	-6.161849	-0.255194	0.237740
28	1	0	-6.069477	-0.487169	1.306640
29	1	0	-6.241848	0.831627	0.105072
30	1	0	-7.056628	-0.734956	-0.160689

10.2.12. Optimization of ^{3}Br2a-B

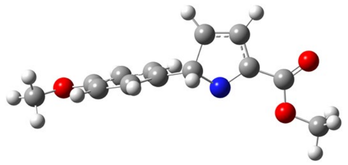

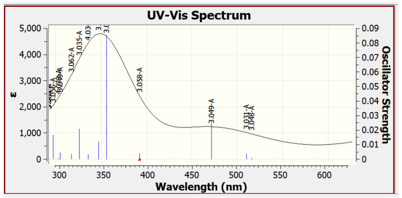

Energy (B3LYP-D/6-31+G(d,p)): -783.580840 Hartrees
Standard Orientation

Center Number	Atomic Number	Atomic Type	Coordinates (Angstroms) X	Y	Z
1	6	0	2.546644	0.515900	-0.092684
2	6	0	2.194702	1.797081	-0.503700
3	6	0	0.842656	1.988758	-0.193456
4	6	0	0.360505	0.786193	0.612702
5	7	0	1.372078	-0.213712	0.220880
6	1	0	2.861706	2.497754	-0.991395
7	6	0	-1.057638	0.363522	0.313489
8	6	0	-2.075685	0.546655	1.250050
9	6	0	-3.401112	0.196154	0.967066
10	6	0	-3.713577	-0.356174	-0.281632
11	6	0	-2.696780	-0.550095	-1.231837
12	6	0	-1.389488	-0.190580	-0.935342
13	1	0	-1.840947	0.967367	2.225056
14	1	0	-4.163883	0.350709	1.720450
15	1	0	-2.959251	-0.986070	-2.190158
16	1	0	-0.605340	-0.354275	-1.668489
17	6	0	3.891803	-0.047295	-0.112334
18	6	0	5.222598	-1.943899	0.339418
19	8	0	4.884691	0.572473	-0.474588
20	8	0	3.922652	-1.328089	0.324061
21	1	0	5.644611	-1.972034	-0.669024
22	1	0	5.902165	-1.389891	0.993099
23	1	0	5.059613	-2.953174	0.717064
24	1	0	0.199784	2.802456	-0.505648
25	1	0	0.459876	0.991938	1.696331
26	8	0	-4.965328	-0.739121	-0.669649
27	6	0	-6.041716	-0.581604	0.248252
28	1	0	-5.879078	-1.170788	1.160024
29	1	0	-6.188527	0.472923	0.515728
30	1	0	-6.928863	-0.950731	-0.267744

10.2.13. Optimization of Nitrene ^{3}1Na-A

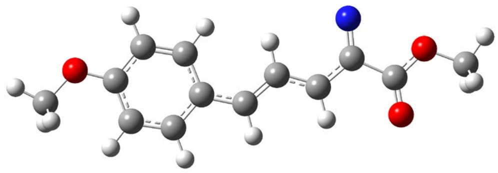

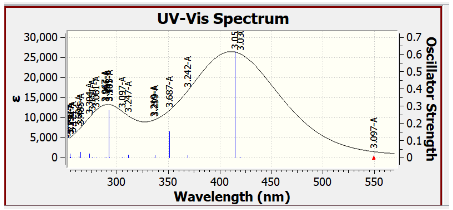

Energy (B3LYP-D/6-31+G(d,p)): -783.605306 Hartrees
Standard Orientation

Center Number	Atomic Number	Atomic Type	Coordinates (Angstroms) X	Y	Z
1	6	0	4.083151	-1.047202	0.000051
2	6	0	2.723316	-1.352311	-0.000061
3	6	0	1.728087	-0.351535	-0.000214
4	6	0	2.169345	0.997894	-0.000352
5	6	0	3.515216	1.314457	-0.000237
6	6	0	4.487775	0.295689	0.000012
7	1	0	4.808028	-1.852068	0.000094
8	1	0	2.421326	-2.396509	-0.000011
9	1	0	1.445475	1.805952	-0.000598
10	1	0	3.849914	2.346567	-0.000375
11	6	0	0.336662	-0.738209	-0.000195
12	6	0	-0.765200	0.098963	-0.000077
13	6	0	-2.080133	-0.379137	-0.000084
14	6	0	-3.241840	0.467761	0.000038
15	6	0	-4.588858	-0.253114	0.000026
16	6	0	-6.931126	0.010317	0.000188
17	8	0	-4.704324	-1.462224	-0.000126
18	8	0	-5.616329	0.605343	0.000200
19	1	0	-7.624419	0.850425	0.000327
20	1	0	-7.067582	-0.607182	-0.891225
21	1	0	-7.067497	-0.607406	0.891459
22	7	0	-3.221625	1.756803	0.000107
23	1	0	0.144314	-1.811055	-0.000258
24	1	0	-0.623227	1.177044	0.000051
25	1	0	-2.272133	-1.448621	-0.000189
26	8	0	5.781584	0.717299	0.000158
27	6	0	6.821127	-0.257839	0.000368
28	1	0	7.754973	0.305042	0.000332
29	1	0	6.774771	-0.888599	0.897051
30	1	0	6.774843	-0.888943	-0.896041

10.2.14. Optimization of T_A of 1a

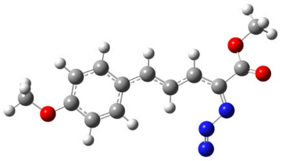

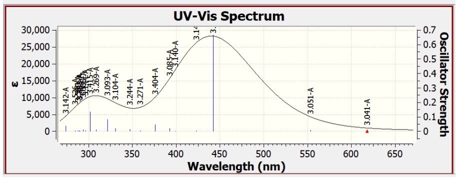

Energy (B3LYP-D/6-31+G(d,p)): -893.074307 Hartrees
Standard Orientation

Center Number	Atomic Number	Atomic Type	Coordinates (Angstroms) X	Y	Z
1	6	0	-4.189928	-1.266510	-0.168277
2	6	0	-2.823443	-1.516183	-0.256636
3	6	0	-1.860920	-0.491636	-0.111447
4	6	0	-2.344386	0.823841	0.126944
5	6	0	-3.697719	1.084371	0.216611
6	6	0	-4.636551	0.042811	0.071109
7	1	0	-4.889278	-2.085147	-0.285576
8	1	0	-2.487277	-2.533026	-0.442098
9	1	0	-1.647948	1.647984	0.238847
10	1	0	-4.066230	2.088573	0.397520
11	6	0	-0.463009	-0.819964	-0.210250
12	6	0	0.607379	0.053694	-0.072442
13	6	0	1.933572	-0.376705	-0.180756
14	6	0	3.129142	0.405082	-0.106161
15	6	0	4.457315	-0.340099	-0.094059
16	6	0	5.528325	-2.376511	0.442792
17	8	0	5.511376	0.117198	-0.471794
18	8	0	4.317797	-1.592853	0.398970
19	1	0	5.235366	-3.337930	0.863540
20	1	0	5.937511	-2.498705	-0.563354
21	1	0	6.272745	-1.885442	1.074336
22	7	0	3.322837	1.704907	-0.047158
23	7	0	2.134227	2.500794	-0.244352
24	7	0	1.941468	3.510153	0.332845
25	1	0	-0.233061	-1.864840	-0.418056
26	1	0	0.409902	1.098107	0.129828
27	1	0	2.096525	-1.441417	-0.317916
28	8	0	-5.939307	0.409201	0.177170
29	6	0	-6.950866	-0.587433	0.038907
30	1	0	-6.856659	-1.358059	0.813872
31	1	0	-7.899271	-0.063707	0.159982
32	1	0	-6.914443	-1.054291	-0.953054

10.2.15. Optimization of 2a-A

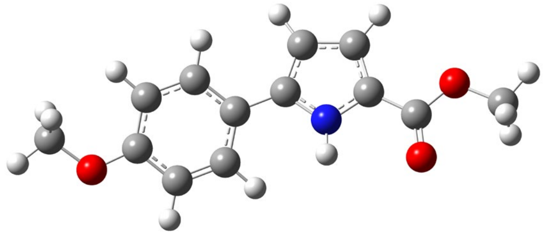

Energy (B3LYP-D/6-31+G(d,p)): -783.715088 Hartrees
Standard Orientation

Center Number	Atomic Number	Atomic Type	Coordinates (Angstroms) X	Y	Z
1	6	0	2.537272	0.320388	-0.048538
2	6	0	2.309901	1.674560	-0.272539
3	6	0	0.912391	1.860927	-0.322720
4	6	0	0.302836	0.615359	-0.129809
5	7	0	1.308208	-0.299420	0.027552
6	1	0	1.208282	-1.279518	0.250926
7	1	0	3.080024	2.421913	-0.394439
8	1	0	0.389331	2.787246	-0.513117
9	6	0	-1.113062	0.247463	-0.100158
10	6	0	-2.086413	1.188351	0.267541
11	6	0	-3.444291	0.864325	0.297199
12	6	0	-3.855507	-0.433698	-0.035388
13	6	0	-2.895573	-1.389029	-0.403367
14	6	0	-1.549823	-1.049866	-0.439036
15	1	0	-1.779096	2.189408	0.554134
16	1	0	-4.160778	1.621582	0.591484
17	1	0	-3.230679	-2.385863	-0.670194
18	1	0	-0.832306	-1.798174	-0.764016
19	6	0	3.739215	-0.479500	0.114585
20	6	0	6.100553	-0.477760	0.170690
21	8	0	3.731191	-1.687795	0.320649
22	8	0	4.871675	0.255405	0.016100
23	1	0	6.143528	-0.950431	1.155690
24	1	0	6.185654	-1.249883	-0.598769
25	1	0	6.893838	0.261931	0.063450
26	8	0	-5.151146	-0.864118	-0.034492
27	6	0	-6.175452	0.059542	0.317857
28	1	0	-7.112462	-0.493723	0.244348
29	1	0	-6.048332	0.425201	1.345060
30	1	0	-6.200500	0.911946	-0.373392

10.2.16. Optimization of 2a-B

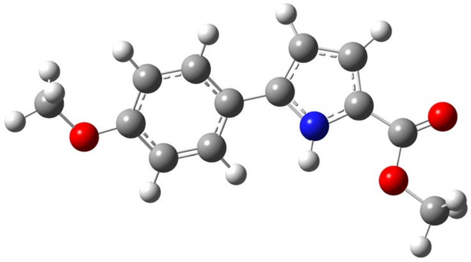

Energy (B3LYP-D/6-31+G(d,p)): -783.713311 Hartrees
Standard Orientation

Center Number	Atomic Number	Atomic Type	Coordinates (Angstroms) X	Y	Z
1	6	0	2.603463	0.653053	-0.060600
2	6	0	2.285378	1.997621	-0.213980
3	6	0	0.878895	2.096207	-0.249023
4	6	0	0.351975	0.806694	-0.118473
5	7	0	1.415094	-0.050516	-0.012552
6	1	0	1.364572	-1.042041	0.165932
7	1	0	3.009221	2.794783	-0.300246
8	1	0	0.296063	2.995170	-0.389897
9	6	0	-1.036885	0.346378	-0.100718
10	6	0	-2.064520	1.197663	0.332141
11	6	0	-3.398244	0.784778	0.352553
12	6	0	-3.729277	-0.514427	-0.056289
13	6	0	-2.714328	-1.380841	-0.490729
14	6	0	-1.393710	-0.953108	-0.516299
15	1	0	-1.818568	2.197095	0.677516
16	1	0	-4.158348	1.474543	0.698669
17	1	0	-2.988149	-2.378632	-0.817292
18	1	0	-0.633707	-1.630867	-0.895365
19	6	0	3.906941	0.018154	0.045667
20	6	0	5.033399	-2.053432	0.315312
21	8	0	4.980821	0.593540	0.008002
22	8	0	3.790827	-1.336411	0.199448
23	1	0	5.641269	-1.911663	-0.582215
24	1	0	5.596342	-1.704418	1.185046
25	1	0	4.756128	-3.101343	0.431159
26	8	0	-4.994315	-1.027622	-0.071710
27	6	0	-6.072696	-0.195481	0.342635
28	1	0	-6.972665	-0.803136	0.241401
29	1	0	-5.959098	0.114970	1.389363
30	1	0	-6.159729	0.693541	-0.295315

10.3. Spin Density Calculations

10.3.1. Spin Density T of 1a-A

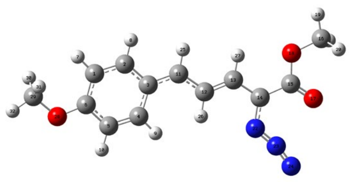

C	1	0.07393
C	2	-0.19495
C	3	0.07116
C	4	-0.16587
C	5	0.07638
C	6	-0.19228
H	7	-0.00198
H	8	0.00612
H	9	0.00516
H	10	-0.00209
H	11	-0.49687
C	12	-0.10225
C	13	-0.17016
C	14	-0.43327
C	15	-0.03062
C	16	0.00224
C	17	-0.12163
O	18	-0.03069
O	19	0
H	20	-0.00152
H	21	-0.00152
H	22	-0.02719
N	23	0.00327
N	24	-0.2339
N	25	0.01446
H	26	0.0029
H	27	0.00538
H	28	-0.05121
O	29	0.00298
C	30	-0.00305

10.3.2. Spin Density T_A of 1a

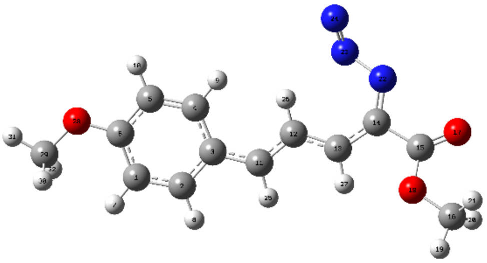

C	1	-0.04207
C	2	0.11207
C	3	-0.06056
C	4	0.08853
C	5	-0.04296
C	6	0.10022
H	7	0.00111
H	8	-0.00349
H	9	-0.00283
H	10	0.00117
C	11	0.34846
C	12	-0.11244
C	13	0.34399
C	14	-0.08076
C	15	0.01553
C	16	0.00087
O	17	-0.00397
O	18	0.00238
H	19	4E-05
H	20	0.00027
H	21	-0.0001
N	22	0.47505
N	23	0.10441
N	24	0.74068
H	25	-0.00994
H	26	0.00302
H	27	-0.01018
O	28	0.02991
C	29	-0.00172
H	30	0.00168
H	31	-4E-05
H	32	0.00168

10.3.2. Spin Density of ^{3}Br1a

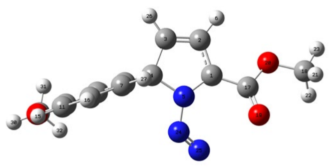

1	-0.43744	C
2	0.13569	C
3	-0.34891	C
4	0.02455	C
5	-0.20904	N
6	-0.00577	H
7	-0.02479	C
8	-0.00066	C
9	0.00000	C
10	-0.00118	C
11	-0.00014	C
12	-0.00082	C
13	0.00017	H
14	0.00000	H
15	-0.00023	H
16	-0.00032	H
17	-0.00570	C
18	0.00194	C
19	-0.11890	O
20	-0.02676	O
21	-0.00166	H
22	-0.00090	H
23	-0.00005	H
24	-0.10324	N
25	-0.85423	N
26	0.01025	H
27	-0.03143	H
28	-0.00032	O
29	0.00000	C
30	0.00000	H
31	0.00000	H
32	0.00000	H

10.3.2. Spin Density of ^{3}Br2a

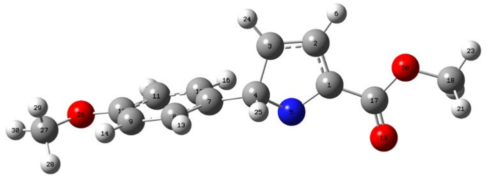

C	1	-0.30214
C	2	0.00885
C	3	-0.60549
C	4	0.05541
N	5	-0.93202
H	6	0.00000
C	7	-0.01787
C	8	-0.00533
C	9	-0.00333
C	10	-0.00802
C	11	0.00414
C	12	0.00731
H	13	0.00000
H	14	0.00000
H	15	-0.00028
H	16	0.00026
C	17	-0.00991
C	18	0.00181
O	19	-0.09573
O	20	-0.02180
H	21	-0.00109
H	22	-0.00108
H	23	-0.00005
H	24	0.01434
H	25	-0.0698
O	26	-0.00345
C	27	0.00017
H	28	-0.00017
H	29	-0.00016
H	30	0.00000

10.3.3. Spin Density ^{3}1Na-A

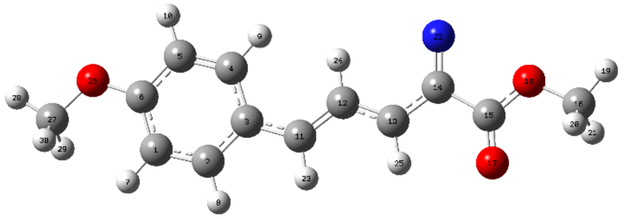

C	1	-0.04304
C	2	0.10848
C	3	-0.06844
C	4	0.08684
C	5	-0.04320
C	6	0.09334
H	7	0.00116
H	8	-0.00337
H	9	-0.00278
H	10	0.00120
C	11	0.35577
C	12	-0.15207
C	13	0.47253
C	14	-0.2057
C	15	0.06848
C	16	0.00054
O	17	0.01249
O	18	-5E-05
H	19	0.00015
H	20	7E-05
H	21	7E-05
N	22	1.30615
H	23	-0.00983
H	24	0.00425
H	25	-0.00948
O	26	0.02497
C	27	-0.00145
H	28	-4E-05
H	29	0.00147
H	30	0.00147

10.3.4. Spin Density ^{3}1Na-B

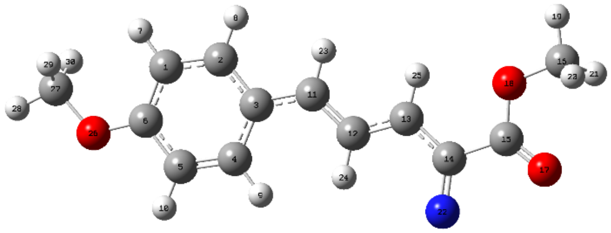

C	1	-0.04226
C	2	0.10700
C	3	-0.06736
C	4	0.08546
C	5	-0.04257
C	6	0.09207
H	7	0.00114
H	8	-0.00334
H	9	-0.00273
H	10	0.00118
C	11	0.35094
C	12	-0.15097
C	13	0.47218
C	14	-0.2102
C	15	0.07471
C	16	0.00479
O	17	-0.02622
O	18	0.00432
H	19	0.00024
H	20	-0.00010
H	21	-0.00010
N	22	1.34015
H	23	-0.00962
H	24	0.00377
H	25	-0.00876
O	26	0.02484
C	27	-0.00144
H	28	-5E-05
H	29	0.00146
H	30	0.00146

10.3.5. Spin Density of T of 1b-A

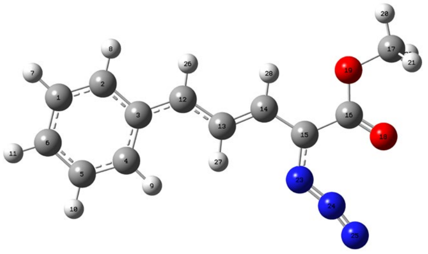

C	1	0.08498
C	2	-0.18593
C	3	0.08388
C	4	-0.19872
C	5	0.08996
C	6	-0.23259
H	7	-0.0024
H	8	0.00576
H	9	0.00597
H	10	-0.00255
H	11	0.00705
C	12	-0.52221
C	13	-0.09147
C	14	-0.17894
C	15	-0.43878
C	16	-0.02709
C	17	0.00224
O	18	-0.11971
O	19	-0.03102
H	20	0
H	21	-0.00154
H	22	0.00155
N	23	-0.03317
N	24	0.00535
N	25	-0.24088
H	26	0.01512
H	27	0.00264

10.3.5. Spin Density of T_A of 1b

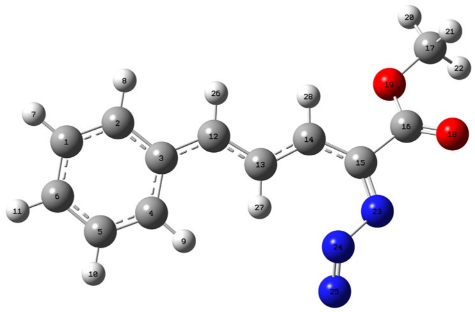

Atom	#	Spin Density
C	1	-0.05314
C	2	0.1109
C	3	0.07233
C	4	0.10985
C	5	-0.05212
C	6	0.1237
H	7	0.00151
H	8	-0.00341
H	9	-0.00338
H	10	0.00148
H	11	-0.00376
C	12	0.37728
C	13	-0.13045
C	14	0.36799
C	15	-0.09125
C	16	0.01623
C	17	0.00090
O	18	-0.00513
O	19	-0.00210
H	20	5E-05
H	21	0.00023
H	22	-0.00010
N	23	0.48135
N	24	0.10295
N	25	0.73657
H	26	-0.01075
H	27	0.00352
H	28	-0.01083

10.3.6. Spin Density of ^{3}Br2b-A

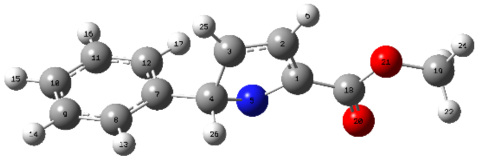

Atom	#	Spin Density
C	1	0.30361
C	2	-0.00793
C	3	0.61280
C	4	-0.05551
N	5	0.93641
H	6	-5E-05
C	7	0.01366
C	8	0.00408
C	9	-0.00039
C	10	0.00712
C	11	-0.00250
C	12	0.00796
H	13	6E-05
H	14	7E-05
H	15	-0.00013
H	16	0.00023
H	17	-0.00035
C	18	0.00825
C	19	-0.00180
O	20	0.09480
O	21	0.02156
H	22	0.00108
H	23	0.00107
H	24	1E-05
H	25	-0.01445
H	26	0.07031

10.3.7. Spin Density of ^{3}Br1b-A

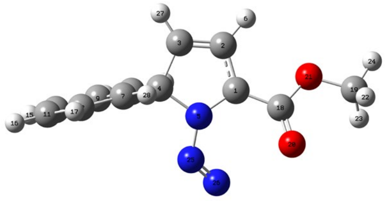

Atom	#	Spin Density
C	1	0.43862
C	2	-0.13640
C	3	0.35055
C	4	-0.02454
N	5	0.20857
H	6	0.00583
C	7	0.02460
C	8	0.00072
C	9	-0.00018
C	10	0.00156
C	11	0
C	12	0.00125
H	13	-0.00019
H	14	0
H	15	0
H	16	0.00025
H	17	0.00032
C	18	0.00482
C	19	-0.00195
O	20	0.11858
O	21	0.02675
H	22	0.00166
H	23	0.00089
H	24	0
N	25	0.10167
N	26	0.85548
H	27	-0.01030
H	28	

10.3.7. Spin Density of ³Br1b-B

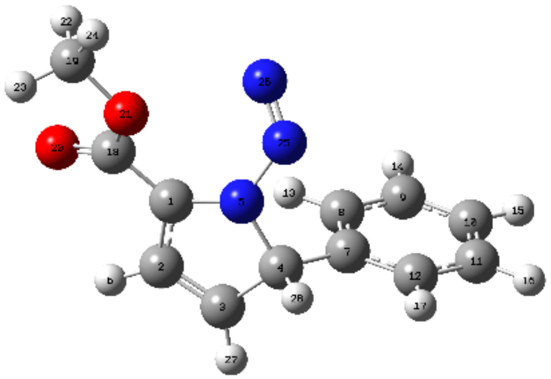

Atom	#	Spin Density
C	1	0.45100
C	2	-0.13895
C	3	0.35851
C	4	-0.02454
N	5	0.19350
H	6	0.00586
C	7	0.02333
C	8	0.00091
C	9	-0.00027
C	10	0.00179
C	11	-0.00011
C	12	0.00147
H	13	-0.00019
H	14	1E-04
H	15	-6E-05
H	16	0.00024
H	17	0.00029
C	18	-1E-05
C	19	4E-05
O	20	0.11873
O	21	0.02491
H	22	0.00105
H	23	0.00076
H	24	2E-05
N	25	0.11242
N	26	0.84915
H	27	-0.01053
H	28	0.03059

10.3.7. Spin Density of ^{3}1Nb-A

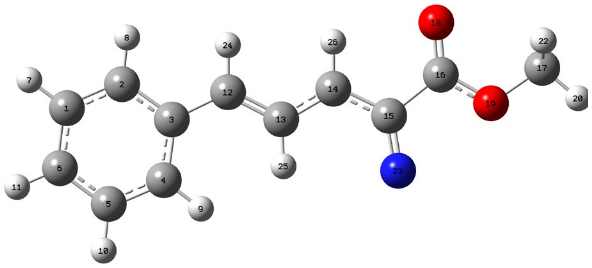

Atom	#	Spin Density
C	1	0.05084
C	2	-0.10572
C	3	-0.0751
C	4	-0.10426
C	5	0.04951
C	6	-0.11478
H	7	-0.00147
H	8	0.00326
H	9	0.00322
H	10	-0.37516
H	11	0.16318
C	12	-0.37516
C	13	0.16318
C	14	-0.48823
C	15	0.21111
C	16	-0.06902
C	17	-0.00057
O	18	-0.01189
O	19	0
H	20	-0.00016
H	21	0
-H	22	0
N	23	-1.30242
H	24	0.01036
H	25	-0.00457
H	26	0.00986

10.4. Calculated Stationary Points on the Energy Diagram for 1

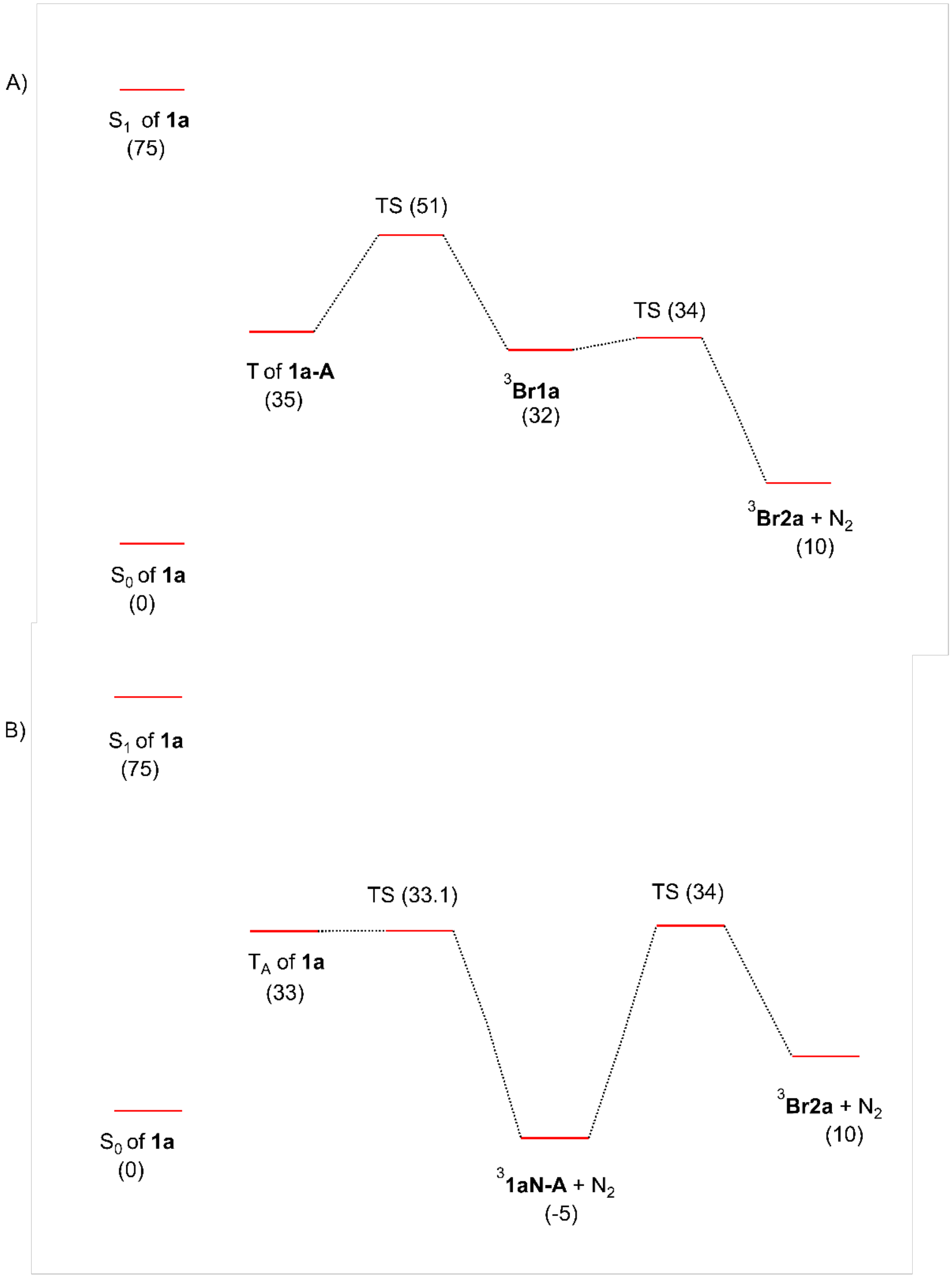

Figure S**59**. Calculated (B3LYP-D3/6-31+G(d,p)) stationary points on the triplet surface of 1a for A) forming 2a from T of 1a and B) forming 2b from 31bN. Energies are in kcal/mol.

11. Emission Spectra of Light Sources

11.1. Emission spectrum of Microscope White Light

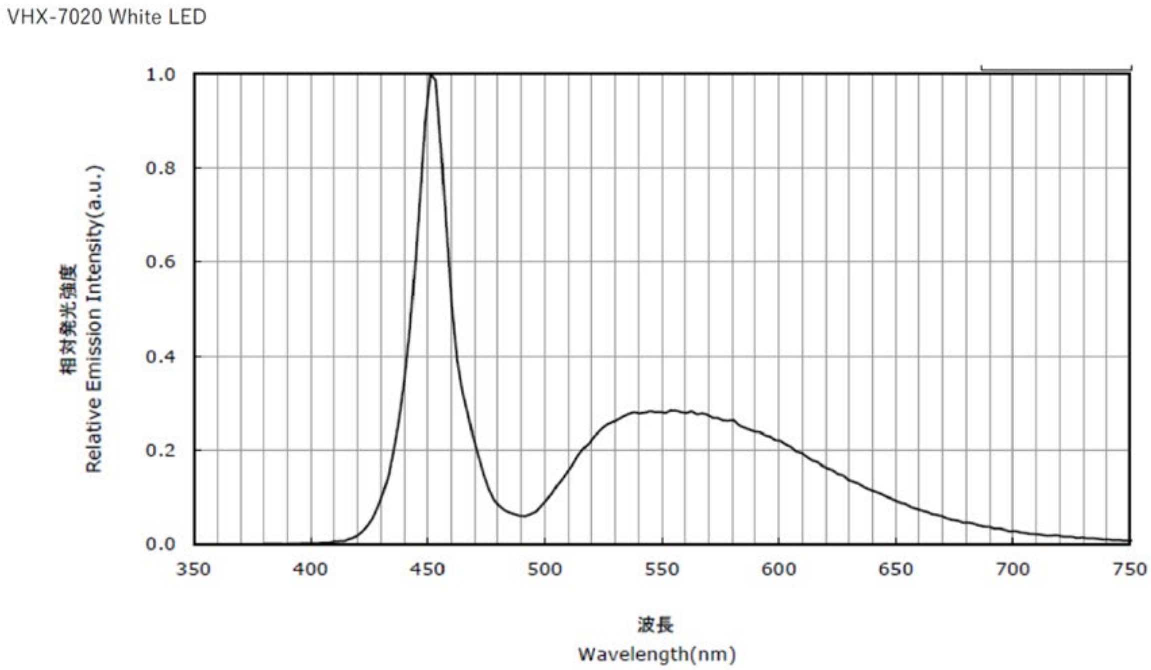

Figure S60. Keyence microscope white light emission for irradiation at 100% intensity with λmax at 450 and 550 nm.

11.2. Emission spectra of LEDs

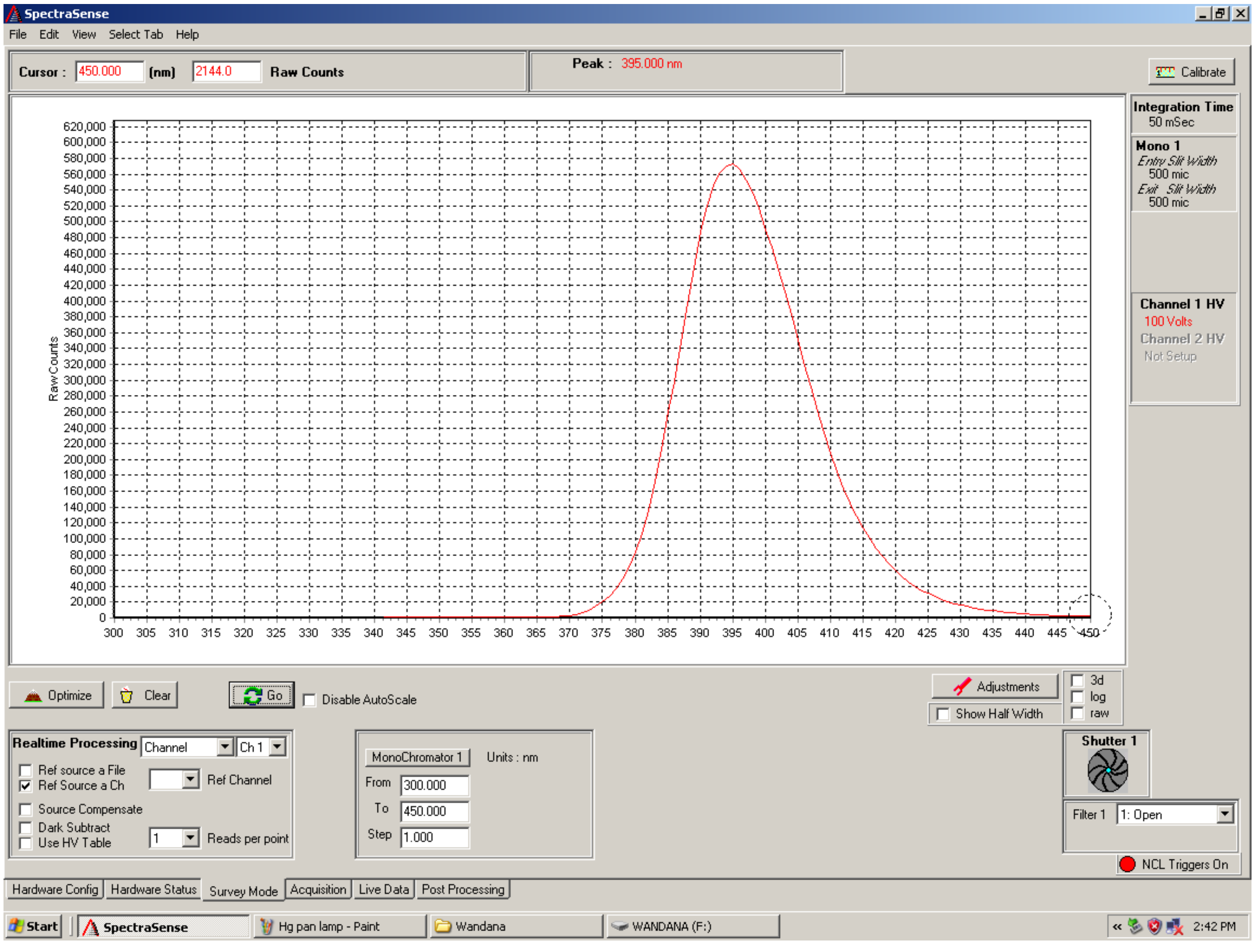

Figure S61. Emission spectrum of LEXEON 395 nm LED

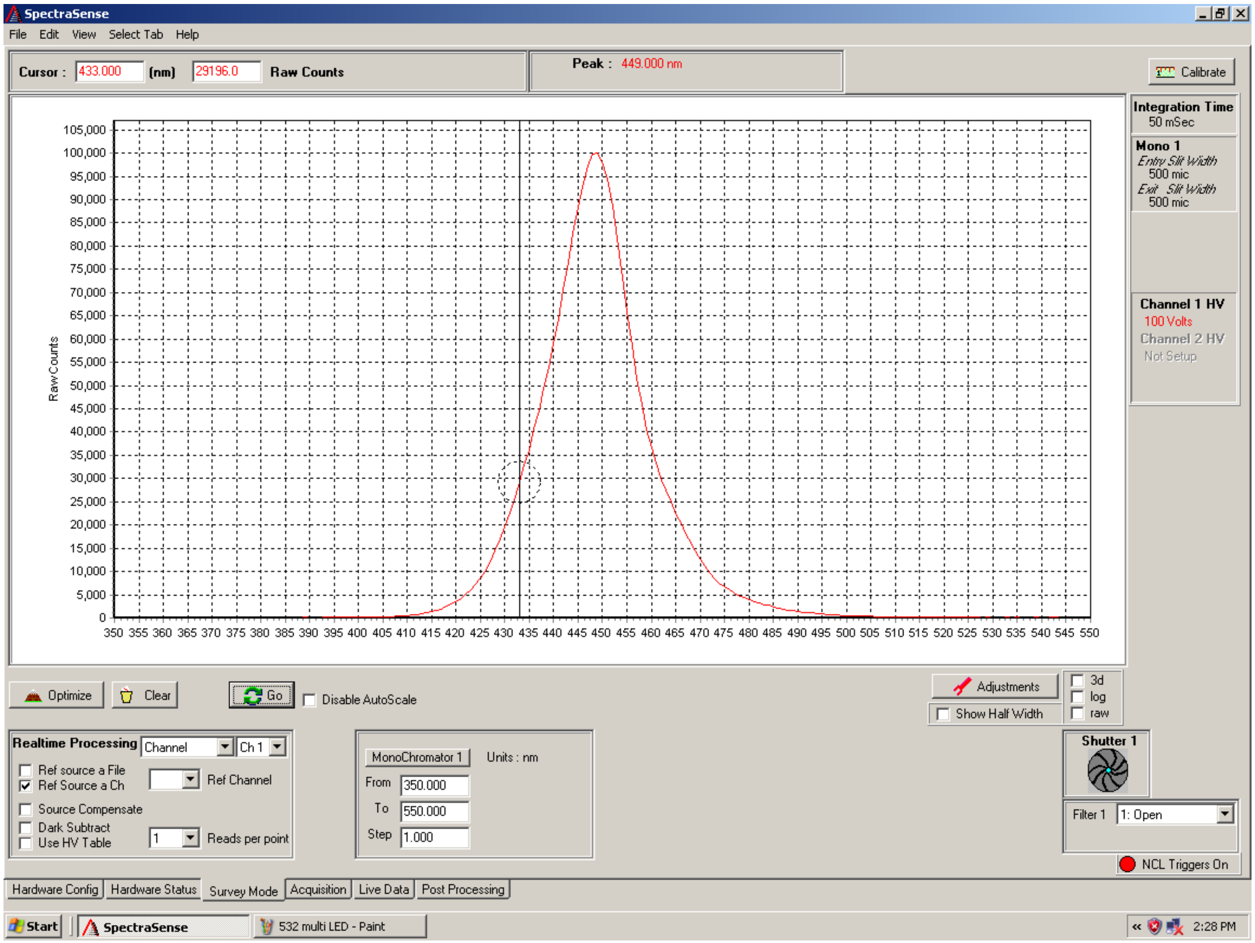

Figure S62. Emission spectrum of LEXEON Z 450 nm LED

Figure S28. Emission spectrum of LEXEON Z 450 nm LED

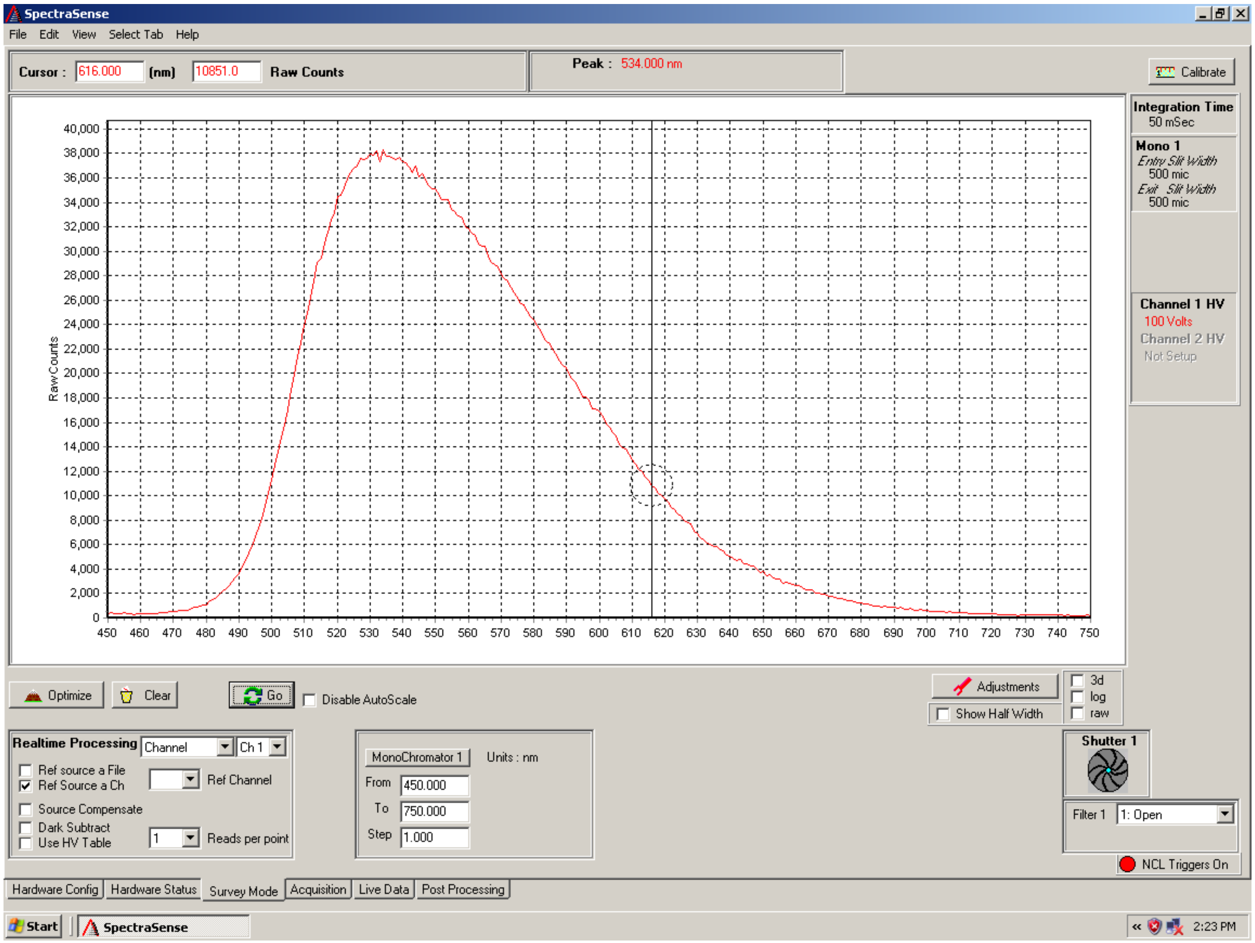

Figure S63. Emission spectrum of LEXEON Z 532 nm LED

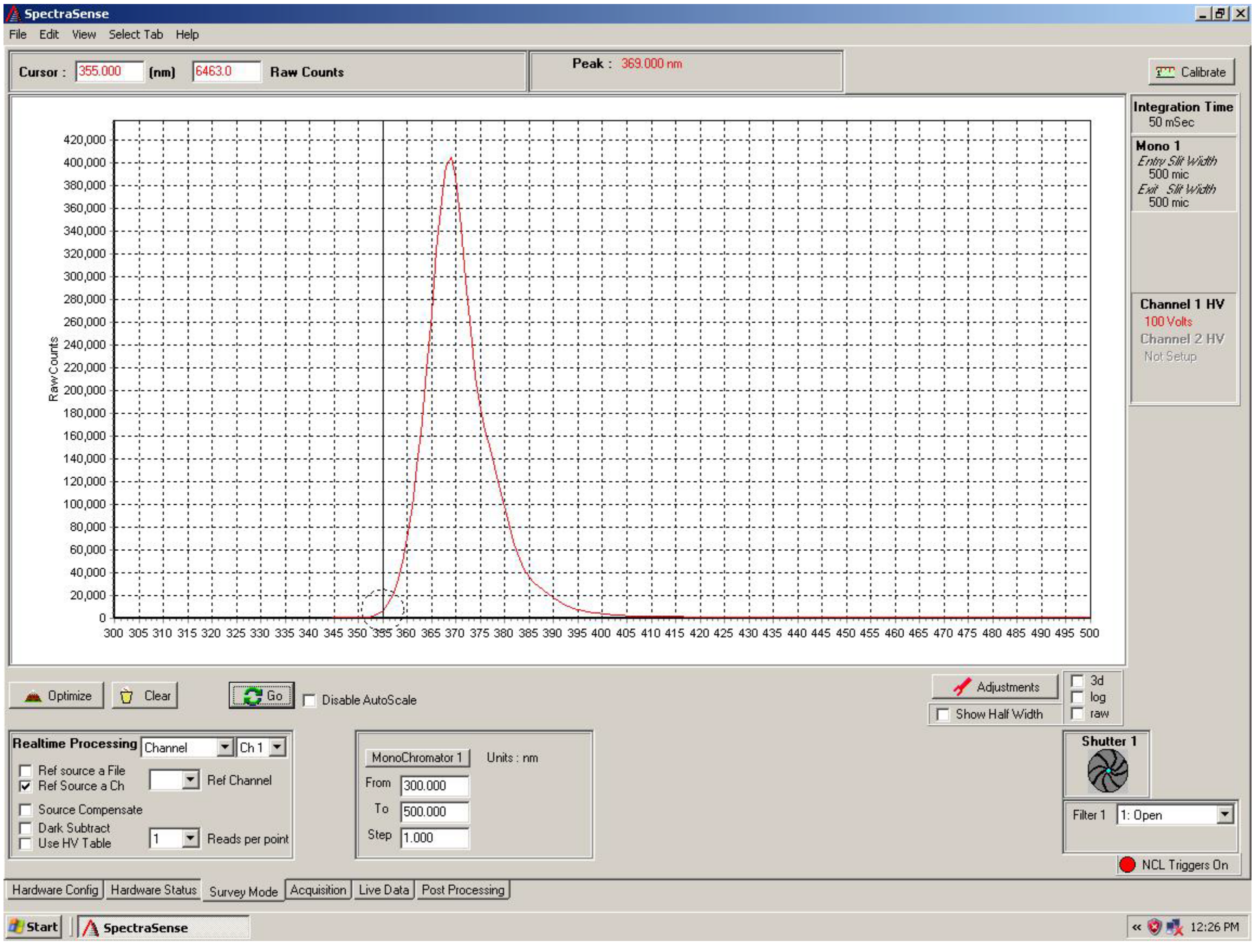

Figure S64. Emission spectrum of 365 nm LED

11.3. 254 nm UV Pen Emission Spectrum

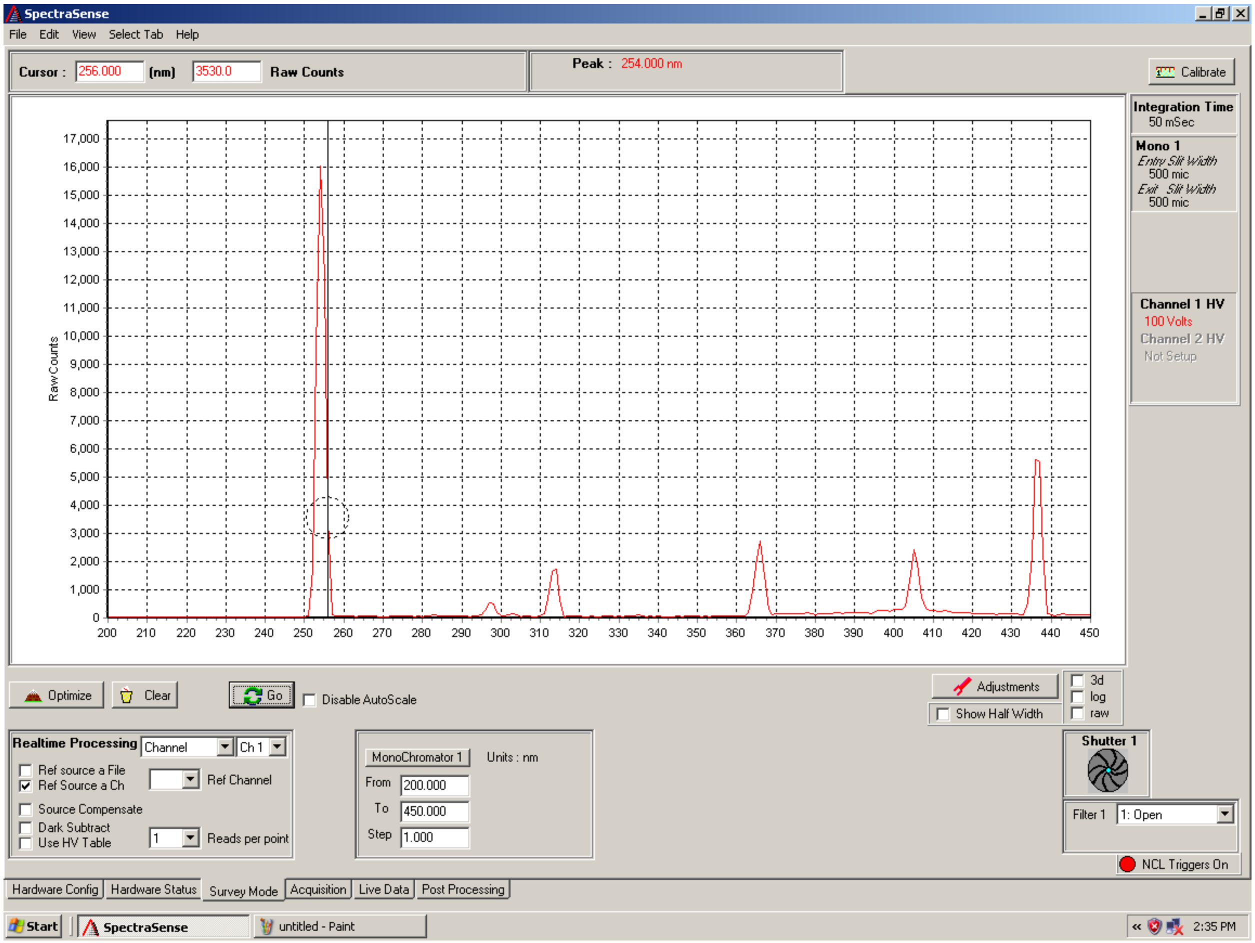

Figure S65. Emission spectrum of 254 nm Analytik Jena (UVP) Pen-Ray 90001201

12. Dynamic Light Scattering Data (DLS)

12.1. DLS analysis of azide 1a nanocrystalline suspension

Table S60. Summary of DLS analyses of azide **1a** nanocrystalline suspension in water

Summary Data	
MV(nm):	536.0
MN(nm):	454.0
MA(nm):	509.0
CS:	11.78
SD:	117.6
PDI:	0.1463
Mz:	534.2
si:	117.8
Ski:	75.83
Kg:	994.7

Table S61. Percentiles of DLS analyses of azide **1a** nanocrystalline suspension in water

Percentiles	
%Tile	Size(nm)
10.00	389.0
20.00	435.0
30.00	469.0
40.00	501.0
50.00	530.0
60.00	560.0
70.00	594.0
80.00	634.0
90.00	692.0
95.00	742.0

Table S62. Raw data of DLS analyses of azide **1a** nanocrystalline suspension in water

Tabular Data		
Size(nm)	%Chan	% Pass
6540	0.00	100.00
5500	0.00	100.00
4620	0.00	100.00
3890	0.00	100.00
3270	0.00	100.00
2750	0.00	100.00
2312	0.00	100.00
1944	0.00	100.00
1635	0.00	100.00
1375	0.00	100.00
1156	0.00	100.00
972.0	1.19	100.00
818.0	9.33	98.81
687.0	23.88	89.48
578.0	30.46	65.60
486.0	21.46	35.14
409.0	9.59	13.68
344.0	3.09	4.09
289.0	0.84	1.00
243.0	0.16	0.16
204.4	0.00	0.00
171.9	0.00	0.00
144.5	0.00	0.00
121.5	0.00	0.00
102.2	0.00	0.00
85.90	0.00	0.00
72.30	0.00	0.00
60.80	0.00	0.00
51.10	0.00	0.00
43.00	0.00	0.00
36.10	0.00	0.00
30.40	0.00	0.00
25.55	0.00	0.00
21.48	0.00	0.00
18.06	0.00	0.00
15.19	0.00	0.00
12.77	0.00	0.00
10.74	0.00	0.00
9.03	0.00	0.00
7.60	0.00	0.00
6.39	0.00	0.00
5.37	0.00	0.00
4.52	0.00	0.00
3.80	0.00	0.00
3.19	0.00	0.00
2.690	0.00	0.00
2.260	0.00	0.00
1.900	0.00	0.00
1.600	0.00	0.00
1.340	0.00	0.00
1.130	0.00	0.00
0.950	0.00	0.00

12.2. DLS analysis of azide 1b nanocrystalline suspension

Table S63. Summary of DLS analyses of azide **1b** nanocrystalline suspension in water

Summary Data	
MV(um):	0.841
MN(um):	0.1100
MA(um):	0.2119
CS:	28.31
SD:	1.320
PDI:	0.5960
Mz:	1.045
si:	1.250
Ski:	0.890
Kg:	7.60

Table S64. Percentiles of DLS analyses of azide **1b** nanocrystalline suspension in water

Percentiles	
%Tile	Size(µm)
10.00	0.0962
20.00	0.1215
30.00	0.1777
40.00	0.2377
50.00	0.2737
60.00	0.303
70.00	0.332
80.00	0.392
90.00	3.30
95.00	3.98

Table S65. Raw data of DLS analyses of azide **1b** nanocrystalline suspension in water

Tabular Data		
Size(um)	%Chan	% Pass
6.54	0.14	100.00
5.50	2.08	99.86
4.62	3.29	97.78
3.89	4.82	94.49
3.27	5.68	89.67
2.750	2.53	83.99
2.312	0.00	81.46
1.944	0.00	81.46
1.635	0.00	81.46
1.375	0.00	81.46
1.156	0.00	81.46
0.972	0.00	81.46
0.818	0.00	81.46
0.687	0.00	81.46
0.578	0.00	81.46
0.486	0.45	81.46
0.409	7.92	81.01
0.344	18.02	73.09
0.2890	13.86	55.07
0.2430	7.47	41.21
0.2044	4.50	33.74
0.1719	4.04	29.24
0.1445	5.21	25.20
0.1215	7.23	19.99
0.1022	7.56	12.76
0.0859	4.66	5.20
0.0723	0.54	0.54
0.0608	0.00	0.00
0.0511	0.00	0.00
0.0430	0.00	0.00
0.0361	0.00	0.00
0.0304	0.00	0.00
0.02555	0.00	0.00
0.02148	0.00	0.00
0.01806	0.00	0.00
0.01519	0.00	0.00
0.01277	0.00	0.00
0.01074	0.00	0.00
0.00903	0.00	0.00
0.00760	0.00	0.00
0.00639	0.00	0.00
0.00537	0.00	0.00
0.00452	0.00	0.00
0.00380	0.00	0.00
0.00319	0.00	0.00
0.00269	0.00	0.00
0.00226	0.00	0.00
0.00190	0.00	0.00
0.00160	0.00	0.00
0.00134	0.00	0.00
0.00113	0.00	0.00
0.00095	0.00	0.00

12.3. DLS analysis of azide 1b nanocrystalline suspension for PXRD analysis

Table S66. Summary of DLS analyses of azide **1b** nanocrystalline suspension in water

Summary Data	
MI(nm):	285.0
MN(nm):	249.8
MA(nm):	274.1
CS:	21.89
SD:	52.90
PDI:	0.0995
Mz:	285.2
si:	53.74
Ski:	2.576
Kg:	1029

Table S67. Raw data of DLS analyses of azide **1b** nanocrystalline suspension in water

Tabular Data		
Size(nm)	%Chan	% Pass
6540	0.00	100.00
5500	0.00	100.00
4620	0.00	100.00
3890	0.00	100.00
3270	0.00	100.00
2750	0.00	100.00
2312	0.00	100.00
1944	0.00	100.00
1635	0.00	100.00
1375	0.00	100.00
1156	0.00	100.00
972.0	0.00	100.00
818.0	0.00	100.00
687.0	0.00	100.00
578.0	0.00	100.00
486.0	1.08	100.00
409.0	12.21	98.92
344.0	33.70	86.71
289.0	31.84	53.01
243.0	14.23	21.17
204.4	4.88	6.94
171.9	1.84	2.06
144.5	0.22	0.22
121.5	0.00	0.00
102.2	0.00	0.00
85.90	0.00	0.00
72.30	0.00	0.00
60.80	0.00	0.00
51.10	0.00	0.00
43.00	0.00	0.00
36.10	0.00	0.00
30.40	0.00	0.00
25.55	0.00	0.00
21.48	0.00	0.00
18.06	0.00	0.00
15.19	0.00	0.00
12.77	0.00	0.00
10.74	0.00	0.00
9.03	0.00	0.00
7.60	0.00	0.00
6.39	0.00	0.00
5.37	0.00	0.00
4.52	0.00	0.00
3.80	0.00	0.00
3.19	0.00	0.00
2.690	0.00	0.00
2.260	0.00	0.00
1.900	0.00	0.00
1.600	0.00	0.00
1.340	0.00	0.00
1.130	0.00	0.00
0.950	0.00	0.00

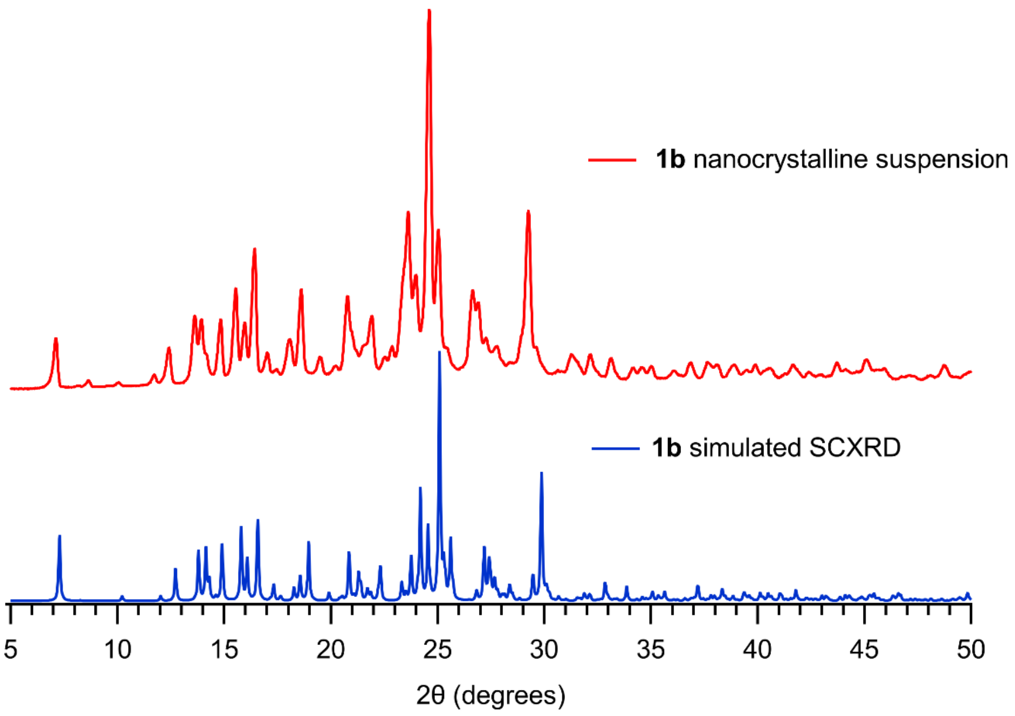

Figure S66. PXRD pattern of nanocrystalline **1b** obtained from the water suspension

Table S68. Volume for unit cell of 1a, 1b, 2a and 2b from their crystal structures.

1a 2588.9 Å^3
2a 2301.9 Å^3
Difference = 287 Å^3

1b 1176.01 x 2 = 2352.02 Å^3
2b 2012.44 Å^3
Difference 339.58 Å^3

13. Video Descriptions

Video 1- Azide **1a** (crystal A) in air irradiated and monitored using an optical microscope with a video camera for 10:26 minutes shortened to a playback speed of 1:02 minutes.

Video 2- Azide **1a** (crystal B) in air irradiated and monitored using an optical microscope with a video camera, for 7:45 minutes shortened to a playback speed of 58 seconds

Video 3- Azide **1a** (crystal C) in mineral oil irradiated and monitored using an optical microscope with a video camera 7:05 minutes shortened to a playback speed of 1 minute

Video 4- Azide **1b** (crystal D) in air irradiated and monitored using an optical microscope with a video camera, shortened from 11 minutes to 30 seconds

Video 5- Azide **1b** (crystal E) in mineral oil monitored using an optical microscope with a video camera for 5 minutes to playback speed of 1:15 minutes

Video 6- Azide **1b** (crystal F) in minimal MeOH irradiated and monitored using an optical microscope with a video camera, shortened from 12 minutes to 31 seconds

11. Experimental Sentence for our PXRD instrument:

Powdered samples were analyzed at room temperature using Cu Ka radiation (3-100 deg in theta-2theta, 2 deg/min, 0.01 deg step width, reflection mode) on a Rigaku Mini-Flex 6G diffractometer.

14. SEM and NMR analyses after Sequential Photoirradiation and PXRD Measurements

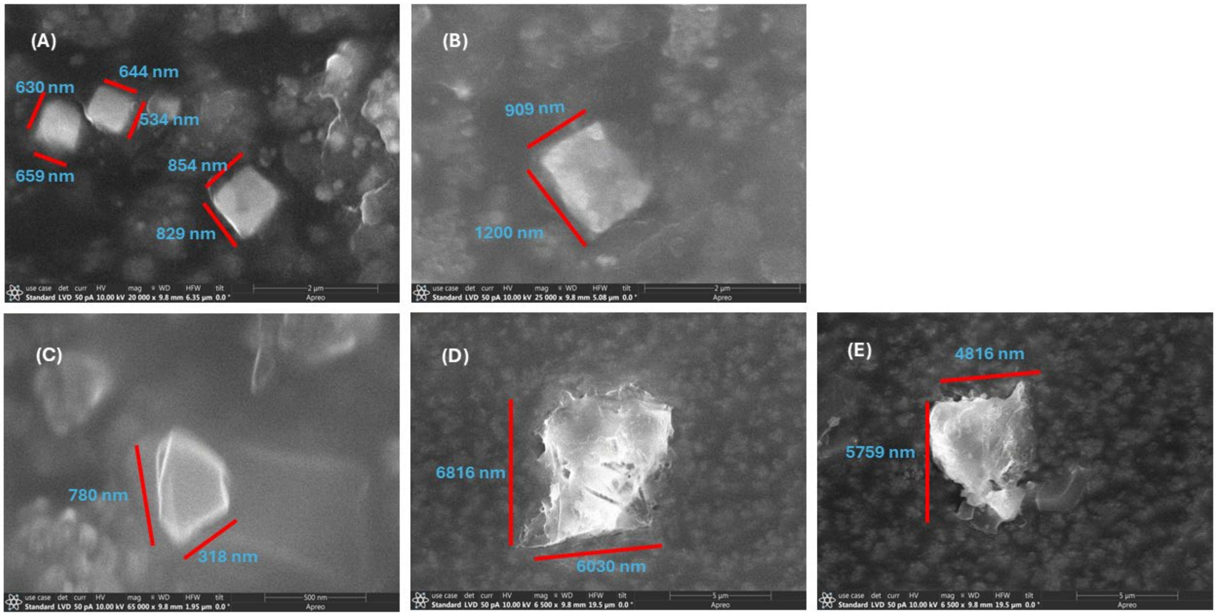

Figure S67. SEM images of the particles after sequential irradiations and PXRD analysis: (A, B) **2a**; (C–E) **2b**.

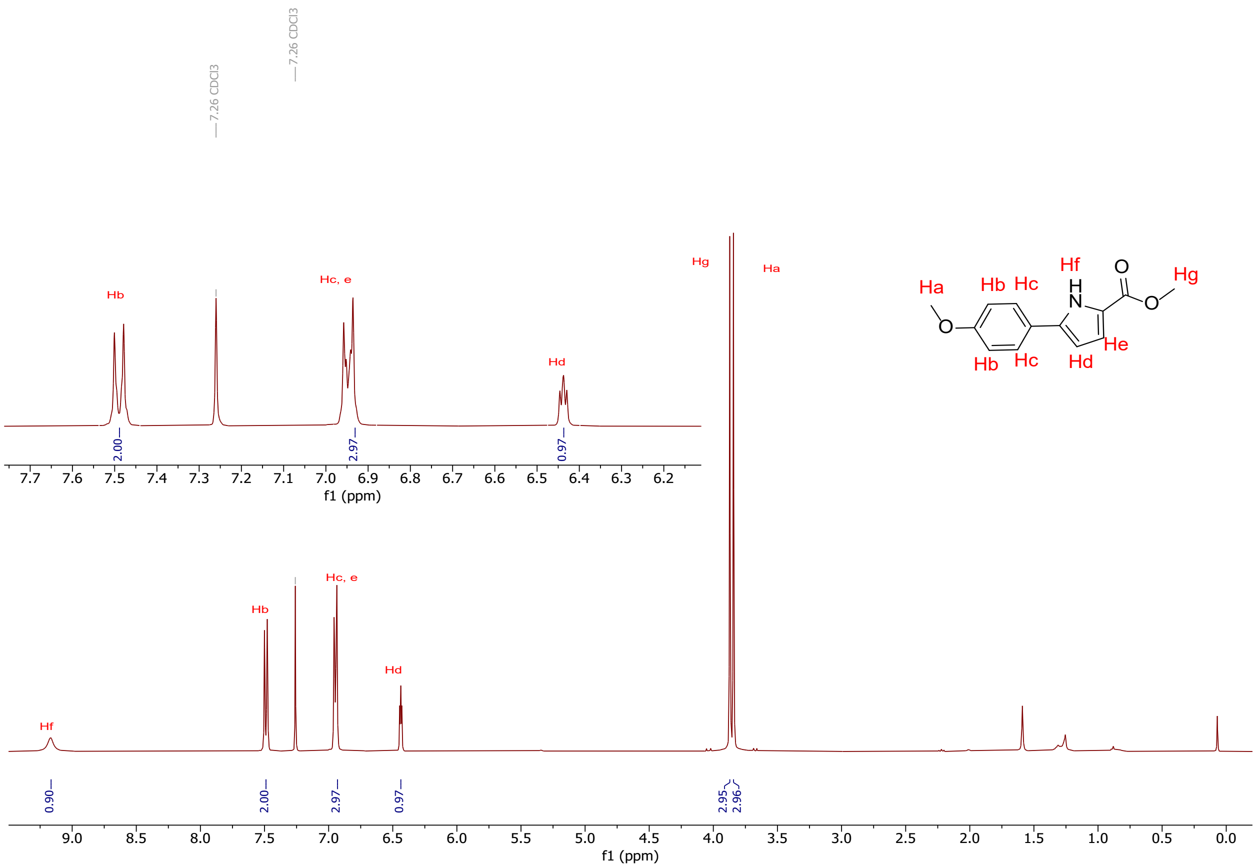

Figure S68. ^{1}H-NMR (400 MHz, $CDCl_3$) spectra after irradiating powdered **1a** crystals (365 nm LED, 71.5 h) and subsequent PXRD measurements

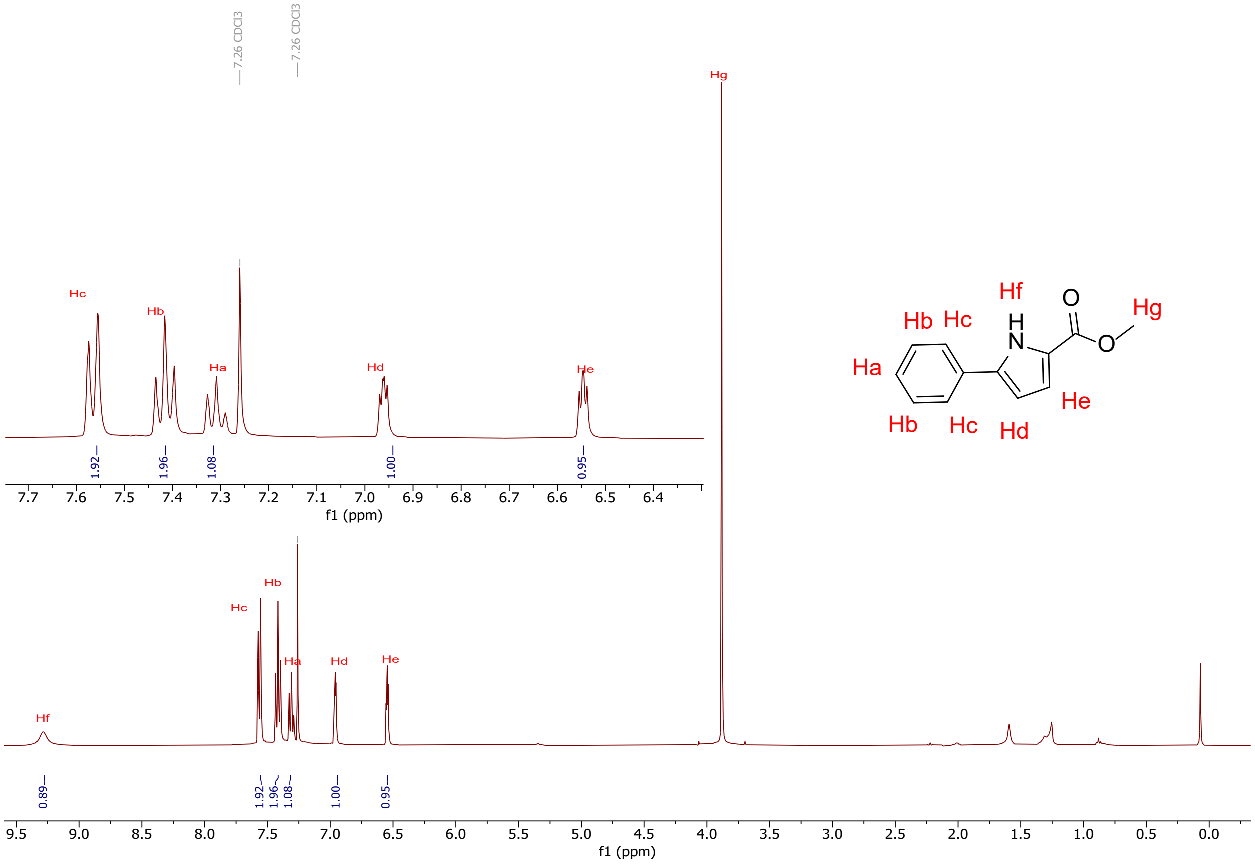

Figure S69. ^{1}H-NMR (400 MHz, $CDCl_3$) spectra after irradiating powdered **1b** crystals (365 nm LED, 41.5 h) and subsequent PXRD measurements

15. CCDC Structure Deposition

checkCIF (basic structural check) running

Checking for embedded fcf data in CIF ...
Found embedded fcf data in CIF. Extracting fcf data from uploaded CIF, please wait . . . . **checkCIF/PLATON (basic structural check)**

Structure factors have been supplied for datablock(s) 18075, 18075c, 20093_150, 20093_200, 20093_250, 20093_295, 22094, 22152
THIS REPORT IS FOR GUIDANCE ONLY. IF USED AS PART OF A REVIEW PROCEDURE FOR PUBLICATION, IT SHOULD NOT REPLACE THE EXPERTISE OF AN EXPERIENCED CRYSTALLOGRAPHIC REFEREE.
No syntax errors found. CIF dictionary
Please wait while processing .... Interpreting this report
Structure factor report

Datablock: 20093_150

Bond precision:		C-C = 0.0020 A	Wavelength=0.71073
Cell:	a=13.0459(14)	b=8.3080(9)	c=23.886(3)
	alpha=90	beta=90	gamma=90
Temperature:	150 K		
		Calculated	Reported
Volume		2588.9(5)	2588.9(5)
Space group		P b c a	P b c a
Hall group		-P 2ac 2ab	-P 2ac 2ab
Moiety formula		C13 H13 N3 O3	C13 H13 N3 O3
Sum formula		C13 H13 N3 O3	C13 H13 N3 O3
Mr		259.26	259.26
Dx,g cm-3		1.330	1.330
Z		8	8
Mu (mm-1)		0.097	0.097
F000		1088.0	1088.0
F000'		1088.51	
h,k,lmax		16,10,31	16,10,31
Nref		2980	2977
Tmin,Tmax		0.984,0.993	0.802,0.862
Tmin'		0.983	

Correction method= # Reported T Limits: Tmin=0.802 Tmax=0.862 AbsCorr = NUMERICAL

Data completeness= 0.999 Theta(max)= 27.531

R(reflections)= 0.0401(1975) wR2(reflections)= 0.0936(2977)

S = 1.033 Npar= 174

NOTE FROM AUTHORS: Displacement of these atoms is perpendicular to the plane of the entire molecule. The shift in electron density due to the resonance ability of the azide group (N=N-N vs N-N=N) may also be a contributing factor, resulting in this alert. This is a common observation in other azides.

Alert level B

PLAT230_ALERT_2_B Hirshfeld Test Diff for N1 --N2 . 8.6 s.u.

Alert level G

PLAT912_ALERT_4_G Missing # of FCF Reflections Above STh/L= 0.600 3 Note
PLAT955_ALERT_1_G Reported (CIF) and Actual (FCF) Lmax Differ by . 1 Units
PLAT969_ALERT_5_G The 'Henn et al.' R-Factor-gap value .... 4.73 Note
Predicted wR2: Based on SigI**2 1.98 or SHELX Weight 9.34
PLAT978_ALERT_2_G Number C-C Bonds with Positive Residual Density. 12 Info

Datablock: 20093_200

Bond precision:		C-C = 0.0020 A	Wavelength=0.71073
Cell:	a=13.0646(8)	b=8.3471(5)	c=23.9088(15)
	alpha=90	beta=90	gamma=90
Temperature:	200 K		

	Calculated	Reported
Volume	2607.3(3)	2607.3(3)
Space group	P b c a	P b c a
Hall group	-P 2ac 2ab	-P 2ac 2ab
Moiety formula	C13 H13 N3 O3	C13 H13 N3 O3
Sum formula	C13 H13 N3 O3	C13 H13 N3 O3
Mr	259.26	259.26
Dx,g cm-3	1.321	1.321
Z	8	8
Mu (mm-1)	0.096	0.096
F000	1088.0	1088.0
F000'	1088.51	
h,k,lmax	16,10,29	16,10,29
Nref	2682	2682
Tmin,Tmax	0.985,0.993	0.819,0.862
Tmin'	0.983	

Correction method= # Reported T Limits: Tmin=0.819 Tmax=0.862 AbsCorr = MULTI-SCAN

Data completeness= 1.000 Theta(max)= 26.400

R(reflections)= 0.0393(1733) wR2(reflections)= 0.1021(2682)

S = 1.020 Npar= 174

NOTE FROM AUTHORS: Displacement of these atoms is perpendicular to the plane of the entire molecule. The shift in electron density due to the resonance ability of the azide group (N=N-N vs N-N=N) may also be a contributing factor, resulting in this alert. This is a common observation in other azides.

Alert level C

PLAT230_ALERT_2_C Hirshfeld Test Diff for N1 --N2 . 6.1 s.u.

Alert level G

PLAT969_ALERT_5_G The 'Henn et al.' R-Factor-gap value .... 4.99 Note

Predicted wR2: Based on SigI**2 2.04 or SHELX Weight 10.35

PLAT978_ALERT_2_G Number C-C Bonds with Positive Residual Density. 4 Info

Datablock: 20093_250

Bond precision:		C-C = 0.0030 A	Wavelength=0.71073
Cell:	a=13.1194(10)	b=8.3878(7)	c=23.9199(19)
	alpha=90	beta=90	gamma=90
Temperature:	250 K		

	Calculated	Reported
Volume	2632.2(4)	2632.2(4)
Space group	P b c a	P b c a
Hall group	-P 2ac 2ab	-P 2ac 2ab
Moiety formula	C13 H13 N3 O3	C13 H13 N3 O3
Sum formula	C13 H13 N3 O3	C13 H13 N3 O3
Mr	259.26	259.26

Dx,g cm-3	1.309		1.308
Z	8		8
Mu (mm-1)	0.095		0.095
F000	1088.0	1088.0	
F000'	1088.51		
h,k,lmax	16,10,29	16,10,29	
Nref	2716		2714
Tmin,Tmax	0.985,0.993	0.811,0.862	
Tmin'	0.983		

Correction method= # Reported T Limits: Tmin=0.811 Tmax=0.862 AbsCorr = MULTI-SCAN

Data completeness= 0.999 Theta(max)= 26.418

R(reflections)= 0.0429(1528) wR2(reflections)= 0.1096(2714)

S = 1.003 Npar= 174

NOTE FROM AUTHORS: Displacement of these atoms is perpendicular to the plane of the entire molecule. The shift in electron density due to the resonance ability of the azide group (N=N-N vs N-N=N) may also be a contributing factor, resulting in this alert. This is a common observation in other azides.

Alert level B

PLAT230_ALERT_2_B Hirshfeld Test Diff for N1 --N2 . 10.9 s.u.

Alert level C

PLAT905_ALERT_3_C Negative K value in the Analysis of Variance ... -0.202 Report

---

Alert level G

PLAT912_ALERT_4_G Missing # of FCF Reflections Above STh/L= 0.600 2 Note
PLAT969_ALERT_5_G The 'Henn et al.' R-Factor-gap value .... 4.96 Note
Predicted wR2: Based on SigI**2 2.21 or SHELX Weight 11.29 PLAT978_ALERT_2_G Number C-C Bonds with Positive Residual Density. 0 Info

---

Datablock: 20093_295

Bond precision:		C-C = 0.0040 A	Wavelength=0.71073
Cell:	a=13.1408(16)	b=8.4392(10)	c=23.972(3)
	alpha=90	beta=90	gamma=90
Temperature:	295 K		

	Calculated	Reported
Volume	2658.4(6)	2658.4(5)
Space group	P b c a	P b c a
Hall group	-P 2ac 2ab	-P 2ac 2ab
Moiety formula	C13 H13 N3 O3	C13 H13 N3 O3
Sum formula	C13 H13 N3 O3	C13 H13 N3 O3
Mr	259.26	259.26
Dx,g cm-3	1.296	1.296
Z	8	8
Mu (mm-1)	0.094	0.094
F000	1088.0	1088.0
F000'	1088.51	
h,k,lmax	16,10,29	16,10,29
Nref	2734	2734
Tmin,Tmax	0.985,0.993	0.772,0.862
Tmin'	0.983	

Correction method= # Reported T Limits: Tmin=0.772 Tmax=0.862 AbsCorr = MULTI-SCAN

Data completeness= 1.000 Theta(max)= 26.391

R(reflections)= 0.0560(1181) wR2(reflections)= 0.1254(2734)

S = 1.005 Npar= 174

NOTE FROM AUTHORS: Displacement of these atoms is perpendicular to the plane of the entire molecule. The shift in electron density due to the resonance ability of the azide group (N=N-N vs N-N=N) may also be a contributing factor, resulting in this alert. This is a common observation in other azides.

Alert level B

PLAT230_ALERT_2_B Hirshfeld Test Diff for N1 --N2 . 8.7 s.u.

Alert level C

RINTA01_ALERT_3_C The value of Rint is greater than 0.12 Rint given 0.139

PLAT026_ALERT_3_C Ratio Observed / Unique Reflections (too) Low .. 43% Check

PLAT906_ALERT_3_C Large K Value in the Analysis of Variance ...... 2.419 Check

Alert level G

PLAT020_ALERT_3_G The Value of Rint is Greater Than 0.12 .... 0.139 Report

PLAT969_ALERT_5_G The 'Henn et al.' R-Factor-gap value .... 3.55 Note

Predicted wR2: Based on SigI**2 3.53 or SHELX Weight 12.90

PLAT978_ALERT_2_G Number C-C Bonds with Positive Residual Density. 0 Info

Datablock: 18075c

Bond precision:		C-C = 0.0025 A	Wavelength=0.71073
Cell:	a=7.9098(3)	b=13.1978(5)	c=13.0071(6)
	alpha=90	beta=119.9924(14)	gamma=90
Temperature:	150 K		

	Calculated	Reported
Volume	1176.01(8)	1176.01(8)
Space group	P 21/c	P 21/c
Hall group	-P 2ybc	-P 2ybc
Moiety formula	C12 H11 N3 O2	?
Sum formula	C12 H11 N3 O2	C12 H11 N3 O2
Mr	229.24	229.24
Dx,g cm-3	1.295	1.295
Z	4	4
Mu (mm-1)	0.091	0.091
F000	480.0	480.0
F000'	480.21	
h,k,lmax	9,16,16	9,16,16
Nref	2411	2411
Tmin,Tmax	0.989,0.993	0.719,0.862
Tmin'	0.989	

Correction method= # Reported T Limits: Tmin=0.719 Tmax=0.862 AbsCorr = NUMERICAL

Data completeness= 1.000 Theta(max)= 26.362

R(reflections)= 0.0434(1796) wR2(reflections)= 0.1132(2411)

S = 1.045 Npar= 187

NOTE FROM AUTHORS: Displacement of these atoms is perpendicular to the plane of the entire molecule. The shift in electron density due to the resonance ability of the azide group (N=N-N vs N-N=N) may also be a contributing factor, resulting in this alert. This is a common observation in other azides.

Alert level B

PLAT230_ALERT_2_B Hirshfeld Test Diff for N1 --N2 . 7.2 s.u.

Alert level C

PLAT230_ALERT_2_C Hirshfeld Test Diff for C3 --C4 . 6.0 s.u.

PLAT906_ALERT_3_C Large K Value in the Analysis of Variance ...... 3.789 Check

---

Alert level G

PLAT128_ALERT_4_G Alternate Setting for Input Space Group P21/c P21/n Note

PLAT910_ALERT_3_G Missing # of FCF Reflection(s) Below Theta(Min). 1 Note

0 1 1,

PLAT941_ALERT_3_G Average HKL Measurement Multiplicity .... 4.8 Low

PLAT969_ALERT_5_G The 'Henn et al.' R-Factor-gap value .... 3.31 Note

Predicted wR2: Based on SigI**2 3.42 or SHELX Weight 11.28 PLAT978_ALERT_2_G Number C-C Bonds with Positive Residual Density. 5 Info

---

Datablock: 22094

Bond precision:	C-C = 0.0019 A		Wavelength=0.71073
Cell:	a=7.2493(5)	b=14.6977(9)	c=21.3712(13)
	alpha=90	beta=91.2626(11)	gamma=90
Temperature:	150 K		

	Calculated	Reported
Volume	2276.5(3)	2276.5(3)
Space group	P 21/c	P 21/c
Hall group	-P 2ybc	-P 2ybc
Moiety formula	C12 H11 N3 O2	C12 H11 N3 O2
Sum formula	C12 H11 N3 O2	C12 H11 N3 O2
Mr	229.24	229.24
Dx,g cm-3	1.338	1.338
Z	8	8
Mu (mm-1)	0.094	0.094
F000	960.0	960.0
F000'	960.41	
h,k,lmax	9,19,28	9,19,28
Nref	5667	5665
Tmin,Tmax	0.990,0.994	0.820,0.862
Tmin'	0.969	

Correction method= # Reported T Limits: Tmin=0.820 Tmax=0.862 AbsCorr = MULTI-SCAN

Data completeness= 1.000 Theta(max)= 28.302

R(reflections)= 0.0405(3998) wR2(reflections)= 0.1000(5665)

S = 1.003 Npar= 309

NOTE FROM AUTHORS: Displacement of these atoms is perpendicular to the plane of the entire molecule. The shift in electron density due to the resonance ability of the azide group (N=N-N vs N-N=N) may also be a contributing factor, resulting in this alert. This is a common observation in other azides.

Alert level B

PLAT230_ALERT_2_B Hirshfeld Test Diff for N1B --N2B . 7.6 s.u.

Alert level C

PLAT230_ALERT_2_C Hirshfeld Test Diff for N1A --N2A . 6.9 s.u.

Alert level G

PLAT912_ALERT_4_G Missing # of FCF Reflections Above STh/L= 0.600 2 Note

PLAT969_ALERT_5_G The 'Henn et al.' R-Factor-gap value .... 4.18 Note

Predicted wR2: Based on SigI**2 2.39 or SHELX Weight 10.26 PLAT978_ALERT_2_G Number C-C Bonds with Positive Residual Density. 21 Info

---

Datablock: 22152

Bond precision:	C-C = 0.0043 A		Wavelength=0.71073
Cell:	a=15.838(3)	b=19.892(4)	c=7.3156(14)
	alpha=90	beta=92.814(3)	gamma=90
Temperature:	150 K		

	Calculated	Reported
Volume	2302.0(8)	2301.9(8)
Space group	P 21/c	P 21/c
Hall group	-P 2ybc	-P 2ybc
Moiety formula	C13 H13 N O3	C13 H13 N O3
Sum formula	C13 H13 N O3	C13 H13 N O3
Mr	231.24	231.24
Dx,g cm-3	1.334	1.334
Z	8	8
Mu (mm-1)	0.095	0.095
F000	976.0	976.0
F000'	976.50	
h,k,lmax	18,23,8	18,23,8
Nref	3922	3908
Tmin,Tmax	0.983,0.988	0.766,0.862
Tmin'	0.982	

Correction method= # Reported T Limits: Tmin=0.766 Tmax=0.862 AbsCorr = MULTI-SCAN

Data completeness= 0.996 Theta(max)= 24.713

R(reflections)= 0.0574(3051) wR2(reflections)= 0.1531(3908)

S = 1.190 Npar= 317

NOTE FROM AUTHORS: Data intensity declined rapidly beyond 0.95A resolution. Data out to 0.85A resolution used in the refinement.

Alert level C

THETM01_ALERT_3_C The value of sine(theta_max)/wavelength is less than 0.590 Calculated sin(theta_max)/wavelength = 0.5882

PLAT340_ALERT_3_C Low Bond Precision on C-C Bonds .... 0.00427 Ang.

PLAT906_ALERT_3_C Large K Value in the Analysis of Variance ...... 4.923 Check

PLAT911_ALERT_3_C Missing FCF Refl Between Thmin & STh/L= 0.588 14 Report

-8 1 1, -5 1 1, -4 1 1, -6 3 1, 7 3 1, 8 3 1, 3 4 1, 4 4 1, 5 4 1, 2 0 4, -1 1 4, -1 3 4, -4 5 5, 2 7 5,

Alert level G

PLAT909_ALERT_3_G Percentage of I>2sig(I) Data at Theta(Max) Still 52% Note

PLAT933_ALERT_2_G Number of HKL-OMIT Records in Embedded .res File 2 Note

2 7 5, -4 5 5,

PLAT969_ALERT_5_G The 'Henn et al.' R-Factor-gap value .... 6.13 Note

Predicted wR2: Based on SigI**2 2.50 or SHELX Weight 13.42

PLAT978_ALERT_2_G Number C-C Bonds with Positive Residual Density. 0 Info

Datablock: 18075

Bond precision:		C-C = 0.0021 A	Wavelength=0.71073
Cell:	a=9.9019(3)	b=20.0776(5)	c=10.4531(3)
	alpha=90	beta=104.4454(9)	gamma=90
Temperature:	150 K		

	Calculated	Reported
Volume	2012.44(10)	2012.44(10)
Space group	P 21/c	P 21/c
Hall group	-P 2ybc	-P 2ybc
Moiety formula	C12 H11 N O2	C12 H11 N O2
Sum formula	C12 H11 N O2	C12 H11 N O2
Mr	201.22	201.22
Dx,g cm-3	1.328	1.328
Z	8	8
Mu (mm-1)	0.091	0.091
F000	848.0	848.0
F000'	848.40	
h,k,lmax	11,24,12	11,24,12
Nref	3678	3674
Tmin,Tmax	0.987,0.991	0.780,0.862
Tmin'	0.984	

Correction method= # Reported T Limits: Tmin=0.780 Tmax=0.862 AbsCorr = NUMERICAL

Data completeness= 0.999 Theta(max)= 25.348

R(reflections)= 0.0373(3097) wR2(reflections)= 0.0933(3674)

S = 1.046 Npar= 337

Alert level G

PLAT910_ALERT_3_G Missing # of FCF Reflection(s) Below Theta(Min). 3 Note
1 0 0, 0 2 0, 0 1 1,
PLAT933_ALERT_2_G Number of HKL-OMIT Records in Embedded .res File 2 Note
0 1 1, 0 2 1,
PLAT969_ALERT_5_G The 'Henn et al.' R-Factor-gap value .... 3.35 Note
Predicted wR2: Based on SigI**2 2.79 or SHELX Weight 9.36
PLAT978_ALERT_2_G Number C-C Bonds with Positive Residual Density. 5 Info

PLATON version of 06/01/2024; check.def file version of 05/01/2024

Datablock 20093_150 - ellipsoid plot

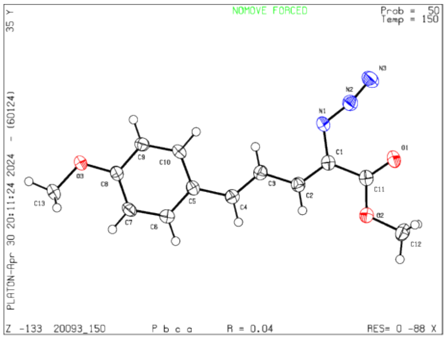

Datablock 20093_200 - ellipsoid plot

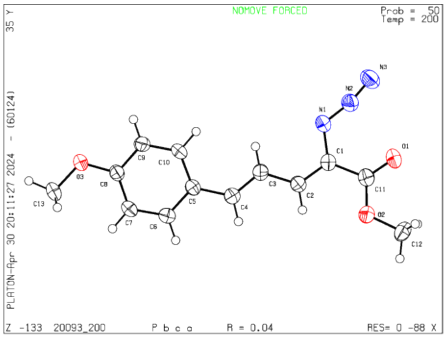

Datablock 20093_250 - ellipsoid plot

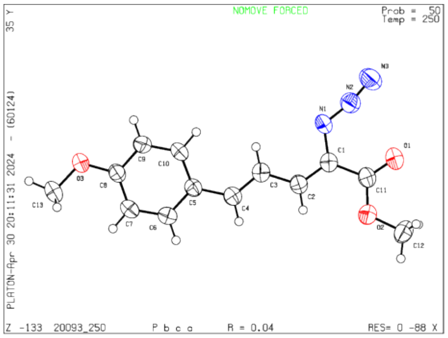

Datablock 20093_295 - ellipsoid plot

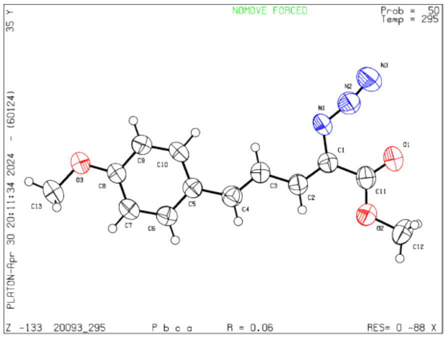

Datablock 18075c - ellipsoid plot

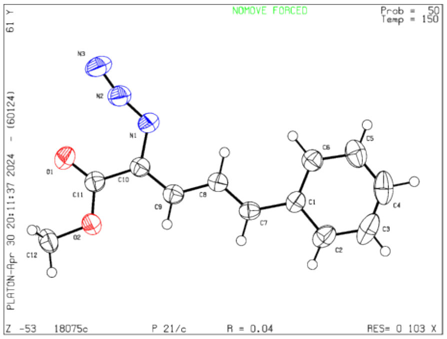

Datablock 22094 - ellipsoid plot

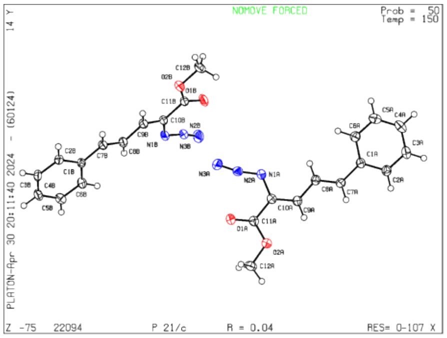

Datablock 22152 - ellipsoid plot

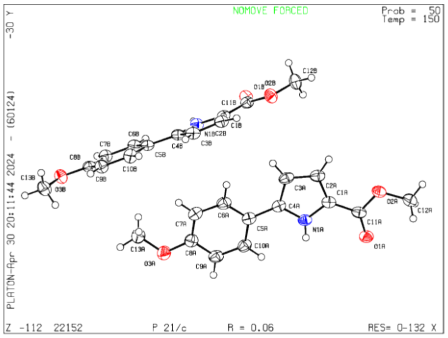

Datablock 18075 - ellipsoid plot

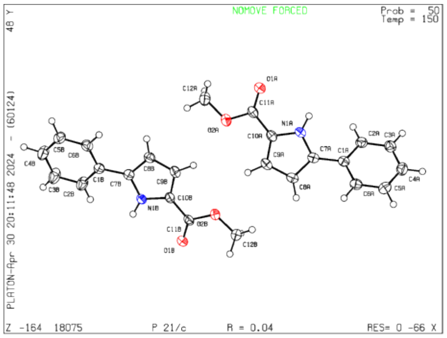

.

16. References

(1) a) Farney, E. P.; Yoon, T. P. Visible-Light Sensitization of Vinyl Azides by Transition-Metal Photocatalysis. *Angew. Chem. Int. Ed* **2014**, *53*, 793-797. DOI: 10.1002/anie.201308820 b) Dong, H.; Shen, M.; Redford, J. E.; Stokes, B. J.; Pumphrey, A. L.; Driver, T. G. Transition Metal-Catalyzed Synthesis of Pyrroles from Dienyl Azides. *Org. Lett.* **2007**, *9*, 5191-5194. DOI: 10.1021/ol702262f.

(2) Geist, B.; Knittel, D. Kathodische Reduktion Von 2-Azido-2,4-Pentadien-1-Onen Elektrolytische Untersuchungen an Vinylaziden, 9. Mitt. *Monatsh. Chem.* **1988**, *119*, 571-581. DOI: 10.1007/BF00809209.

(3) O'Brien, A. G.; Lévesque, F.; Seeberger, P. H. Continuous Flow Thermolysis of Azidoacrylates for the Synthesis of Heterocycles and Pharmaceutical Intermediates. *Chem. Comm.* **2011**, *47*, 2688-2690. DOI: 10.1039/C0CC04481D.

(4) a) Lee, C.; Yang, W.; Parr, R. G. Development of the Colle-Salvetti Correlation-Energy Formula into a Functional of the Electron Density. *Phys. Rev. B* **1988**, *37*, 785-789. DOI: 10.1103/PhysRevB.37.785 b) Frisch, M. J.; Trucks, G. W.; Schlegel, H. B.; Scuseria, G. E.; Robb, M. A.; Cheeseman, J. R.; Scalmani, G.; Barone, V.; Petersson, G. A.; Nakatsuji, H.; Li, X.; Caricato, M.; Marenich, A. V.; Bloino, J.; Janesko, B. G.; Gomperts, R.; Mennucci, B.; Hratchian, H. P.; Ortiz, J. V.; Izmaylov, A. F.; Sonnenberg, J. L.; Williams; Ding, F.; Lipparini, F.; Egidi, F.; Goings, J.; Peng, B.; Petrone, A.; Henderson, T.; Ranasinghe, D.; Zakrzewski, V. G.; Gao, J.; Rega, N.; Zheng, G.; Liang, W.; Hada, M.; Ehara, M.; Toyota, K.; Fukuda, R.; Hasegawa, J.; Ishida, M.; Nakajima, T.; Honda, Y.; Kitao, O.; Nakai, H.; Vreven, T.; Throssell, K.; Montgomery Jr., J. A.; Peralta, J. E.; Ogliaro, F.; Bearpark, M. J.; Heyd, J. J.; Brothers, E. N.; Kudin, K. N.; Staroverov, V. N.; Keith, T. A.; Kobayashi, R.; Normand, J.; Raghavachari, K.; Rendell, A. P.; Burant, J. C.; Iyengar, S. S.; Tomasi, J.; Cossi, M.; Millam, J. M.; Klene, M.; Adamo, C.; Cammi, R.; Ochterski, J. W.; Martin, R. L.; Morokuma, K.; Farkas, O.; Foresman, J. B.; Fox, D. J. Wallingford, CT, 2016 c) Chai, J.-D.; Head-Gordon, M. Long-Range Corrected Hybrid Density Functionals with Damped Atom–Atom Dispersion Corrections. *Phys. Chem. Chem. Phys.* **2008**, *10*, 6615-6620. DOI: 10.1039/B810189B.

(5) a) Bauernschmitt, R.; Ahlrichs, R. Treatment of Electronic Excitations within the Adiabatic Approximation of Time Dependent Density Functional Theory. *Chem. Phys. Lett.* **1996**, *256*, 454-464. DOI: https://doi.org/10.1016/0009-2614(96)00440-X b) Stratmann, R. E.; Scuseria, G. E.; Frisch, M. J. An Efficient Implementation of Time-Dependent Density-Functional Theory for the Calculation of Excitation Energies of Large Molecules. *J. Chem. Phys.* **1998**, *109*, 8218-8224. DOI: 10.1063/1.477483.

(6) Gonzalez, C.; Schlegel, H. B. An Improved Algorithm for Reaction Path Following. *J. Chem. Phys.* **1989**, *90*, 2154-2161. DOI: 10.1063/1.456010.

(7) Turner, M. J.; Thomas, S. P.; Shi, M. W.; Jayatilaka, D.; Spackman, M. A. Energy Frameworks: Insights into Interaction Anisotropy and the Mechanical Properties of Molecular Crystals. *Chem. Comm.* **2015**, *51*, 3735-3738. DOI: 10.1039/C4CC09074H.

(8) Dolomanov, O. V.; Bourhis, L. J.; Gildea, R. J.; Howard, J. a. K.; Puschmann, H. OLEX2: A Complete Structure Solution, Refinement and Analysis Program. *J. Appl. Crystallogr* **2009**, *42*, 339-341. DOI: doi:10.1107/S0021889808042726.

(9) Sheldrick, G. SHELXL - Integrated Space-Group and Crystal-Structure Determination. *Acta Crystallogr. A* **2015**, *71*, 3-8. DOI: doi:10.1107/S2053273314026370.

(10) Sheldrick, G. M. Crystal Structure Refinement with SHELXL. *Acta Crystallogr. C,* **2015**, *71*, 3-8. DOI: 10.1107/s2053229614024218.
